# Supplementary material for: Sixteen isostructural phosphonate metal-organic frameworks with controlled Lewis acidity and chemical stability for asymmetric catalysis
Source: Nat Commun. 2017 Dec 19;8:2171. doi: 10.1038/s41467-017-02335-0 (PMC5736719; doi:10.1038/s41467-017-02335-0)
Supplement: Supplementary file 1 — Description of Additional Supplementary Files [file 41467_2017_2335_MOESM1_ESM.pdf]

Description of Additional Supplementary Files

File Name: Supplementary Data 1
Description: Cif combine

File Name: Supplementary Data 2
Description: Checkcif combine
