# Supplementary material for: Sixteen isostructural phosphonate metal-organic frameworks with controlled Lewis acidity and chemical stability for asymmetric catalysis
Source: Nat Commun. 2017 Dec 19;8:2171. doi: 10.1038/s41467-017-02335-0 (PMC5736719; doi:10.1038/s41467-017-02335-0)
Supplement: Supplementary file 3 — Supplementary Information [file 41467_2017_2335_MOESM3_ESM.pdf]

Supplementary Methods

Synthesis of the ligand

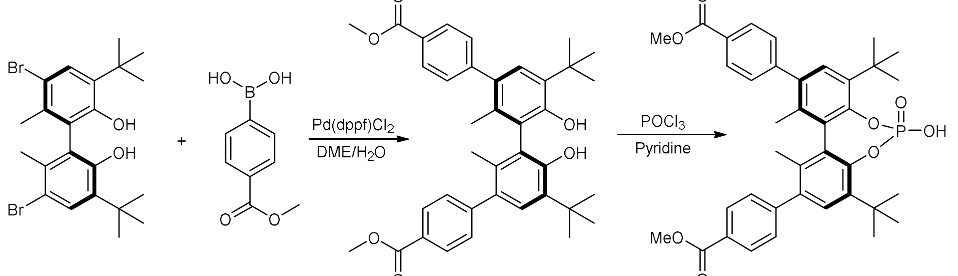

S1 S2 (*S*)-Me_2L

(*S*)-3,3'-di-*tert*-butyl-5,5'-dibromo-6,6'-dimethylbiphenyl-2,2'-diol (**S1**, 2 g, 4.15 mmol), 4-(methoxycarbonyl)phenylboronic acid (2.25 g, 12.45 mmol), and K_2CO_3 (6.0 g, 41.5 mmol) in DME/H_2O (2/1, 60 mL) was degassed for 10 mins. Pd(dppf)Cl_2 (100 mg, 0.12 mmol) was added, and the suspension was stirred under N_2 for 10 h at 95 ^oC. After cooling, the mixture was concentrated and extracted with DCM. The brown solution was washed with brine, dried over Na_2SO_4, and then the solvent was removed under reduced pressure. The brown solid was purified by silica gel column chromatography with petroleum ether/DCM (5:1 v/v) as the eluent affording (S)-3,3'-di-*tert*-butyl-5,5'-di(4-methoxycarbonylphenyl)-6,6'-dimethylbiphenyl-2,2'-diol. Yield: (**S2**, 1.8 g, 73%). ^{1}H NMR ($CDCl_3$) δ: 8.08-8.10 (d, 4H, ArH), 7.42-7.44 (d, 4H, ArH), 7.26 (s, 2H, ArH), 5.17 (s, 2H, OH), 3.94 (s, 6H, OCH_3), 1.88 (s, 6H, CH_3), 1.44 (s, 18H, $C(CH_3)_3$) . ^{13}C NMR ($CDCl_3$) δ: 167.29, 152.28, 147.30, 134.55, 134.07, 133.35, 129.91, 129.64, 129.53, 128.54, 121.31, 52.34, 34.97, 29.71, 17.50. FTIR (KBr, cm^{-1}): 3443(m), 2953(w), 2866(w), 1722(s), 1609(m), 1439(m), 1416(w), 1364(w), 1278(s), 1189(m), 1175(m), 1105(m), 1073(w), 1017(w), 961(w), 860(w), 779(w), 713(w), 604(w), 581(w), 516(w).

In a two-neck, 50 mL flame-dried flask, (*S*)-3,3'-di-*tert*-butyl-5,5'-di(4-methoxy carbonylphenyl)-6,6'- dimethylbiphenyl-2,2'-diol (**S2,** 1.8 g, 3 mmol) was added under N_2. Anhydrous pyridine (10 mL) was added, followed by addition of phosphorus oxychloride (0.6 mL, 6.0 mmol), and the solution was stirred under N_2 for 24 h at 95 ^oC. After cooling to ambient temperature, deionized water (2.5 mL) was added, and the mixture was heated to 95 ^oC for 5 h. Upon cooling, the reaction mixture was slowly poured into ice-water and acidified to a pH of ~2 and extracted with DCM, The organic layer was washed with brine, dried over Na_2SO_4 and evaporated under reduced pressure to give a brown solid. The crude product was

purified by column chromatography on silica gel (CH_2Cl_2/MeOH, 10:1, v/v) to give (*S*)-3,3'-di-*tert*-butyl-5,5'-di(4-methoxycarbonyl phenyl)-6,6'-dimethylbiphenyl-2,2'-hydrogen phosphate (**Me_2L**, 1.57 g, 80%) as a white solid. ^1H NMR ($CDCl_3$) δ: 8.08 (d, J = 8.3 Hz, 4H), 7.36 (d, J = 8.2 Hz, 4H), 7.25 (s, 2H), 3.94 (s, 6H), 1.89 (s, 6H), 1.50 (s, 18H). ^{13}C NMR ($CDCl_3$) δ: 166.97, 146.84, 146.71, 142.76, 138.58, 138.54, 133.90, 129.51, 129.00, 128.76, 127.15, 52.17, 35.10, 31.40, 18.15. ^{31}P NMR ($CDCl_3$) δ: -2.28.

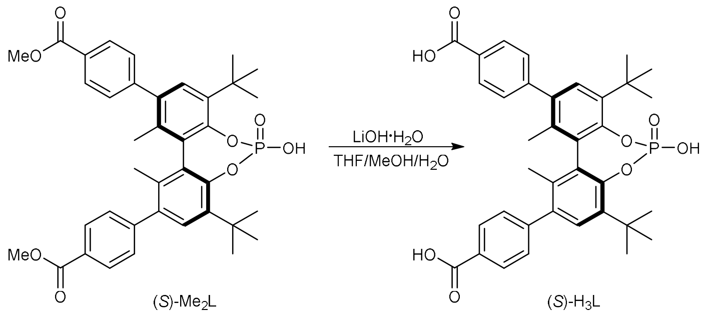

A solution of **Me_2L** (1.57 g, 2.40 mmol) and $LiOH•H_2O$ (1.01 g, 24 mmol) in THF (10 mL), MeOH (30 mL) and H_2O (30 mL) was heated to reflux and reacted for 8 h. After that, the solution was cooled to room temperature, acidified to pH = 3~4 with 2 M HCl, filtered and washed with distilled water. White solid was obtained (***S***)-H_3**L**. Yield: (1.28 g, 85%). ^1H NMR(DMSO-d_6) δ: 7.97-7.99 (d, 4H, ArH), 7.48-7.50 (d, 4H, ArH), 7.24 (s, 2H, ArH), 1.86 (s, 6H, CH_3), 1.43 (s, 18H, $C(CH_3)_3$). ^{13}C NMR (DMSO-d_6) δ: 167.79, 147.51, 146.37, 138.83, 138.22, 134.11, 130.33, 130.25, 130.13, 129.98, 129.24, 35.39, 31.75, 18.66. ^{31}P NMR (DMSO-d_6) δ: 1.51. FTIR (KBr pellet): 3431(m), 2962(m), 2875(w), 2650(w), 2530(w), 1699(s), 1610(m), 1567(w), 1415(m), 1264(m), 1225(s), 1179(w), 1093(w), 1039(w), 1019(w), 907(w), 850(w), 875(w), 784(w), 728(w), 691(w), 611(w), 543(w). ESI-MS: m/z 627.26 (Calcd *m/z* 627.26 for [**L**-H]$^-$).

Synthesis of 1-Mg/Ca/Mn/Co/Ni/Cu/Zn/Pd/Pb

A mixture of $Mg(NO_3)_2·6H_2O$ ($Ca(NO_3)_2·4H_2O$/$MnCl_2·6H_2O$/$Co(NO_3)_2·6H_2O$/$Ni(NO_3)_2·6H_2O$/$Cu(NO_3)_2·6H_2O$/$Zn(NO_3)_2·6H_2O$/$PdCl_2$/$Pb(NO_3)_2·6H_2O$, 0.04 mmol), H_3**L** (20 mg, 0.03 mmol), MeOH (5 mL) and HOAc (0.5mL) was sealed in a 10 mL vial with a screw cap and heated at 80 ^oC for 1 day. The mixture was cooled to room temperature, then block-like crystals were obtained, washed with ether and dried in air.

Yield: 80%, 75%, 72%, 76%, 72%, 68%, 77%, 82% and 61% for **1-Mg/Ca/Mn/Co/Ni/Cu/Zn/Pd/Pb** (based on metal salts), respectively.

1-Mg: Anal (%). Calcd for $[Mg_3\mathbf{L}_2(H_2O)_2]\cdot 2CH_3OH\cdot 2H_2O/C_{74}H_{84}Mg_3O_{22}P_2$: C, 60.86; H, 5.80. Found: C, 59.82; H, 5.73. FTIR (KBr pellet): 3417(m), 2962(m), 2922(w), 2874(w), 1608(s), 1547(m), 1407(s), 1249(m), 1239(m), 1183(w), 1105(s), 1072(w), 1040(m), 986(w), 878(w), 851(w), 793(w), 728(w), 694(w), 620(w), 551(w).

1-Ca: Anal (%). Calcd for $[Ca_3\mathbf{L}_2(H_2O)_2]\cdot 2CH_3OH\cdot 2H_2O/C_{74}H_{84}Ca_3O_{22}P_2$: C, 58.95; H, 5.62. Found: C, 57.88; H, 5.57. FTIR (KBr pellet): 3409(m), 3228(w), 2961(m), 2873(w), 1608(s), 1544(m), 1405(s), 1235(m), 1183(w), 1102(s), 1040(m), 1020(w), 986(w), 910(w), 874(w), 850(w), 792(w), 728(w), 695(w), 620(w), 551(w).

1-Mn: Anal (%). Calcd for $[Mn_3\mathbf{L}_2(H_2O)_2]\cdot 2CH_3OH\cdot 2H_2O/C_{74}H_{84}Mn_3O_{22}P_2$: C, 57.26; H, 5.45. Found: C, 56.31; H, 5.38. FTIR (KBr pellet): 3423(m), 2962(m), 2931(w), 2873(w), 1609(s), 1549(m), 1406(s), 1250(m), 1235(m), 1183(w), 1105(s), 1071(w), 1040(m), 985(w), 911(w), 880(w), 850(w), 792(w), 728(w), 694(w), 620(w), 551(w).

1-Co: Anal (%). Calcd for $[Co_3\mathbf{L}_2(H_2O)_2]\cdot 2CH_3OH\cdot 2H_2O/C_{74}H_{84}Co_3O_{22}P_2$: C, 56.82; H, 5.41. Found: C, 55.90; H, 5.38. FTIR (KBr pellet): 3419(m), 2962(m), 2926(w), 2874(w), 1608(s), 1546(m), 1405(s), 1236(m), 1183(w), 1104(s), 1040(m), 986(w), 910(w), 877(w), 851(w), 792(w), 728(w), 694(w), 620(w), 551(w).

1-Ni: Anal (%). Calcd for $[Ni_3\mathbf{L}_2(H_2O)_2]\cdot 2CH_3OH\cdot 4H_2O/C_{74}H_{88}Ni_3O_{24}P_2$: C, 55.57; H, 5.55. Found: C, 54.87; H, 5.48. FTIR (KBr pellet): 3425(m), 2962(m), 2926(w), 2875(w), 1608(s), 1548(m), 1406(s), 1238(m), 1182(w), 1103(s), 1040(m), 985(w), 910(w), 874(w), 850(w), 792(w), 728(w), 694(w), 620(w), 551(w).

1-Cu: Anal (%). Calcd for $[Cu_3\mathbf{L}_2(H_2O)_2]\cdot 2CH_3OH\cdot 2H_2O/C_{74}H_{84}Cu_3O_{22}P_2$: C, 56.32; H, 5.37. Found: C, 55.53; H, 5.33. FTIR (KBr pellet): 3420(m), 2962(m), 2931(w), 2875(w), 1609(s), 1548(m), 1405(s), 1235(m), 1182(w), 1104(s), 1040(m), 1021(w), 986(w), 910(w), 879(w), 851(w), 792(w), 728(w), 694(w), 620(w), 551(w).

1-Zn: Anal (%). Calcd for $[Zn_3\mathbf{L}_2(H_2O)_2]\cdot 2CH_3OH\cdot 2H_2O/C_{74}H_{84}O_{22}P_2Zn_3$: C, 56.13; H, 5.35. Found: C, 55.16; H, 5.30. FTIR (KBr pellet): 3410(m), 2962(m), 2926(w), 2873(w), 1609(s), 1549(m), 1407(s), 1242(m), 1183(w), 1107(s), 1040(m), 986(w), 911(w), 880(w), 851(w), 793(w), 728(w), 694(w), 620(w), 551(w).

1-Pb: Anal (%). Calcd for $[Pb_3\mathbf{L}_2(H_2O)_2]\cdot 2CH_3OH\cdot 3H_2O/C_{74}H_{86}O_{23}P_2Pb_3$: C, 43.05; H, 4.27. Found: C, 43.92; H, 4.32. FTIR (KBr pellet): 3405(m), 2962(m), 2926(w),

2870(w), 1608(s), 1542(m), 1409(s), 1234(m), 1182(w), 1106(s), 1040(m), 985(w), 884(w), 851(w), 792(w), 728(w), 694(w), 620(w), 551(w).

1-Pd: Anal (%). Calcd for [Pd_3**L**$_2(H_2O)_2$]·$2CH_3OH$·$3H_2O$/$C_{74}H_{86}O_{23}P_2Pd_3$: C, 51.53; H, 5.03. Found: C, 50.72; H, 4.97. FTIR (KBr pellet): 3393(m), 2963(m), 2875(w), 1609(s), 1542(m), 1400(s), 1239(m), 1182(w), 1106(s), 1040(m), 986(w), 910(w), 881(w), 851(w), 792(w), 728(w), 694(w), 621(w), 550(w).

Synthesis of 1-Cr/Fe/Al/Ga

A mixture of $Cr(acac)_3$ ($Fe(acac)_3$/$AlCl_3$/$GaCl_3$, 0.04 mmol), H_3**L** (20 mg, 0.03 mmol), MeOH (1 mL) and HOAc (0.5 mL) was sealed in a 10 mL vial with a screw cap and heated at 80 ^{o}C for 2 days. The mixture was cooled to room temperature, then block-like crystals were obtained, washed with ether and dried in air. Yield: 80%, 68%, 60% and 80% (based on metal salts) for **1-Cr/Fe/Al/Ga**, respectively.

1-Cr: Anal (%). Calcd for [Cr_3**L**$_2$(OAc)(H_2O)][OAc]$_2$·$4H_2O$/$C_{78}H_{87}Cr_3O_{27}P_2$: C, 55.95; H, 5.24. Found: C, 55.12; H, 5.30. FTIR (KBr pellet): 3409(m), 3219(m), 2963(m), 2875(m), 1609(s), 1550(m), 1406(s), 1240(m), 1180(w), 1106(s), 1040(m), 985(w), 882(m), 851(m), 792(m), 728(m), 695(w), 621(w), 551(w).

1-Fe: Anal (%). Calcd for [Fe_3**L**$_2$(OAc)(H_2O)][OAc]$_2$·$4H_2O$/$C_{78}H_{87}Cr_3O_{27}P_2$: C, 55.57; H, 5.20. Found: C, 54.62; H, 5.11. FTIR (KBr pellet): 3419(m), 2961(w), 2924(w), 2875(w), 1608(s), 1544(m), 1404(s), 1240(m), 1178(w), 1102(s), 1039(m), 985(w), 911(w), 873(w), 850(w), 793(w), 728(w), 695(w), 620(w), 551(w).

1-Al: Anal (%). Calcd for [Al_3**L**$_2$(OAc)(H_2O)][OAc]$_2$·$3H_2O$/$C_{78}H_{85}Al_3O_{26}P_2$: C, 59.24; H, 5.42. Found: C, 58.42; H, 5.35. FTIR (KBr pellet): 3415(m), 2962(m), 2922(w), 2874(w), 1609(s), 1548(m), 1405(s), 1251(m), 1240(m), 1183(w), 1105(s), 1071(w), 1040(m), 986(w), 911(w), 879(w), 850(w), 792(w), 728(w), 694(w), 620(w), 550(w).

1-Ga: Anal (%). Calcd for [Ga_3**L**$_2$(OAc)(H_2O)][OAc]$_2$·$4H_2O$/$C_{78}H_{85}Ga_3O_{26}P_2$: C, 54.80; H, 5.01. Found: C, 53.76; H, 4.97. FTIR (KBr pellet): 3409(m), 2962(w), 2873(w), 1608(s), 1544(m), 1405(s), 1235(m), 1183(w), 1102(s), 1072(w), 1040(m), 1020(w), 986(w), 911(w), 874(w), 850(w), 792(w), 728(w), 695(w), 620(w), 551(w).

Synthesis of 1-Zr/Ti/Sn

A mixture of $Zr(NO_3)_4$·$5H_2O$/(Ti(O-*i*Pr)$_4$/$SnCl_4$·$6H_2O$, 0.04 mmol), H_3**L** (20 mg, 0.03 mmol, MeOH (3 mL) and HOAc (0.5 mL) was sealed in a 10 mL vial with a screw cap and heated at 80 ^{o}C for 2 days. The mixture was cooled to room

temperature, then yellow block-like crystals were obtained, washed with ether and dried in air. Yield: 70%, 70% and 52% for **1-Zr/Ti/Sn** (based on metal salts), respectively.

1-Zr: Anal (%). Calcd for $[Zr_3L_2(OAc)(H_2O)][OAc]_2[OH]_3\cdot3H_2O/C_{78}H_{88}O_{29}P_2Zr_3$: C, 51.33; H, 4.86. Found: C, 50.82; H, 4.81. FTIR (KBr pellet): 3424(m), 2962(w), 2931(w), 2875(w), 1608(s), 1544(m), 1405(s), 1237(m), 1178(w), 1102(s), 1040(m), 987(w), 875(w), 851(w), 793(w), 728(w), 695(w), 619(w), 550(w).

1-Ti: Anal (%). Calcd for $[Ti_3\mathbf{L}_2(OAc)(H_2O)][OAc]_2[OH]_3\cdot3H_2O/C_{78}H_{88}O_{29}P_2Ti_3$: C, 55.27; H, 5.23. Found: C, 54.62; H, 5.17. FTIR (KBr pellet): 3417(m), 2962(w), 2931(w), 2874(w), 1609(s), 1547(m), 1407(s), 1251(m), 1238(m), 1183(w), 1105(s), 1072(w), 1040(m), 986(w), 911(w), 879(w), 851(w), 792(w), 728(w), 694(w), 620(w), 552(w).

1-Sn: Anal (%). Calcd for $[Sn_3\mathbf{L}_2(OAc)(H_2O)][OAc]_2[OH]_3\cdot3H_2O/C_{78}H_{88}O_{29}P_2Sn_3$: C, 49.11; H, 4.65. Found: C,48.32; H, 4.57. FTIR (KBr pellet): 3412(m), 2962(m), 2931(w), 2873(w), 1609(s), 1549(m), 1405(s), 1243(m), 1183(w), 1106(s), 1040(m), 986(w), 911(w), 879(w), 851(w), 793(w), 728(w), 694(w), 620(w), 551(w).

Synthesis of Me_2L-Cr/Mg/Mn

A mixture of $Cr(acac)_3$ ($Mg(NO_3)_2\cdot6H_2O/MnCl_2\cdot6H_2O$, 0.10 mmol), Me_2**L** (0.10 mmol) and DCE (3 mL) was sealed in a 10 mL bottle and stirred at room temperature for 1 h. The mixture was then evaporated to get the homogeneous catalyst. Yield: 80%, 85% and 88% for **Me_2L-Cr/Mg/Mn**, respectively.

Crystallization of Me_2L-Mg/Mn/Cr for X-ray Structural Analyse: Me_2L-Mg (3 mg) was dissolved in diethyl ether (2 mL) and vaporize at 16 ^oC. After 6 h, colorless plate-like crystal was obtained.

Me_2L-Mn (3 mg) was dissolved in isopropyl ether/diethylether (1/1; 2 mL) and vaporize at 16 ^oC. After 12 h, colorless plate-like crystal was obtained.

Me_2L-Cr (3 mg) was dissolved in DME (2 mL) and vaporize at 16 ^oC. After 24 h, small green plate-like crystal was obtained.

Single-crystal X-ray diffraction showed that both Me_2**L-Mn** and Me_2**L-Mg** have a similar trimetal structure. However, the diffraction data for Me_2**L-Cr** had extremely weak diffraction, but cell parameter determination showed that it has a isostructural structure, which was given in Supplementary Table 1

The structure of Me_2**L-Mn**/**Mg**: The complex crystallizes in the chiral space group $P2_12_12_1$. The structure has a trimeric M unit, which is linked together by six bridging phosphonate groups of six Me_2L. The central metal is coordinated by six oxygen atoms from two bridging phosphonate groups and four water molecules, and each terminal metal is coordinated by two water and three oxygen atoms from three phosphonate groups. Adjacent metal ions are linked by one phosphonate group.

Me₂L-Cr: Anal (%). Calcd for $[Cr_3(\mathbf{Me_2L})_6(H_2O)_5][OH]_3\cdot 3H_2O/C_{228}H_{258}O_{58}P_6Cr_3$: C, 64.15; H, 6.05. Found: C, 64.38; H, 5.84. ICP-OES analysis showed that the P/Cr ratio was ~1.9:1. FTIR (KBr pellet): 3434(m), 2953(m), 2925(m), 2869(w), 1725(s), 1609(m), 1566(w), 1435(m), 1417(w), 1365(w), 1275(s), 1230(w), 1179(w), 1113(m), 1101(s), 1038(m), 1020(m), 982(w), 892(w), 850(m), 817(w), 779(m), 727(w), 713(m), 617(w), 552(w), 526(w). a = 22.25, b = 34.49, c = 37.26, $\alpha = \beta = \gamma = 90^o$, V= 28597, Orthorhombic P.

Me₂L-Mg: Anal (%). Calcd for $[Mg_3(\mathbf{Me_2L})_6(H_2O)_8]\cdot 4H_2O/C_{228}H_{264}O_{60}P_6Mg_3$: C, 64.83; H, 6.26;. Found: C, 65.43; H, 6.02. ICP-OES analysis showed that the P/Mg ratio was ~2.2:1. FTIR (KBr pellet): 3419(m), 2954(s), 2925(s), 2869(m), 1725(s), 1609(m), 1567(w), 1482(w), 1435(s), 1417(w), 1396(m), 1365(m), 1276(s), 1238(w), 1218(w), 1180(m), 1114(m), 1101(s), 1070(w), 1039(m), 1020(m), 985(w), 910(w), 870(m), 849(s), 825(w), 779(m), 727(m), 714(m),613(w), 550(m).

Me₂L-Mn: Anal (%). Calcd for $[Mn_3(\mathbf{Me_2L})_6\ (H_2O)_8]\cdot 3H_2O/C_{228}H_{262}O_{59}P_6Mn_3$: C, 63.72; H, 6.10;. Found: C,64.02; H, 5.89. ICP-OES analysis showed that the P/Mn ratio was ~2.3:1. FTIR (KBr pellet): 3429(m), 2954(s), 2925(s), 2869(m), 1725(s), 1609(m), 1567(w), 1434(s), 1417(w), 1396(w), 1365(w), 1276(s), 1235(w), 1217(w), 1179(m), 1101(s), 1072(w), 1039(m), 1020(m), 985(w), 873(w), 850(m), 817(w), 779(m), 757(w), 727(w), 713(m), 613(w), 549(m), 465(w).

Synthesis of H_3L-Cr/Mg/Mn

A mixture of $Cr(acac)_3$ ($Mg(NO_3)_2\cdot 6H_2O/MnCl_2\cdot 6H_2O$, 0.15 mmol), H_3**L** (0.10 mmol) and DCE (3 mL) was sealed in a 10 mL bottle and stirred at room temperature for 1 h. The solid comes out and then filtered to get the catalyst which cannot dissolve in DCE. Quantitative yield: for H_3**L-Cr/Mg/Mn**, respectively.

Efforts in getting single-crystal of H_3**L-Cr/Mg/Mn** resulted in the related MOFs **1-Cr/Mg/Mn**, and we have obtained the parameter of unit cell (Supplementary Table 1).

H_3L-Cr: Anal (%). Calcd for $[Cr_3\mathbf{L}_2(OH)(H_2O)][OH]_2\cdot4H_2O/C_{72}H_{81}Cr_3O_{24}P_2$: C, 55.85; H, 5.24. Found: C, 56.12; H, 5.03. ICP-OES analysis showed that the P/Cr ratio was ~1:1.7. FTIR (KBr pellet): 3414(m), 2963(m), 2873(w), 1663(w), 1614(s), 1547(m), 1408(s), 1385(s), 1251(s), 1235(s), 1184(m), 1107(s), 1040(m), 1020(w), 986(w), 911(w), 881(m), 851(m), 793(m), 728(m), 693(w), 621(m), 551(w).

H_3L-Mg: Anal (%). Calcd for $[Mg_3\mathbf{L}_2(H_2O)_2]\cdot6H_2O/C_{72}H_{80}Mg_3O_{22}P_2$: C, 61.89; H, 6.01;. Found: C, 60.53; H, 5.23. ICP-OES analysis showed that the P/Mg ratio was ~1:1.6. FTIR (KBr pellet): 3429(m), 2962(m), 2872(w), 1610(s), 1551(m), 1411(s), 1236(s), 1183(w), 1105(s), 1071(w), 1039(m), 1020(w), 988(w), 912(w), 878(m), 851(m), 793(m), 728(m), 694(w), 621(w), 552(w)

H_3L-Mn: Anal (%). Calcd for $[Mn_3\mathbf{L}_2(H_2O)_2]\cdot4H_2O/C_{74}H_{84}Mn_3O_{22}P_2$: C, 56.73; H, 5.25;. Found: C,55.83; H, 5.44. ICP-OES analysis showed that the P/Mn ratio was ~1:1.3. FTIR (KBr pellet): 3468(m), 3414(m), 2959(m), 2924(m), 2872(w), 1615(s), 1547(m), 1407(s), 1385(m), 1237(m), 1183(w), 1108(s), 1073(w), 1040(m), 1020(w), 986(w), 912(w), 878(m), 850(m), 793(w), 728(w), 694(w), 621(w), 552(w).

Study of MOFs chemical stabilities

The stability of the pristine framework, evacuated sample and sample soaked in different solvents were confirmed by PXRD using **1-**Cr/Mg/Mn/Zr as examples.

PXRD、BET and N_2 adsorption curves of the four MOFs after heating in methanol, toluene, DCE, water, and aqueous HCl (pH = 4) and NaOH solutions (pH = 9) for 7 days showed almost identical features, which support the framework rigidity during the experiments.

The slightly splitting and shifting of some peaks are explained by partial flexibility of the framework after the guest exchange.

General procedure for asymmetric catalysis

Prior to catalysis, the MOFs should be activated: As-synthesized MOFs were exchanged with fresh methanol three times and then evacuated at 100 °C for two hours.

The method used for calculating amount of MOF and metal.

[Amount of MOF] = [Weight of MOF] / [Molecular Weight of MOF]

For example **1-Cr**; the molecular formula is $[Cr_3\mathbf{L}_2(OAc)(H_2O)][OAc]_2\cdot 4H_2O/C_{78}H_{87}Cr_3O_{27}P_2$ and the Molecular Weight of **1-Cr** is ~1673. When the weight of **1-Cr** is 3.4 mg, the amount of **MOF** is

(3.4 mg) / (1673) = 0.002 mol

There are three Cr ions and two **L** in the molecular formula, so the Amount of metal catalyst was: 0.002*3=0.006 mmol. the Amount of **L** was: 0.002*2=0.04 mmol

Allylboration of aldehydes with 1-Cr

To a 10 mL flame-dried Schlenk tube, the activated **1-Cr** (2 mol%, catalyst based on MOF), aldehydes (0.1 mmol) and dry DCE (0.5 mL) were added. The reaction mixture was stirred for 1 h and then cooled to -10 ^{o}C followed by dropwise addition of dry DCE (0.3 mL) containing allylboronic acid pinacol ester (0.12 mmol). The mixture was stirred for 12 h at this temperature and then purified by flash chromatography using EtOAc and petroleum ether.

The control reaction catalyzed by **Me_2L-Cr** or **H_3L-Cr** were performed according to the following procedure:

To a 10 mL flame-dried Schlenk tube, Me_2**L-Cr** or H_3**L-Cr** (6 mol% catalyst based on Cr) aldehydes (0.1 mmol) and dry DCE (0.5 mL) were added. The reaction mixture was stirred for 1 h and then cooled to -10 ^{o}C followed by dropwise addition of dry DCE (0.3 mL) containing allylboronic acid pinacol ester (0.12 mmol). The mixture was stirred for 12 h at this temperature and then purified by flash chromatography using EtOAc and petroleum ether.

The homogeneous control reaction catalyzed by Me_2**L** or H_3**L** was performed according to the following procedure:

To a flame-dried Schlenk tube was added with Me_2**L** (0.004 mmol) or H_3**L** (0.004 mmol), aldehydes (0.1 mmol) and dry DCE (0.5 mL). The reaction mixture was stirred for 1 h and then cooled to -10 ^{o}C followed by dropwise addition of dry DCE (0.3 mL) containing allylboronic acid pinacol ester (0.12 mmol). The mixture was stirred for 12 h at this temperature and then purified by flash chromatography using EtOAc and petroleum ether.

Propargylation of aldehydes with 1-Cr

To a 10 mL flame-dried Schlenk tube, the activated **1-Cr** (2 mol%, catalyst based on MOF), aldehydes (0.1 mmol) and dry DCE (0.5 mL) were added. The reaction

mixture was stirred for about 1 h and then cooled to -10 °C followed by dropwise addition of dry DCE (0.3 mL) containing allenylboronic acid pinacol ester (0.15 mmol). The mixture was stirred for 12 h at this temperature and then purified by flash chromatography using EtOAc and petroleum ether.

The control reaction catalyzed by **Me_2L-Cr** or **H_3L-Cr** was performed according to the following procedure:

To a 10 mL flame-dried Schlenk tube, Me_2**L-Cr** or H_3**L-Cr** (6 mol% catalyst based on Cr), aldehydes (0.1 mmol) and dry DCE (0.5 mL) were added. The reaction mixture was stirred for 1 h and then cooled to -10 °C followed by dropwise addition of dry DCE (0.3 mL) containing allenylboronic acid pinacol ester (0.15 mmol). The mixture was stirred for 12 h at this temperature and then purified by flash chromatography using EtOAc and petroleum ether.

The homogeneous control reaction catalyzed by Me_2**L** or H_3**L** was performed according to the following procedure:

To a flame-dried Schlenk tube was added with Me_2**L** (0.004 mmol) or H_3**L** (0.004 mmol), aldehydes (0.1 mmol) and dry DCE (0.5 mL). The reaction mixture was stirred for 1 h and then cooled to -10 °C followed by dropwise addition of dry DCE (0.3 mL) containing allenylboronic acid pinacol ester (0.15 mmol). The mixture was stirred for 12 h at this temperature and then purified by flash chromatography using EtOAc and petroleum ether.

Friedel-Crafts alkylation catalyzed by 1-Mg

To a 10 mL flame-dried Schlenk tube, the activated **1-Mg** (2 mol%, catalyst based on MOF), pyrrole (0.3 mmol) and dry DCE (0.3 mL) were added. The reaction mixture was stirred for 1 h and then cooled to -10 °C followed by dropwise addition of dry DCE (0.5 mL) containing nitroalkenes (0.1 mmol). The mixture was stirred for 12 h at this temperature and then purified by flash chromatography using EtOAc and petroleum ether.

The control reaction catalyzed by **Me_2L-Mg** or **H_3L-Mg** was performed according to the following procedure:

To a 10 mL flame-dried Schlenk tube, Me_2**L-Mg** or H_3**L-Mg** (6 mol% catalyst based on Mg)), pyrrole (0.3 mmol) and dry DCE (0.3 mL) were added. The reaction mixture was stirred for 1 h and then cooled to -10 °C followed by dropwise addition of of dry DCE (0.5 mL) containing nitroalkenes (0.1 mmol). The mixture was stirred

for 12 h at this temperature and then purified by flash chromatography using EtOAc and petroleum ether.

The homogeneous control reaction catalyzed by Me_2**L** or H_3**L** was performed according to the following procedure:

To a flame-dried Schlenk tube was added with Me_2**L** (0.004 mmol) or H_3**L** (0.004 mmol), pyrrole (0.3 mmol) and dry DCE (0.3 mL). The reaction mixture was stirred for 1 h and then cooled to -10 ^{o}C followed by dropwise addition of of dry DCE (0.5 mL) containing nitroalkenes (0.1 mmol). The mixture was stirred for 12 h at this temperature and then purified by flash chromatography using EtOAc and petroleum ether.

Oxidation of sulfides with 1-Mn

A 10 mL flame-dried Schlenk tube equipped with a stir bar and the activated **1-Mn** (2 mol%, catalyst based on MOF) was added followed by addition of sulfides (0.1 mmol). The mixture was added with DCE (0.5 mL) and stirred for 1 h, then cooled to -30 ^{o}C. Aqueous H_2O_2 (30%, 14 μL, 1.2 equiv.) in DCE (0.3 mL) was added dropwise to the suspension and stirred for 4 h at this temperature. Purification by column chromatography on silica gel using EtOAc and petroleum ether as an eluent gave the desired sulfoxide. The ratios of sulfoxide/sulfone were determined by ^{1}H NMR of the crude product. The ee was determined by HPLC with a chiralcel OD-H column and the absolute configuration was assigned by comparing HPLC elution order with known literature data.

The control reaction catalyzed by Me_2**L-Mn** or H_3**L-Mn** was performed according to the following procedure:

A 10 mL flame-dried Schlenk tube equipped with a stir bar and Me_2**L-Mn** or H_3**L-Mn** (6 mol% catalyst based on Mn) was added followed by addition of sulfides (0.1 mmol). The mixture was added with DCE (0.5 mL) and stirred for 1 h, then cooled to -30 ^{o}C. Aqueous H_2O_2 (30%, 14 μL, 1.2 equiv.) in DCE (0.3 mL) was added dropwise to the suspension and stirred for 4 h at this temperature. Purification by column chromatography on silica gel using EtOAc and petroleum ether as an eluent gave the desired sulfoxide. The ratios of sulfoxide/sulfone were determined by ^{1}H NMR of the crude product. The ee was determined by HPLC with a chiralcel OD-H column and the absolute configuration was assigned by comparing HPLC elution order with known literature data.

The homogeneous control reaction catalyzed by Me_2**L** or H_3**L** was performed according to the following procedure:

To a flame-dried Schlenk tube was added with Me_2**L** (0.004 mmol) or H_3**L** (0.004 mmol) and sulfides (0.1 mmol). The mixture was added with DCE (0.5 mL) and stirred for 1 h, then cooled to -30 ^{o}C. Aqueous H_2O_2 (30%, 14 μL, 1.2 equiv.) in DCE (0.3 mL) was added dropwise to the suspension and stirred for 4 h at this temperature. Purification by column chromatography on silica gel using EtOAc and petroleum ether as an eluent gave the desired sulfoxide. The ratios of sulfoxide/sulfone were determined by ^{1}H NMR of the crude product. The ee was determined by HPLC with a chiralcel OD-H column and the absolute configuration was assigned by comparing HPLC elution order with known literature data.

Hydrogenation of quinoxalines with Hanztsch esters[1]

To a 10 mL flame-dried Schlenk tube was added the activated MOFs (10 mol%, catalyst based on MOF), quinoxaline (0.1 mmol) and anhydrous $CHCl_3$ (0.5 mL). The reaction mixture was stirred for 1 h and then Hantzsch Ester (60.72 mg, 2.4 equiv) was added in one portion. The resulting mixture was stirred at 60 ^{o}C for 24 h and then purified by flash chromatography using EtOAc and petroleum ether to afford the desired products. Yield was the isolated yield.

The homogeneous control reactions catalyzed by Me_2**L** or H_3**L** were performed according to the following procedure:

To a 10 mL flame-dried Schlenk tube was added the activated Me_2**L** or H_3**L** (0.02 mmol), quinoxaline (0.1 mmol) and anhydrous $CHCl_3$ (0.5 mL). The reaction mixture was stirred for 1 h and then Hantzsch Ester (60.72 mg, 2.4 equiv) was added in one portion. The resulting mixture was stirred for 24 h and then purified by flash chromatography using EtOAc and petroleum ether to afford the desired products. Yield was the isolated yield.

Recycling experiment

The recycling experiments were done in the similar procedure as above mentioned using the related substrates.

For MOFs catalyzed reactions: The catalysts were separated by centrifugation, and the supernatant was collected to give yields/conversions and ee values. The recovered MOFs were washed with fresh methanol and acetone for three times, reactivated at 100 °C for 1 h and then used for the next runs directly.

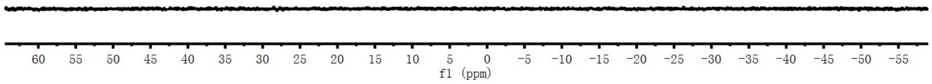

Supplementary Figure 1. ^{31}P NMR spectra of the supernatant of the reaction catalyzed by 1-Cr

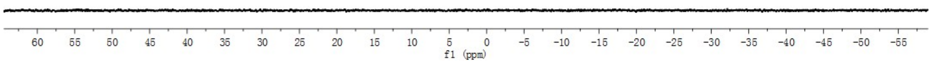

Supplementary Figure 2. ^{31}P NMR spectra of the supernatant of the reaction catalyzed by 1-Cr

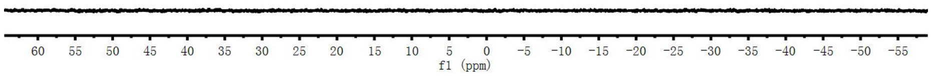

Supplementary Figure 3. ^{31}P NMR spectra of the supernatant of the reaction catalyzed by 1-Mg

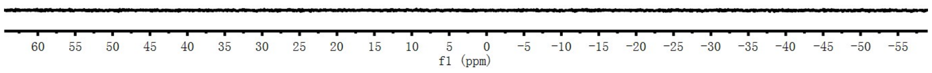

Supplementary Figure 4. ^{31}P NMR spectra of the supernatant of the reaction catalyzed by 1-Mn

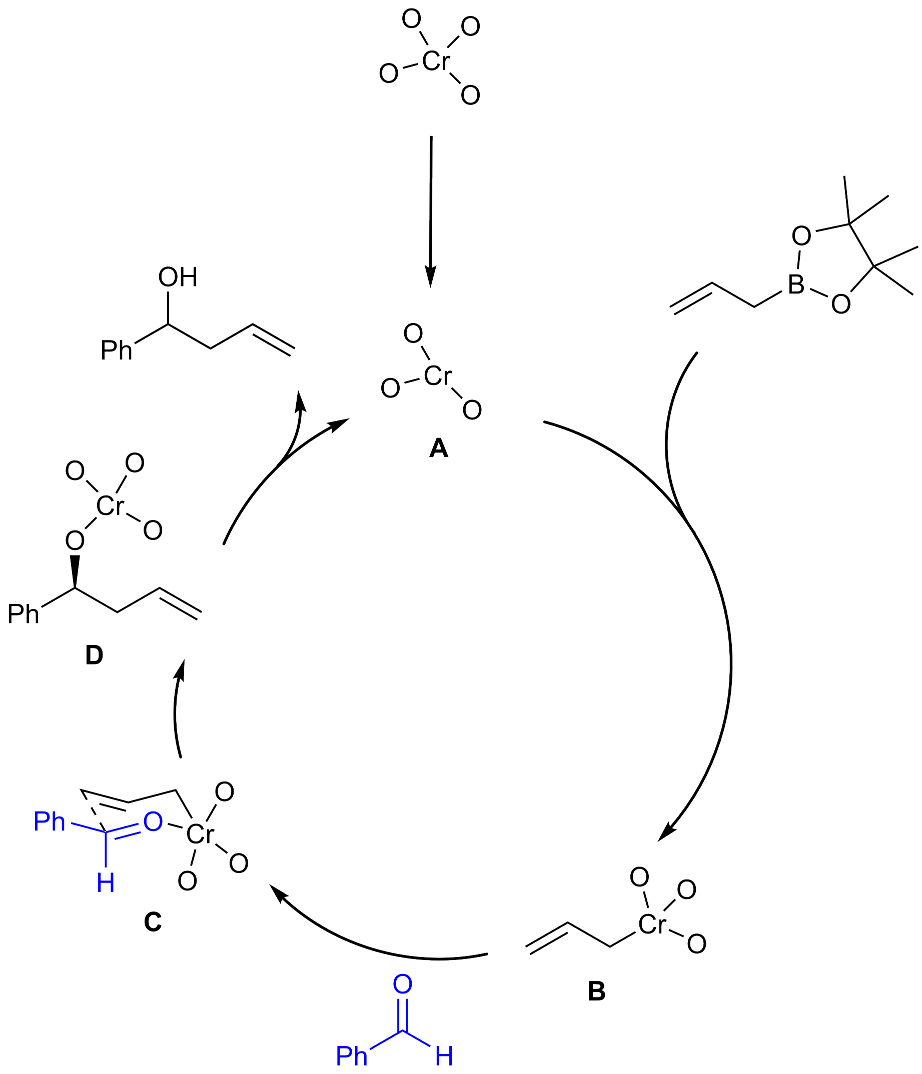

Supplementary Figure 5. Proposed a possible catalytic cycle for Allyboration of aldehydes (Cr was the terminal Cr in the linear trimeric Cr_3 unit) Based on the obtained results and reported literature[2-4], the catalytic cycle involves the following steps: Firstly, the one water or methanol molecule coordinated to the terminal Cr was removed to form coordinatively unsaturated metal center **A**. Secondly, exchange of ally active specie from boron to Cr to form intermediate **B**. Thirdly, reacting with aldehyde through six-membered transition state **C** in a γ-addition fashion to give **D**. Lastly, hydrolysis of **D** to get homoallylic alcohol.

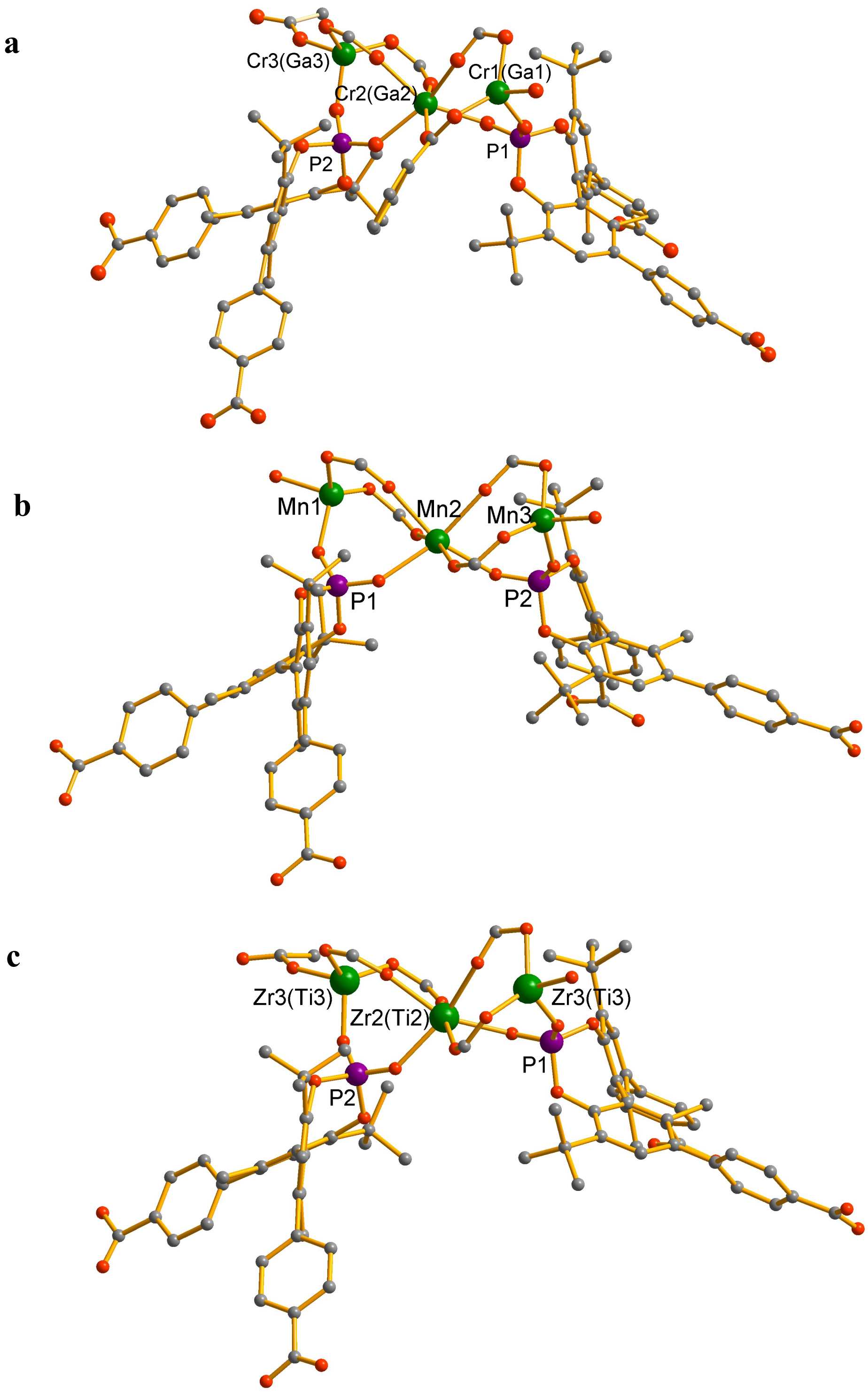

Supplementary Figure 6. The coordination environments of trimetallic clusters in MOFs 1-M: a, 1-Cr. **b, 1-**Mn. **c, 1-**Zr. (green, Metal; purple, P; red, O; gray, C).

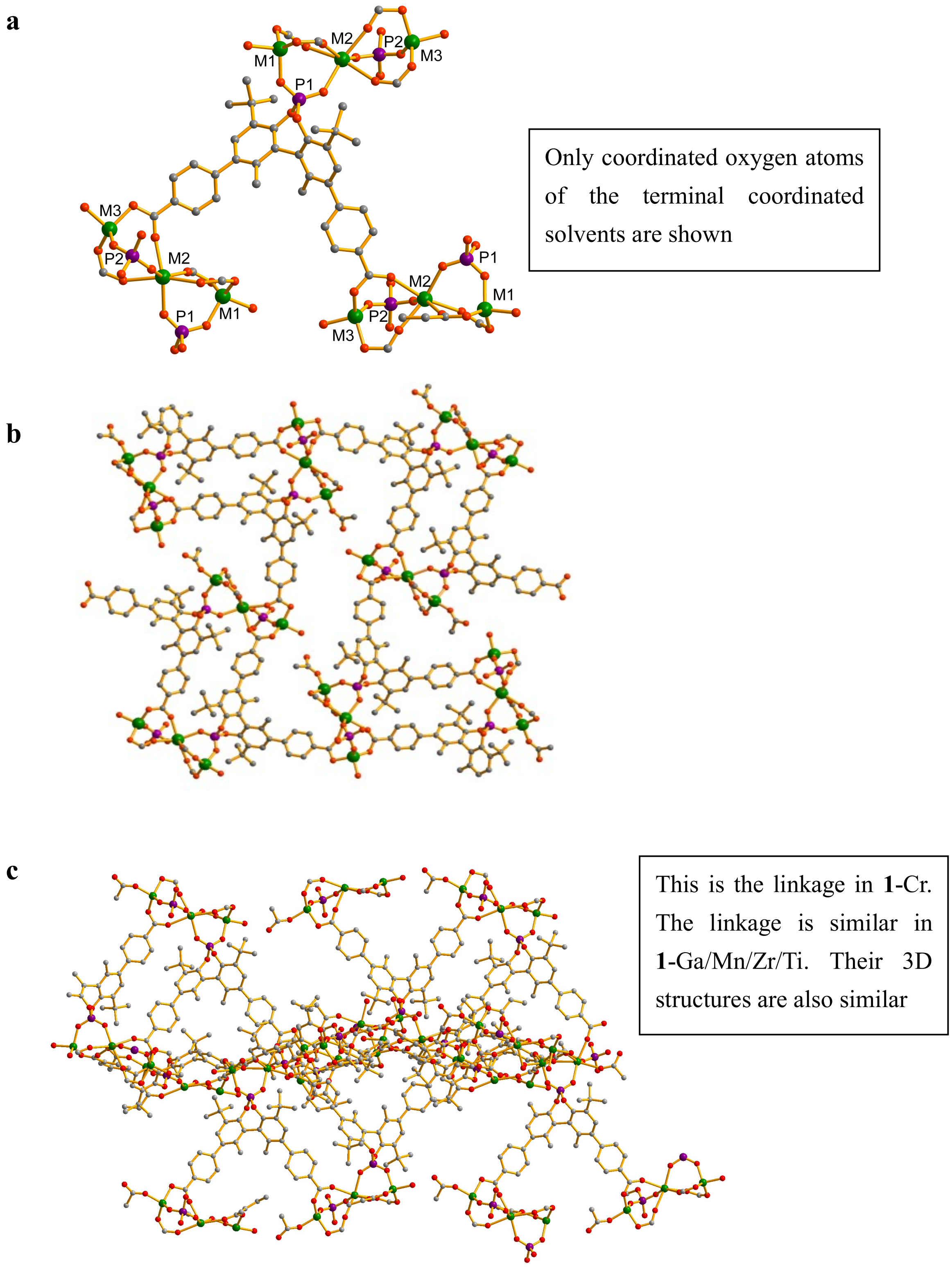

Supplementary Figure 7. a, The coordination mode of **L**. **b,** A view of 2D Sheet. **c,** Linking 2D sheet to 3D structure by **L** in **1**-Cr/Ga/Mn/Zr/Ti.

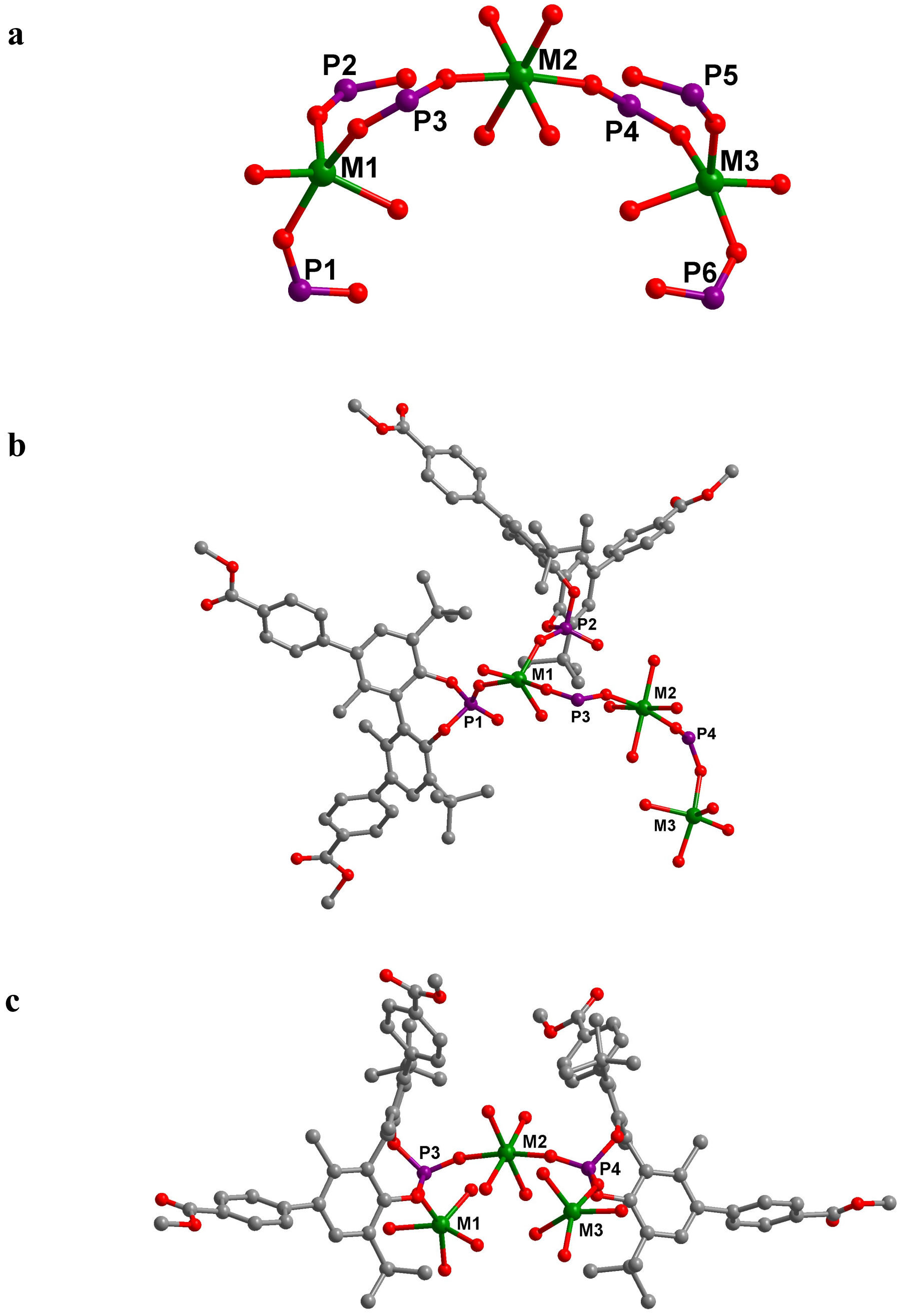

Supplementary Figure 8. a, The coordination mode of trimeric M_3 unit of homogeneous catalyst. **b,** The coordination mode of terminal M. **c,** The coordination mode of central M.

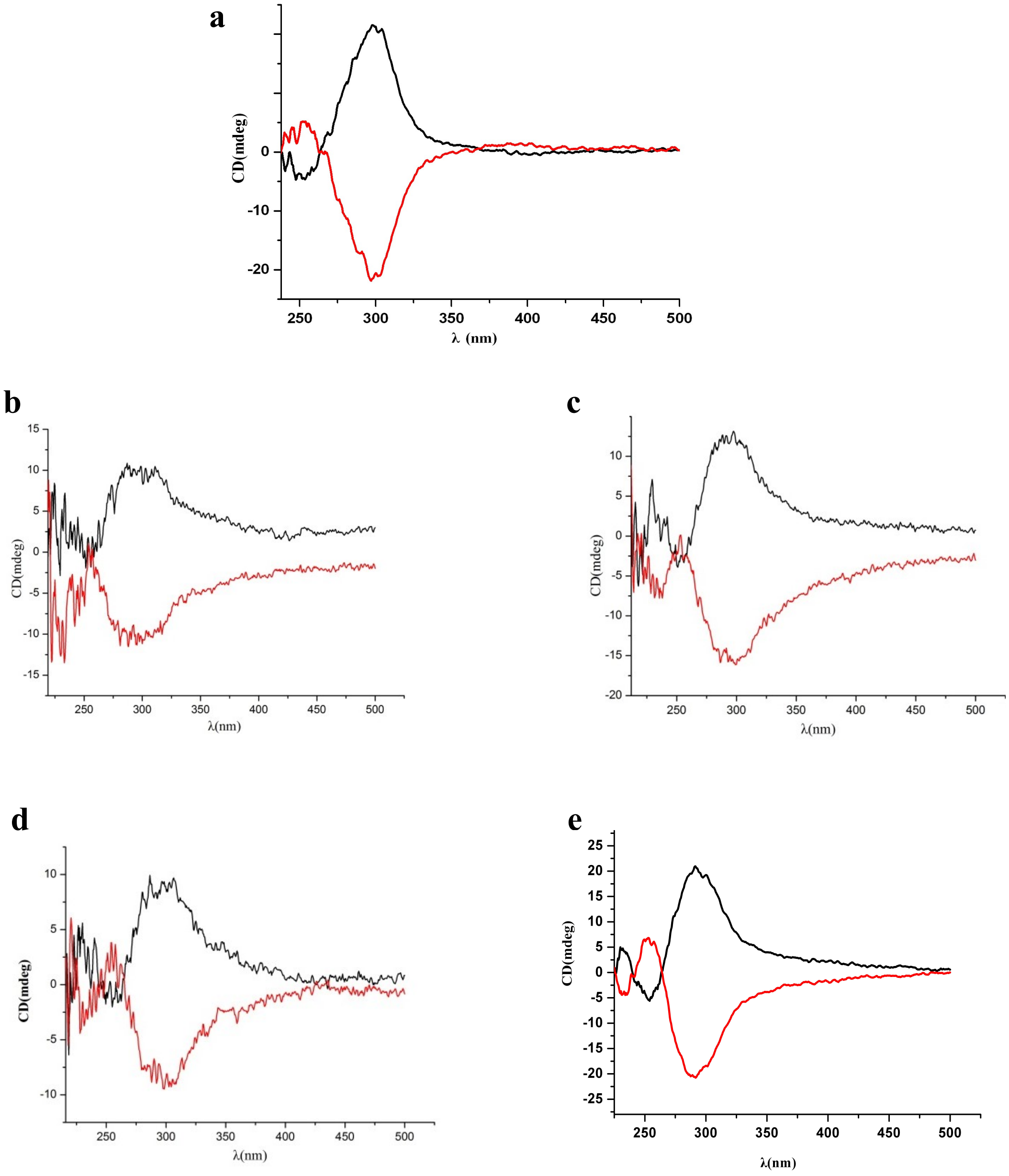

Supplementary Figure 9. Solid-state CD spectra: a, Ligand H_3**L**. **b**, **1**-Cr. **c**, **1**-Mg. **d**, **1**-Mn. **e**, **1**-Zr.

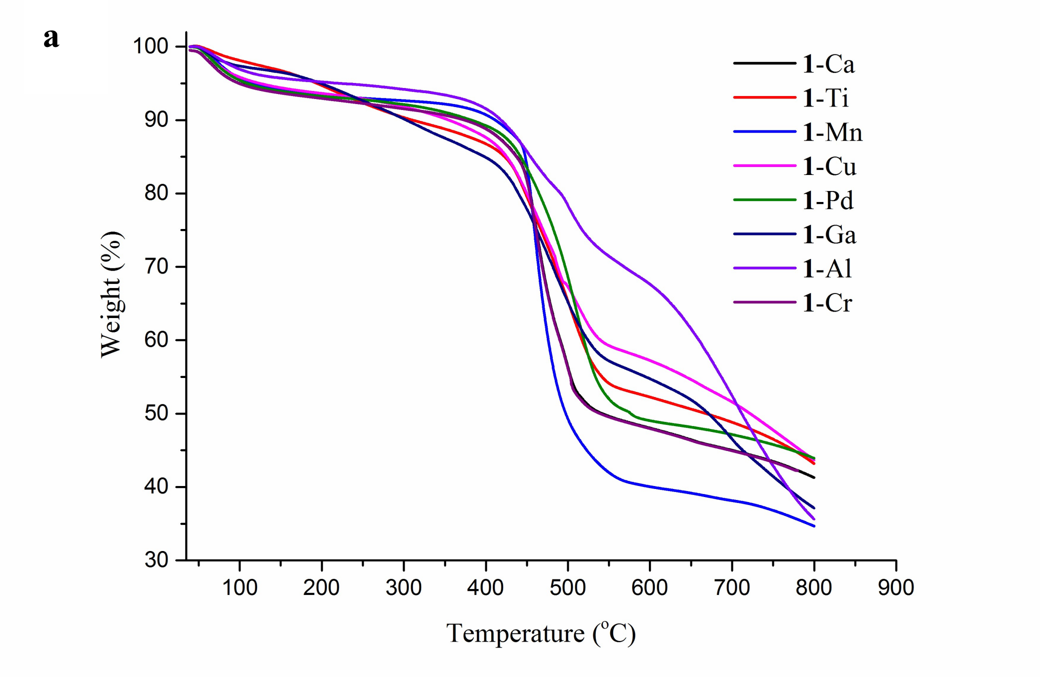

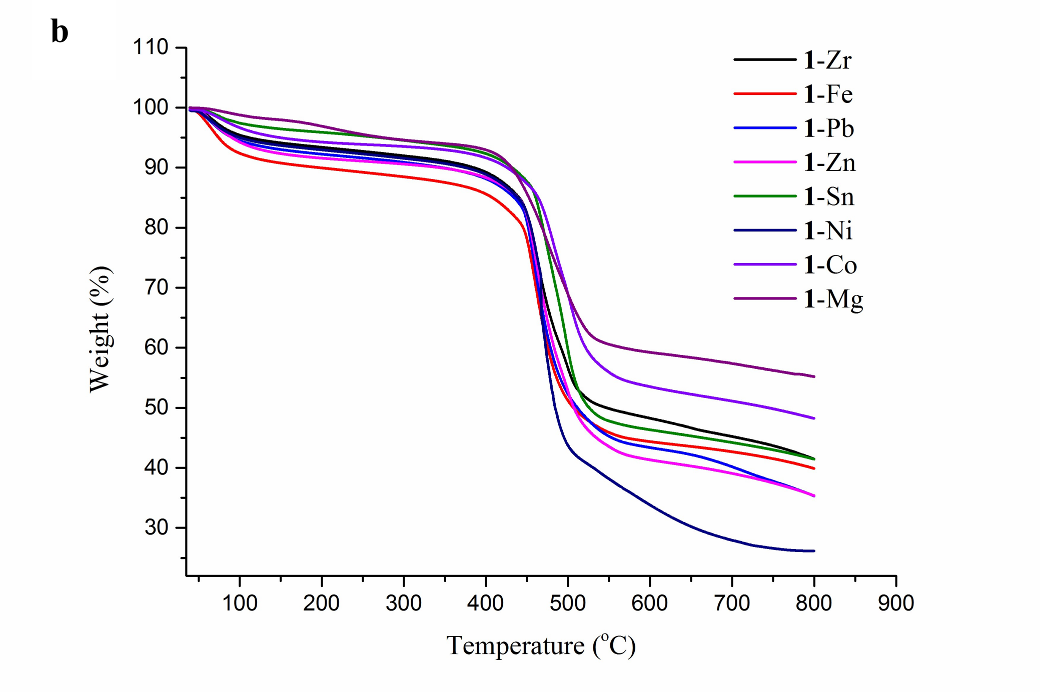

Supplementary Figure 10. TGA curves of 1-M: **a,** M=Ca, Ti, Mn, Cu, Pd, Ga, Al and Cr. **c,** M=Zr, Fe, Pb, Zn,Sn, Ni, Co and Mg.

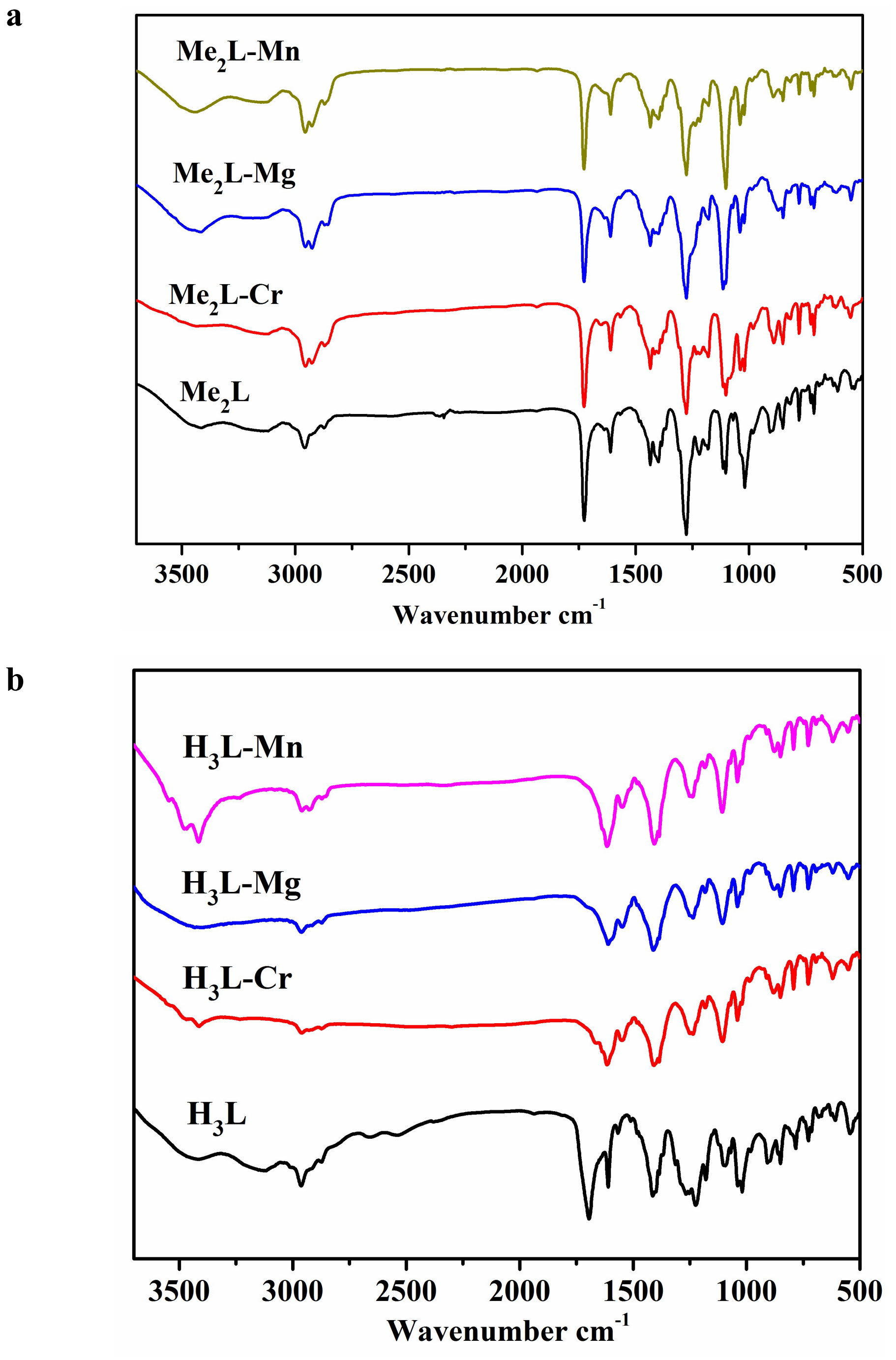

Supplementary Figure 11. FT-IR spectra of metal phosphates: a, Me_2L-M. **b,** H_3**L**-M.

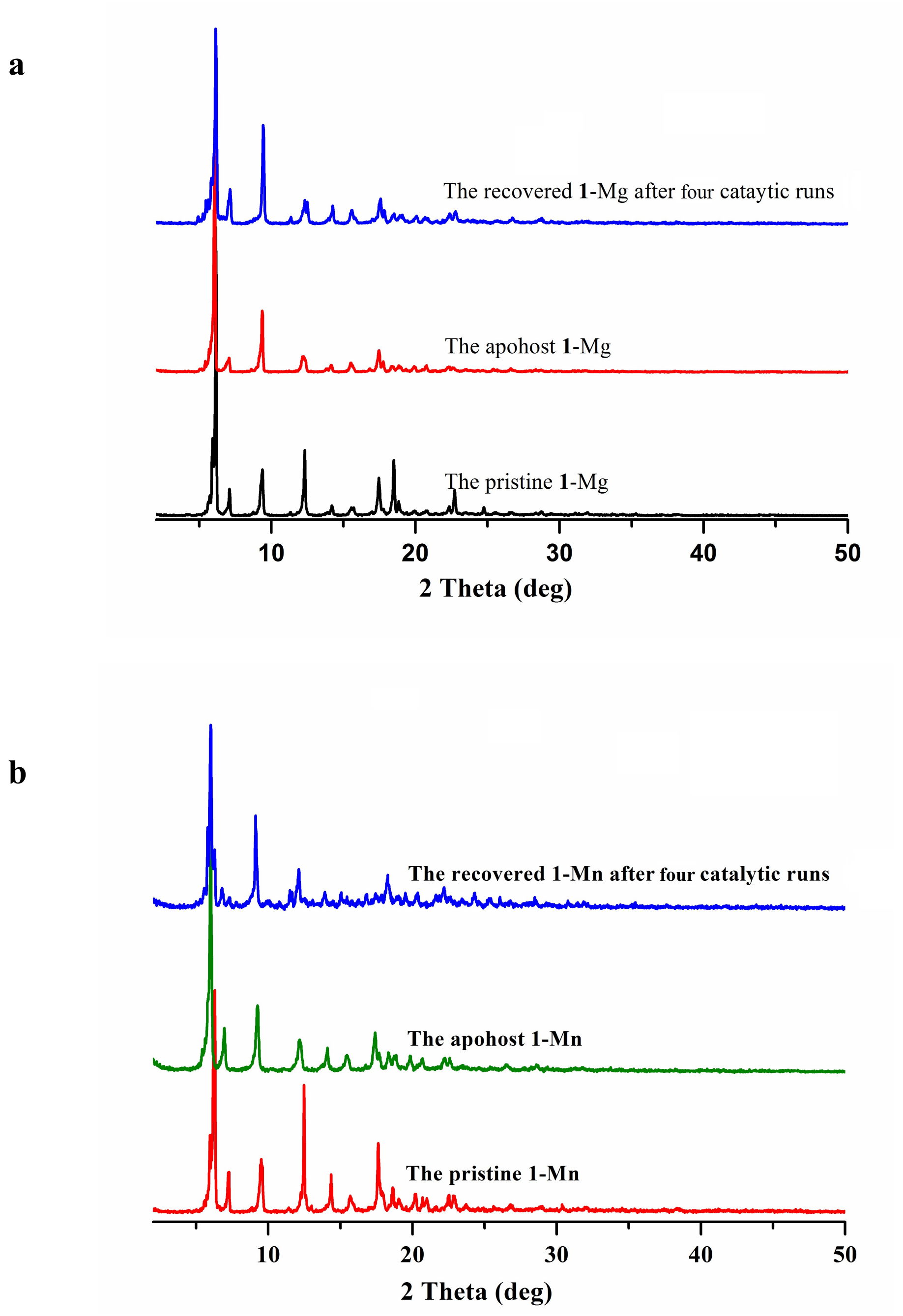

Supplementary Figure 12. PXRD of the apohost and recovered 1-M after four catalytic runs: a, 1-Mg. **b, 1**-Mn.

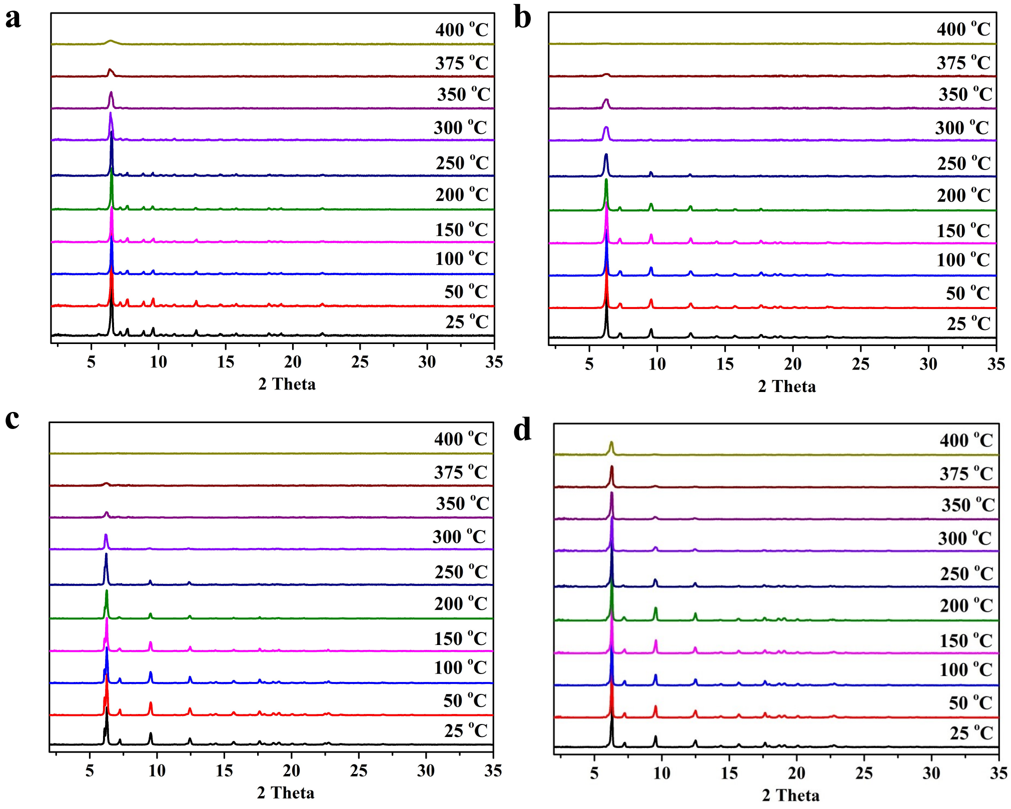

Supplementary Figure 13. Variable temperature PXRD of 1-M: a, **1**-Cr. **b**, **1**-Mn. **c**, **1**-Mg. **d**, **1**-Zr

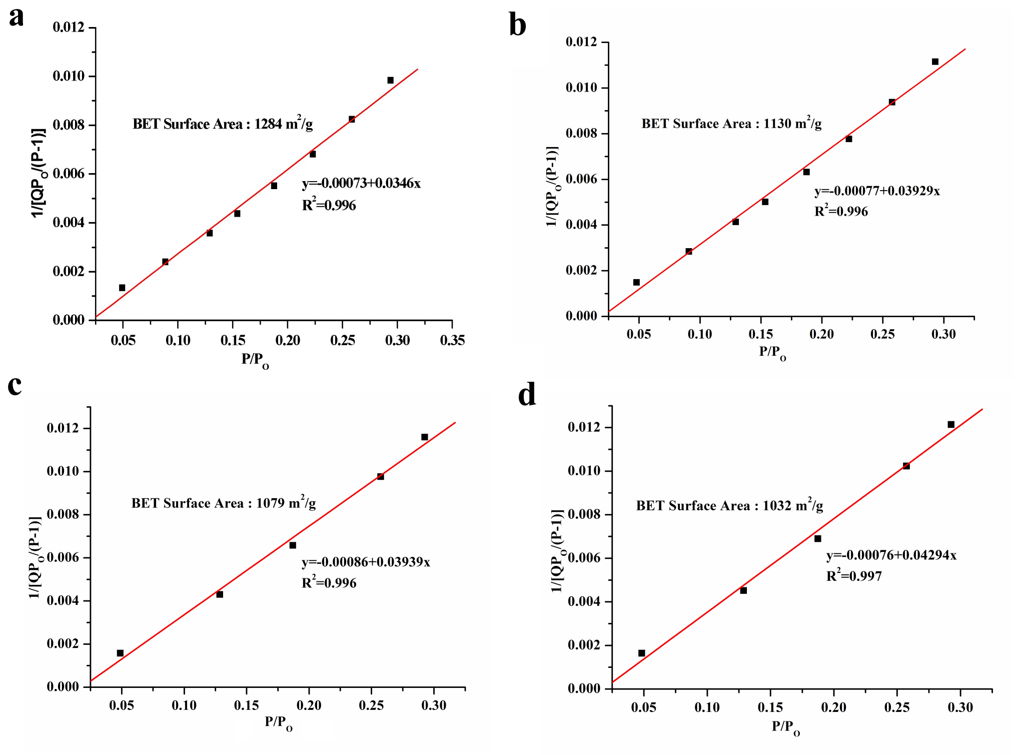

Supplementary Figure 14. BET plots of 1-Cr: a, The pristine sample. **b,** @boiling water after 7 days. **c,** @pH=4 solution after 7 days. **d,** @pH=9 solution after 7 days.

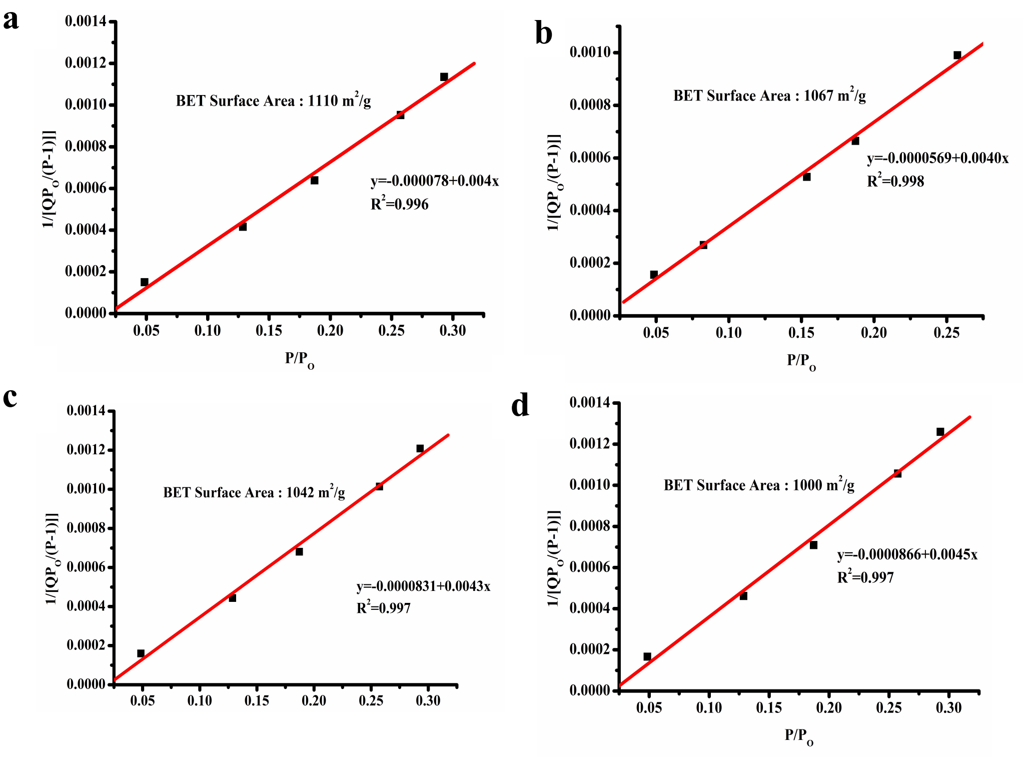

Supplementary Figure 15. BET plots of 1-Mg: a, The pristine sample. **b,** @boiling water after 7 days. **c,** @pH=4 solution after 7 days. **d,** @pH=9 solution after 7 days.

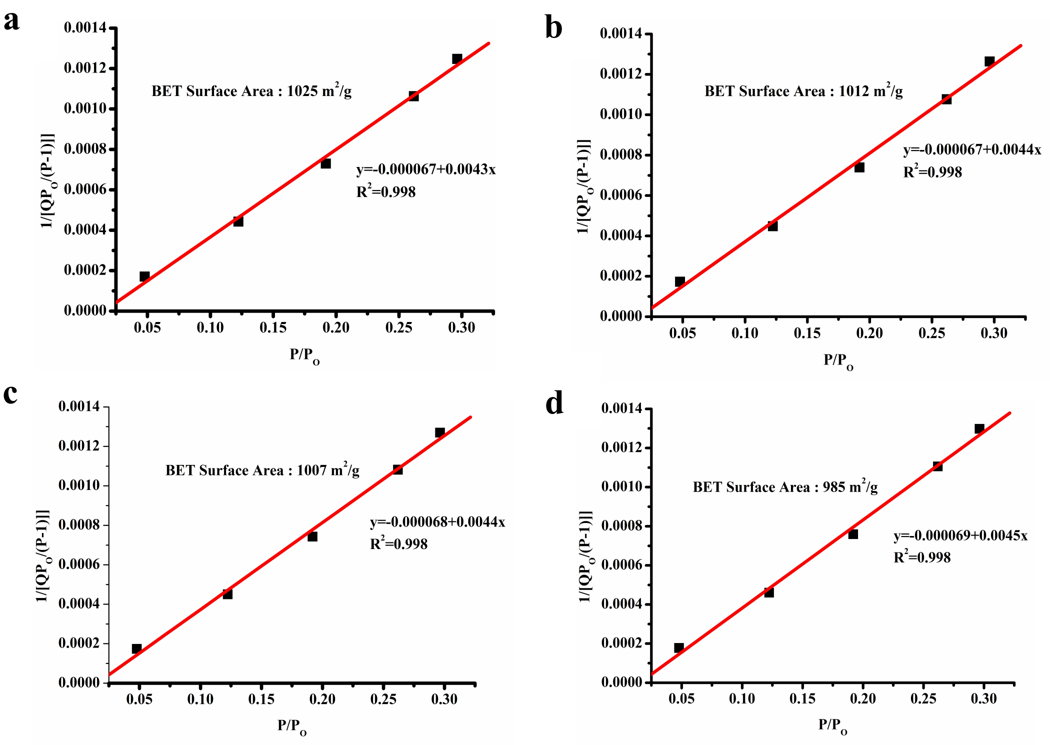

Supplementary Figure 16. BET plotsof 1-Mn: **a,** The pristine sample. **b,** @boiling water after 7 days. **c,** @pH=4 solution after 7 days. **d,** @pH=9 solution after 7 days.

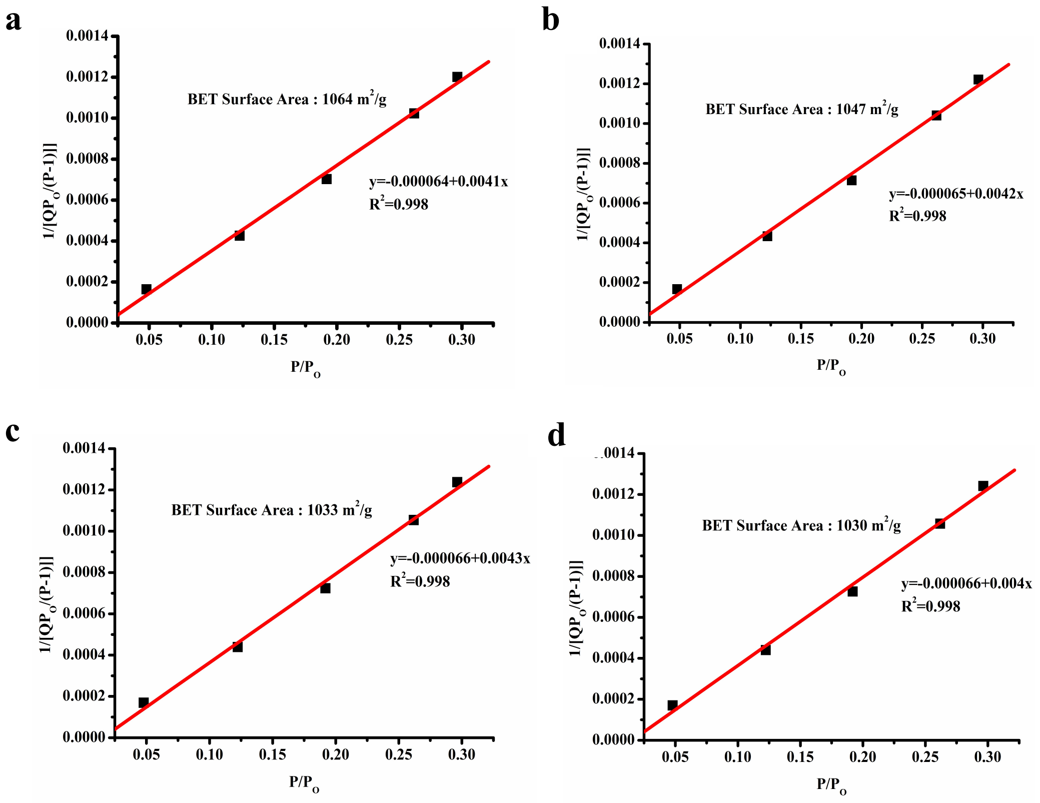

Supplementary Figure 17. BET plots of 1-Zr: a, The pristine sample. **b,** @boiling water after 7 days. **c,** @pH=4 solution after 7 days. **d,** @pH=9 solution after 7 days.

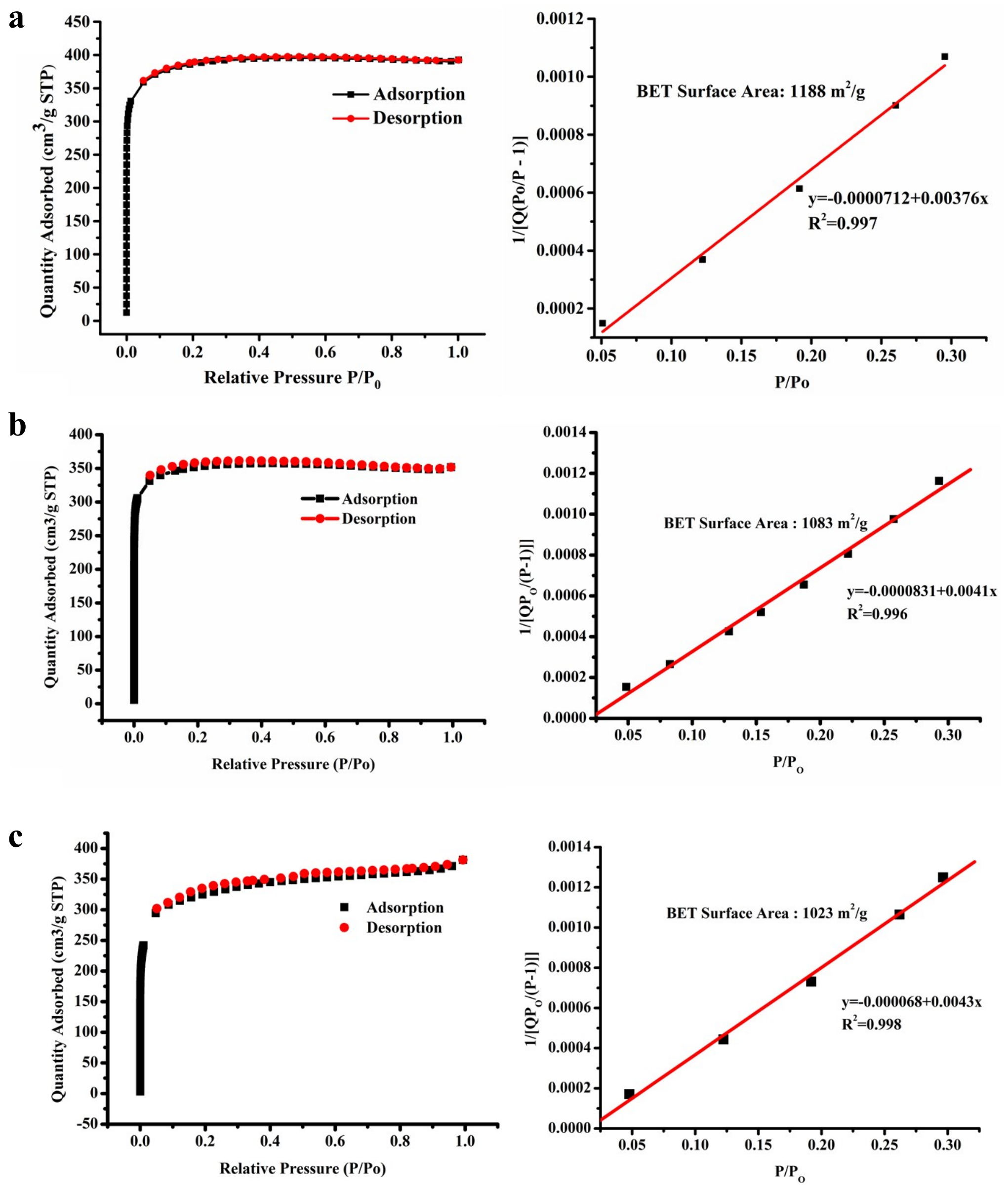

Supplementary Figure 18. N_2 adsorption isotherms (left) and BET plots (right): **a,** Pristine **1**-Cr. Recovered **1**-M after four catalytic runs **b, 1**-Mg. **c, 1**-Mn.

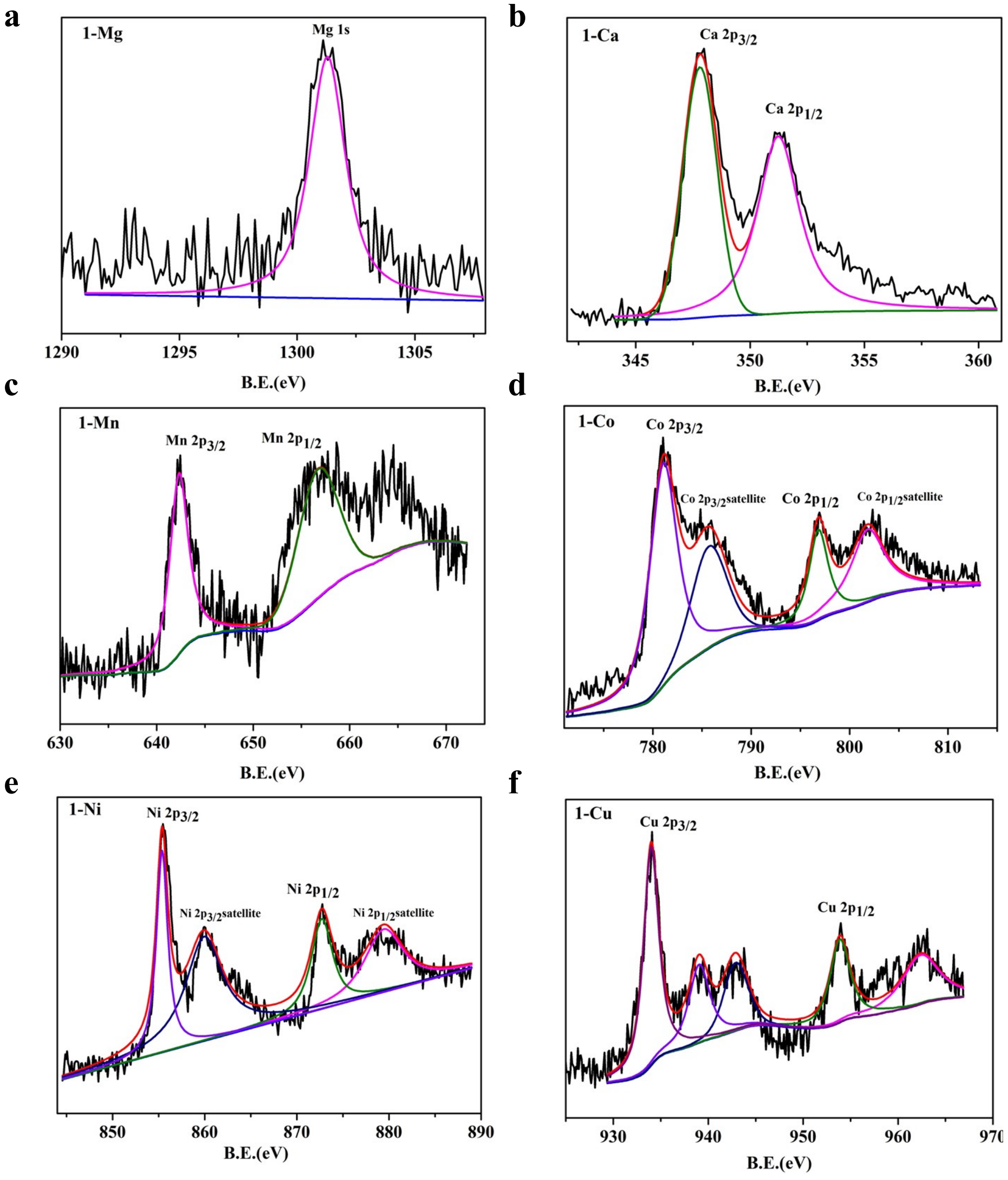

Supplementary Figure 19. XPS spectra of 1-M: **a, 1**-Mg. **b, 1**-Ca. **c, 1**-Mn. **d, 1**-Co. **e, 1**-Ni. **f, 1**-Cu.

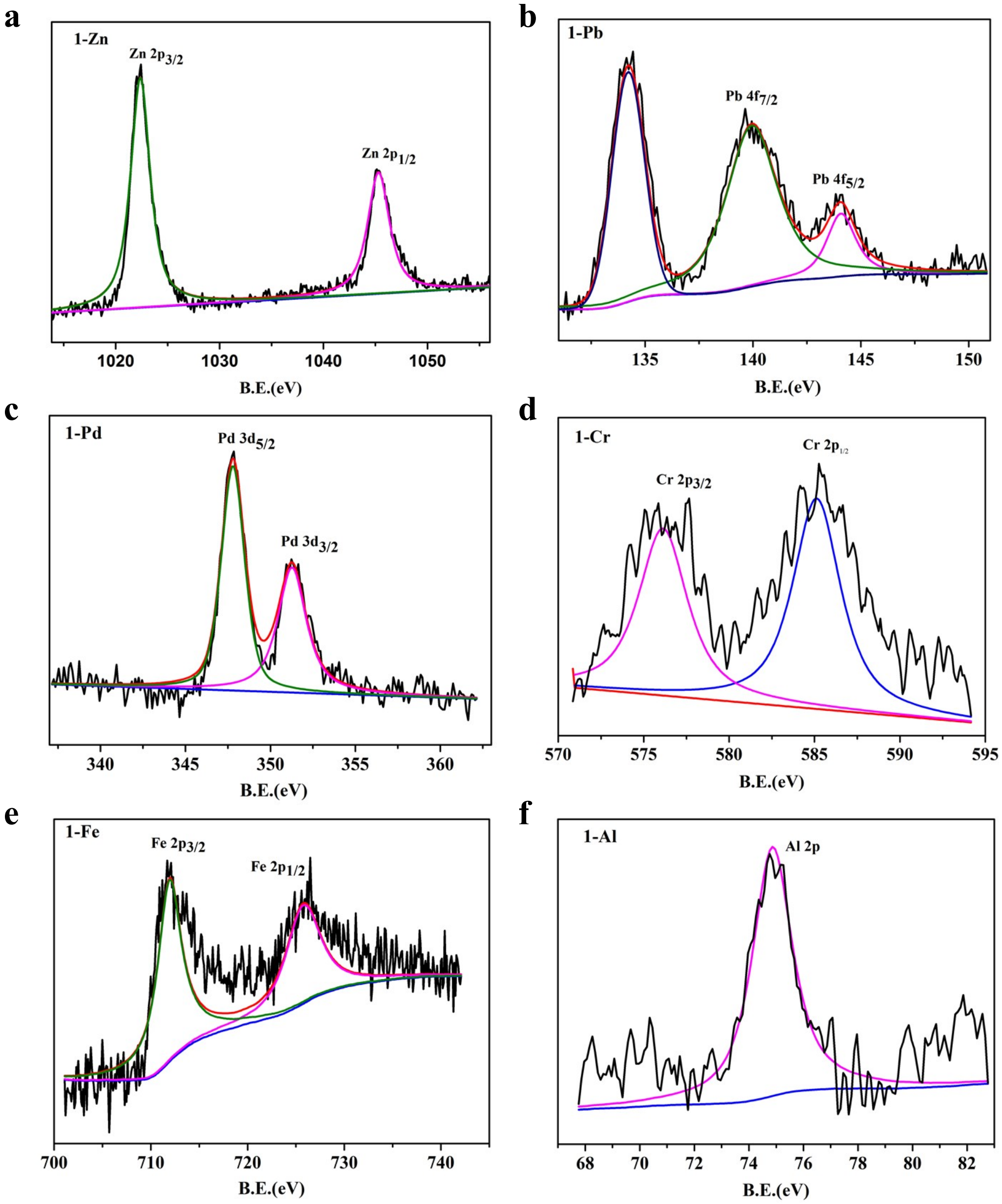

Supplementary Figure 20. XPS spectra of 1-M: a, 1-Zn. **b, 1**-Pb. **c, 1**-Pd. **d, 1**-Cr. **e, 1**-Fe. **f, 1**-Al.

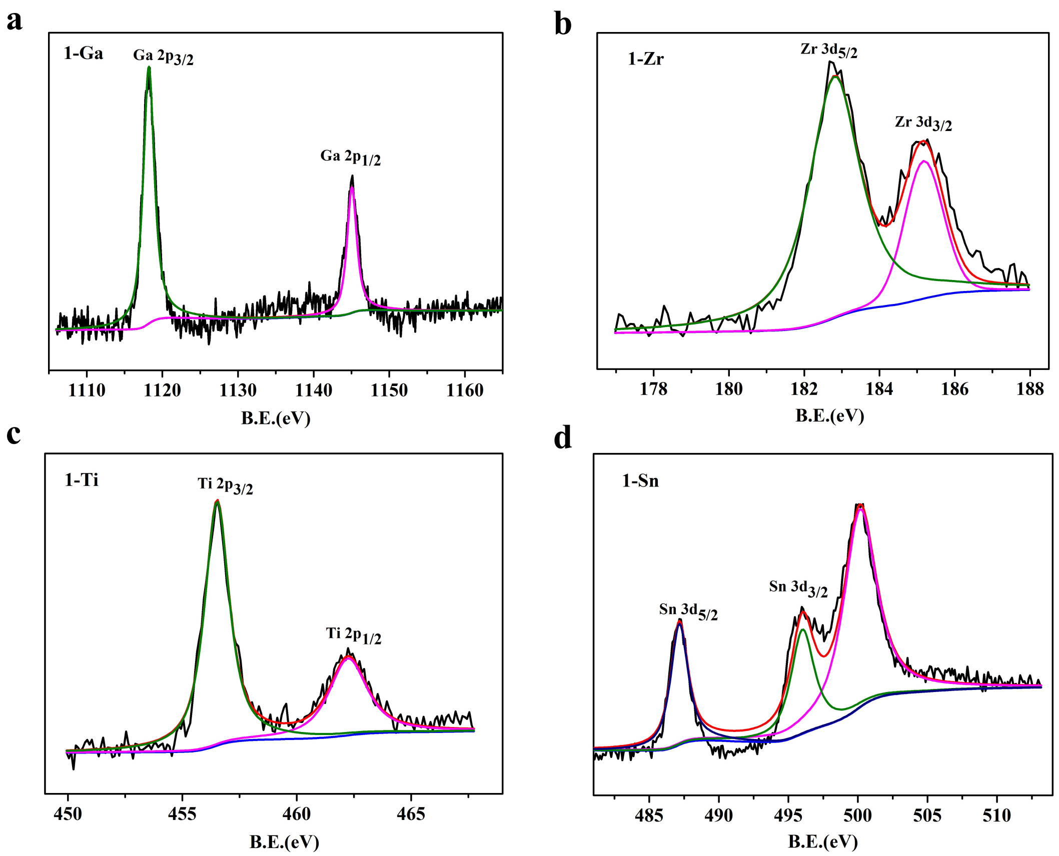

Supplementary Figure 21. XPS spectra of 1-M: a, 1-Ga. **b, 1**-Zr. **c, 1**-Ti. **d, 1**-Sn.

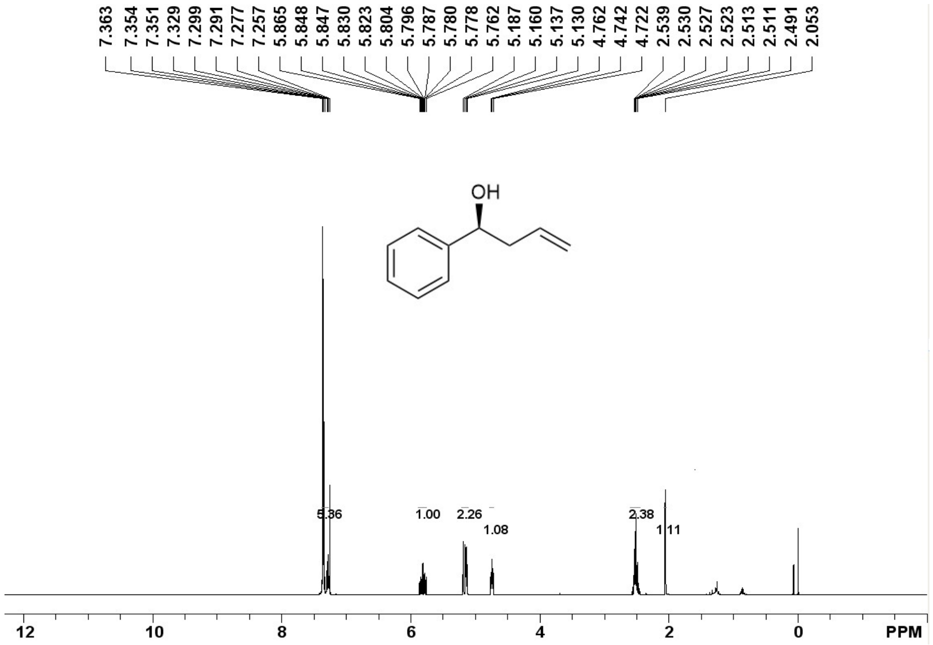

Supplementary Figure 22. **^{1}H NMR spectra for (*S*)-1-phenyl-but-3-en-1-ol**

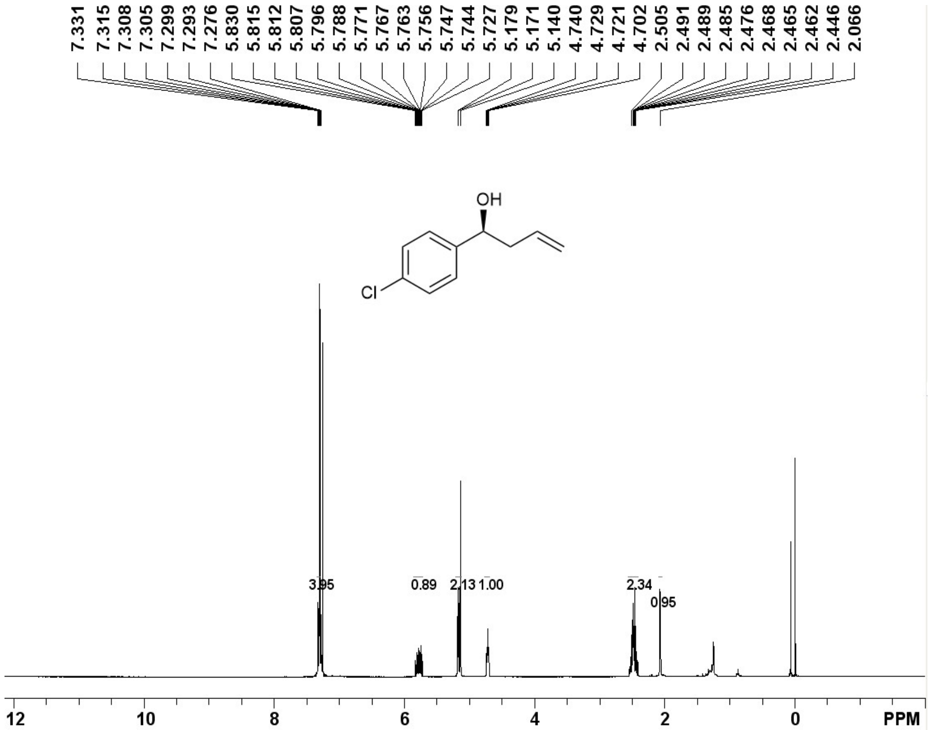

Supplementary Figure 23. **^{1}H NMR spectra for (*S*)-1-(4-chloro-phenyl)-but-3-en-1-ol**

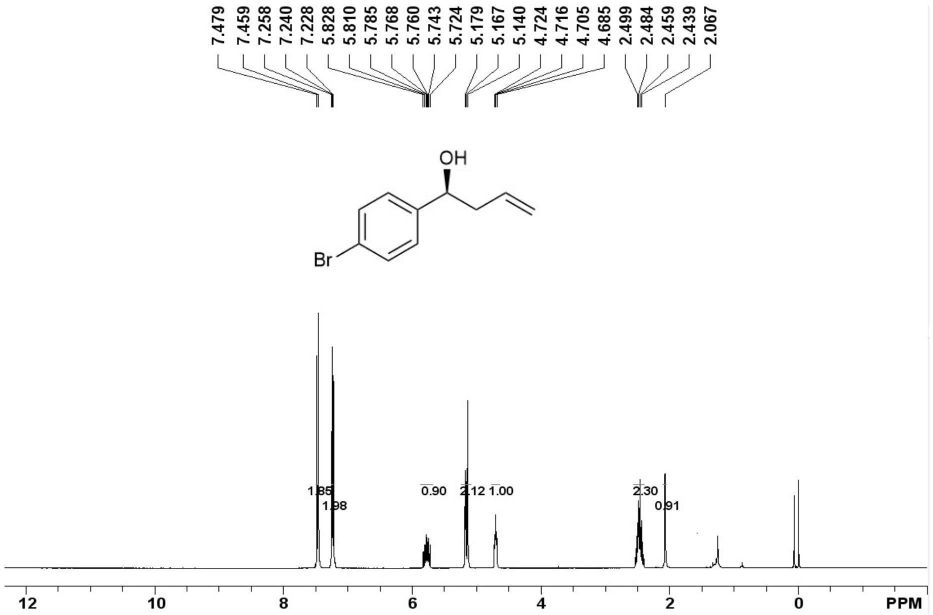

Supplementary Figure 24. ^{1}H NMR spectra for (*S*)-1-(4-bromo-phenyl)-but-3-en-1-ol

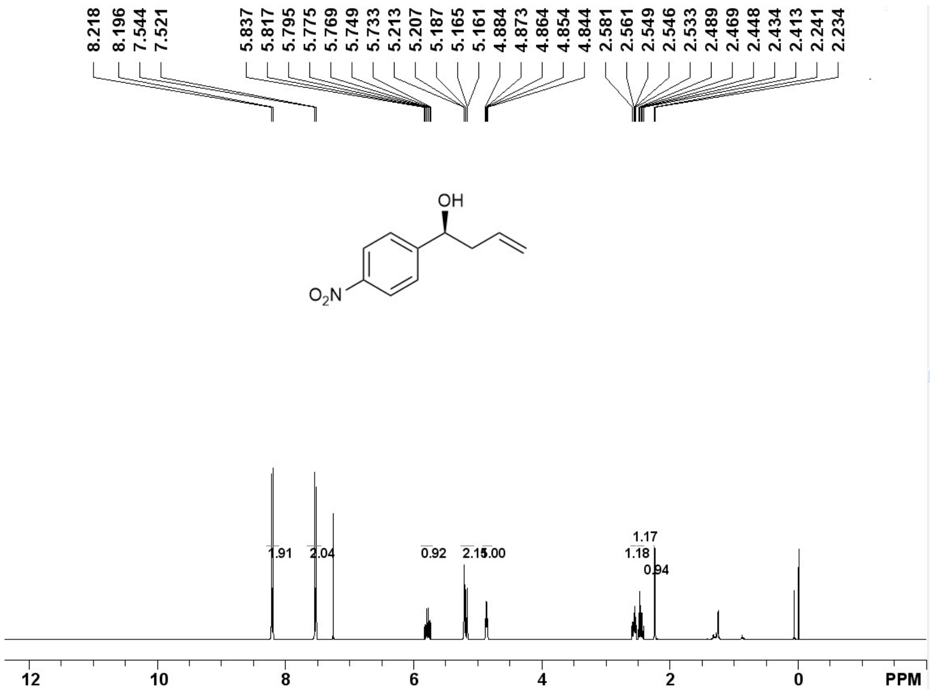

Supplementary Figure 25. ^{1}H NMR spectra for (*S*)-1-(4-nitro-phenyl)-but-3-en-1-ol

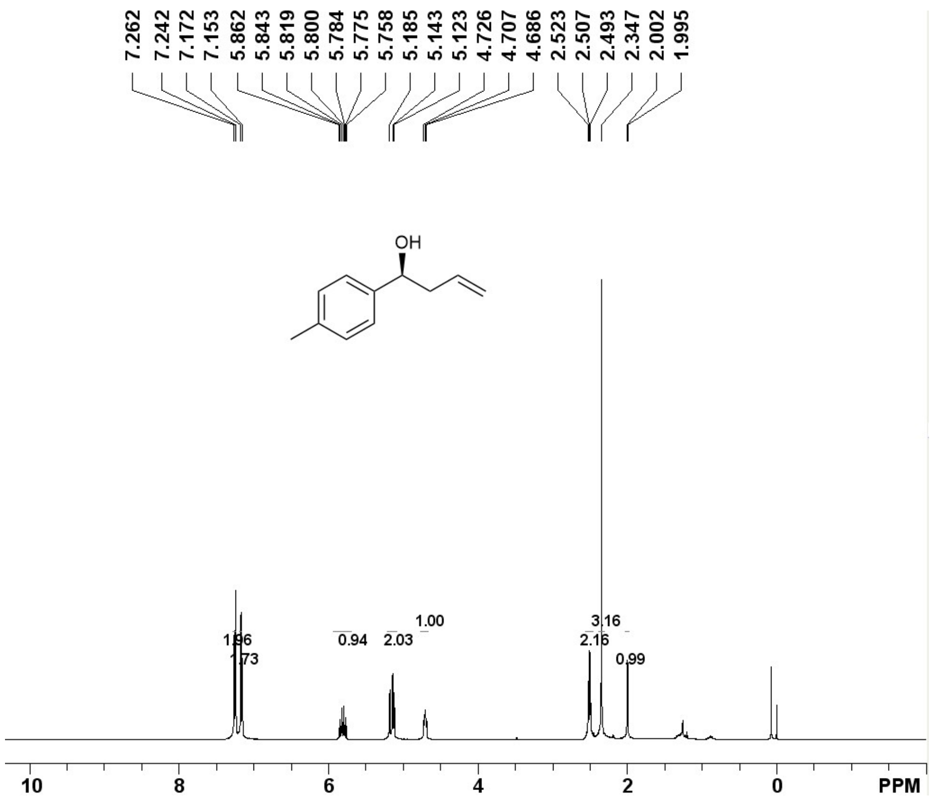

Supplementary Figure 26. ^{1}H NMR spectra for (*S*)-1-(4-Methyl-phenyl)-but-3-en-1-ol

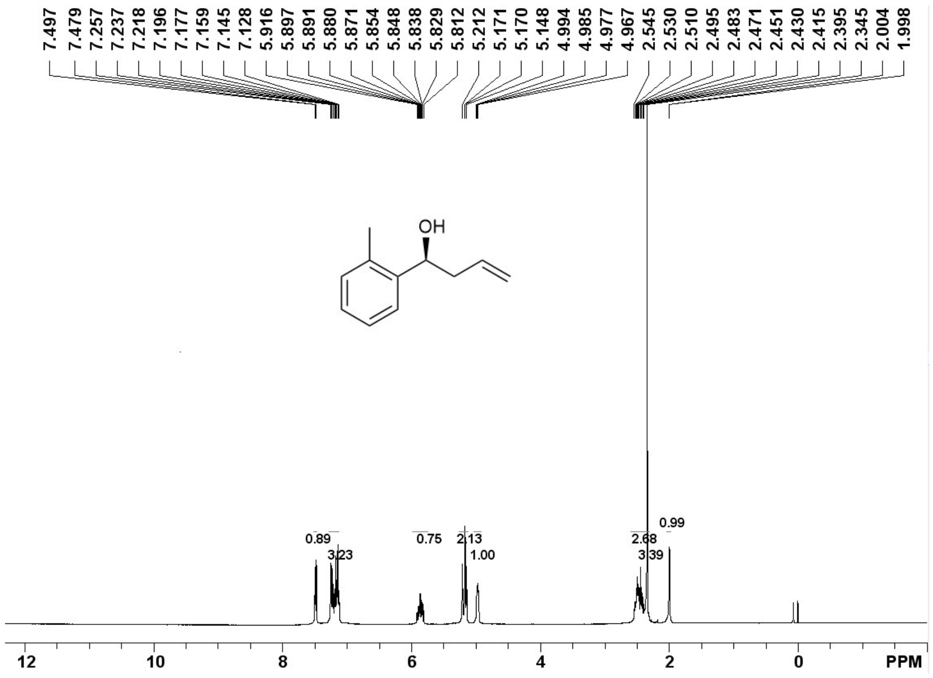

Supplementary Figure 27. ^{1}H NMR spectra for (*S*)-1-(*o*-methyl-phenyl)-but-3-en -1-ol

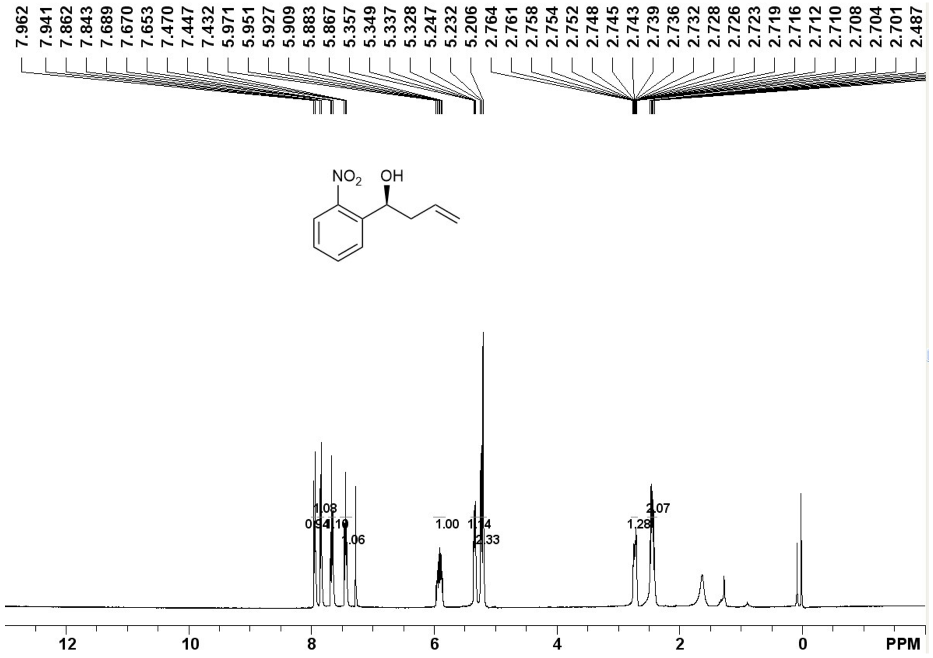

Supplementary Figure 28. ^{1}H NMR spectra for (*S*)-1-(2-Nitro-phenyl)-but-3-en-1-ol

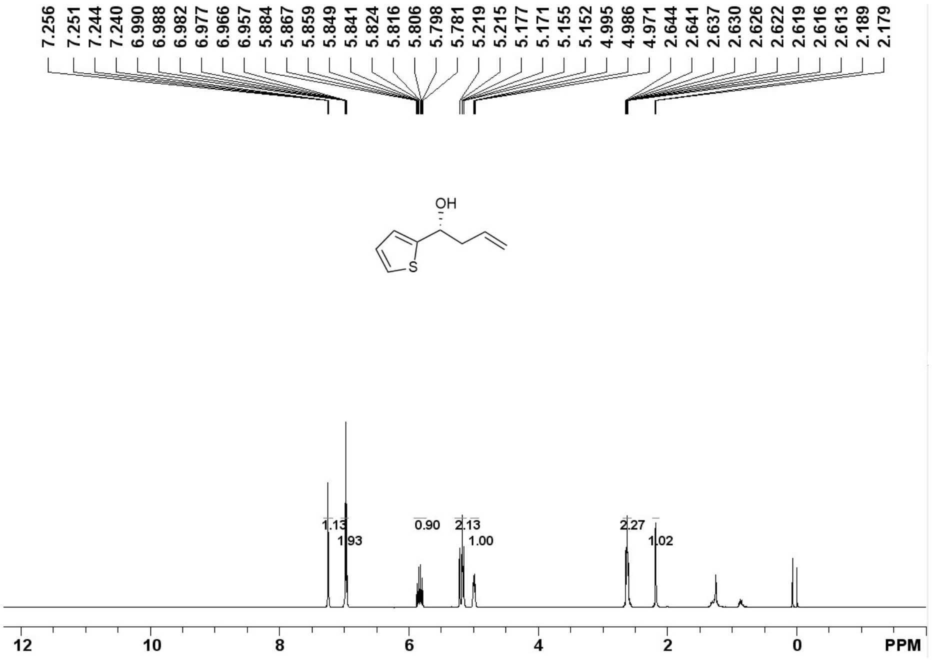

Supplementary Figure 29. ^{1}H NMR spectra for (*R*)-1-Thiophen-2-yl-but-3-en-1-ol

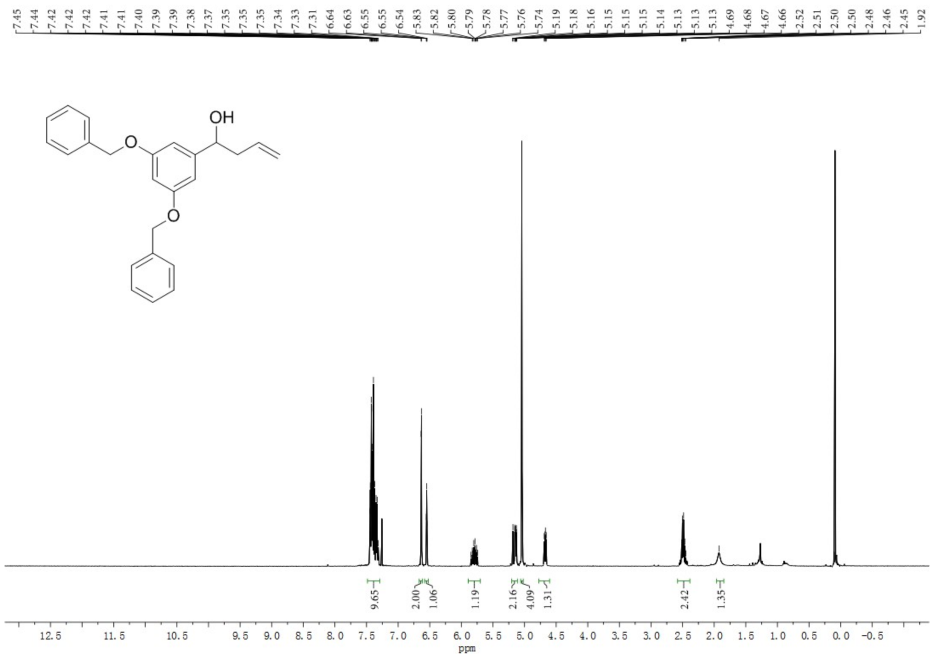

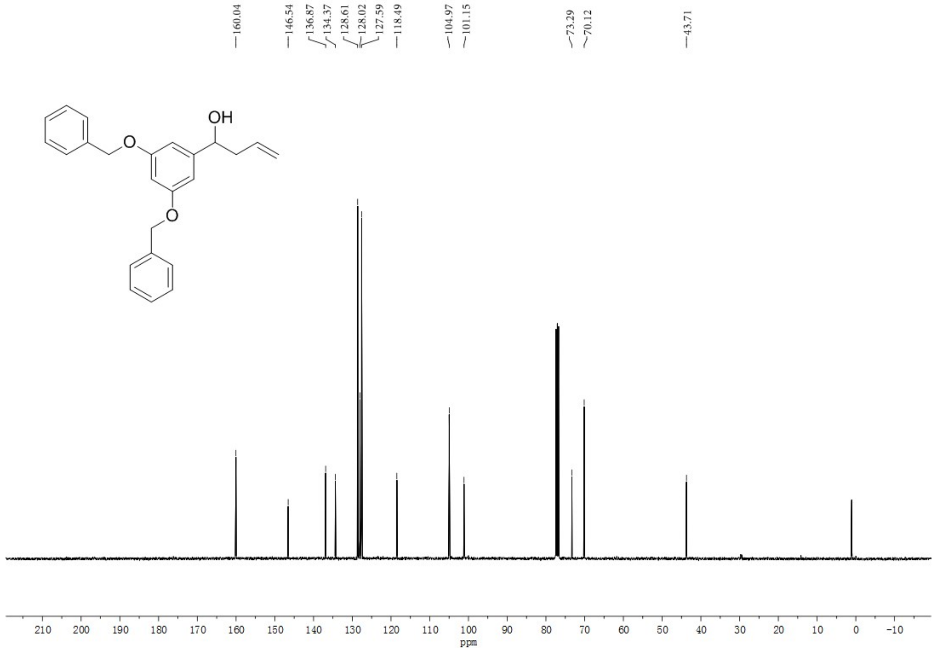

Supplementary Figure 30. ^{1}H and ^{13}C NMR spectra for 1-(3,5-bis(benzyloxy)ph enyl) but-3-en-1-ol

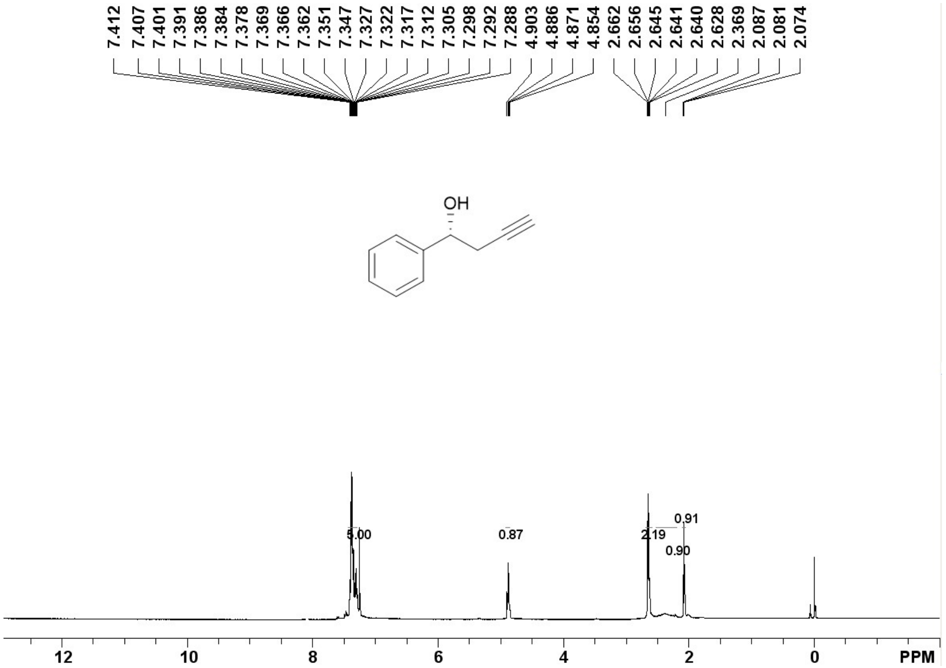

Supplementary Figure 31. ^{1}H NMR spectra for (*R*)-1-Phenyl-but-3-yn-1-ol

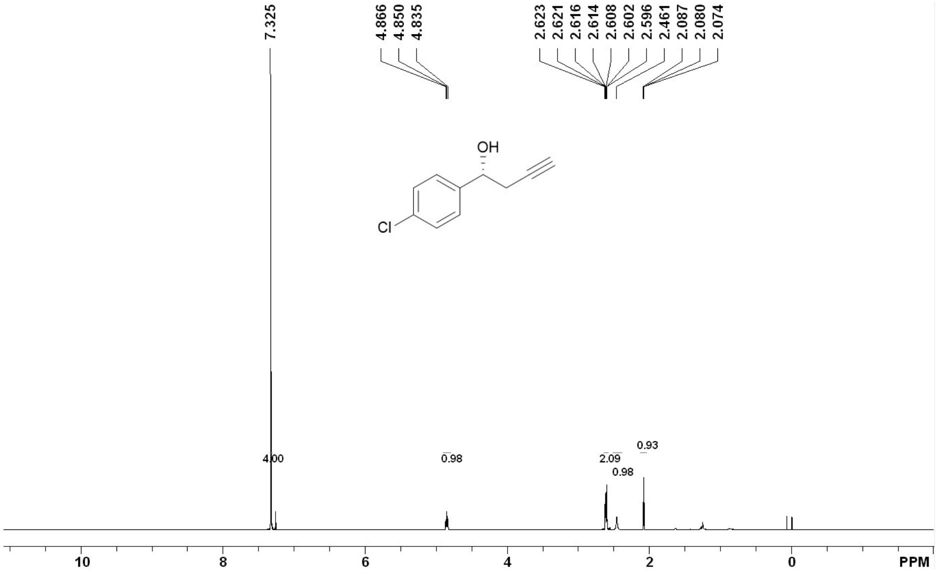

Supplementary Figure 32. ^{1}H NMR spectra for (*R*)-1-(4-Chloro-phenyl)-but-3-yn-1-ol

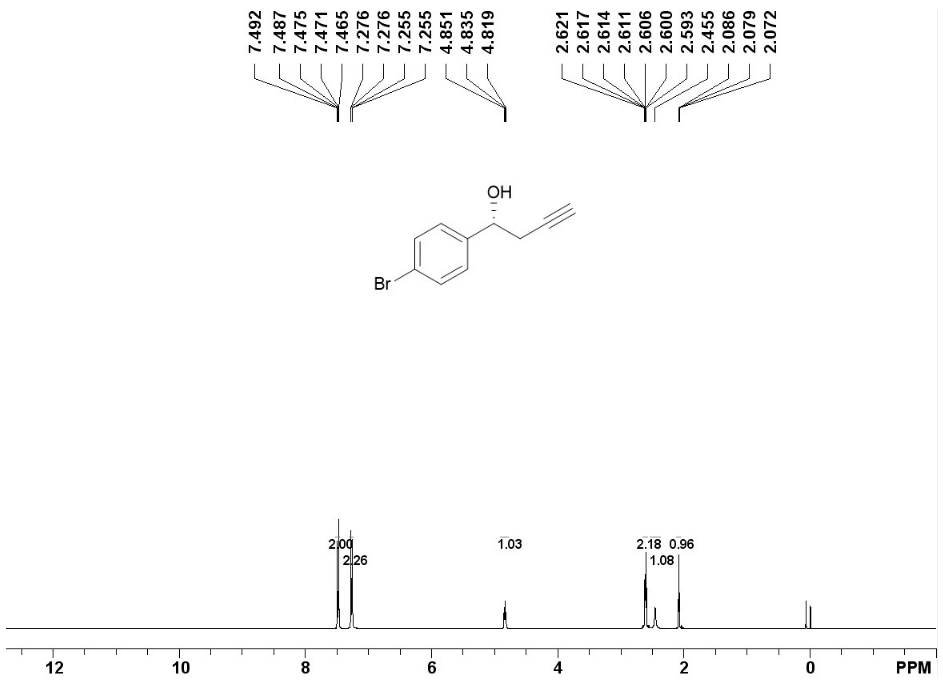

Supplementary Figure 33. ^{1}H NMR spectra for (R)-1-(4-Bromo-phenyl)-but-3-yn -1-ol

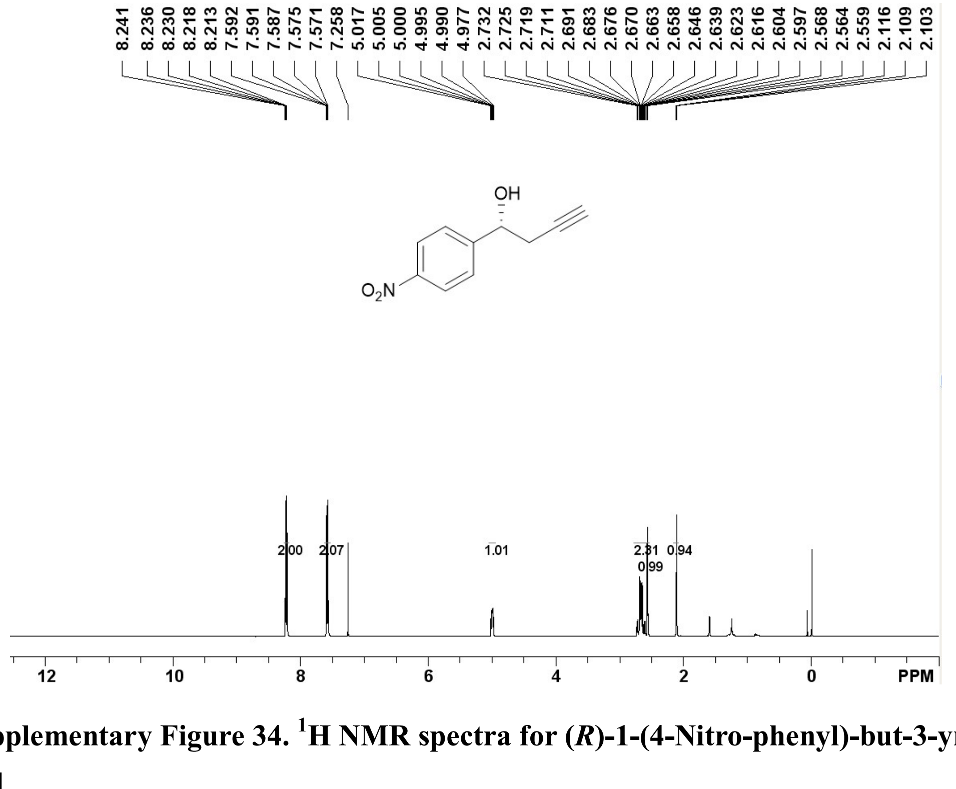

Supplementary Figure 34. ^{1}H NMR spectra for (*R*)-1-(4-Nitro-phenyl)-but-3-yn-1-ol

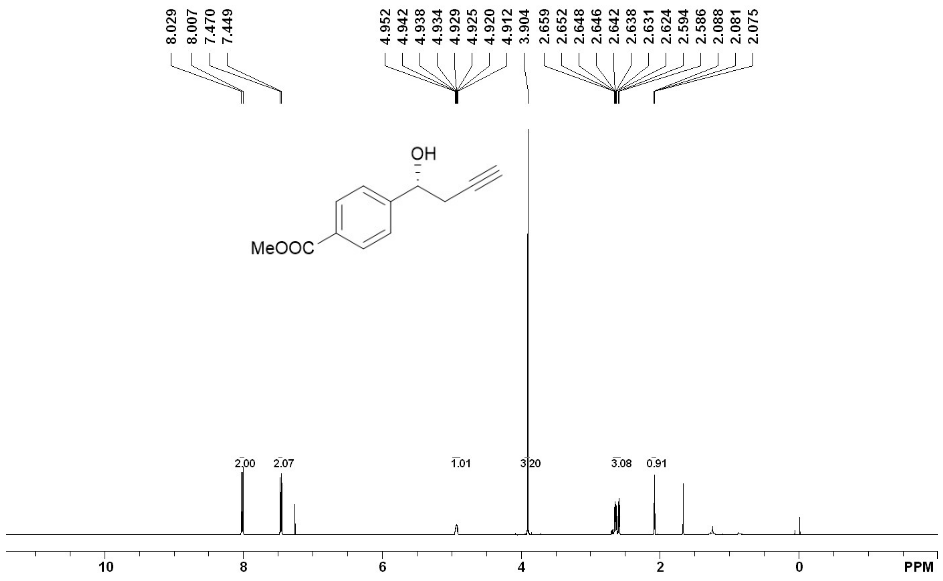

Supplementary Figure 35. ^{1}H NMR spectra for ((*R*)-Methyl 4-(1-hydroxybut-3-yn-1-yl)benzoate

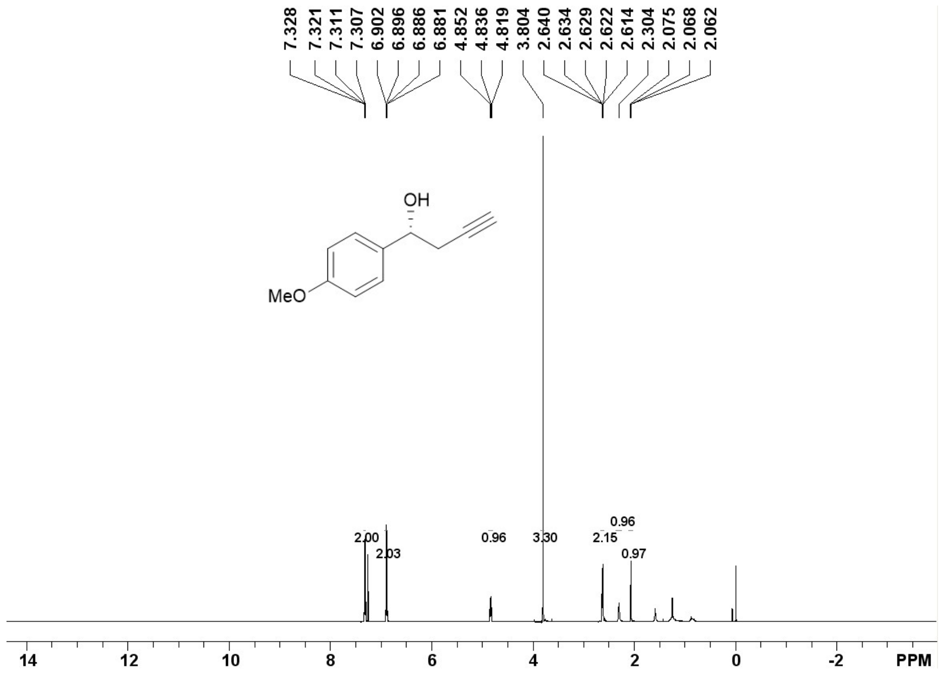

Supplementary Figure 36. ^{1}H NMR spectra for (*R*)-1-(4-Methoxy-phenyl)-but-3-yn-1-ol

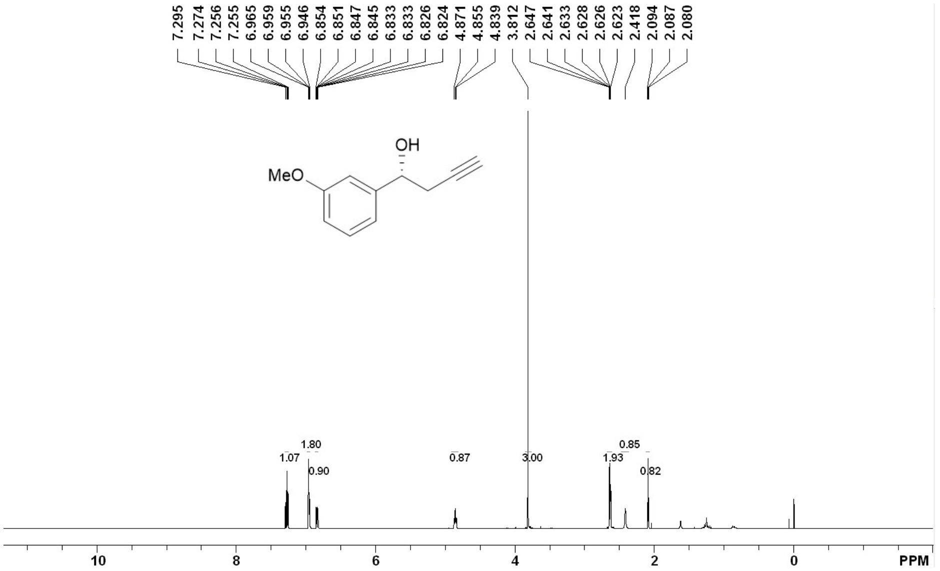

Supplementary Figure 37. ^{1}H NMR spectra for (*R*)-1-(3-Methoxy-phenyl)-but-3-yn-1-ol

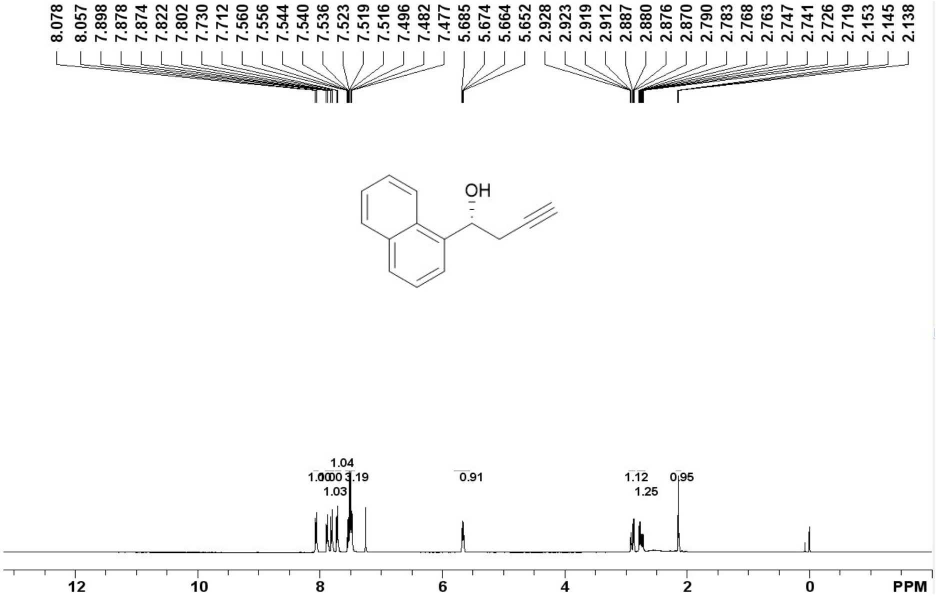

Supplementary Figure 38. ^{1}H NMR spectra for (*R*)-1-(Naphthalen-1-yl)but-3-yn-1-ol

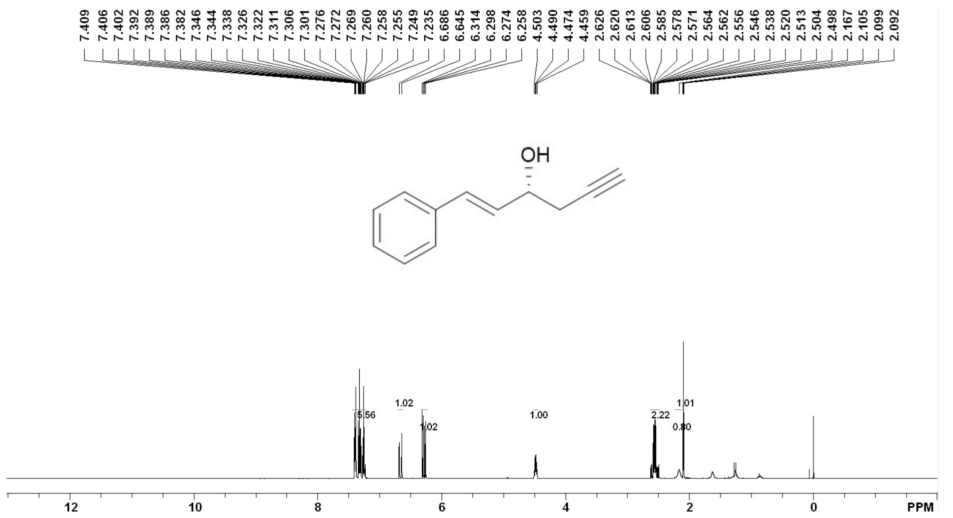

Supplementary Figure 39. ^{1}**H NMR spectra for (*R*, *E*)-1-phenylhex-1-en-5-yn-3-ol**

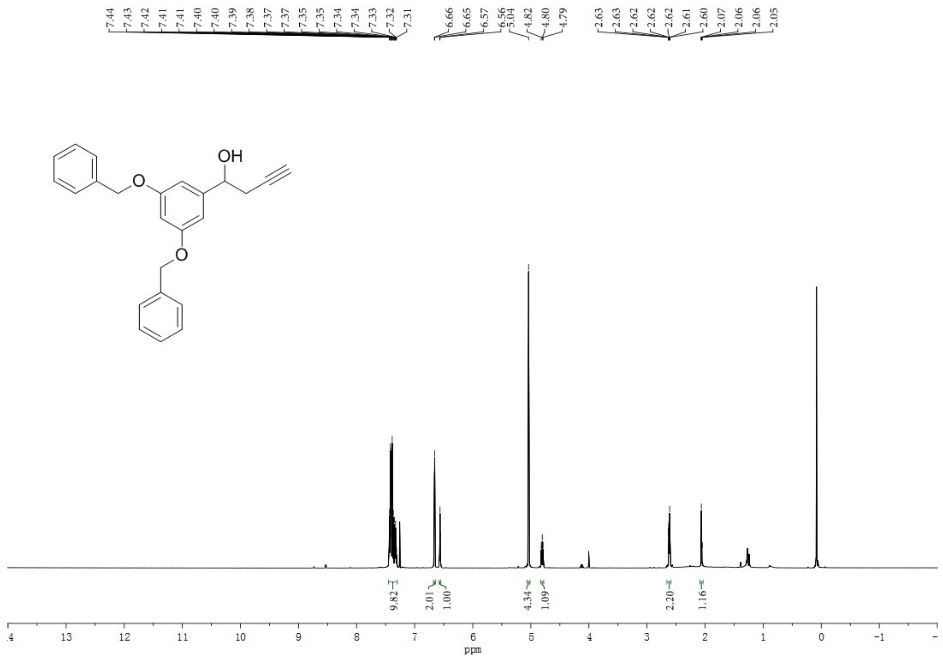

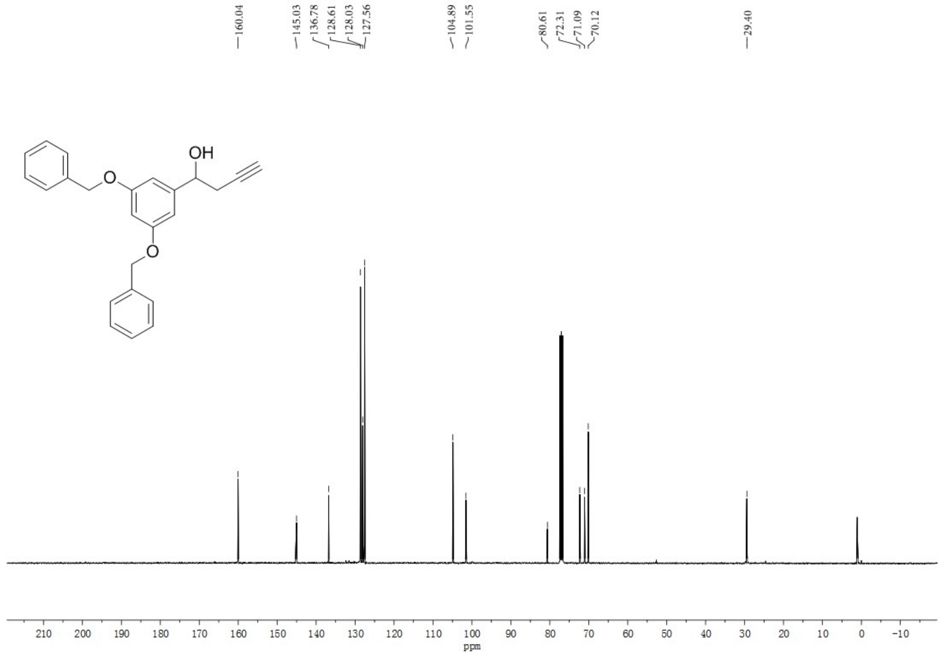

Supplementary Figure 40. ^{1}H NMR spectra for 1-(3, 5-bis(benzyloxy)phenyl)but-3-yn-1-ol

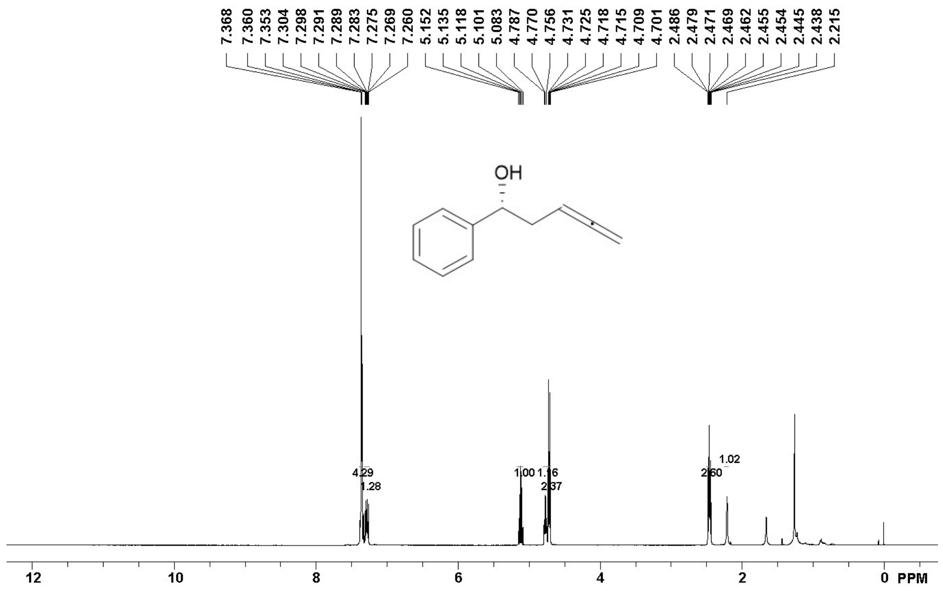

Supplementary Figure 41. **^{1}H NMR spectra for (*R*)-1-phenylpenta-3,4-dien-1-ol**

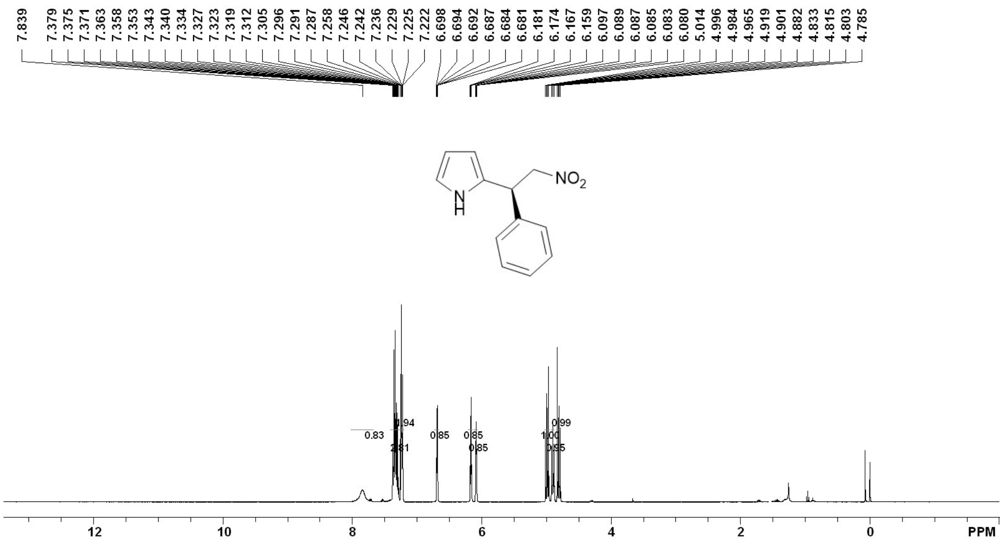

Supplementary Figure 42. ^{1}H NMR spectra for (*R*)-2-(2-nitro-1-phenylethyl)-1*H*-pyrrole

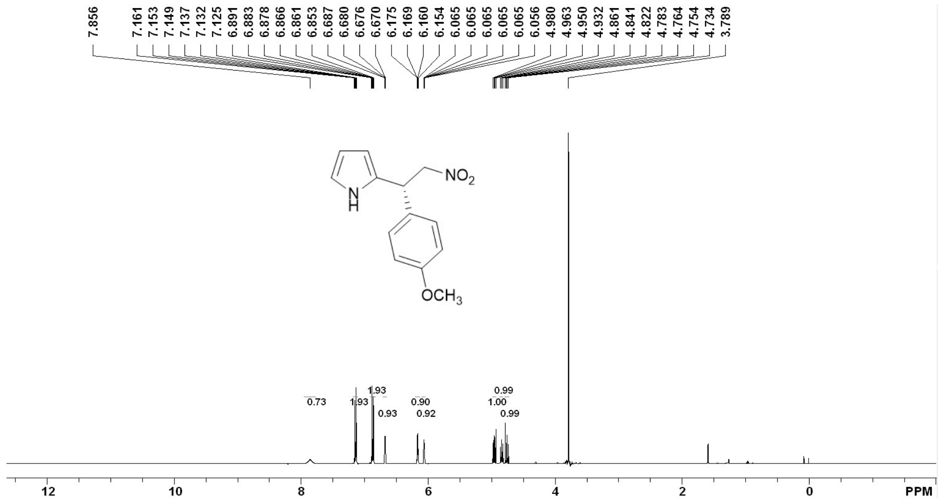

Supplementary Figure 43. ¹H NMR spectra for (*S*)-2-(1-(4-methoxyphenyl)-2-nitroethyl)-1 *H*-pyrrole

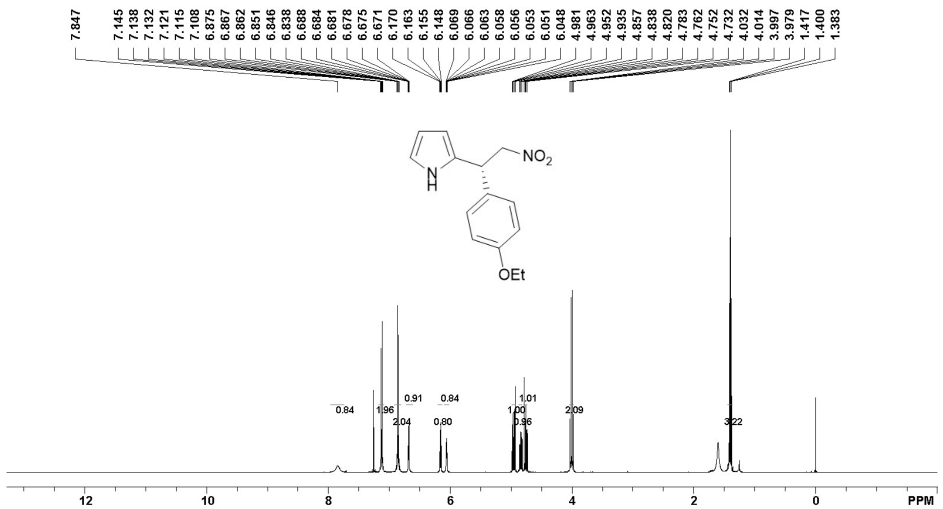

Supplementary Figure 44. ¹H NMR spectra for (*S*)-2-(1-(4-ethoxyphenyl)-2-nitro ethyl)-1 *H*-pyrrole

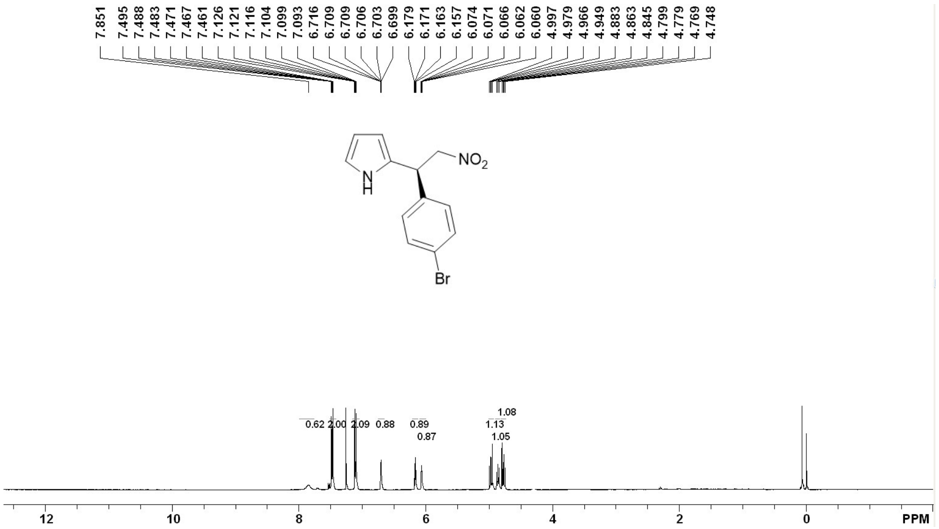

Supplementary Figure 45. ^{1}H NMR spectra for (*R*)-2-(1-(4-bromophenyl)-2-nitro ethyl)-1*H*-pyrrole

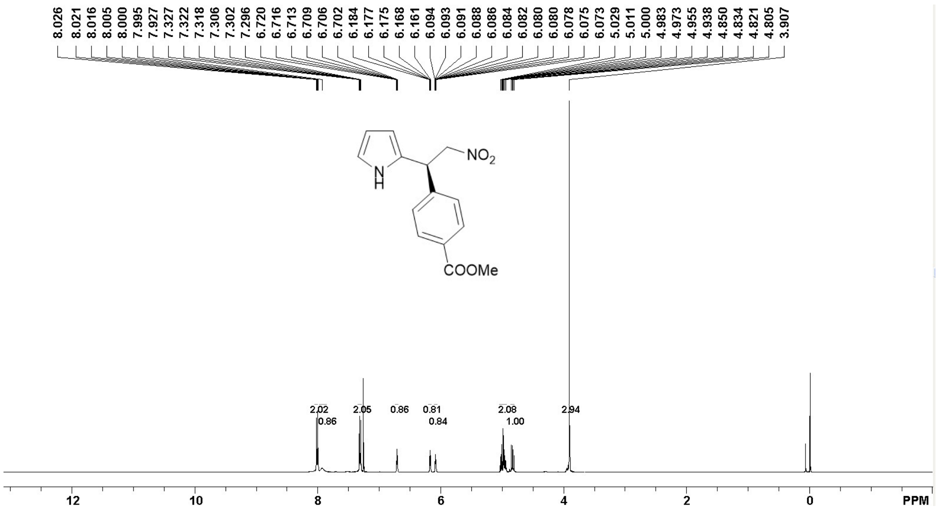

Supplementary Figure 46. ^{1}H NMR spectra for (*R*)-2-(1-(4-methoxycarbonyl)-2-nitroethyl)-1 *H*-pyrrole

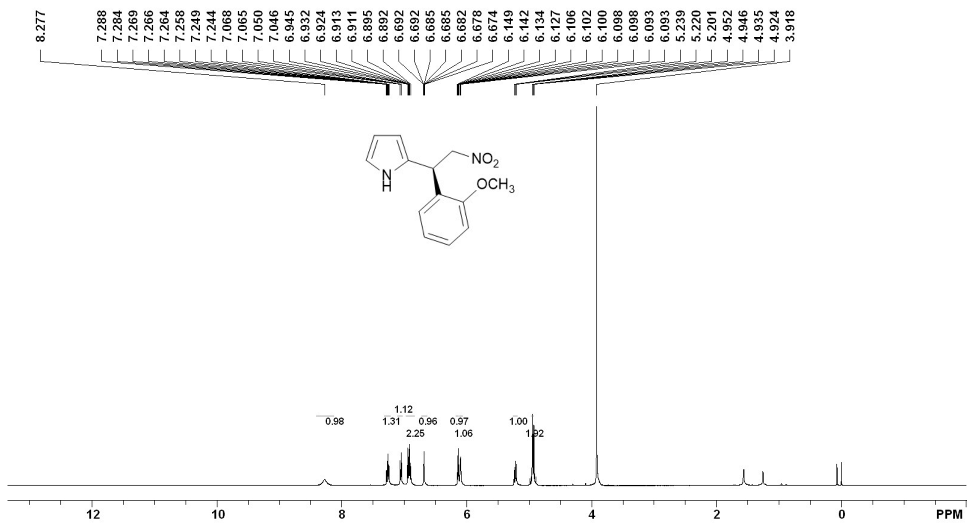

Supplementary Figure 47. ^{1}H NMR spectra for (*R*)-2-(1-(2-methoxyphenyl)-2-nitroethyl)- 1*H*-pyrrole

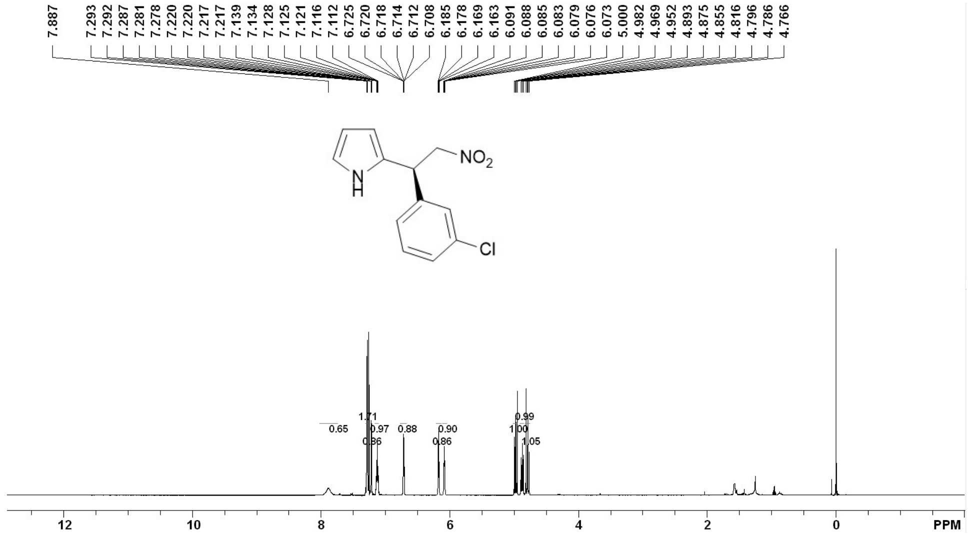

Supplementary Figure 48. ^{1}H NMR spectra for (*R*)-2-(1-(3-chlorophenyl)-2-nitroethyl)- 1*H*-pyrrole

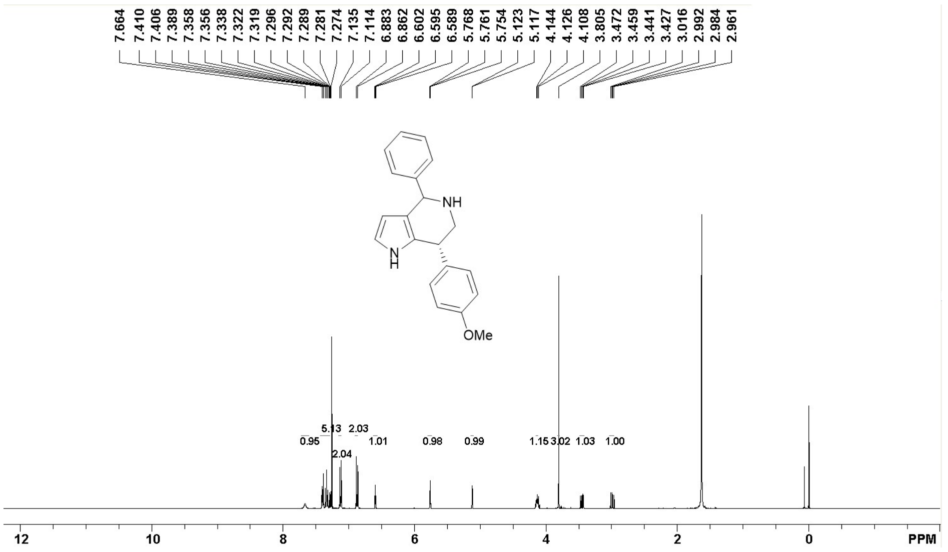

Supplementary Figure 49. ^{1}H NMR spectra for (7*S*)-4-phenyl-7-(4-methoxy phenyl)-4,5,6,7-tetrahydro-1*H*-pyrrolo[3,2-c]pyridine

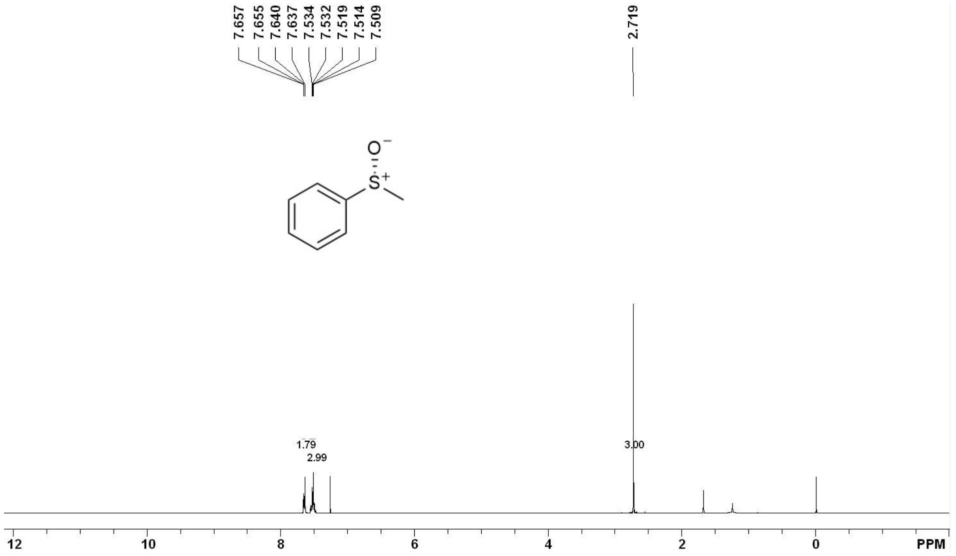

Supplementary Figure 50. ^{1}H NMR spectra for (*S*)-Phenyl methyl sulfoxide

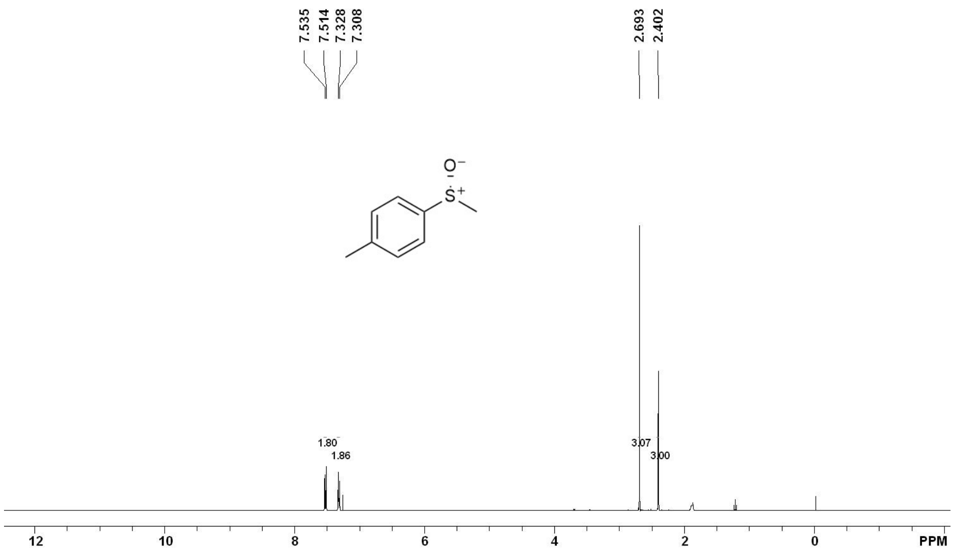

Supplementary Figure 51. ^{1}**H NMR spectra for (*S*)-*p*-Tolyl methyl sulfoxide**

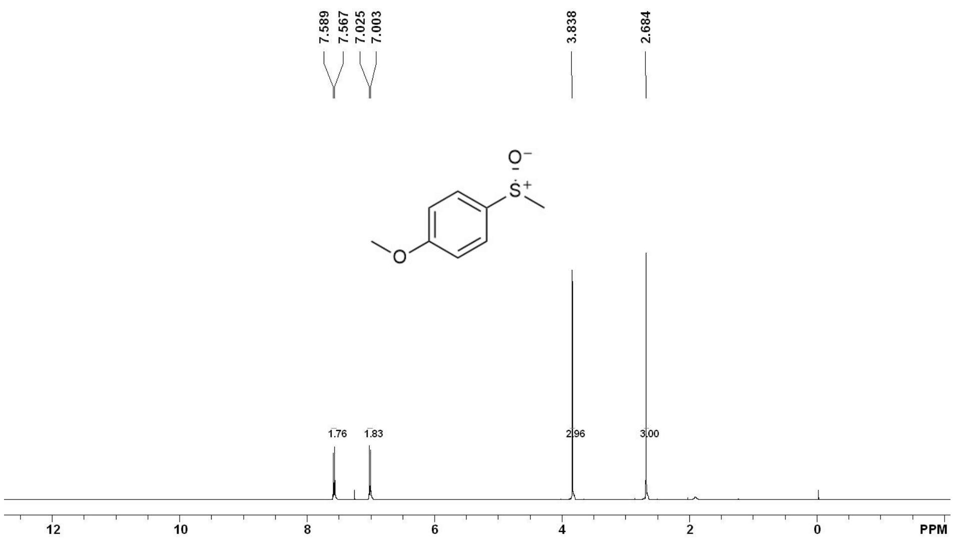

Supplementary Figure 52. ^{1}**H NMR spectra for (*S*)-*p*-Methoxyphenyl methyl sulfoxide**

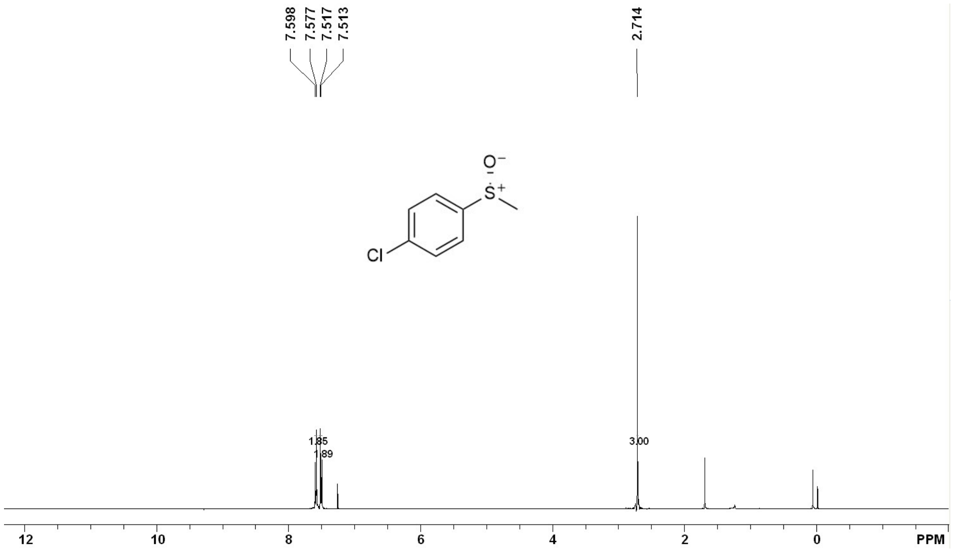

Supplementary Figure 53. ¹H NMR spectra for (*S*)-*p*-Chlorophenyl methylsul foxide

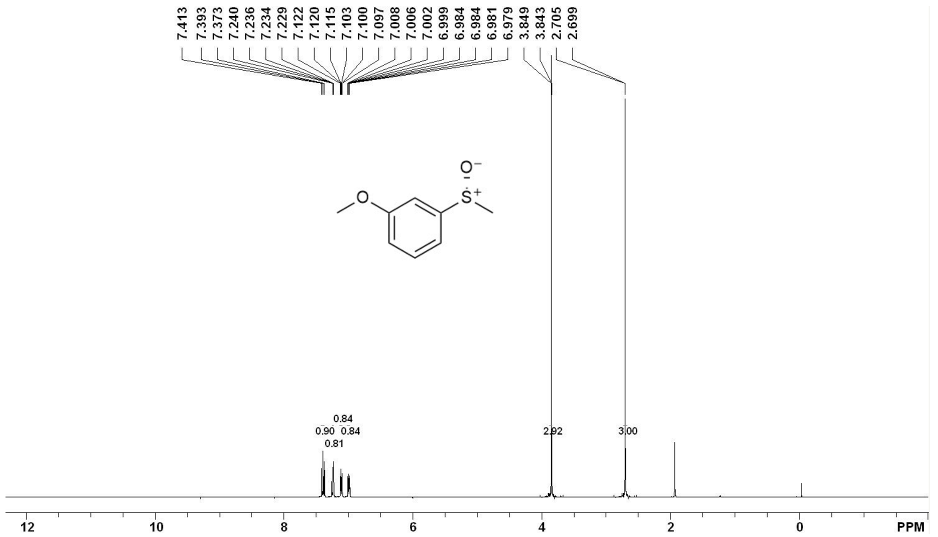

Supplementary Figure 54. ¹H NMR spectra for (*S*)-*m*-Methoxyphenyl methyl sulfoxide

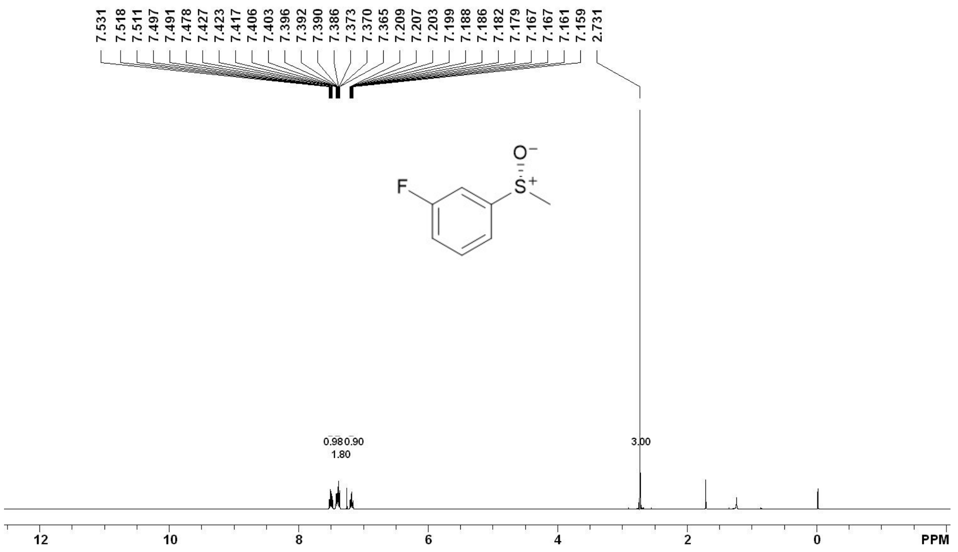

Supplementary Figure 55. ^{1}H NMR spectra for (*S*)-*m*-Fluorophenyl methyl sulfoxide

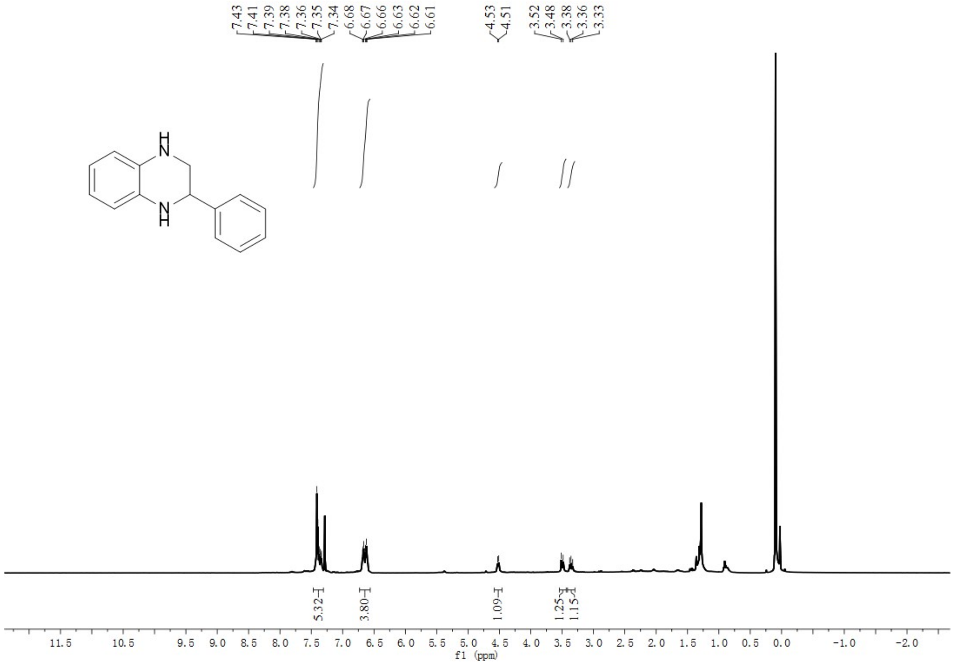

Supplementary Figure 56. ^{1}H NMR spectra for 2-Phenyl-1,2,3,4-tetrahydroqui noxaline

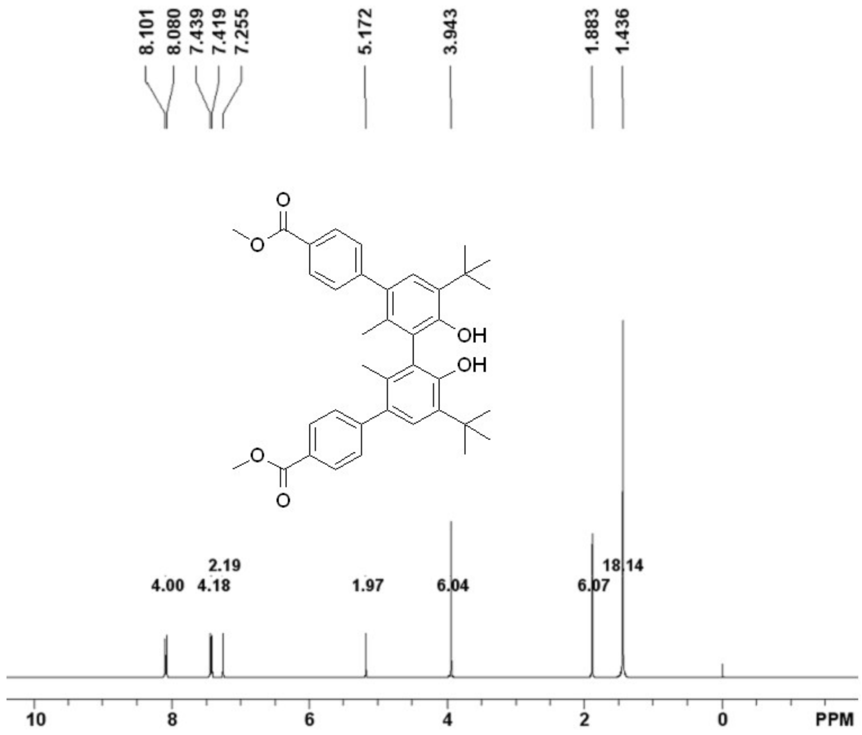

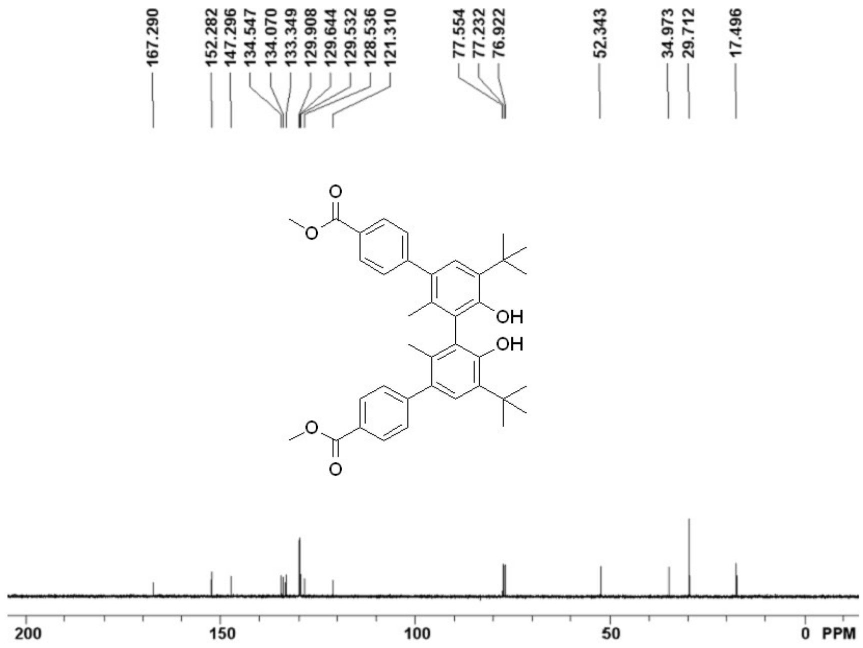

Supplementary Figure 57 ^{1}H NMR and ^{13}C NMR spectra for S2

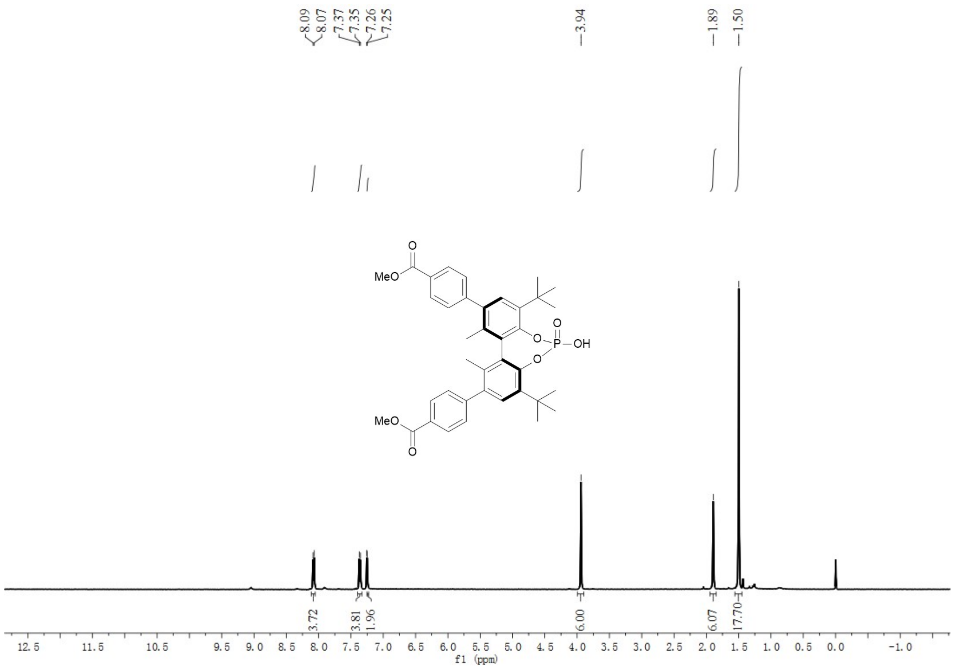

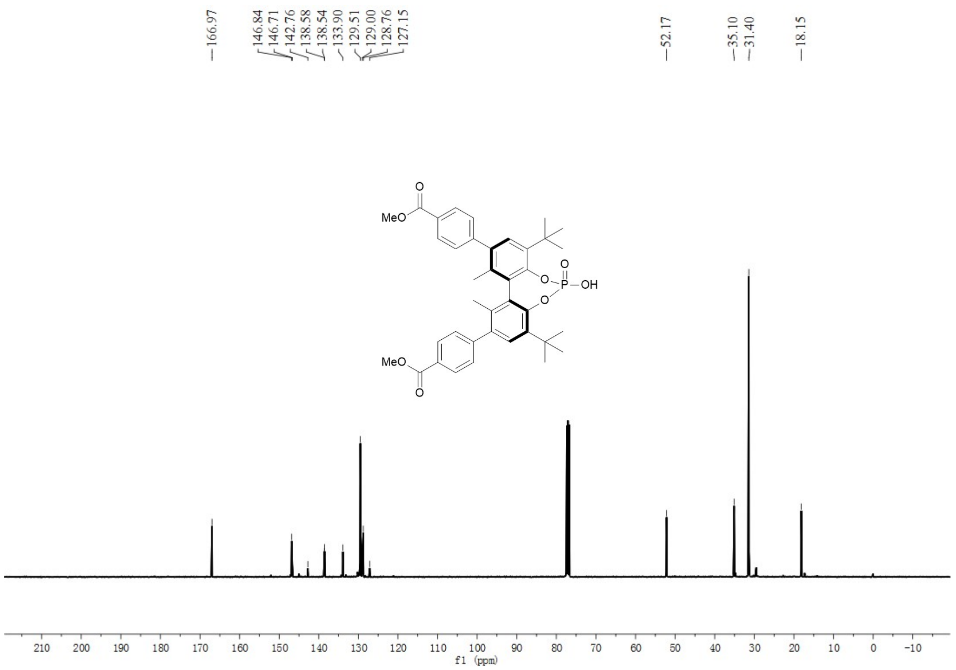

Supplementary Figure 58. ^{1}H NMR and ^{13}C NMR spectra for Me_2L

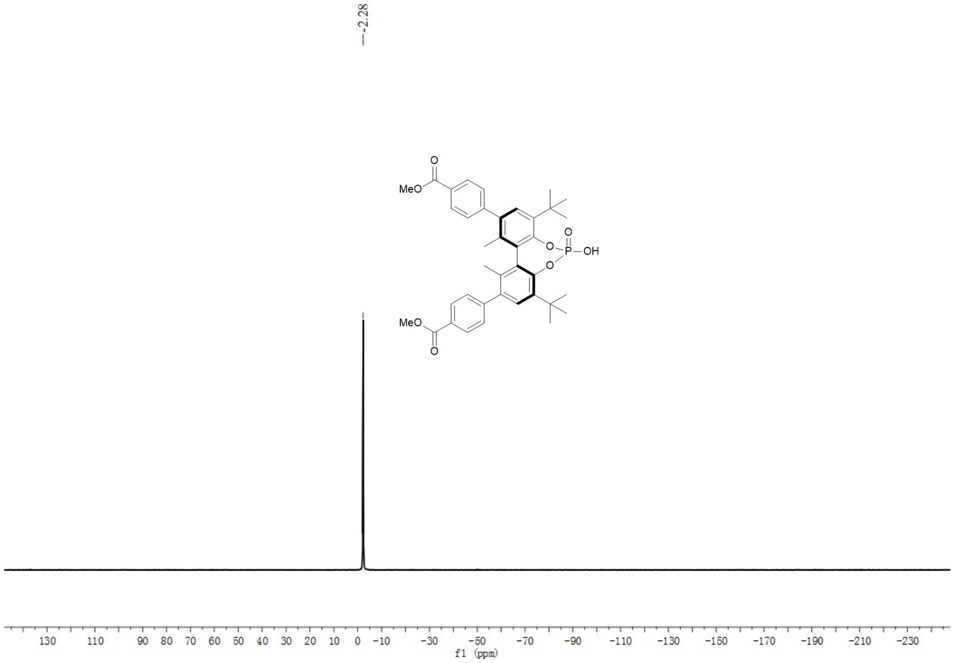

Supplementary Figure 59. ^{31}P NMR spectra for Me_2L

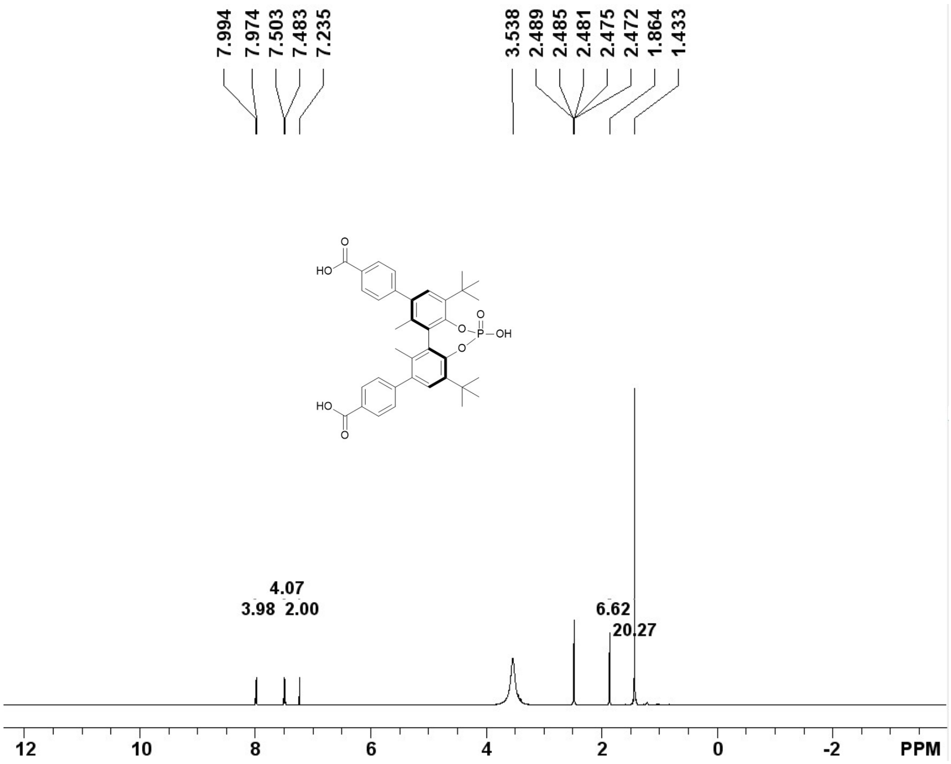

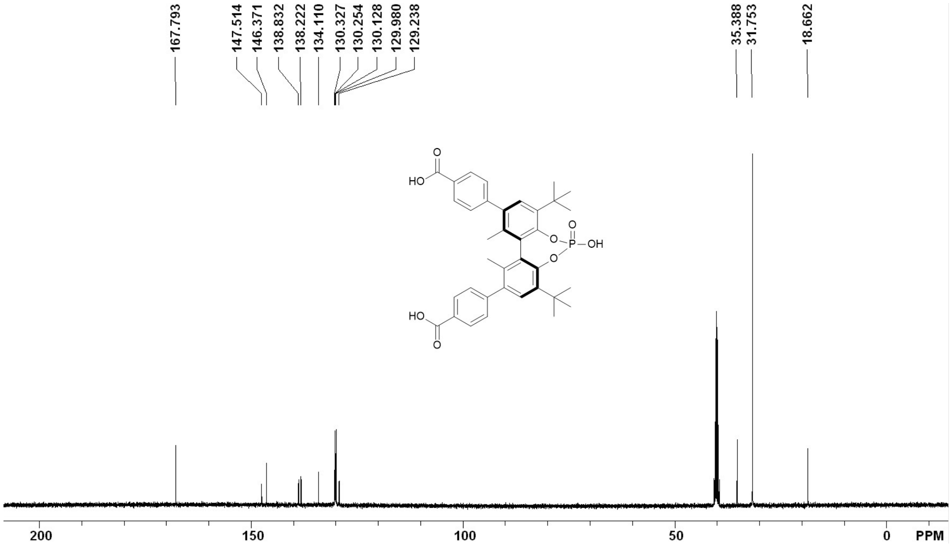

Supplementary Figure 60. **^{1}H NMR and ^{13}C NMR spectra for H_3L**

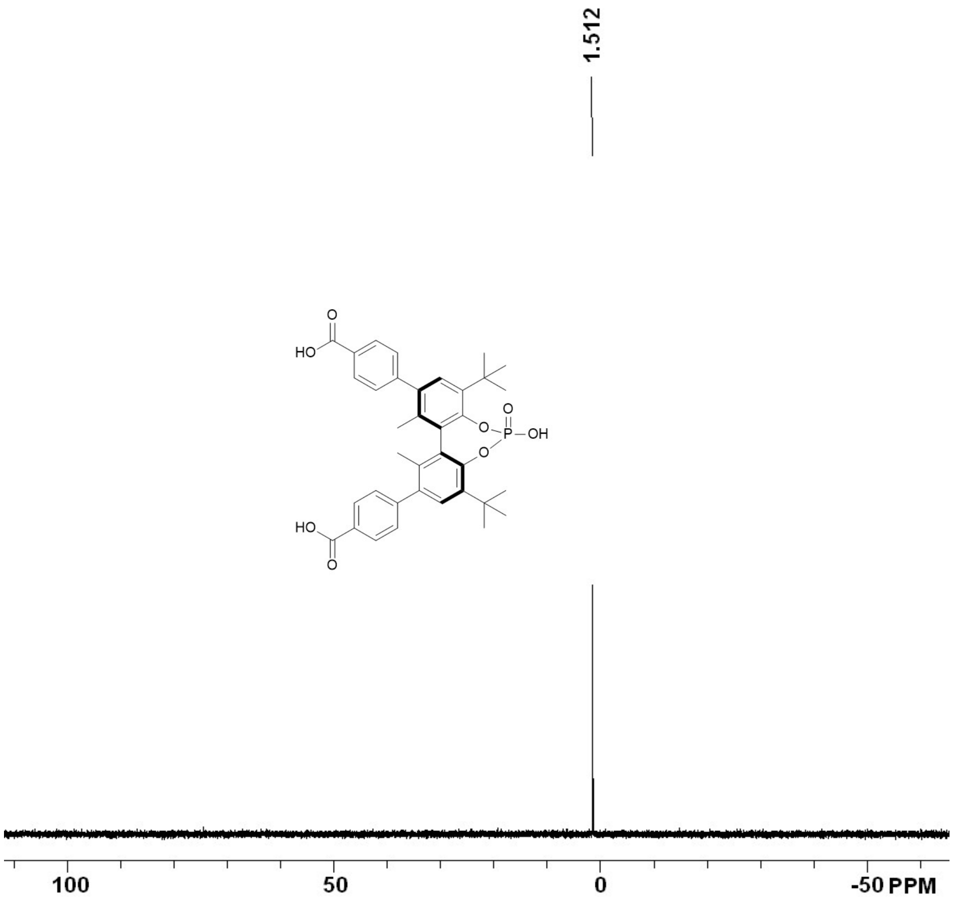

Supplementary Figure 61. ^{31}P NMR spectra for H_3L

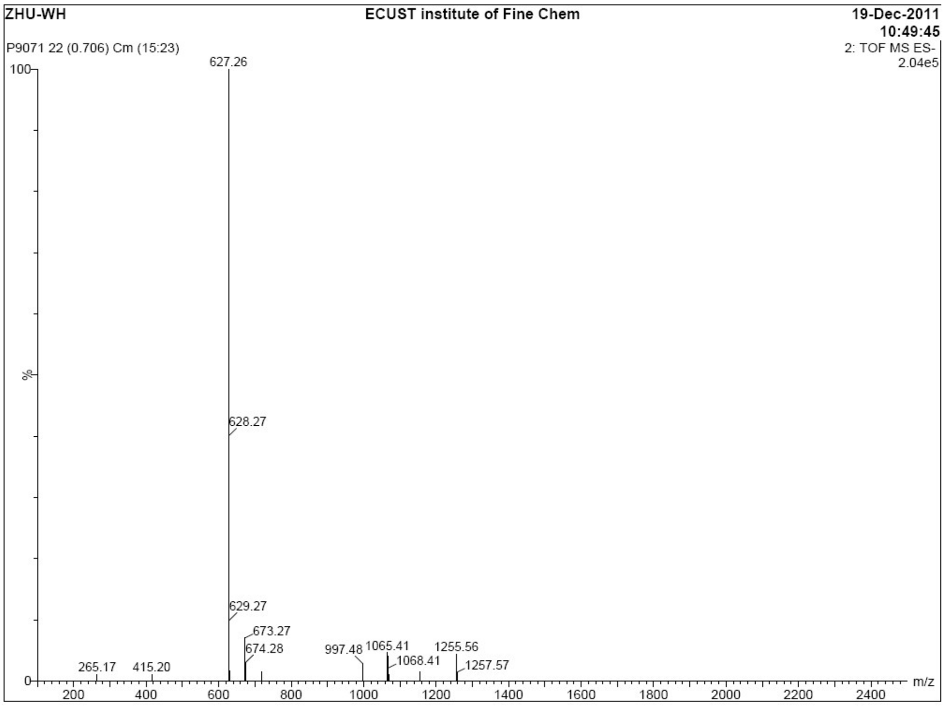

Supplementary Figure 62. ESI-MS for H_3L

Supplementary Table 1. The parameter of unit cell for 1-Cr/Mg/Mn

**H_3L-Cr (1-Cr)**	**H_3L-Mg (1-Mg)**	**H_3L-Mn (1-Mn)**
a = 26.27<br>b = 28.30<br>c = 28.54 V = 21215<br>$\alpha = \beta = \gamma = 90^o$<br>Orthorhombic C	a = 27.29<br>b = 27.47<br>c = 28.88 V = 21647<br>$\alpha = \beta = \gamma = 90^o$<br>Orthorhombic C	a = 27.49<br>b = 28.48<br>c = 29.32 V=22959<br>$\alpha = \beta = \gamma = 90^o$<br>Orthorhombic C

Supplementary Table 2. Allyboration of aldehydes catalyzed by control catalysts[a]

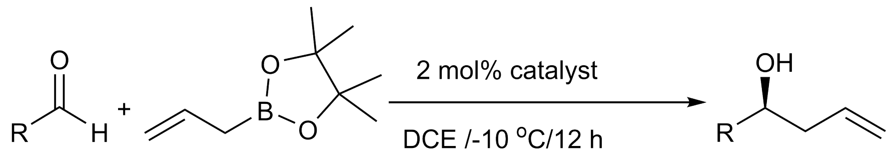

2 3 4

entry	R	catalyst	yield (%)[b]	ee (%)[c]
1	Ph	Me_2L	72	0
2	Ph	H_3L	88	0
3[e]	Ph	H_3L-Cr	75	20 (*S*)
4[f]	Ph	1-Cr	98	83 (*S*)
5[g]	Ph	1-Cr	70	23 (*S*)
6	Ph	--	40	0
7	4-NO_2Ph	Me_2L	78	0
8	4-NO_2Ph	H_3L	85	0
9[e]	4-NO_2Ph	H_3L-Cr	74	35 (*S*)
10	4-ClPh	Me_2L	70	0
11	4-ClPh	H_3L	88	0
12[e]	4-ClPh	H_3L-Cr	71	33 (*S*)
13	DBBA[d]	Me_2L	84	0
14	DBBA[d]	H_3L	88	0
15[e]	DBBA[d]	H_3L-Cr	38	0

[a]Reaction conditions: **2** (0.10 mmol), **3** (0.12 mmol) and **Cat**. (**1-Cr**, 2 mol% loading, based on MOF; H_3**L-Cr**, 6 mol% loading, based on Cr; Me_2**L**/H_3**L**, 0.004 mmol) in DCE (0.8 mL), -10 °C, 12 h. [b]Isolated yield. [c]Determined by chiral HPLC analysis. [d]DBBA = 3,5-dibenzyloxybenzaldehyde. [e]H_3**L**-Cr (6 mol% loading, based on Cr) was used as the catalyst, which cannot dissolve in DCE. [f]After completion of reaction, the supernatant was condensed and analyzed by ^{31}P NMR to detect free phosphoric acid (Supplementary Fig. 1). [g]Unactivated **1-Cr**.

Supplementary Table 3. Propargylation of aldehydes catalyzed by control catalysts[a]

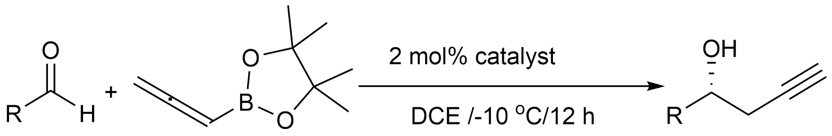

entry	R	catalyst	yield (%)[b]	ee (%)[c]
1	Ph	Me_2L	70	0
2	Ph	H_3L	88	0
3[e]	Ph	H_3L-Cr	67	50 (*R*)
4[f]	Ph	1-Cr	92	92 (*R*)
5[g]	Ph	1-Cr	65	55 (*R*)
6	4-MeOPh	Me_2L	70	0
7	4-MeOPh	H_3L	83	0
8[e]	4-MeOPh	H_3L-Cr	71	42 (*R*)
9	DBBA[d]	Me_2L	78	0
10	DBBA[d]	H_3L	84	0
11[e]	DBBA[d]	H_3L-Cr	0	0

[a]Reaction conditions: **2** (0.10 mmol), **5** (0.15 mmol) and **Cat**. (**1-Cr**, 2 mol% loading, based on MOF; H_3**L-Cr**, 6 mol% loading, based on Cr; Me_2**L**/H_3**L**, 0.004 mmol) in DCE (0.8 mL), -10 °C, 12 h. [b]Isolated yield. [c]Determined by chiral HPLC analysis. [d]DBBA = 3,5-dibenzyloxybenzaldehyde. [e]H_3**L**-Cr (6 mol% loading, based on Cr) was used as the catalyst, which cannot dissolve in DCE. [f]After completion of reaction, the supernatant was condensed and analyzed by ^{31}P NMR to detect free phosphoric acid (Supplementary Fig. 2). [g]Unactivated **1-Cr**.

Supplementary Table 4. Friedel-Crafts alkylation of pyrrole with nitroalkenes catalyzed by control catalysts[a]

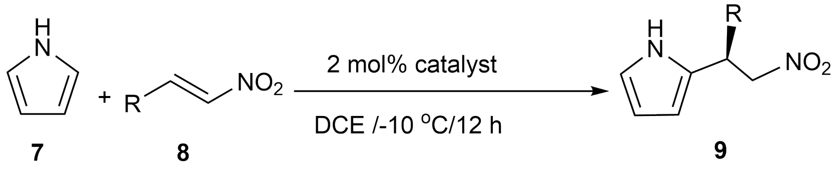

entry	R	catalyst	yield (%)[b]	ee (%)[c]
1	Ph	Me_2L	63	0
2	Ph	H_3L	88	0
3[d]	Ph	H_3L-Mg	50	29 (*S*)
4[e]	Ph	1- Mg	89	90 (*S*)
5[f]	Ph	1- Mg	43	31 (*S*)
6	4-MeOPh	Me_2L	61	0
7	4-MeOPh	H_3L	80	0
8[d]	4-MeOPh	H_3L- Mg	65	38 (*S*)
9	4-EtOPh	Me_2L	65	0
10	4-EtOPh	H_3L	80	0
11[d]	4-EtOPh	H_3L- Mg	55	35 (*S*)

[a]Reaction conditions: 7 (0.3 mmol), **8** (0.1 mmol), **Cat**. (**1-Mg**, 2 mol% loading, based on MOF; H_3**L-Mg**, 6 mol% loading, based on Mg; Me_2**L**/H_3**L**, 0.004 mmol) in DCE (0.8 mL), -10 °C, 12 h. [b]Isolated yields. [c]Determined by chiral HPLC analysis. [d]H_3**L**-Cr (6 mol% loading , based on Mg) was used as the catalyst. [e]After completion of reaction, the supernatant was condensed and analyzed by ^{31}P NMR to detect free phosphoric acid (Supplementary Fig. 3). [f]Unactivated **1-Mg**.

Supplementary Table 5. Oxidation of sulfide catalyzed by control catalysts[a]

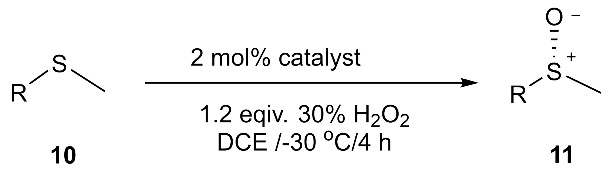

entry	R	catalyst	conv. (%)[b]	yield (%)[b]	ee (%)[c]
1	Ph	Me_2L	59	48	0
2	Ph	H_3L	75	50	0
3[e]	Ph	H_3L-Mn	64	50	41 (*S*)
4[f]	Ph	1-Mn	99	93	92 (*S*)
5[g]	Ph	1-Mn	53	41	47 (*S*)
6	4-MePh	Me_2L	56	47	0
7	4-MePh	H_3L	60	41	0
8[e]	4-MePh	H_3L-Mn	71	65	51 (*S*)
9	4-MeOPh	Me_2L	65	54	0
10	4-MeOPh	H_3L	72	59	0
11[e]	4-MeOPh	H_3L-Mn	55	47	44 (*S*)

[a]Reaction conditions: **10** (0.1 mmol), **Cat**. (**1-Mn**, 2 mol% loading, based on MOF; H_3**L**-Mn, 6 mol% loading, based on Mn; Me_2**L**/H_3**L**, 0.004 mmol), 1.2 equiv. aqueous H_2O_2 in DCE (0.8 mL), -30 °C, 4 h. Over-oxidized sulfone byproducts were detected. [b]Determined by ^{1}H NMR analysis. [c]Isolated yields. [d]Determined by chiral HPLC analysis. [e]H_3**L**-Cr (6 mol% loading, based on Mn) was used as the catalyst. [f]After completion of reaction, the supernatant was condensed and analyzed by ^{31}P NMR to detect free phosphoric acid (Supplementary Fig. 4). [g]Unactivated **1-Mn**.

Supplementary Table 6. Hydrogenation of quinoxalines with Hanztsch esters applied as the hydrogen source[a]

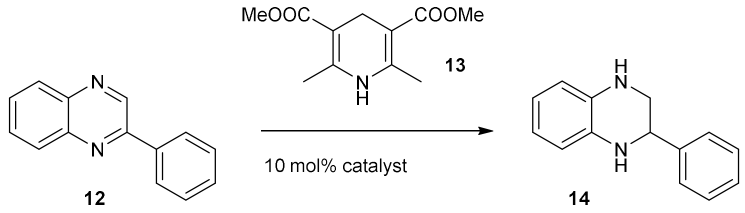

**entry**	**catalyst**	**yield (%)[b]**
1	1-Cr	0
2	1-Mn	0
3	1-Mg	0
4	Me_2L	90
5	H_3L	98

[a]Reaction conditions: **12** (0.1 mmol), **13** (2.4 equiv), **Cat.** (MOFs, 10 mol% loading, based on MOF; Me_2**L**/H_3**L**, 0.02 mmol), $CHCl_3$ (0.5 mL), 60 °C, 24 h. [b]Isolated yields.

Supplementary Table 7. X-ray crystallography of 1-Cr/Mn/Ga[5]

Compound	**1-Cr**	**1-Mn**	**1-Ga**
Empirical formula	C_{78} $H_{79}Cr_3O_{23}P_2$	$C_{72}H_{72}$ $Mn_3O_{18}P_2$	$C_{78}H_{79}Ga_3O_{23}P_2$
Formula weight	1602.35	1452.6	1655.51
Temperature (K)	123(2)	123(2)	123(2)
Wavelength (Å)	1.54178	1.5478	1.5478
Crystal system	Orthorhombic	Orthorhombic	Orthorhombic
Space group	$C222_1$	$C222_1$	$C222_1$
	a = 26.8122(5),	a = 27.3307(12)	a = 27.4162(7)
Unit cell dimensions	b = 29.1445(5),	b = 29.1106(12)	b = 29.2521(8)
	c = 29.4502(7),	c = 29.5760(12)	c = 29.6153(7)
	$\alpha=\beta=\gamma=90°$	$\alpha=\beta=\gamma=90°$	$\alpha=\beta=\gamma=90°$
Volume (Å^3),	23013.2(7) ,	23531.1(17),	23750.9(11),
Z	8	8	8
Density (calcd. mg/m^3)	0.925	0.820	0.926
Absorption coeff. (mm^{-1})	2.980	3.181	1.461
F(000)	6664	6024	6832
θ range data collection	2.24 ~ 55.00	2.22 ~ 60	4.25 ~ 68.41
Limiting indices	$-16\leqslant h\leqslant 28$,	$-30\leqslant h\leqslant 30$,	$-32\leqslant h\leqslant 30$,
	$-30\leqslant k\leqslant 30$,	$-32\leqslant k\leqslant 31$,	$-28\leqslant k\leqslant 34$,
	$-31\leqslant l\leqslant 31$	$-33\leqslant l\leqslant 33$	$-27\leqslant l\leqslant 35$
Reflections collected	27381	50084	39265
Independent reflections	13840 (R_{int} = 0.0260)	17096 (R_{int} = 0.0876)	20235 (R_{int} = 0.0529)
Completeness to theta	55.00/ 98.8 %	60.00/99.3%	68.41/97.9%
Refinement method	Full-matrix least-squares on F^2		
Data / restraints / parameters	13840 /33 /790	17096 /19 /760	20356 /36 /787
Goodness-of-fit on F^2	1.158	1.050	1.101
Final R indices [I>2σ(I)]	R_1 = 0.0893,	R_1 = 0.0944,	R_1 = 0.0851,

	wR_2 = 0.2676	wR_2 = 0.2489	wR_2 = 0.2356
R indices (all data)	R_1 = 0.0919, wR_2 = 0.2788	R_1 = 0.1094, wR_2 = 0.2672	R_1 = 0.1187, wR_2 = 0.2563
Absolute structure parameter	0.282(6)	0.358(7)	-0.09(3)
Largest diff. peak and hole (e.Å^{-3})	1.535 and -0.780	1.100 and -0.664	0.623 and -0.855

Supplementary Table 8. X-ray crystallography of 1-Zr and 1-Ti.

Compound	**1-Zr**	**1-Ti**
Empirical formula	$C_{78}H_{82}O_{26}P_2Zr_3$	$C_{78}H_{82}O_{26}P_2Ti_3$
Formula weight	1771.04	1641.08
Temperature (K)	123(2)	123(2)
Wavelength (Å)	1.54178	0.71073
Crystal system	Orthorhombic	Orthorhombic
Space group	$C222_1$	$C222_1$
	a = 27.0284(6)	a = 27.341(11)
Unit cell dimensions	b = 29.1892(6)	b = 29.274(10)
	c = 29.5728(6)	c = 29.662(10)
	$\alpha = \beta = \gamma = 90^{o}$	$\alpha = \beta = \gamma = 90^{o}$
Volume (Å^3)	23331.1(8)	23741(14)
Z	8	8
Density (calcd. mg/m^3)	1.008	0.918
Absorption coeff. (mm^{-1})	2.868	0.276
F(000)	7264	6832
θ range data collection	2.23 ~ 55.00	1.02 ~ 25
Limiting indices	$-19 \leqslant h \leqslant 28$,	$-32 \leqslant h \leqslant 22$,
	$-30 \leqslant k \leqslant 30$,	$-28 \leqslant k \leqslant 34$,
	$-22 \leqslant l \leqslant 31$	$-35 \leqslant l \leqslant 35$
Reflections collected	26597	67695
Independent reflections	13060 (R_{int} = 0.0325)	20885 (R_{int} = 0.1818)
Completeness to theta	55.00/ 97.0 %	25.00/99.8%
Refinement method	Full-matrix least-squares on F^2	
Data / restraints / parameters	13060 /79/815	20885 /46 /797
Goodness-of-fit on F^2	1.183	1.020
Final R indices [I>2σ(I)]	R_1 = 0.0795,	R_1 = 0.0941,
	wR_2 = 0.2457	wR_2 = 0.1863

R indices (all data)	R_1 = 0.0919, wR_2 = 0.2527	R_1 = 0.2133, wR_2 = 0.2130
Absolute structure parameter	0.138(14)	-0.27(4)
Largest diff. peak and hole (e.Å^{-3})	0.854 and -1.230	0.684 and -0.501

Supplementary Table 9. X-ray crystallography of Me_2L-Mg and Me_2L-Mn.

Compound	Me_2L-Mg	Me_2L-Mn
Empirical formula	$C_{242}H_{240}Mg_3O_{59}P_6$	$C_{242}H_{240}Mn_3O_{59}P_6$
Formula weight	4327.06	4442.97
Temperature (K)	173.0	173.0
Wavelength (Å)	1.54178	1.54178
Crystal system	Orthorhombic	Orthorhombic
Space group	$P2_12_12_1$	$P2_12_12_1$
	a = 21.9287(12)	a = 22.1113(18)
Unit cell dimensions	b = 34.0887(18)	b = 33.662(3)
	c = 37.283(2)	c = 37.400(3)
	$\alpha = \beta = \gamma = 90^o$	$\alpha = \beta = \gamma = 90^o$
Volume ($Å^3$)	27870(3)	27837(4)
Z	4	4
Density (calcd. mg/m^3)	1.031	1.060
Absorption coeff. (mm^{-1})	0.973	1.968
F(000)	9112	9316
θ range data collection	2.337 ~ 59.218	2.321 ~ 52.873
Limiting indices	$-24 \leqslant h \leqslant 21$,	$-22 \leqslant h \leqslant 15$,
	$-37 \leqslant k \leqslant 37$,	$-34 \leqslant k \leqslant 34$,
	$-38 \leqslant l \leqslant 41$	$-36 \leqslant l \leqslant 38$
Reflections collected	135669	78889
Independent reflections	39778 (R_{int} = 0.0840)	31396 (R_{int} = 0.0568)
Completeness to theta	67.679/79.6 %	67.679/63.8%
Refinement method	Full-matrix least-squares on F^2	
Data / restraints / parameters	39778/2750/2500	31396/4462/2580
Goodness-of-fit on F^2	1.244	1.623
Final R indices [I>2σ(I)]	R_1 = 0.1313,	R_1 = 0.1394,
	wR_2 = 0.3150	wR_2 = 0.3632

R indices (all data)	R_1 = 0.1542,<br>wR_2 = 0.3439	R_1 = 0.1489,<br>wR_2 = 0.3769
Absolute structure parameter	0.104(14)	0.112(4)
Largest diff. peak and hole (e.Å^{-3})	1.488 and -0.552	1.103 and -0.688

Supplementary Table 10. Selected bond lengths [Å] and angles [º] for 1-Cr.

Cr(1)-O(9)#1	1.917(4)
Cr(1)-O(5)	1.961(5)
Cr(1)-O(15)#2	1.983(5)
Cr(1)-O(17)	2.000(6)
Cr(2)-O(6)	2.267(5)
Cr(2)-O(14)	2.277(4)
Cr(2)-O(2)#3	2.324(5)
Cr(2)-O(16)#2	2.332(5)
Cr(2)-O(8)#4	2.365(4)
Cr(2)-O(10)#1	2.369(4)
Cr(3)-O(7)#4	1.911(4)
Cr(3)-O(18)	1.947(6)
Cr(3)-O(1)#3	1.954(5)
Cr(3)-O(13)	1.959(6)
O(9)#1-Cr(1)-O(5)	113.2(2)
O(9)#1-Cr(1)-O(15)#2	128.8(3)
O(5)-Cr(1)-O(15)#2	111.5(3)
O(9)#1-Cr(1)-O(17)	100.8(2)
O(5)-Cr(1)-O(17)	100.9(3)
O(15)#2-Cr(1)-O(17)	94.0(3)
O(6)-Cr(2)-O(14)	119.4(2)
O(6)-Cr(2)-O(2)#3	152.67(19)
O(14)-Cr(2)-O(2)#3	82.6(2)
O(6)-Cr(2)-O(16)#2	81.34(19)
O(14)-Cr(2)-O(16)#2	152.55(19)
O(2)#3-Cr(2)-O(16)#2	84.08(19)
O(6)-Cr(2)-O(8)#4	81.36(16)
O(14)-Cr(2)-O(8)#4	90.92(17)

O(2)#3-Cr(2)-O(8)#4	82.50(16)
O(16)#2-Cr(2)-O(8)#4	111.03(17)
O(6)-Cr(2)-O(10)#1	88.52(17)
O(14)-Cr(2)-O(10)#1	84.47(16)
O(2)#3-Cr(2)-O(10)#1	110.88(17)
O(16)#2-Cr(2)-O(10)#1	77.99(15)
O(8)#4-Cr(2)-O(10)#1	165.04(17)
O(7)#4-Cr(3)-O(18)	109.6(2)
O(7)#4-Cr(3)-O(1)#3	123.1(2)
O(18)-Cr(3)-O(1)#3	95.4(3)
O(7)#4-Cr(3)-O(13)	110.2(2)
O(18)-Cr(3)-O(13)	100.5(3)
O(1)#3-Cr(3)-O(13)	114.3(2)

Symmetry transformations used to generate equivalent atoms:

#1 -x+1,y,-z+1/2 #2 x+1/2,-y+1/2,-z #3 -x+3/2,y+1/2,-z+1/2

#4 x,-y,-z #5 -x+3/2,y-1/2,-z+1/2 #6 x-1/2,-y+1/2,-z

Supplementary Table 11. Selected bond lengths [Å] and angles [º] for 1-Mn.

Mn(1)-O(9)#1	1.927(5)
Mn(1)-O(5)	1.957(6)
Mn(1)-O(15)#2	1.979(5)
Mn(1)-O(17)	2.040(6)
Mn(2)-O(6)	2.290(5)
Mn(2)-O(14)	2.292(5)
Mn(2)-O(2)#3	2.299(5)
Mn(2)-O(16)#2	2.326(5)
Mn(2)-O(10)#1	2.350(4)
Mn(2)-O(8)#4	2.360(5)
Mn(3)-O(7)#4	1.888(5)
Mn(3)-O(1)#3	1.932(6)
Mn(3)-O(13)	1.935(6)
Mn(3)-O(18)	1.988(6)
O(9)#1-Mn(1)-O(5)	113.3(2)
O(9)#1-Mn(1)-O(15)#2	127.5(3)
O(5)-Mn(1)-O(15)#2	111.9(3)
O(9)#1-Mn(1)-O(17)	101.3(2)
O(5)-Mn(1)-O(17)	100.3(3)
O(15)#2-Mn(1)-O(17)	95.6(3)
O(6)-Mn(2)-O(14)	117.5(2)
O(6)-Mn(2)-O(2)#3	153.8(2)
O(14)-Mn(2)-O(2)#3	83.1(2)
O(6)-Mn(2)-O(16)#2	81.9(2)
O(14)-Mn(2)-O(16)#2	155.0(2)
O(2)#3-Mn(2)-O(16)#2	83.9(2)
O(6)-Mn(2)-O(10)#1	88.81(19)
O(14)-Mn(2)-O(10)#1	85.39(17)

O(2)#3-Mn(2)-O(10)#1	109.95(19)
O(16)#2-Mn(2)-O(10)#1	79.17(17)
O(6)-Mn(2)-O(8)#4	81.39(18)
O(14)-Mn(2)-O(8)#4	89.52(18)
O(2)#3-Mn(2)-O(8)#4	82.95(18)
O(16)#2-Mn(2)-O(8)#4	109.90(19)
O(10)#1-Mn(2)-O(8)#4	165.37(19)
O(7)#4-Mn(3)-O(1)#3	125.6(3)
O(7)#4-Mn(3)-O(13)	110.3(2)
O(1)#3-Mn(3)-O(13)	114.0(3)
O(7)#4-Mn(3)-O(18)	100.7(3)
O(1)#3-Mn(3)-O(18)	98.5(4)
O(13)-Mn(3)-O(18)	103.0(4)

Symmetry transformations used to generate equivalent atoms:

#1 -x,y,-z+3/2 #2 x+1/2,-y+1/2,-z+2 #3 -x+1/2,y-1/2,-z+3/2

#4 x,-y+1,-z+2 #5 -x+1/2,y+1/2,-z+3/2 #6 x-1/2,-y+1/2,-z+2

Supplementary Table 12. Selected bond lengths [Å] and angles [º] for 1-Ga.

Ga(1)-O(5)	1.939(5)
Ga(1)-O(9)#1	1.947(4)
Ga(1)-O(15)#2	1.993(4)
Ga(1)-O(17)	2.028(5)
Ga(2)-O(14)	2.257(4)
Ga(2)-O(6)	2.292(4)
Ga(2)-O(10)#1	2.339(4)
Ga(2)-O(8)#3	2.339(4)
Ga(2)-O(2)#4	2.342(5)
Ga(2)-O(16)#2	2.347(5)
Ga(3)-O(7)#3	1.952(4)
Ga(3)-O(18)	1.956(4)
Ga(3)-O(1)#4	1.958(5)
Ga(3)-O(13)	1.979(5)
O(5)-Ga(1)-O(9)#1	111.3(2)
O(5)-Ga(1)-O(15)#2	114.5(3)
O(9)#1-Ga(1)-O(15)#2	127.8(3)
O(5)-Ga(1)-O(17)	102.2(3)
O(9)#1-Ga(1)-O(17)	101.8(2)
O(15)#2-Ga(1)-O(17)	91.9(2)
O(14)-Ga(2)-O(6)	120.8(2)
O(14)-Ga(2)-O(10)#1	85.67(15)
O(6)-Ga(2)-O(10)#1	88.51(17)
O(14)-Ga(2)-O(8)#3	88.92(17)
O(6)-Ga(2)-O(8)#3	83.20(15)
O(10)#1-Ga(2)-O(8)#3	166.01(19)
O(14)-Ga(2)-O(2)#4	81.55(19)
O(6)-Ga(2)-O(2)#4	153.29(18)

O(10)#1-Ga(2)-O(2)#4	109.00(18)
O(8)#3-Ga(2)-O(2)#4	82.87(16)
O(14)-Ga(2)-O(16)#2	153.10(19)
O(6)-Ga(2)-O(16)#2	81.57(19)
O(10)#1-Ga(2)-O(16)#2	79.87(16)
O(8)#3-Ga(2)-O(16)#2	109.85(18)
O(2)#4-Ga(2)-O(16)#2	81.91(18)
O(7)#3-Ga(3)-O(18)	105.5(2)
O(7)#3-Ga(3)-O(1)#4	125.8(2)
O(18)-Ga(3)-O(1)#4	97.7(2)
O(7)#3-Ga(3)-O(13)	110.6(2)
O(18)-Ga(3)-O(13)	100.7(3)
O(1)#4-Ga(3)-O(13)	112.1(2)

Symmetry transformations used to generate equivalent atoms:

#1 -x+1,y,-z+1/2 #2 x+1/2,-y+1/2,-z #3 x,-y,-z

#4 -x+3/2,y+1/2,-z+1/2 #5 -x+3/2,y-1/2,-z+1/2 #6 x-1/2,-y+1/2,-z

Supplementary Table 13. Selected bond lengths [Å] and angles [º] for 1-Zr.

Zr(1)-O(9)#1	1.930(4)
Zr(1)-O(5)	1.939(6)
Zr(1)-O(17)	1.999(5)
Zr(1)-O(15)#2	2.008(6)
Zr(2)-O(14)	2.271(4)
Zr(2)-O(6)	2.274(4)
Zr(2)-O(2)#3	2.292(4)
Zr(2)-O(16)#2	2.349(5)
Zr(2)-O(10)#1	2.353(4)
Zr(2)-O(8)#4	2.365(4)
Zr(3)-O(7)#4	1.918(4)
Zr(3)-O(13)	1.953(6)
Zr(3)-O(1)#3	1.965(5)
Zr(3)-O(18)	1.975(6)
O(9)#1-Zr(1)-O(5)	113.1(2)
O(9)#1-Zr(1)-O(17)	100.9(2)
O(5)-Zr(1)-O(17)	102.3(3)
O(9)#1-Zr(1)-O(15)#2	127.4(3)
O(5)-Zr(1)-O(15)#2	112.3(3)
O(17)-Zr(1)-O(15)#2	94.1(3)
O(14)-Zr(2)-O(6)	116.87(18)
O(14)-Zr(2)-O(2)#3	83.26(19)
O(6)-Zr(2)-O(2)#3	153.54(19)
O(14)-Zr(2)-O(16)#2	155.38(18)
O(6)-Zr(2)-O(16)#2	82.07(19)
O(2)#3-Zr(2)-O(16)#2	84.6(2)
O(14)-Zr(2)-O(10)#1	86.00(15)
O(6)-Zr(2)-O(10)#1	88.76(16)
O(2)#3-Zr(2)-O(10)#1	110.79(17)

O(16)#2-Zr(2)-O(10)#1	78.46(16)
O(14)-Zr(2)-O(8)#4	88.82(16)
O(6)-Zr(2)-O(8)#4	80.86(15)
O(2)#3-Zr(2)-O(8)#4	82.71(17)
O(16)#2-Zr(2)-O(8)#4	110.74(17)
O(10)#1-Zr(2)-O(8)#4	164.80(16)
O(7)#4-Zr(3)-O(13)	110.1(2)
O(7)#4-Zr(3)-O(1)#3	124.3(3)
O(13)-Zr(3)-O(1)#3	113.2(3)
O(7)#4-Zr(3)-O(18)	108.3(2)
O(13)-Zr(3)-O(18)	103.8(3)
O(1)#3-Zr(3)-O(18)	93.9(3)

Symmetry transformations used to generate equivalent atoms:

#1 -x+1,y,-z+1/2 #2 x+1/2,-y+1/2,-z #3 -x+3/2,y+1/2,-z+1/2

#4 x,-y,-z #5 -x+3/2,y-1/2,-z+1/2 #6 x-1/2,-y+1/2,-z

Supplementary Table 14. Selected bond lengths [Å] and angles [º] for 1-Ti.

Ti(1)-O(5)	1.874(8)
Ti(1)-O(9)#1	1.945(8)
Ti(1)-O(17)	2.023(6)
Ti(1)-O(15)#2	2.057(7)
Ti(2)-O(14)	2.215(8)
Ti(2)-O(6)	2.280(6)
Ti(2)-O(10)#1	2.316(6)
Ti(2)-O(2)#3	2.326(8)
Ti(2)-O(8)#4	2.350(6)
Ti(2)-O(16)#2	2.370(9)
Ti(3)-O(7)#4	1.897(8)
Ti(3)-O(13)	1.915(8)
Ti(3)-O(1)#3	1.926(7)
Ti(3)-O(18)	1.970(6)
O(5)-Ti(1)-O(9)#1	112.7(4)
O(5)-Ti(1)-O(17)	101.3(3)
O(9)#1-Ti(1)-O(17)	101.0(3)
O(5)-Ti(1)-O(15)#2	114.4(4)
O(9)#1-Ti(1)-O(15)#2	127.2(4)
O(17)-Ti(1)-O(15)#2	92.1(3)
O(14)-Ti(2)-O(6)	120.1(3)
O(14)-Ti(2)-O(10)#1	86.4(2)
O(6)-Ti(2)-O(10)#1	89.7(2)
O(14)-Ti(2)-O(2)#3	83.7(3)
O(6)-Ti(2)-O(2)#3	151.8(3)
O(10)#1-Ti(2)-O(2)#3	107.9(2)
O(14)-Ti(2)-O(8)#4	91.0(3)
O(6)-Ti(2)-O(8)#4	81.1(2)

O(10)#1-Ti(2)-O(8)#4	167.7(3)
O(2)#3-Ti(2)-O(8)#4	83.8(2)
O(7)#4-Ti(3)-O(13)	112.2(3)
O(7)#4-Ti(3)-O(1)#3	126.8(4)
O(13)-Ti(3)-O(1)#3	110.2(4)
O(7)#4-Ti(3)-O(18)	107.6(3)
O(13)-Ti(3)-O(18)	96.4(4)
O(1)#3-Ti(3)-O(18)	97.9(3)

Symmetry transformations used to generate equivalent atoms:

#1 -x+1,y,-z+1/2 #2 x+1/2,-y+1/2,-z #3 -x+3/2,y+1/2,-z+1/2

#4 x,-y,-z #5 -x+3/2,y-1/2,-z+1/2 #6 x-1/2,-y+1/2,-z

Supplementary Table 15. Selected bond lengths [Å] and angles [º] for Me_2L-Mg.

Mg(1)-O(4)	2.000(8)
Mg(1)-O(12)	2.035(9)
Mg(1)-O(25)	1.935(9)
Mg(1)-O(29)	2.086(9)
Mg(1)-O(45)	2.038(11)
Mg(2)-O(7)	2.019(9)
Mg(2)-O(13)	1.947(9)
Mg(2)-O(20)	1.993(9)
Mg(2)-O(31)	2.070(9)
Mg(2)-O(37)	2.079(10)
Mg(3)-O(2)	2.007(9)
Mg(3)-O(8)	2.127(8)
Mg(3)-O(17)	2.076(8)
Mg(3)-O(19)	2.109(9)
Mg(3)-O(23)	2.001(9)
Mg(3)-O(24)	2.144(9)
O(4)-Mg(1)-O(12)	150.7(4)
O(4)-Mg(1)-O(29)	86.4(3)
O(4)-Mg(1)-O(45)	85.2(4)
O(12)-Mg(1)-O(29)	90.5(4)
O(12)-Mg(1)-O(45)	83.7(4)
O(25)-Mg(1)-O(4)	109.8(4)
O(25)-Mg(1)-O(12)	99.5(4)
O(25)-Mg(1)-O(29)	97.9(4)
O(25)-Mg(1)-O(45)	110.8(6)
O(45)-Mg(1)-O(29)	151.3(6)
O(7)-Mg(2)-O(31)	91.5(3)
O(7)-Mg(2)-O(37)	82.7(4)

O(13)-Mg(2)-O(7)	101.3(4)
O(13)-Mg(2)-O(20)	105.6(4)
O(13)-Mg(2)-O(31)	100.2(4)
O(13)-Mg(2)-O(37)	115.5(4)
O(20)-Mg(2)-O(7)	152.8(4)
O(20)-Mg(2)-O(31)	88.2(3)
O(20)-Mg(2)-O(37)	81.8(4)
O(31)-Mg(2)-O(37)	144.3(4)
O(2)-Mg(3)-O(8)	87.6(3)
O(2)-Mg(3)-O(17)	91.3(3)
O(2)-Mg(3)-O(19)	93.3(3)
O(2)-Mg(3)-O(24)	88.4(3)
O(8)-Mg(3)-O(24)	94.3(3)
O(17)-Mg(3)-O(8)	87.4(3)
O(17)-Mg(3)-O(19)	91.6(3)
O(17)-Mg(3)-O(24)	178.3(4)
O(19)-Mg(3)-O(8)	178.7(4)
O(19)-Mg(3)-O(24)	86.7(3)
O(23)-Mg(3)-O(2)	171.2(4)
O(23)-Mg(3)-O(8)	88.5(3)
O(23)-Mg(3)-O(17)	96.4(4)
O(23)-Mg(3)-O(19)	90.7(3)
O(23)-Mg(3)-O(24)	84.0(3)

Supplementary Table 16. Selected bond lengths [Å] and angles [º] for Me_2L-Mn.

Mn(1)-O(6)	2.095(9)
Mn(1)-O(11)	2.027(11)
Mn(1)-O(27)	2.187(11)
Mn(1)-O(29)	2.070(11)
Mn(1)-O(32)	2.187(13)
Mn(2)-O(8)	2.254(9)
Mn(2)-O(9)	2.113(11)
Mn(2)-O(10)	2.155(9)
Mn(2)-O(19)	2.251(9)
Mn(2)-O(22)	2.079(12)
Mn(2)-O(23)	2.217(10)
Mn(3)-O(4)	2.087(10)
Mn(3)-O(17)	2.206(11)
Mn(3)-O(21)	2.056(11)
Mn(3)-O(24)	2.113(10)
Mn(3)-O(55)	2.160(15)
O(6)-Mn(1)-O(27)	93.3(4)
O(6)-Mn(1)-O(32)	80.0(5)
O(11)-Mn(1)-O(6)	101.6(4)
O(11)-Mn(1)-O(27)	98.3(4)
O(11)-Mn(1)-O(29)	108.8(5)
O(11)-Mn(1)-O(32)	117.1(6)
O(29)-Mn(1)-O(6)	149.1(5)
O(29)-Mn(1)-O(27)	87.3(4)
O(29)-Mn(1)-O(32)	81.8(5)
O(32)-Mn(1)-O(27)	144.7(5)
O(9)-Mn(2)-O(8)	86.1(4)
O(9)-Mn(2)-O(10)	91.7(4)

O(9)-Mn(2)-O(19)	86.2(4)
O(9)-Mn(2)-O(23)	93.3(4)
O(10)-Mn(2)-O(8)	177.7(5)
O(10)-Mn(2)-O(19)	85.5(4)
O(10)-Mn(2)-O(23)	95.1(4)
O(19)-Mn(2)-O(8)	94.2(4)
O(22)-Mn(2)-O(8)	84.5(4)
O(22)-Mn(2)-O(9)	168.5(4)
O(22)-Mn(2)-O(10)	97.7(4)
O(22)-Mn(2)-O(19)	87.8(4)
O(22)-Mn(2)-O(23)	92.6(4)
O(23)-Mn(2)-O(8)	85.1(4)
O(23)-Mn(2)-O(19)	179.2(4)
O(4)-Mn(3)-O(17)	86.2(4)
O(4)-Mn(3)-O(24)	144.6(4)
O(4)-Mn(3)-O(55)	84.6(5)
O(21)-Mn(3)-O(4)	113.8(4)
O(21)-Mn(3)-O(17)	94.9(4)
O(21)-Mn(3)-O(24)	101.6(4)
O(21)-Mn(3)-O(55)	102.4(6)
O(24)-Mn(3)-O(17)	91.3(4)
O(24)-Mn(3)-O(55)	87.6(6)
O(55)-Mn(3)-O(17)	162.6(6)

(*S*)-1-Phenyl-but-3-en-1-ol:

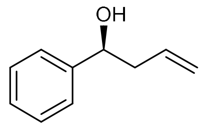

Following the general procedure for the allylation of aldehydes, the title compound was obtained in 99 % yield with spectral properties reported in literature. Enantiomeric excess was determined by HPLC with a chiralcel OD-H column: hexane/iPrOH = 90/10; flow rate = 0.5 mL/min; t_{minor} = 11.27 min, t_{major} = 11.85 min; ee = 83%. ^{1}H NMR (400 MHz, $CDCl_3$) δ: 2.05 (br s, 1H), 2.49-2.54 (m, 2H), 4.72-4.74 (m,1H), 4.74-5.19 (m, 2H), 5.76-5.87 (m, 1H), 7.26-7.36 (m, 5H).

a

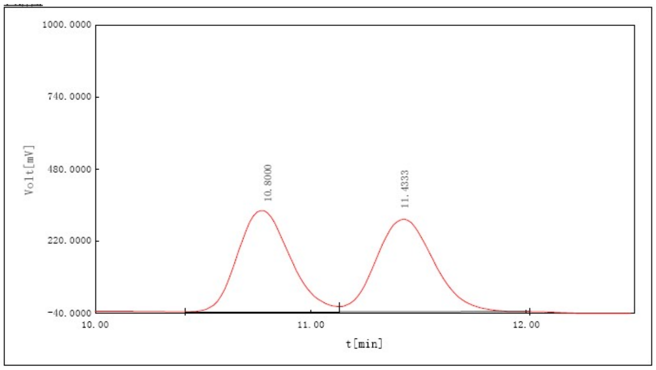

b

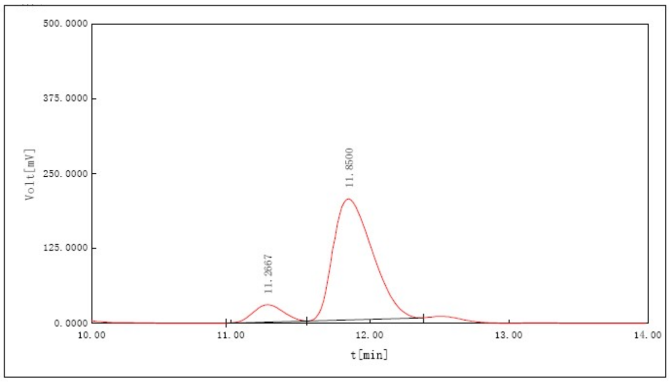

c

Serial Number	Retention Time[min]	Area[mAbs*s]	Type	Area%
1	11.2667	372.3930	BB	8.4400
2	11.8500	4039.8469	BB	91.5600
The Total		4412.2399		

Supplementary Figure 63. HPLC spectra for (*S*)-1-Phenyl-but-3-en-1-ol. a, Racemic standard. **b**, After reaction. **c**, The detail of integration

(*S*)-1-(4-Chloro-phenyl)-but-3-en-1-ol:

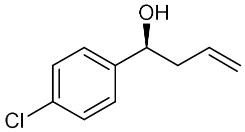

Following the general procedure for the allylation of aldehydes, the title compound was obtained in 99 % yield with spectral properties reported in literature. Enantiomeric excess was determined by HPLC with a chiralcel AD-H column: hexane/iPrOH = 98/2; flow rate = 1.0 mL/min; t_{minor} = 15.88 min, t_{major} = 16.80 min; ee = 92%. ^{1}H NMR (400 MHz, $CDCl_3$) δ: 2.07 (br s, 1H), 2.45-2.51 (m, 2H), 4.70-4.73 (m. 1H), 5.14-5.18 (m, 2H), 5.72-5.83 (m, 1H), 7.28-7.33 (m, 4H).

a

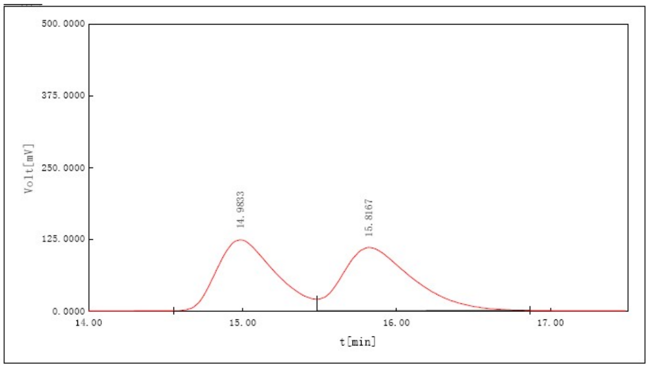

b

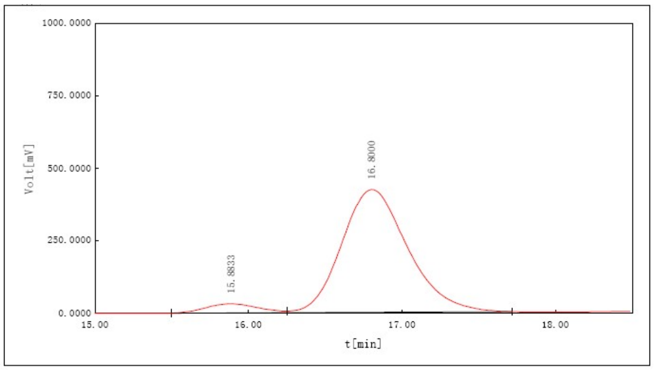

c

Serial Number	Retention Time[min]	Area[mAbs*s]	Type	Area%
1	15.8833	565.1163	BB	4.2121
2	16.8000	12851.3808	BB	95.7879
The Total		13416.4971		

Supplementary Figure 64. HPLC spectra for (*S*)-1-(4-Chloro-phenyl)-but-3-en-1-ol. **a**, Racemic standard. **b**, After reaction. **c**, The detail of integration

(*S*)-1-(4-Bromo-phenyl)-but-3-en-1-ol:

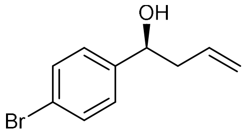

Following the general procedure for the allylation of aldehydes, the title compound was obtained in 99 % yield with spectral properties reported in literature. Enantiomeric excess was determined by HPLC with a chiralcel OJ-H column: hexane/iPrOH = 95/5; flow rate = 0.4 mL/min; t_{major} = 25.50 min, t_{minor} = 28.00 min; ee = 90%. ^{1}H NMR (400 MHz, $CDCl_3$) δ: 2.07 (br s, 1H), 2.44-2.50 (m, 2H), 4.69-4.72 (m, 1H), 5.14-5.18 (m, 2H), 5.72-5.83 (m, 1H), 7.23-7.48 (m, 4H).

a

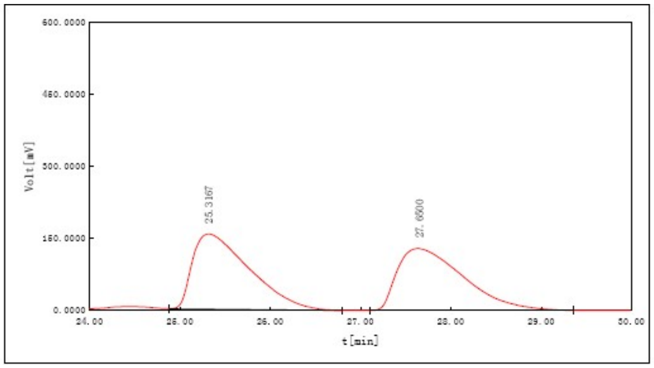

b

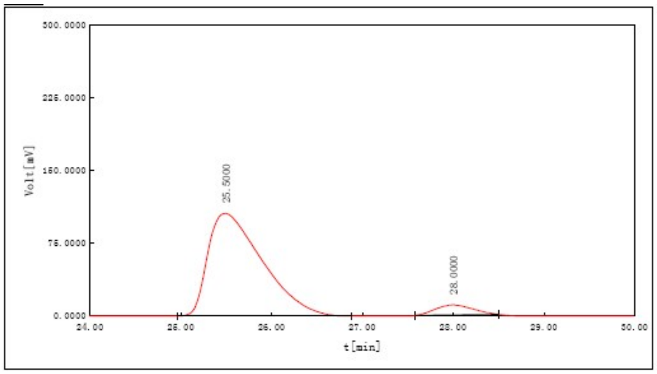

c

Serial Number	Retention Time[min]	Area[mAbs*s]	Type	Area%
1	25.5000	4489.2434	BB	94.7772
2	28.0000	247.3846	BB	5.2228
The Total		4736.6280		

Supplementary Figure 65. HPLC spectra for (*S*)-1-(4-Bromo-phenyl)-but-3-en-1-ol a, Racemic standard. **b**, After reaction. **c**, The detail of integration

(*S*)-1-(4-Nitro-phenyl)-but-3-en-1-ol:

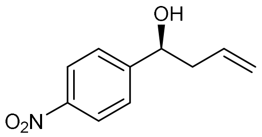

Following the general procedure for the allylation of aldehydes, the title compound was obtained in 98% yield with spectral properties reported in literature. Enantiomeric excess was determined by HPLC with a chiralcel AS-H column: hexane/iPrOH = 97/3; flow rate = 0.5 mL/min; t_{minor} = 72.63 min, t_{major} = 77.30 min; ee = 96%. ^{1}H NMR (400 MHz, $CDCl_3$) δ: 2.23 (br s, 1H), 2.41-2.58 (m, 2H), 4.84 (m, 1H), 4.85-5.21 (m, 2H), 5.73-5.84 (m, 1H), 7.53 (d, *J* = 8.8 Hz, 2H), 8.20 (d, *J* = 8.8 Hz, 2H).

a

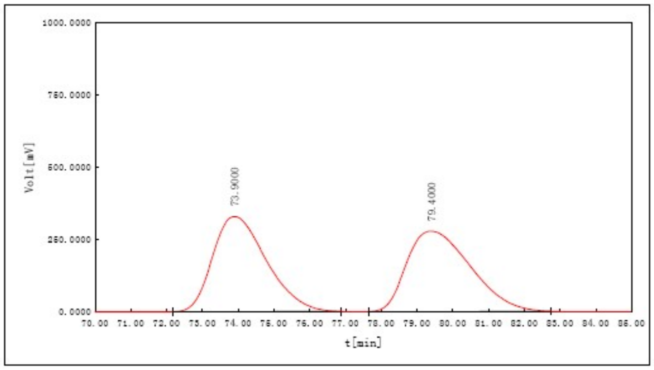

b

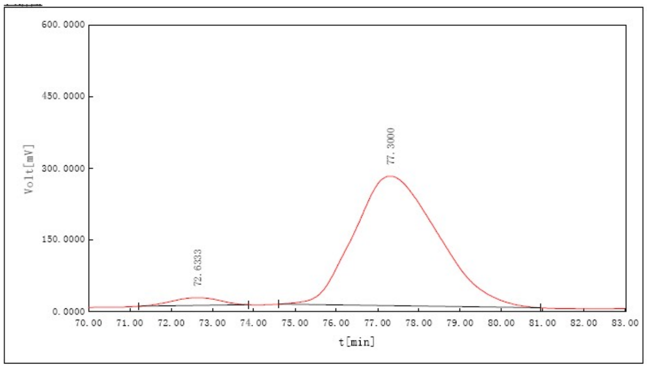

c

Serial Number	Retention Time[min]	Area[mAbs*s]	Type	Area%
1	72.6333	877.2764	BB	2.2327
2	77.3000	38414.9004	BB	97.7673
The Total		39292.1768		

Supplementary Figure 66. HPLC spectra for (*S*)-1-(4-Nitro-phenyl)-but-3-en-1-ol
a, Racemic standard. **b**, After reaction. **c**, The detail of integration.

(*S*)-1-(4-Methyl-phenyl)-but-3-en-1-ol:

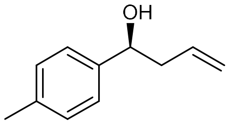

Following the general procedure for the allylation of aldehydes, the title compound was obtained in 98% yield with spectral properties reported in literature. Enantiomeric excess was determined by HPLC with a chiralcel OD-H column: hexane/iPrOH = 99/1; flow rate = 1.0 mL/min; t_{minor} = 19.95 min, t_{major} = 21.35 min; ee = 84%. ^{1}H NMR (400 MHz, $CDCl_3$) δ: 2.00 (br s, 1H), 2.35 (s, 3H), 2.50-2.52 (m, 2H), 4.70 (m, 1H), 5.12-5.19 (m, 2H), 5.76-5.86 (m, 1H), 7.16 (d, *J* = 8.8 Hz, 2H), 7.25 (d, *J* = 8.8 Hz, 2H).

a

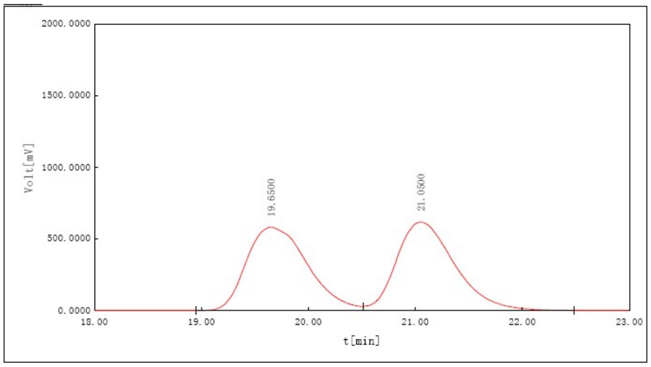

b

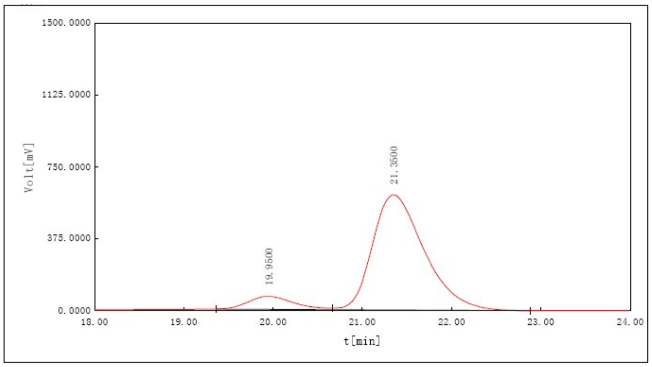

c

Serial Number	Retention Time[min]	Area[mAbs*s]	Type	Area%
1	19.9500	2192.7060	BB	8.2506
2	21.3500	24383.6154	BB	91.7494
The Total		26576.3217		

Supplementary Figure 67. HPLC spectra for (*S*)-1-(4-Methyl-phenyl)-but-3-en-1-ol a, Racemic standard. **b**, After reaction. **c**, The detail of integration.

(*S*)-1-(*o*-methyl-phenyl)-but-3-en-1-ol:

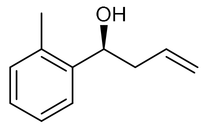

Following the general procedure for the allylation of aldehydes, the title compound was obtained in 97% yield with spectral properties reported in literature. Enantiomeric excess was determined by HPLC with a chiralcel AD-H column: hexane/iPrOH = 95/5; flow rate = 0.5 mL/min; t_{minor} = 13.98 min, t_{major} = 16.37min; ee = 86%. ^{1}H NMR (400 MHz, $CDCl_3$) δ: 2.00 (br s, 1H), 2.34 (s, 3H), 2.35-2.55 (m, 2H), 4.98 (dd, 1H), 5.15-5.21 (m, 2H), 5.81-5.92 (m, 1H), 7.13-7.50 (m, 4H).

a

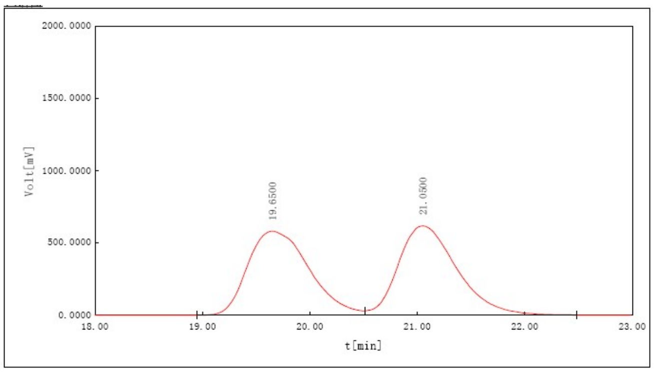

b

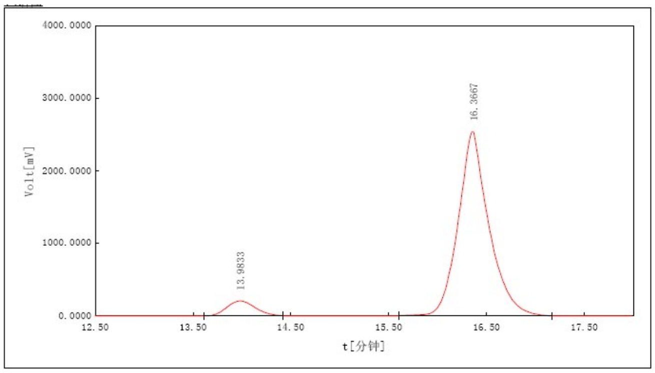

c

Serial Number	Retention Time[min]	Area[mAbs*s]	Type	Area%
1	13.9833	4192.3245	BB	7.0663
2	16.3667	55135.7892	BB	92.9337
The Total		59328.1137		

Supplementary Figure 68. HPLC spectra for (*S*)-1-(*o*-methyl-phenyl)-but-3-en-1-ol. a, Racemic standard. **b**, After reaction. **c**, The detail of integration

(*S*)-1-(2-Nitro-phenyl)-but-3-en-1-ol:

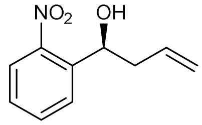

Following the general procedure for the allylation of aldehydes, the title compound was obtained in 97% yield with spectral propertiesreported in literature. Enantiomeric excess was determined by HPLC with a chiralcel OD-H column: hexane/iPrOH = 99/1; flow rate = 0.5 mL/min; t_{minor} = 57.53 min, t_{minor} = 62.15 min; ee = 98%. ^{1}H NMR (400 MHz, $CDCl_3$) δ: 7.95 (d, 2H), 7.85 (d, 2H), 7.65-7.69 (t, 1H), 7.43-7.47 (t, 1H), 5.87-5.97 (m, 1H), 5.33-5.36 (dd, 1H), 2.21-2.25 (m, 2H), 2.70-2.76 (m,1H), 2.49-2.41 (m, 2H).

a

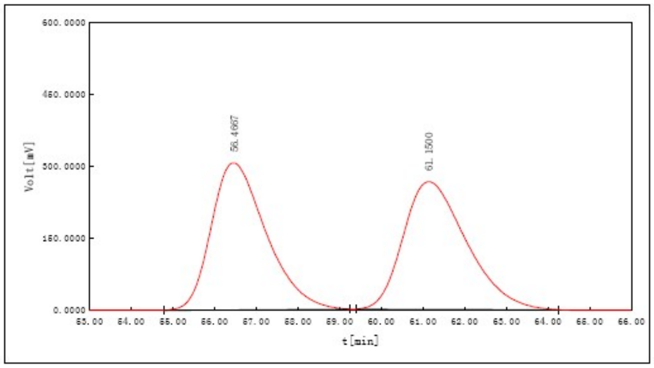

b

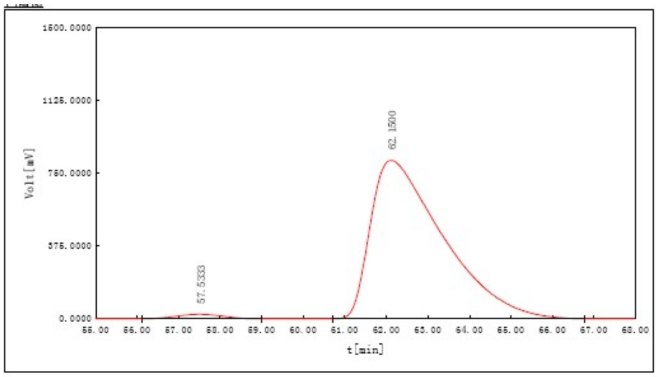

c

Serial Number	Retention Time[min]	Area[mAbs*s]	Type	Area%
1	57.5333	1253.2147	BB	1.2419
2	62.1500	99657.8631	BB	98.7581
The Total		100911.0778		

Supplementary Figure 69. HPLC spectra for (*S*)-1-(2-Nitro-phenyl)-but-3-en-1-ol
a, Racemic standard. **b**, After reaction. **c**, The detail of integration

(*R*)-1-Thiophen-2-yl-but-3-en-1-ol:

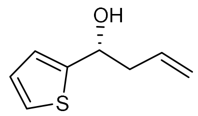

Following the general procedure for the allylation of aldehydes, the title compound was obtained in 93% yield with spectral properties reported in literature. Enantiomeric excess was determined by HPLC with a chiralcel OJ-H column: hexane/iPrOH = 93/7; flow rate = 0.5 mL/min; t_{major} = 16.30 min, t_{minor} = 18.57 min; ee = 88 %. ^{1}H NMR (400 MHz, $CDCl_3$) δ: 2.18-2.19 (m, 1H), 2.61-2.64 (m, 2H), 4.97-5.00 (m, 1H), 5.15-5.22 (m, 2H), 5.78-5.88 (m, 1H), 6.96-6.99 (m, 2H), 7.24-7.26 (m, 1H).

a

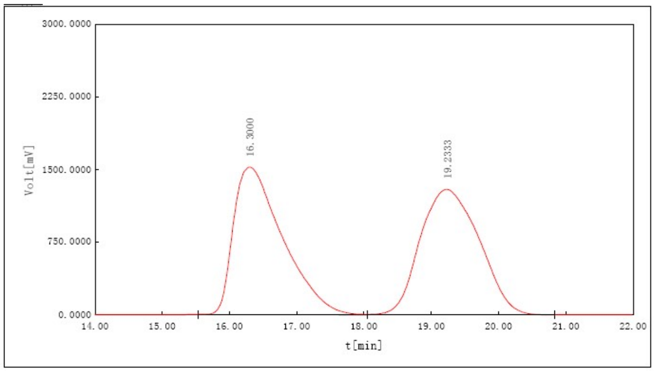

b

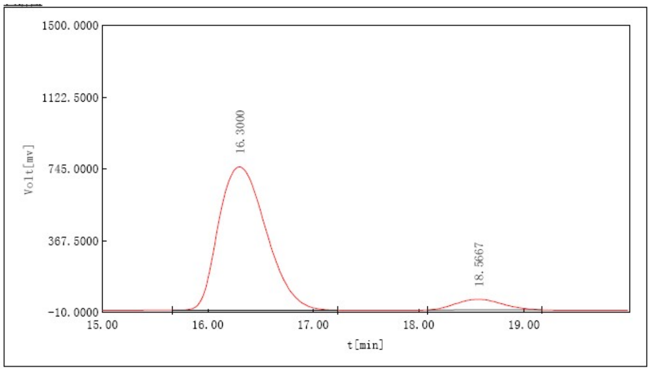

c

Serial Number	Retention Time[min]	Area[mAbs*s]	Type	Area%
1	16.3000	23767.3160	BB	93.7585
2	18.5667	1582.2297	BB	6.2415
The Total		25350.1517		

Supplementary Figure 70. HPLC spectra for (*R*)-1-Thiophen-2-yl-but-3-en-1-ol. **a**, Racemic standard. **b**, After reaction. **c**, The detail of integration.

1-(3,5-bis(benzyloxy)phenyl)but-3-en-1-ol:

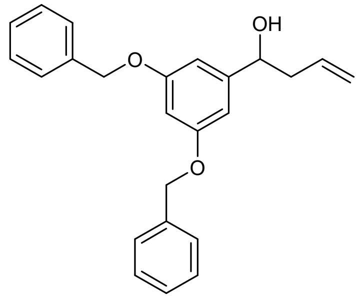

Following the general procedure for the allylation of aldehydes, the title compound was obtained in 93% yield with spectral properties reported in literature. ^{1}H NMR (400 MHz, $CDCl_3$) δ: 7.48-7.29 (m, 10H), 6.63 (d, J = 2.3 Hz, 2H), 6.55 (t, J = 2.3 Hz, 1H), 5.89-5.70 (m, 1H), 5.20-5.11 (m, 2H), 5.04 (s, 4H), 4.67 (dd, J = 7.6, 5.1 Hz, 1H), 2.58-2.39 (m, 2H), 1.92 (br s, 1H). ^{13}C NMR (400 MHz, $CDCl_3$) δ: 160.04, 146.54, 136.87, 134.37, 128.61, 128.02, 127.59, 118.49, 104.97, 101.15, 73.29, 70.12, 43.71. HRMS-ESI (m/z): Calculated for $C_{24}H_{25}O_3$ $(M+H)^+$: 361.1804, Found: 361.1797.

(*R*)-1-Phenyl-but-3-yn-1-ol:

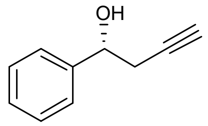

Following the general procedure for the propargylation of aldehydes, the title compound was obtained in 92% yield with spectral properties reported in literature. Enantiomeric excess was determined by HPLC with a chiralcel OD-H column: hexane/iPrOH = 95/5; flow rate = 1.0 mL/min; t_{major} = 12.07 min, t_{minor} = 14.70 min; ee = 92%. ^{1}H NMR (400 MHz, $CDCl_3$) δ: 7.41-7.29 (m, 5H), 4.88 (t, *J* = 6.8 Hz, 1H), 2.66-2.63 (m, 2H), 2.37 (br.s., 1H), 2.08 (t, *J* = 2.4 Hz, 1H).

a

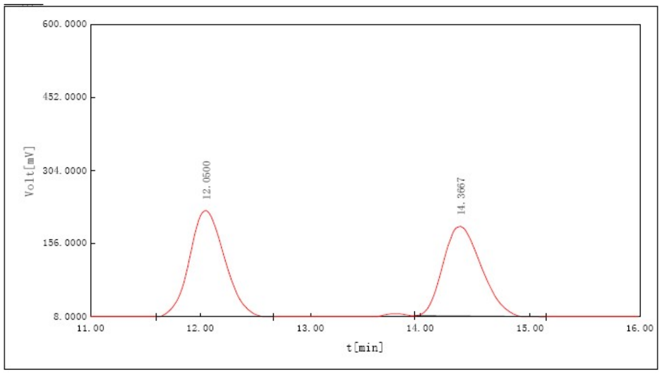

b

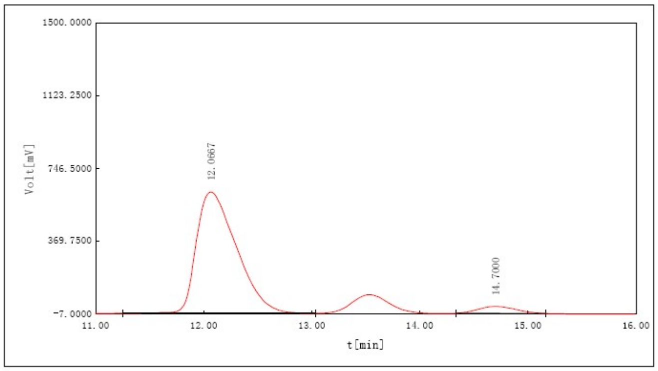

c

Serial Number	Retention Time[min]	Area[mAbs*s]	Type	Area%
1	12.0667	16260.8175	BB	95.7549
2	14.7000	720.8904	BB	4.2451
The Total		16981.7080		

Supplementary Figure 71. HPLC spectra for (*R*)-1-Phenyl-but-3-yn-1-ol. a, Racemic standard. **b**, After reaction. **c**, The detail of integration.

(*R*)-1-(4-Chloro-phenyl)-but-3-yn-1-ol:

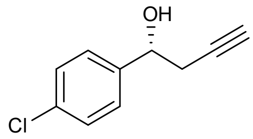

Following the general procedure for the propargylation of aldehydes, the title compound was obtained in 93% yield with spectral properties reported in literature. Enantiomeric excess was determined by HPLC with a chiralcel OD-H column: hexane/iPrOH = 98/2; flow rate = 1.0 mL/min; t_{major} = 20.38 min, t_{minor} = 22.58 min; ee = 92%. ^{1}H NMR (400 MHz, $CDCl_3$) δ: 7.33 (s, 4H), 4.85 (t, *J* = 6.4 Hz 1H), 2.62-2.60 (m, 2H), 2.46 (br.s., 1H), 2.08 (t, *J* = 2.8 Hz, 1H).

a

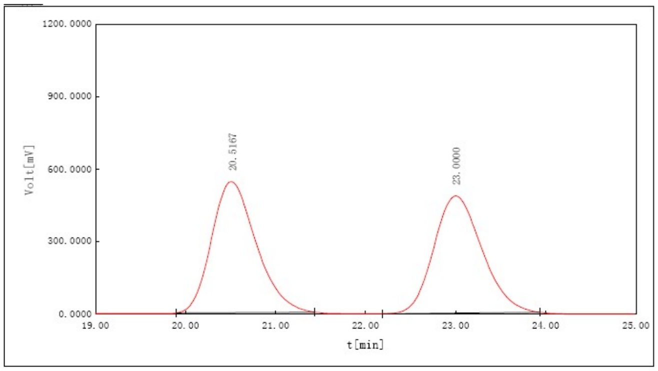

b

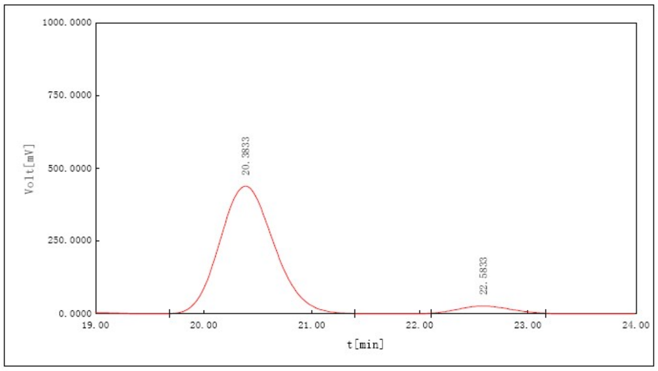

c

Serial Number	Retention Time[min]	Area[mAbs*s]	Type	Area%
1	20.3833	15428.8393	BB	95.7556
2	22.5833	683.8899	BB	4.2544
The Total		16112.7279		

Supplementary Figure 72. HPLC spectra for (*R*)-1-(4-Chloro-phenyl)-but-3-yn-1-ol. a, Racemic standard. **b**, After reaction. **c**, The detail of integration.

(*R*)-1-(4-Bromo-phenyl)-but-3-yn-1-ol:

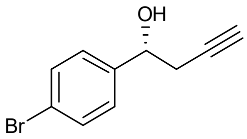

Following the general procedure for the propargylation of aldehydes, the title compound was obtained in 91% yield with spectral properties reported in literature. Enantiomeric excess was determined by HPLC with a chiralcel OD-H column: hexane/iPrOH = 98/2; flow rate = 1.0 mL/min; t_{major} = 23.67 min, t_{minor} = 26.28 min; ee = 92%. ^{1}H NMR (400 MHz, $CDCl_3$) δ: 7.49-7.47 (m, 2H), 7.28-7.26 (m, 2H), 4.85-4.82 (m, 1H), 2.62-2.59 (m, 2H), 2.46 (br.s., 1H), 2.09-2.07 (m, 1H).

a

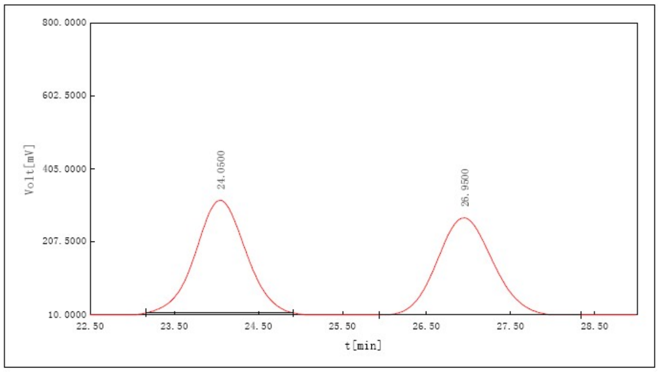

b

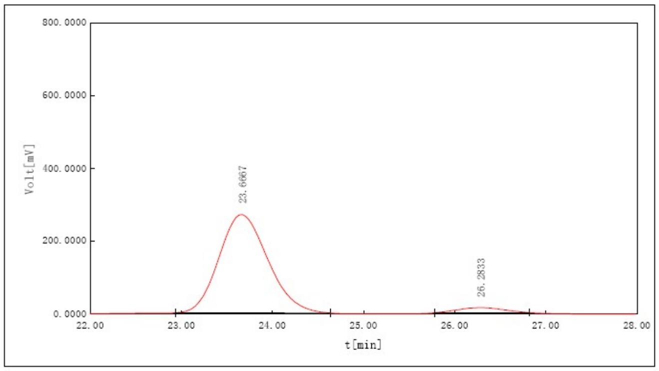

c

Serial Number	Retention Time[min]	Area[mAbs*s]	Type	Area%
1	23.6667	10113.1791	BB	95.9430
2	26.2833	427.6411	BB	4.0570
The Total		10540.8202		

Supplementary Figure 73. HPLC spectra for (*R*)-1-(4-Bromo-phenyl)-but-3-yn-1-ol. **a**, Racemic standard. **b**, After reaction. **c**, The detail of integration.

(*R*)-1-(4-Nitro-phenyl)-but-3-yn-1-ol:

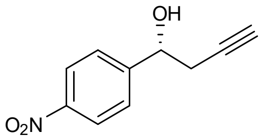

Following the general procedure for the propargylation of aldehydes, the title compound was obtained in 94% yield with spectral properties reported in literature. Enantiomeric excess was determined by HPLC with a chiralcel OJ-H column: hexane/iPrOH = 90/10; flow rate = 1.0 mL/min; t_{minor} = 41.08 min, t_{major} = 47.18 min; ee = 91%. ^{1}H NMR (400 MHz, $CDCl_3$) δ: 8.24-8.21 (m, 2H), 7.59-7.57 (m, 2H), 5.02-4.98 (m, 1H), 2.73-2.56 (m, 3H), 2.11 (d, *J* = 2.8 Hz, 1H).

a

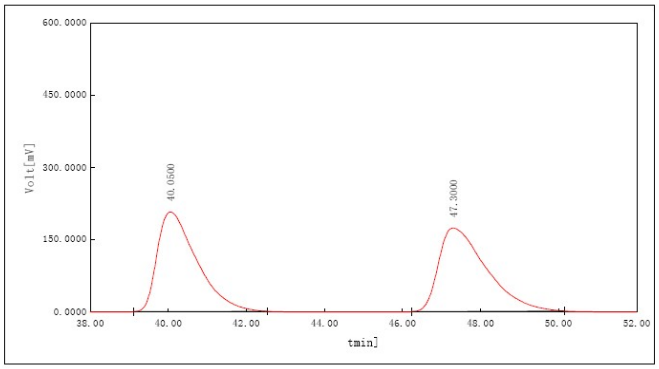

b

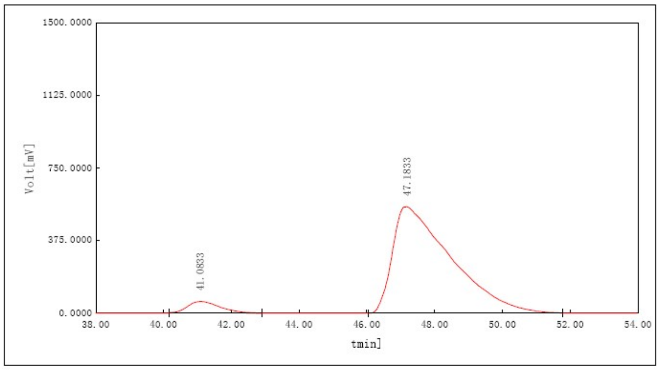

c

Serial Number	Retention Time[min]	Area[mAbs*s]	Type	Area%
1	41.0833	3639.5838	BB	4.2462
2	47.1833	65736.0373	BB	94.7538
The Total		69375.6211		

Supplementary Figure 74. HPLC spectra for (*R*)-1-(4-Nitro-phenyl)-but-3-yn-1-ol. a, Racemic standard. **b**, After reaction. **c**, The detail of integration.

(*R*)-Methyl 4-(1-hydroxybut-3-yn-1-yl)benzoate:

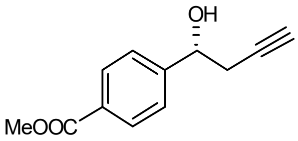

Following the general procedure for the propargylation of aldehydes, the title compound was obtained in 92% yield with spectral properties reported in literature. Enantiomeric excess was determined by HPLC with a chiralcel OD-H column: hexane/iPrOH = 90/10; flow rate =1.0 mL/min; t_{major} = 12.83 min, t_{minor} = 17.47 min; ee = 94%. ^{1}H NMR (400 MHz, $CDCl_3$) δ: 8.01 (d, J = 8.8 Hz, 2H), 7.46 (d, J = 8.4 Hz, 2H), 4.95-4.91 (m, 1H), 3.90 (s, 3H), 2.66-2.59 (m, 3H), 2.08 (d, J = 2.8 Hz, 1H).

a

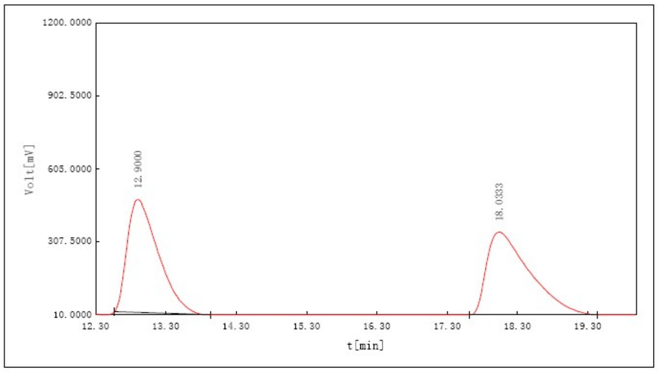

b

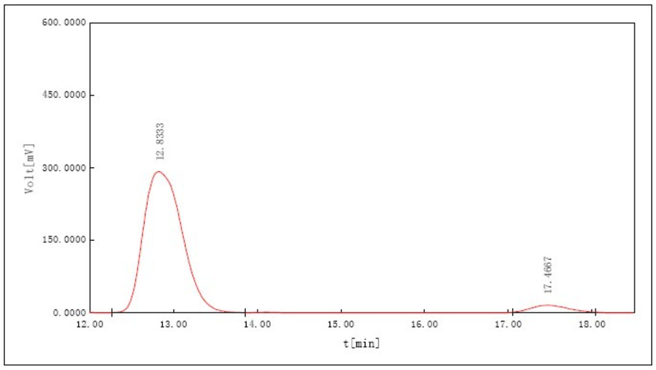

c

Serial Number	Retention Time[min]	Area[mAbs*s]	Type	Area%
1	12.8333	9462.9816	BB	96.7058
2	17.4667	322.3483	BB	3.2942
The Total		9785.3299		

Supplementary Figure 75. HPLC spectra for (*R*)-Methyl 4-(1-hydroxybut-3-yn-1-yl)benzoate. **a**, Racemic standard. **b**, After reaction. **c**, The detail of integration.

(*R*)-1-(4-Methoxy-phenyl)-but-3-yn-1-ol:

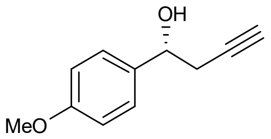

Following the general procedure for the propargylation of aldehydes, the title compound was obtained in 85% yield with spectral properties reported in literature. Enantiomeric excess was determined by HPLC with a chiralcel OD-H column: hexane/iPrOH = 95/5; flow rate = 1.0 mL/min; t_{major} = 12.7 min, t_{minor} = 23.1 min; ee = 96%. ^{1}H NMR (400 MHz, $CDCl_3$) δ: 7.33-7.31 (m, 2H), 6.90-6.88 (m, 2H), 4.84 (t, *J* = 6.4 Hz, 1H), 3.80 (s, 3H), 2.64-2.61 (m, 2H), 2.30 (br.s., 1H), 2.07 (t, *J* = 2.8 Hz, 1H).

a

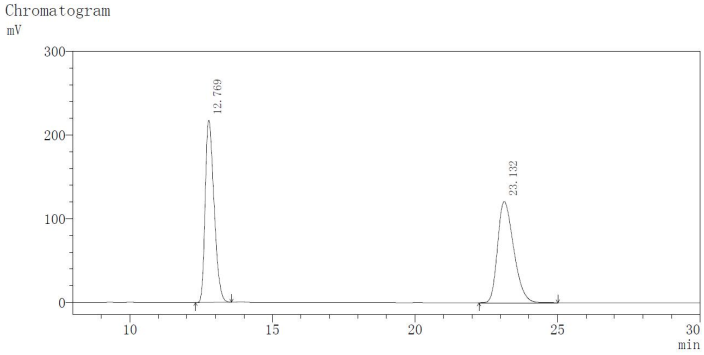

b

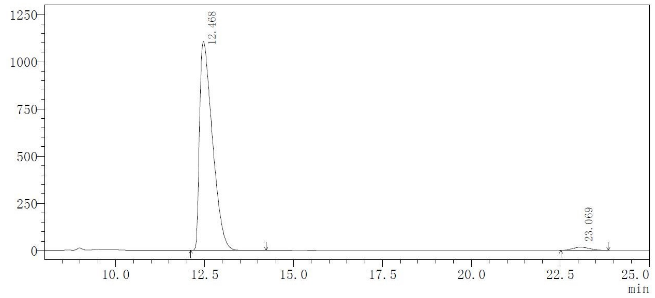

c

Serial Number	Retention Time[min]	Area[mAbs*s]	Type	Area%
1	12.468	27800792	BB	97.934
2	23.069	586549	BB	2.066
The Total		28387340		

Supplementary Figure 76. HPLC spectra for (*R*)-1-(4-Methoxy-phenyl)-but-3-yn-1-ol. a, Racemic standard. **b**, After reaction. **c**, The detail of integration.

(*R*)-1-(3-Methoxy-phenyl)-but-3-yn-1-ol:

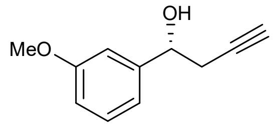

The product was obtained in 90% yield with spectral properties reported in literature. Enantiomeric excess was determined by HPLC with a chiralcel AD-H column: hexane/iPrOH = 98/2; flow rate = 1.0 mL/min; t_{major} = 27.60 min, t_{minor} = 30.48 min; ee = 99%. ^{1}H NMR (400 MHz, $CDCl_3$) δ: 7.30-7.26 (m, 1H), 6.97-6.95 (m, 2H), 6.83 (ddd, J = 7.6, 2.8, 1.2 Hz, 1H), 4.86 (d, J = 6.4 Hz, 1H), 3.81 (s, 3H), 2.65-2.62 (m, 2H), 2.42 (br.s., 1H), 2.09 (t, J = 2.8 Hz, 1H).

a

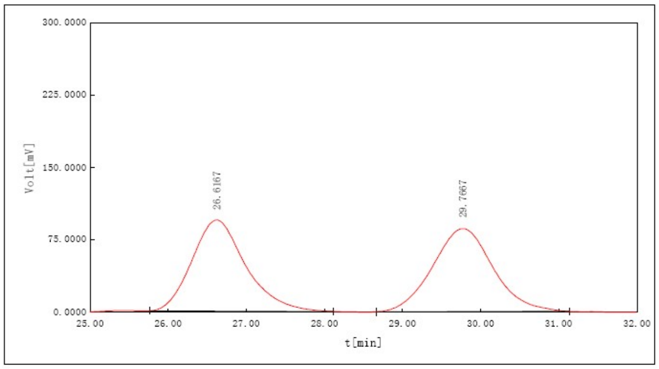

b

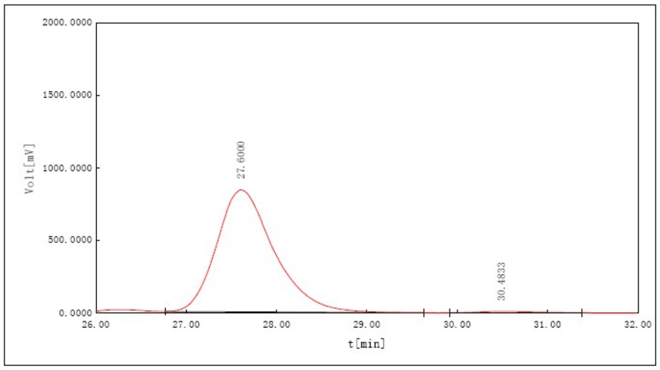

c

Serial Number	Retention Time[min]	Area[mAbs*s]	Type	Area%
1	27.6000	37073.3921	BB	99.5194
2	30.4833	405.0096	BB	0.4806
The Total		37478.4016		

Supplementary Figure 77. HPLC spectra for (*R*)-1-(3-Methoxy-phenyl)-but-3-yn-1-ol. a, Racemic standard. **b**, After reaction. **c**, The detail of integration.

(*R*)-1-(Naphthalen-1-yl)but-3-yn-1-ol:

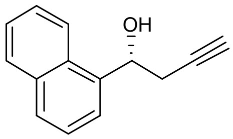

Following the general procedure for the propargylation of aldehydes, the title compound was obtained in 92% yield with spectral properties reported in literature. Enantiomeric excess was determined by HPLC with a chiralcel OJ-H column: hexane/iPrOH = 90/10; flow rate = 1.0 mL/min; t_{minor} = 24.50 min, t_{major} = 31.85 min; ee = 93%. ^{1}H NMR (400 MHz, $CDCl_3$) δ: 8.07 (d, *J* = 8.4 Hz, 1H), 7.90-7.80 (m, 2H), 7.72 (d, *J* = 7.2 Hz, 1H), 7.56-7.48 (m, 3H), 5.66 (dd, *J* = 4.4 Hz, 1H), 2.93-2.87 (m, 1H), 2.79-2.72 (m, 1H), 2.14 (t, *J* = 3.2 Hz, 1H).

a

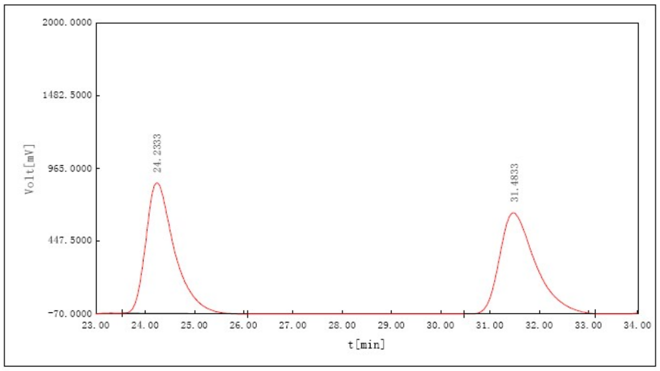

b

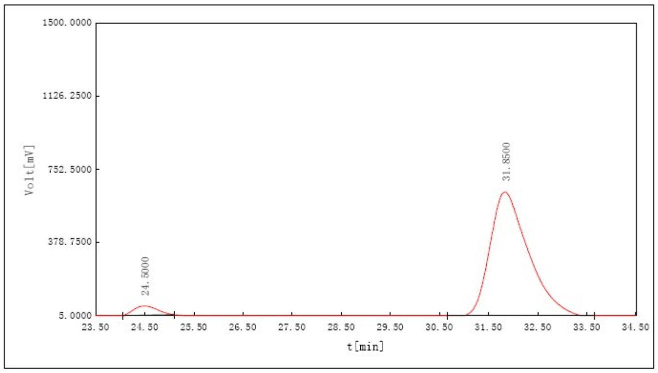

c

Serial Number	Retention Time[min]	Area[mAbs*s]	Type	Area%
1	24.5000	1260.2184	BB	3.3727
2	31.8500	36105.0486	BB	96.6273
The Total		37365.2670		

Supplementary Figure 78. HPLC spectra for (*R*)-1-(Naphthalen-1-yl)but-3-yn-1-ol. a, Racemic standard. **b**, After reaction. **c**, The detail of integration.

(*R*, *E*)-1-phenylhex-1-en-5-yn-3-ol:

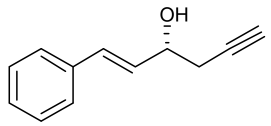

Following the general procedure for the propargylation of aldehydes, the title compound was obtained in 90% yield with spectral properties reported in literature. Enantiomeric excess was determined by HPLC with a chiralcel OD-H column: hexane/iPrOH = 95/5; flow rate = 0.8 mL/min; t_{major} = 25.07 min, t_{minor} = 34.83 min; ee = 97%. ^{1}H NMR (400 MHz, $CDCl_3$) δ: 7.41-7.24 (m, 5 H), 6.66 (d, *J* = 16.4 Hz, 1 H), 6.28(dd, *J* = 16.00 Hz, 6.4 Hz, 1 H), 4.48 (d, *J* = 6.4 Hz, 1 H), 2.63-2.50 (m, 2 H), 2.17 (br.s., 1 H), 2.10 (t, *J* = 2.4 Hz, 1 H).

a

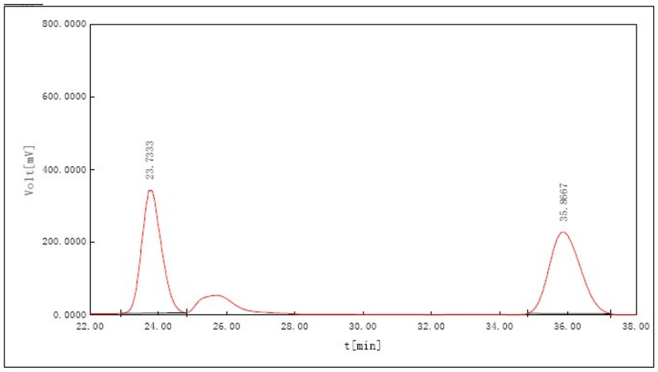

b

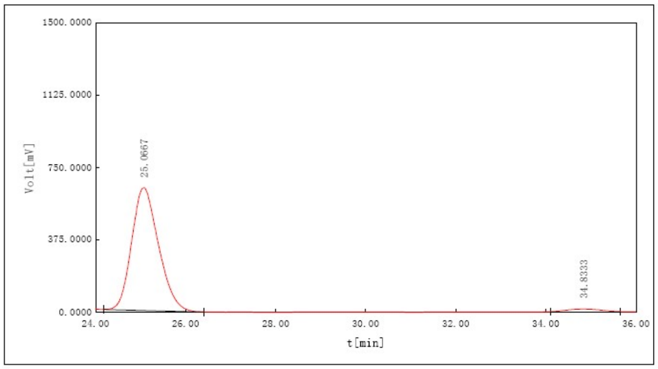

c

Serial Number	Retention Time[min]	Area[mAbs*s]	Type	Area%
1	25.0667	26637.5978	BB	98.5565
2	34.8333	384.1455	BB	1.4445
The Total		27021.7433		

Supplementary Figure 79. HPLC spectra for (*R*, *E*)-1-phenylhex-1-en-5-yn-3-ol.
a, Racemic standard. **b**, After reaction. **c**, The detail of integration.

1-(3, 5-bis(benzyloxy)phenyl)but-3-yn-1-ol:

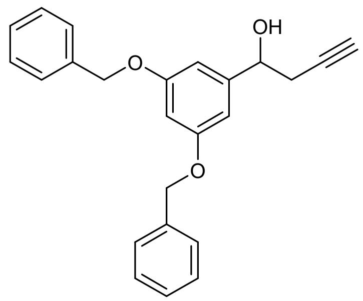

Following the general procedure for the allylation of aldehydes, the title compound was obtained in 93% yield with spectral properties reported in literature. ^{1}H NMR (400 MHz, $CDCl_3$) δ: 7.45-7.30 (m, 10H), 6.66 (d, J = 2.3 Hz, 2H), 6.56 (t, J = 2.2 Hz, 1H), 5.04 (s, 4H), 4.83-4.78 (m, 1H), 2.66-2.59 (m, 2H), 2.07-2.05 (m, 1H). ^{13}C NMR (400 MHz, $CDCl_3$) δ: 160.04, 145.03, 136.78, 128.61, 128.03, 127.56, 104.89, 101.55, 80.61, 72.31, 71.09, 70.12, 29.40. HRMS-ESI (m/z): Calculated for $C_{24}H_{23}O_3$ (M + H)$^+$: 359.1647, Found: 359.1632.

Recycling 1-Cr in propargylation of 3-methoxybenzaldehde:

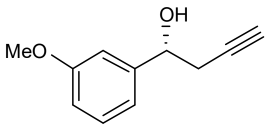

Following the general procedure for the propargylation of aldehydes, enantiomeric excess was determined by HPLC with a chiralcel AD-H column: hexane/iPrOH = 98/2; flow rate = 1.0 mL/min.

a

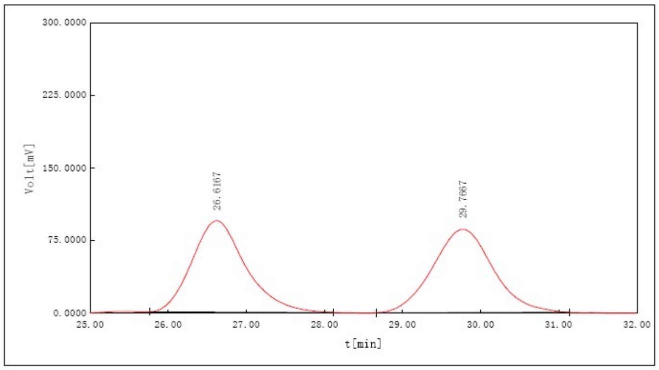

b

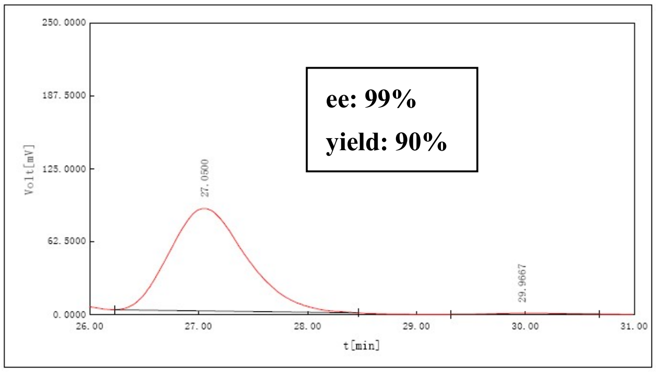

c

Serial Number	Retention Time[min]	Area[mAbs*s]	Type	Area%
1	27.0500	4333.1531	BB	99.5221
2	29.9667	20.8076	BB	0.4779
The Total		4353.9607		

Supplementary Figure 80. HPLC spectra for the second run. a, Racemic standard. **b**, After reaction. **c**, The detail of integration.

a

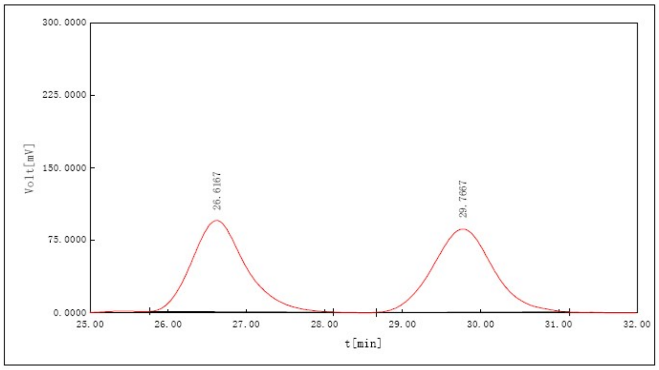

b

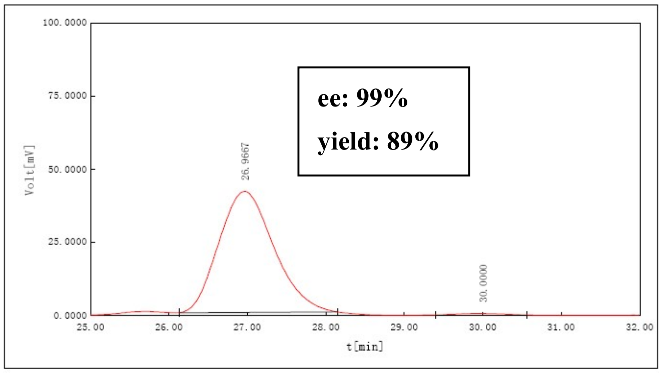

c

Serial Number	Retention Time[min]	Area[mAbs*s]	Type	Area%
1	26.9667	2027.1225	BB	99.5094
2	30.0000	9.9941	BB	0.4906
The Total		2037.1166		

Supplementary Figure 81. HPLC spectra for the third run. a, Racemic standard. **b,** After reaction. **c**, The detail of integration.

a

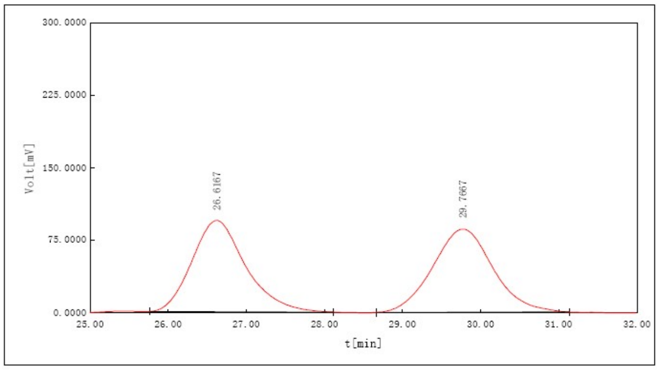

b

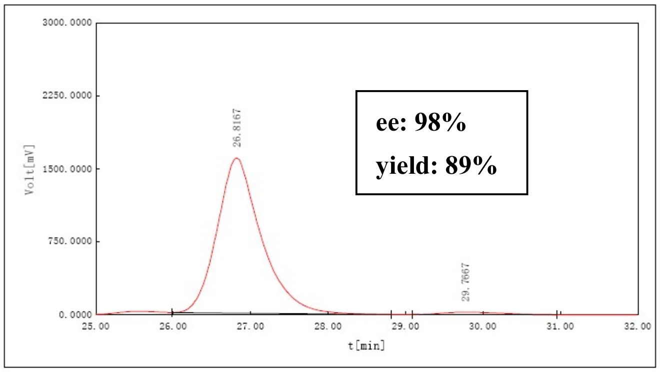

c

Serial Number	Retention Time[min]	Area[mAbs*s]	Type	Area%
1	26.8167	63778.0600	BB	98.8226
2	29.7667	759.8696	BB	1.1774
The Total		64537.9296		

Supplementary Figure 82. HPLC spectra for the fourth run. a, Racemic standard. **b**, After reaction. **c**, The detail of integration.

Recycling 1-Cr in propargylation of 4-methoxybenzaldehde:

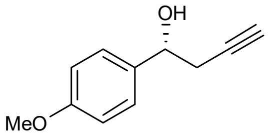

Following the general procedure for the propargylation of aldehydes, enantiomeric excess was determined by HPLC with a chiralcel OD-H column: hexane/iPrOH = 95/5; flow rate = 1.0 mL/min.

a

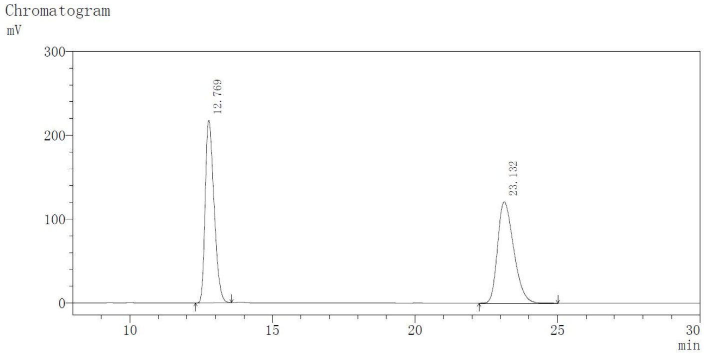

b

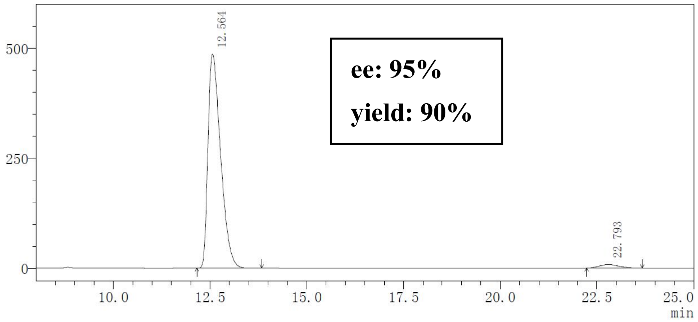

c

Serial Number	Retention Time[min]	Area[mAbs*s]	Type	Area%
1	12.564	11049258	BB	97.454
2	22.793	288716	BB	2.546
The Total		11337973		

Supplementary Figure 83. HPLC spectra for the second run. a, Racemic standard. **b**, After reaction. **c**, The detail of integration.

a

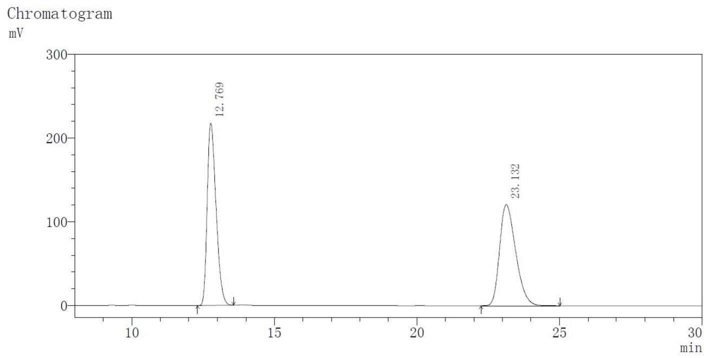

b

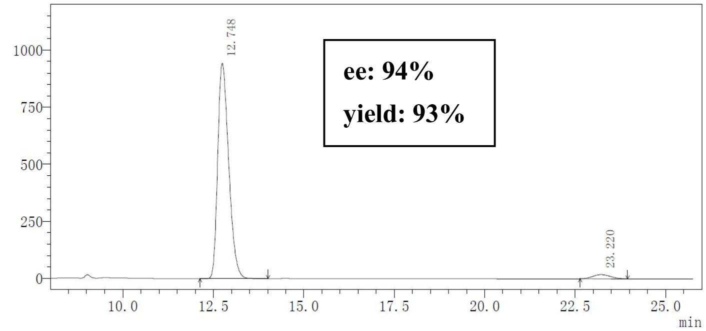

c

Serial Number	Retention Time[min]	Area[mAbs*s]	Type	Area%
1	12.748	19938310	BB	96.912
2	23.220	635347	BB	3.088
The Total		20573657		

Supplementary Figure 84. HPLC spectra for the third run. a, Racemic standard. **b**, After reaction. **c**, The detail of integration.

a

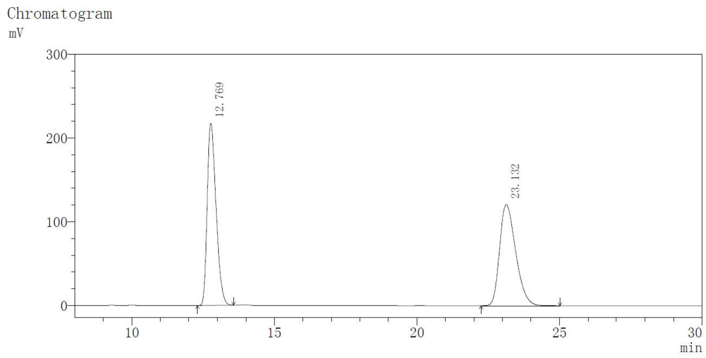

b

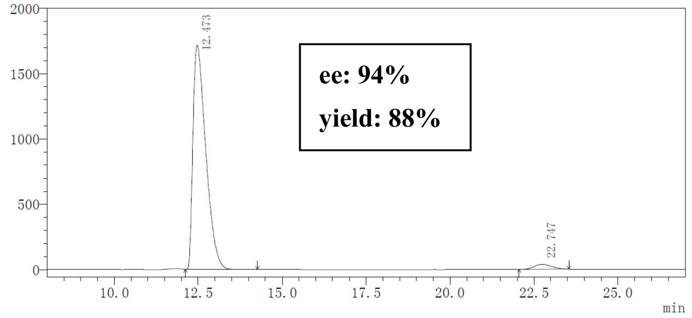

c

Serial Number	Retention Time[min]	Area[mAbs*s]	Type	Area%
1	12.473	45527542	BB	96.995
2	22.747	1410253	BB	3.005
The Total		46937795		

Supplementary Figure 85. HPLC spectra for the fourth run. a, Racemic standard. **b**, After reaction. **c**, The detail of integration.

a

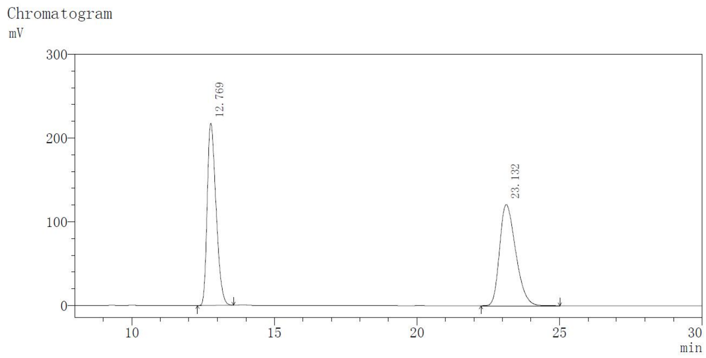

b

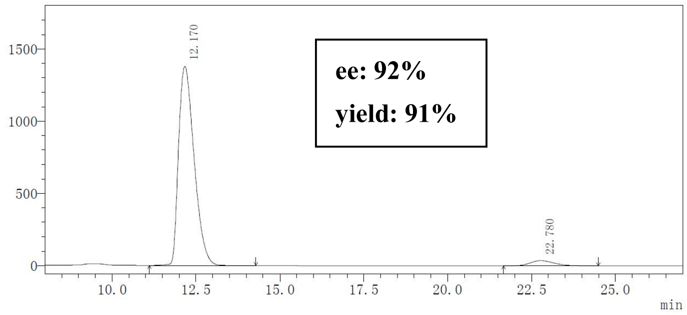

c

Serial Number	Retention Time[min]	Area[mAbs*s]	Type	Area%
1	12.170	44458733	BB	96.170
2	22.780	1770357	BB	3.830
The Total		46229090		

Supplementary Figure 86. HPLC spectra for the fifth run. **a**, Racemic standard. **b**, After reaction. **c**, The detail of integration.

a

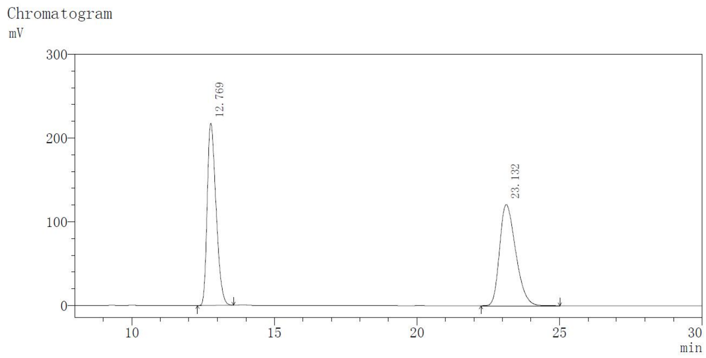

b

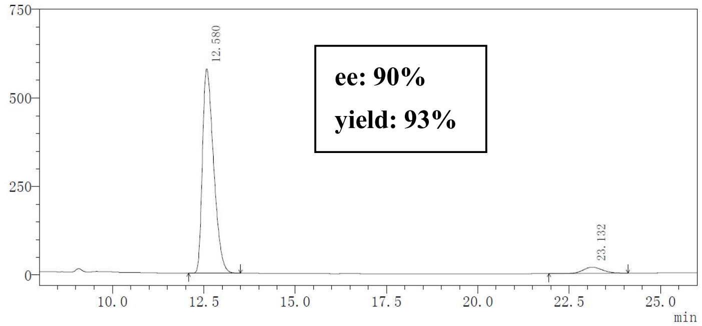

c

Serial Number	Retention Time[min]	Area[mAbs*s]	Type	Area%
1	12.580	12261499	BB	95.040
2	23.132	639883	BB	4.960
The Total		12901382		

Supplementary Figure 87. HPLC spectra for the sixth run. a, Racemic standard. **b**, After reaction. **c**, The detail of integration.

a

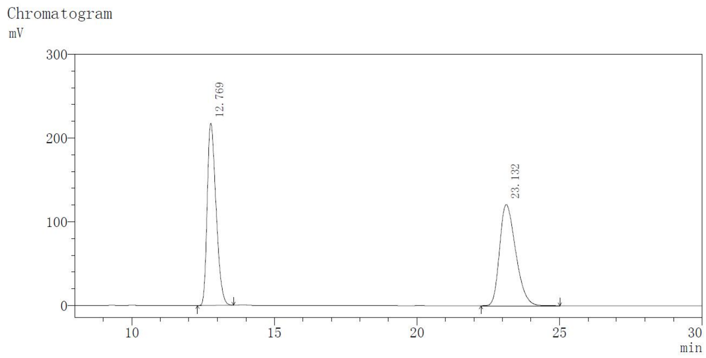

b

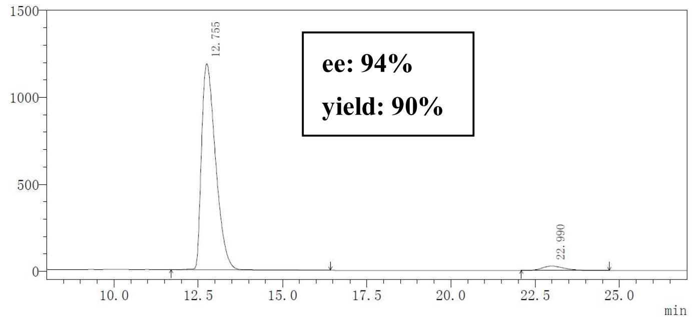

c

Serial Number	Retention Time[min]	Area[mAbs*s]	Type	Area%
1	12.755	34533252	BB	96.747
2	22.990	1161004	BB	3.253
The Total		35694257		

Supplementary Figure 88. HPLC spectra for the seventh run. a, Racemic standard. **b**, After reaction. **c**, The detail of integration.

a

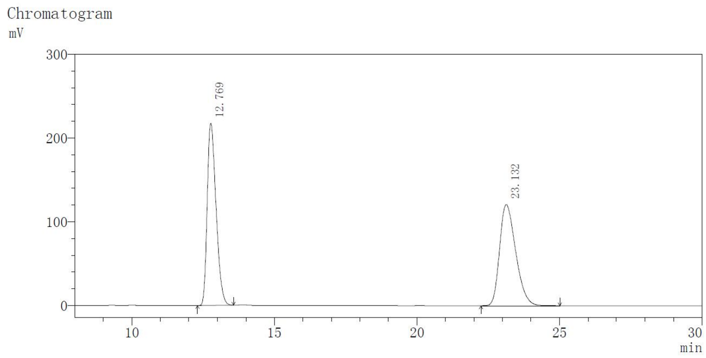

b

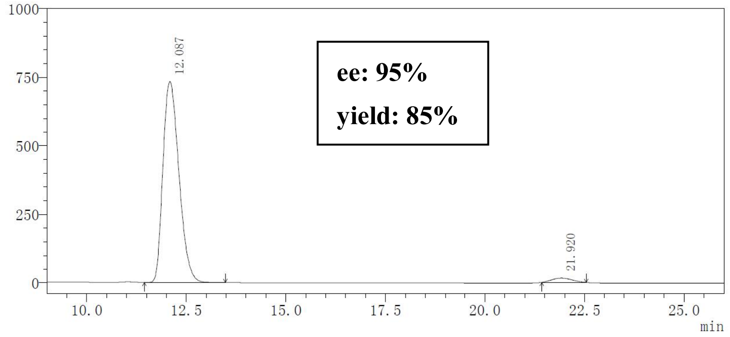

c

Serial Number	Retention Time[min]	Area[mAbs*s]	Type	Area%
1	12.087	20499969	BB	97.396
2	21.920	548089	BB	2.604
The Total		21048058		

Supplementary Figure 89. HPLC spectra for the eighth run. a, Racemic standard. **b**, After reaction. **c**, The detail of integration.

a

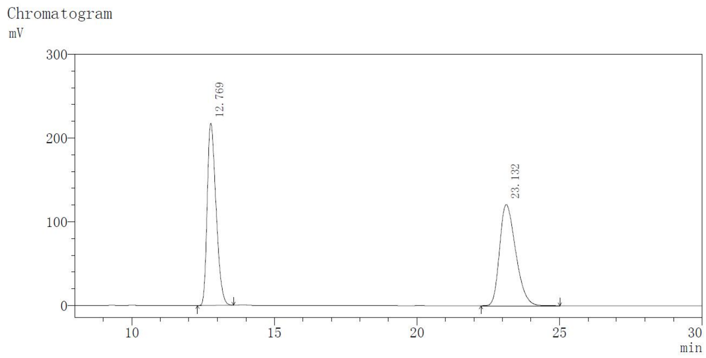

b

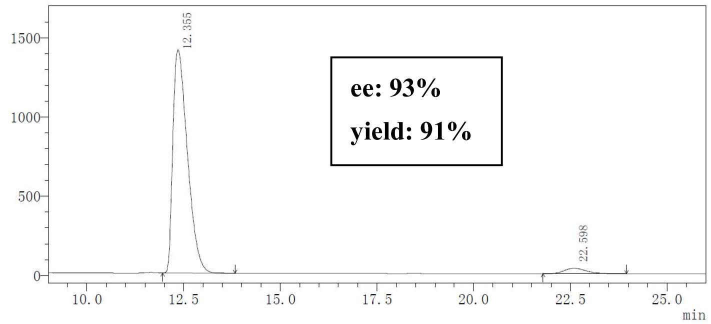

c

Serial Number	Retention Time[min]	Area[mAbs*s]	Type	Area%
1	12.355	36853281	BB	96.403
2	22.598	1375253	BB	3.597
The Total		38228535		

Supplementary Figure 90. HPLC spectra for the ninth run. a, Racemic standard. **b**, After reaction. **c**, The detail of integration.

a

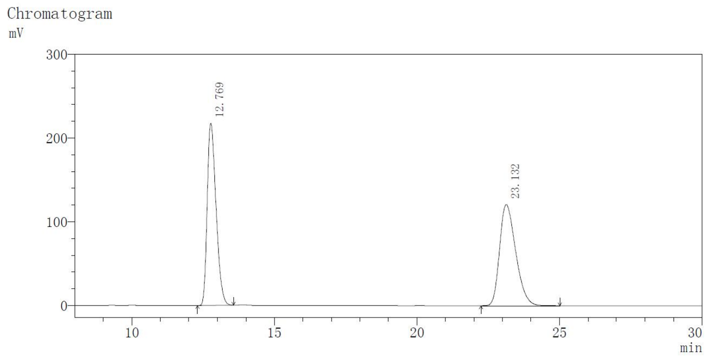

b

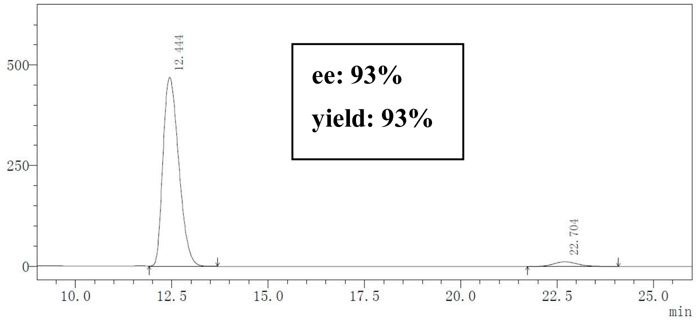

c

Serial Number	Retention Time[min]	Area[mAbs*s]	Type	Area%
1	12.444	13343090	BB	96.320
2	22.704	509781	BB	3.680
The Total		13852870		

Supplementary Figure 91. HPLC spectra for the tenth run. a, Racemic standard. **b**, After reaction. **c**, The detail of integration.

A bioactive compound obtained from propargylation of aldehydes with 1-Cr

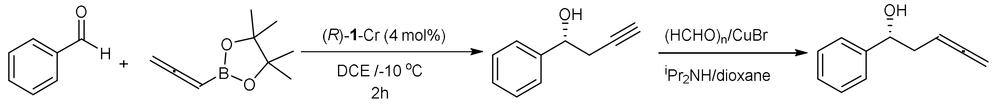

(*R*)-1-phenylpenta-3,4-dien-1-ol: Following the reported procedure (Shumaila, A. M et al. *Tetrahedron,* **2011**, *67*, 936), the title compound was obtained in 83 % two-step yield with spectral properties reported in literature. Enantiomeric excess was determined by HPLC with a chiralcel OD-H column: hexane/iPrOH = 90/10; flow rate = 1.0 mL/min; tmajor = 9.00 min, tminor = 11.87 min; ee = 92%. ^{1}H NMR (400 MHz, $CDCl_3$) δ: 7.37-7.35 (m, 4H), 7.30-7.26 (m, 1H), 5.12 (p, 1H), 4.77 (t, *J* = 6.2 Hz, 1H), 4.72 (dt, *J* = 6.4, 2.4 Hz, 2H), 2.49-2.44 (m, 2H), 2.22 (br s, 1H).

a

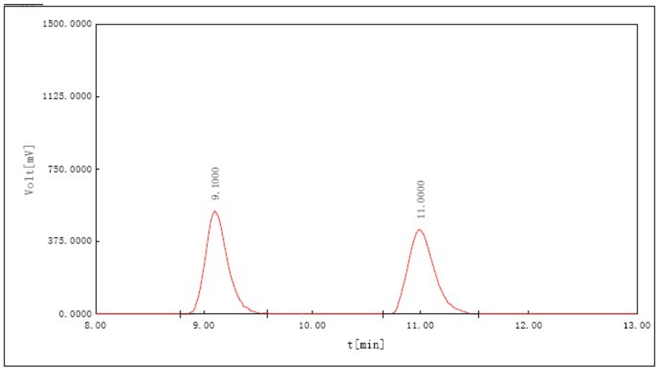

b

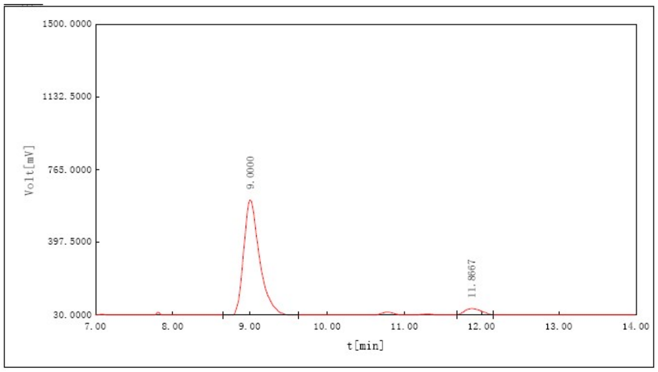

c

Serial Number	Retention Time[min]	Area[mAbs*s]	Type	Area%
1	9.0000	8720.2894	BB	95.8355
2	11.8667	378.9373	BB	4.1645
The Total		9099.2267		

Supplementary Figure 92. HPLC spectra for (*R*)-1-phenylpenta-3,4-dien-1-ol. a, Racemic standard. **b**, After reaction. **c**, The detail of integration.

(*R*)-2-(2-nitro-1-phenylethyl)-1*H*-pyrrole:

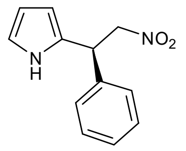

The product was prepared in 89% yield with spectral properties reported in literature. Enantiomeric excess was determined by HPLC with a chiralcel AD-H column: hexane/iPrOH = 90/10; flow rate = 1.0 mL/min; t_{major} = 9.85 min, t_{minor} = 10.83 min; ee = 90%. ^{1}H NMR (400 MHz, $CDCl_3$) δ: 7.84 (br, 1 H), 7.29-7.38 (m, 3 H), 7.22-7.25 (m, 2 H), 6.68-6.70 (m, 1 H), 6.17 (dd, J =6.0 Hz, 2.8 Hz, 1 H), 6.08-6.10 (m, 1 H), 4.99 (dd, *J* = 12.0 Hz, 4.8 Hz, 1 H), 4.90 (t, *J* = 7.2 Hz, 1 H), 4.81 (dd, *J* = 12.0 Hz, 4.8 Hz, 1 H).

a

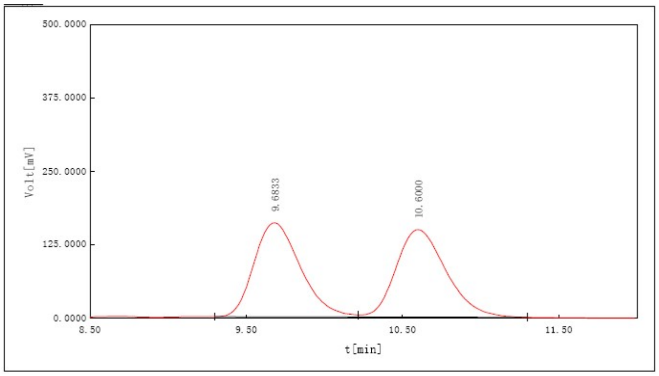

b

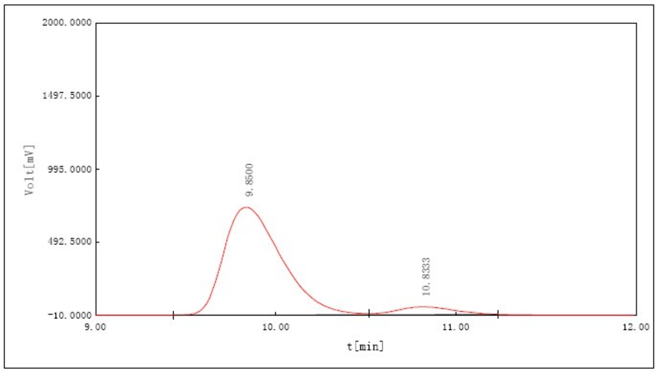

c

Serial Number	Retention Time[min]	Area[mAbs*s]	Type	Area%
1	9.8500	16678.7536	BB	94.8253
2	10.8333	910.1743	BB	5.1747
The Total		17588.9279		

Supplementary Figure 93. HPLC spectra for (*R*)-2-(2-nitro-1-phenylethyl)-1*H*-pyrrole. **a**, Racemic standard. **b**, After reaction. **c**, The detail of integration.

(*S*)-2-(1-(4-methoxyphenyl)-2-nitroethyl)-1*H*-pyrrole:

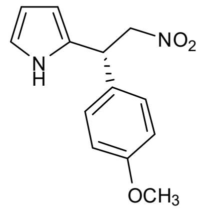

The product was prepared in 90% yield with spectral properties reported in literature. Ee was determined by HPLC with a chiralcel OD-H column: hexane/iPrOH = 80/20; flow rate = 1.0 mL/min; t_{minor} = 16.55 min, t_{major} = 20.95 min; ee = 96%. ^{1}H NMR (400 MHz, $CDCl_3$) δ: 7.86 (br, 1 H), 7.13-7.16 (m, 2 H), 6.85-6.89 (m, 2 H), 6.68 (m, 1 H), 6.16 (m, 1 H), 6.07 (m, 1 H), 4.96 (dd, *J* = 12 Hz, 6.8 Hz, 1 H), 4.84 (t, *J* = 8.0 Hz, 1 H), 4.76 (dd, *J* = 11.6 Hz, 8.0 Hz, 1 H), 3.79 (s, 3 H).

a

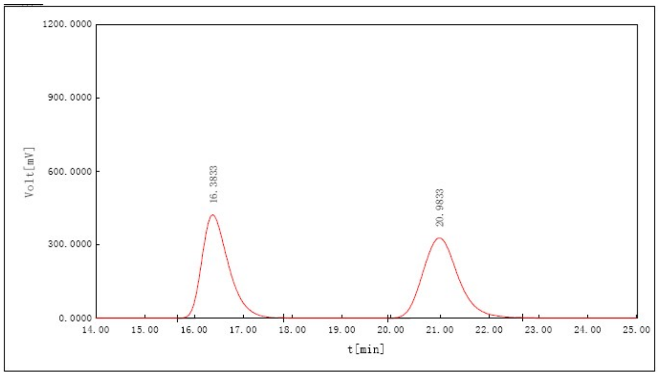

b

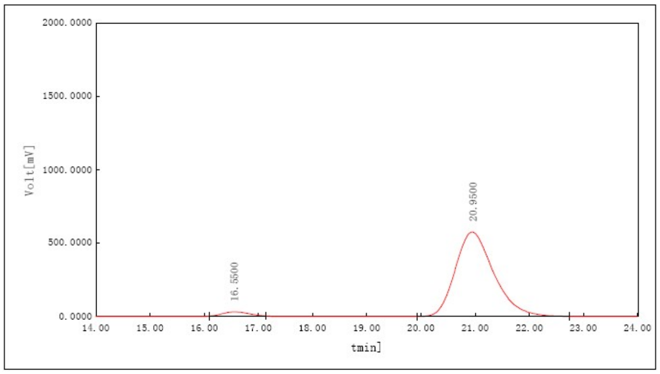

c

Serial Number	Retention Time[min]	Area[mAbs*s]	Type	Area%
1	16.5500	640.7340	BB	2.7834
2	20.9500	28704.9655	BB	97.8166
The Total		29345.6995		

Supplementary Figure 94. HPLC spectra for (*S*)-2-(1-(4-methoxyphenyl)-2-nitro ethyl)-1 *H*-pyrrole. **a**, Racemic standard. **b**, After reaction. **c**, The detail of integration.

(*S*)-2-(1-(4-ethoxyphenyl)-2-nitroethyl)-1*H*-pyrrole:

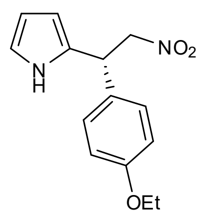

The product was prepared in 89% yield with spectral properties reported in literature. Ee was determined by HPLC with a chiralcel OD-H column: hexane/iPrOH = 80/20; flow rate = 1.0 mL/min; t_{minor} = 11.38 min, t_{major} = 12.42 min; ee = 94%. ^{1}H NMR (400 MHz, $CDCl_3$) δ: 7.85 (br, 1 H), 7.11-7.15 (m, 2 H), 6.84-6.88 (m, 2 H), 6.68 (m, 1 H), 6.16 (m, 1 H), 6.06 (m, 1 H), 4.96 (dd, *J* = 11.4 Hz, 7.0 Hz, 1 H), 4.84 (t, *J* = 7.4 Hz, 1 H), 4.76 (dd, *J* = 12.2 Hz, 8.2 Hz, 1 H), 4.00 (q, 2 H), 1.40 (t, 3 H).

a

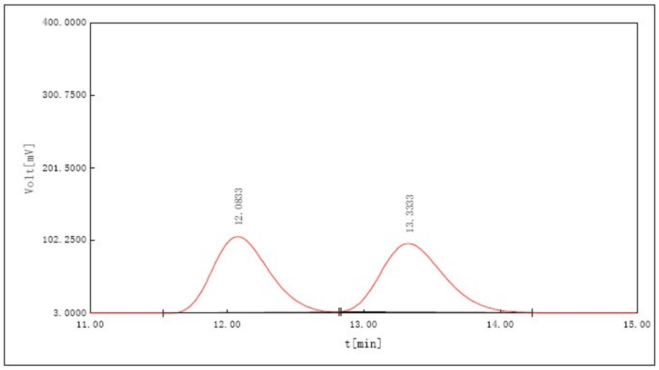

b

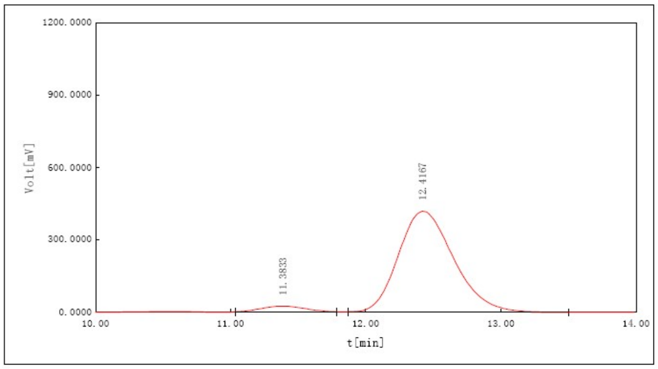

c

Serial Number	Retention Time[min]	Area[mAbs*s]	Type	Area%
1	11.3833	390.6334	BB	3.2474
2	12.4167	12020.6723	BB	96.8526
The Total		12411.3057		

Supplementary Figure 95. HPLC spectra for (*S*)-2-(1-(4-ethoxyphenyl)-2-nitro ethyl)-1*H*-pyrrole. **a**, Racemic standard. **b**, After reaction. **c**, The detail of integration.

(*R*)-2-(1-(4-bromophenyl)-2-nitroethyl)-1*H*-pyrrole:

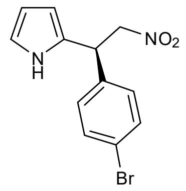

The product was prepared in 90% yield with spectral properties reported in literature. Ee was determined by HPLC with a chiralcel AD-H column: hexane/iPrOH = 90/10; flow rate = 1.0 mL/min; t_{major} = 12.77 min, t_{minor} = 14.13 min; ee = 99%. ^{1}H NMR (400 MHz, $CDCl_3$) δ: 7.85 (br, 1 H), 7.46-7.50 (m, 2 H), 7.09-7.13 (m, 2 H), 6.71 (m, 1 H), 6.16 (dd, *J* = 6.0 Hz, 2.8 Hz, 1 H), 6.07 (m, 1 H), 4.98 (dd, *J* = 12.4 Hz, 7.2 Hz, 1 H), 4.86 (t, *J* = 8.0 Hz, 1 H), 4.78 (dd, *J* = 12.0 Hz, 8.0 Hz, 1 H).

a

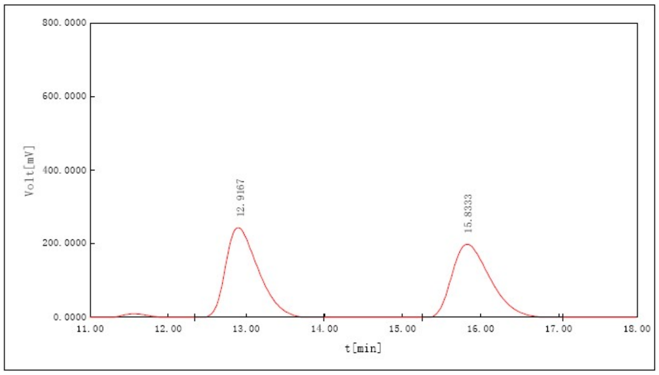

b

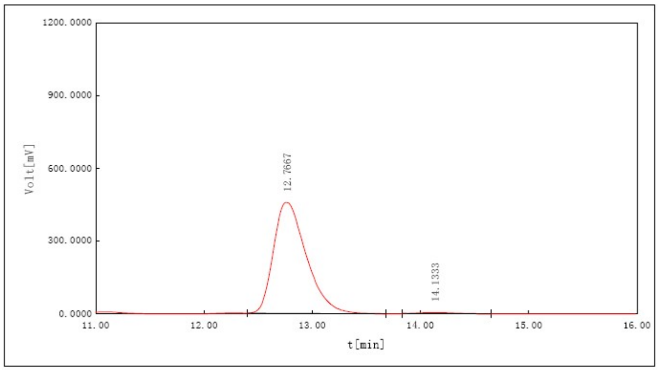

c

Serial Number	Retention Time[min]	Area[mAbs*s]	Type	Area%
1	12.7667	9683.5348	BB	99.3570
2	14.1333	62.6681	BB	0.6430
The Total		9746.2029		

Supplementary Figure 96. HPLC spectra for (*R*)-2-(1-(4-bromophenyl)-2-nitro ethyl)-1*H*-pyrrole. **a**, Racemic standard. **b**, After reaction. **c**, The detail of integration.

(*R*)-2-(1-(4-methoxycarbonyl)-2-nitroethyl)-1*H*-pyrrole:

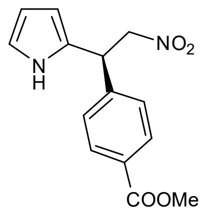

The product was prepared in 90% yield with spectral properties reported in literature. Ee was determined by HPLC with a chiralcel AD-H column: hexane/iPrOH = 90/10; flow rate = 1.0 mL/min; t_{minor} = 21.03 min, t_{major} = 32.35 min; ee = 88%. ^{1}H NMR (400 MHz, $CDCl_3$) δ: 7.93 (br, 1 H), 8.00-8.03 (m, 2 H), 7.30-7.33 (m, 2 H), 6.71 (m, 1 H), 6.17 (m, 1 H), 6.08 (m, 1 H), 5.00 (dd, *J* = 11.4 Hz, 7.0 Hz, 1 H), 4.96 (t, *J* = 7.0 Hz, 1 H), 4.83 (dd, *J* = 11.6 Hz, 6.4 Hz, 1 H).

a

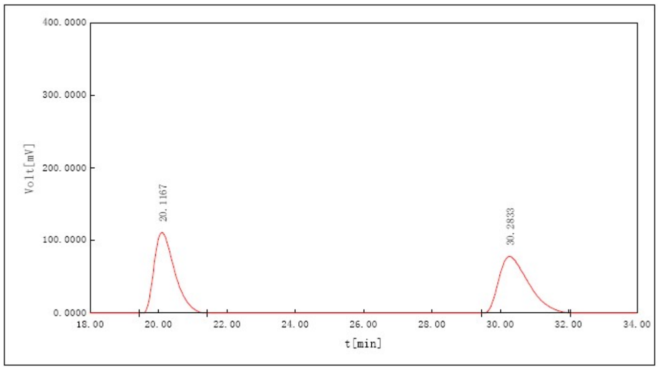

b

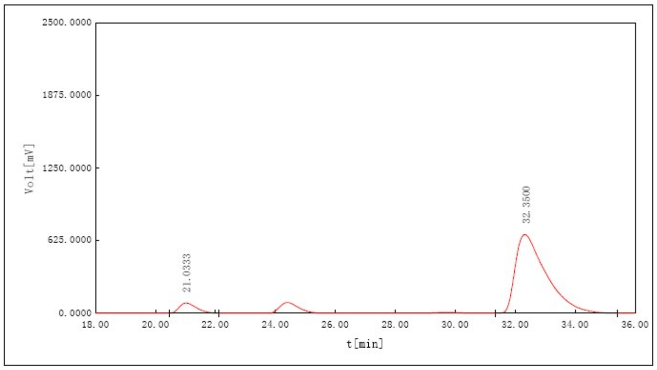

c

Serial Number	Retention Time[min]	Area[mAbs*s]	Type	Area%
1	21.0333	3218.9686	BB	6.1475
2	32.3500	49134.2705	BB	93.8525
The Total		52362.2391		

Supplementary Figure 97. HPLC spectra for (*R*)-2-(1-(4-methoxycarbonyl)-2-nitroethyl)-1 *H*-pyrrole. **a**, Racemic standard. **b**, After reaction. **c**, The detail of integration.

(*R*)-2-(1-(2-methoxyphenyl)-2-nitroethyl)-1*H*-pyrrole:

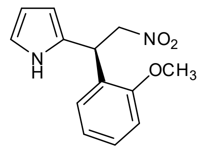

The product was prepared in 87% yield with spectral properties reported in literature. Ee was determined by HPLC with a chiralcel AD-H column: hexane/iPrOH = 95/5; flow rate = 1.0 mL/min; t_{major} = 18.00 min, t_{minor} = 21.37 min; ee = 88%. ^{1}H NMR (400 MHz, $CDCl_3$) δ: 8.28 (br, 1 H), 7.24-7.29 (m, 1H), 7.06 (dd, *J* = 7.2 Hz, 1.6 Hz, 1 H), 6.89-6.95 (m, 2 H), 6.68 (m, 1 H), 6.14 (m, 1 H), 6.10 (m, 1 H), 5.22 (m, 1 H), 4.92-4.95 (m, 2 H), 3.92 (s, 3 H).

a

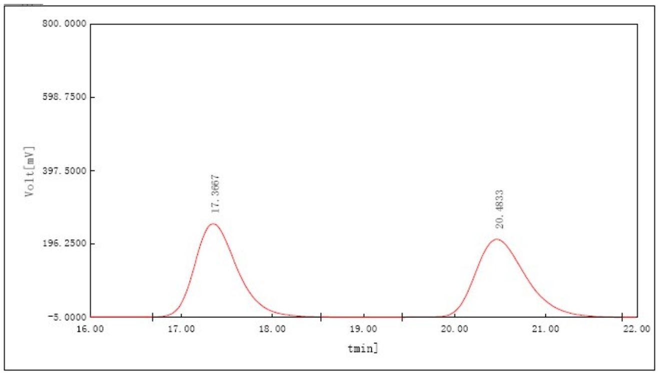

b

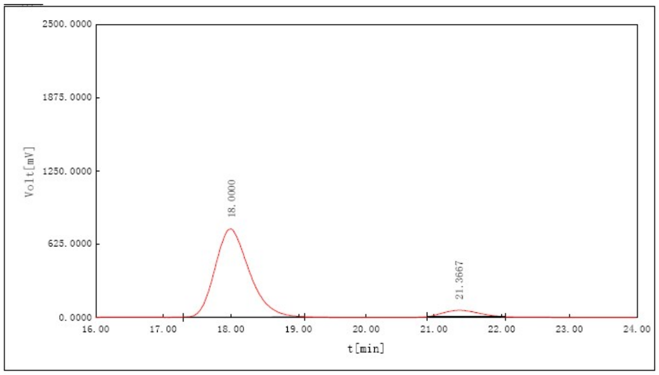

c

Serial Number	Retention Time[min]	Area[mAbs*s]	Type	Area%
1	18.0000	26571.5244	BB	94.1535
2	21.3667	1649.9697	BB	5.9465
The Total		28221.4941		

Supplementary Figure 98. HPLC spectra for (*R*)-2-(1-(2-methoxyphenyl)-2-nitro ethyl)-1*H*-pyrrole. **a**, Racemic standard. **b**, After reaction. **c**, The detail of integration.

(*R*)-2-(1-(3-chlorophenyl)-2-nitroethyl)-1*H*-pyrrole:

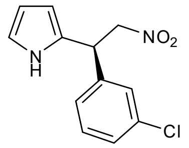

The product was prepared in 82% yield with spectral properties reported in literature. Ee was determined by HPLC with a chiralcel AD-H column: hexane/iPrOH = 90/10; flow rate = 1.0 mL/min; t_{major} = 9.25 min, t_{minor} = 10.15 min; ee = 91%. ^{1}H NMR (400 MHz, $CDCl_3$) δ: 7.89 (br, 1 H), 7.28-7.29 (m, 2 H), 7.22 (m, 1 H), 7.11-7.14 (m, 1 H), 6.72 (m, 1 H), 6.17 (dd, J = 6.4 Hz, 2.8 Hz, 1 H), 6.08 (m, 1 H), 4.97 (dd, J = 12.0 Hz, 7.0 Hz, 1 H), 4.88 (t, J = 8.0 Hz, 1 H), 4.79 (dd, J = 12.0 Hz, 8.0 Hz, 1 H).

a

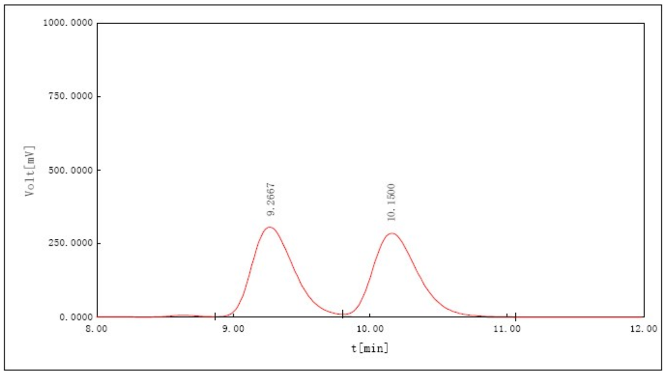

b

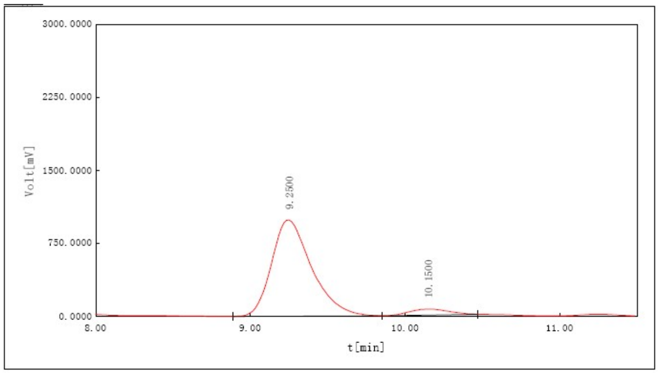

c

Serial Number	Retention Time[min]	Area[mAbs*s]	Type	Area%
1	9.2500	17942.1076	BB	95.3543
2	10.1500	874.1476	BB	4.7457
The Total		18816.2543		

Supplementary Figure 99. HPLC spectra for (*R*)-2-(1-(3-chlorophenyl)-2-nitroethyl)-1*H*-pyrrole. a, Racemic standard. **b**, After reaction. **c**, The detail of integration.

Recycling 1-Mg in Friedel-Crafts alkylation reaction.

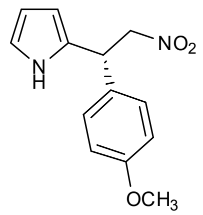

Ee was determined by HPLC with a chiralcel OD-H column: hexane/iPrOH = 80/20; flow rate = 1.0 mL/min.

a

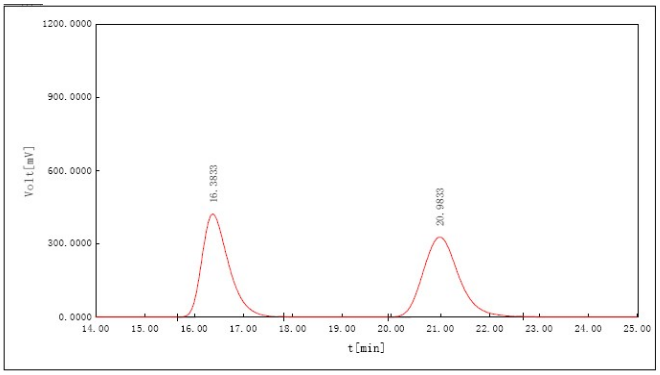

b

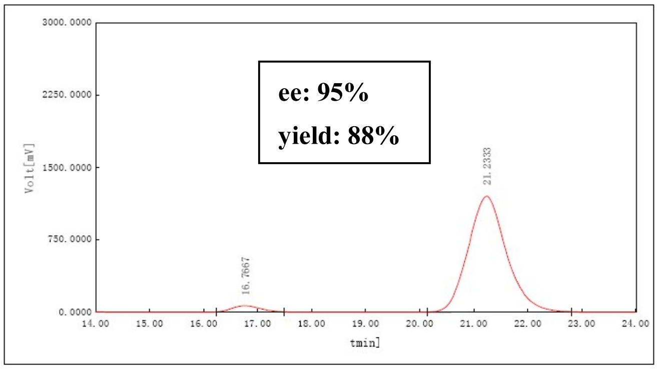

c

Serial Number	Retention Time[min]	Area[mAbs*s]	Type	Area%
1	16.7667	1490.8938	BB	2.4386
2	21.2333	59646.3888	BB	97.5614
The Total		61137.2826		

Supplementary Figure 100. HPLC spectra for the second run. a, Racemic standard. **b**, After reaction. **c**, The detail of integration.

a

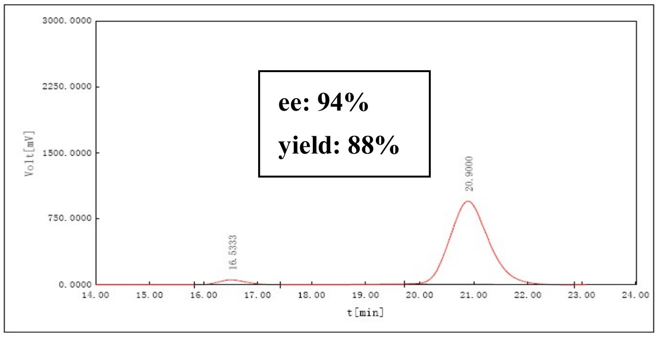

b

Serial Number	Retention Time[min]	Area[mAbs*s]	Type	Area%
1	16.5333	2074.4006	BB	3.1390
2	20.9000	47327.3584	BB	96.6610
The Total		49401.7590		

Supplementary Figure 101. HPLC spectra for the third run. a, After reaction. **b**, The detail of integration.

a

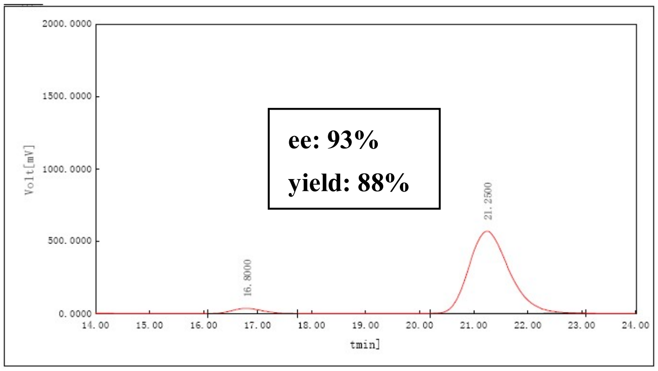

b

Serial Number	Retention Time[min]	Area[mAbs*s]	Type	Area%
1	16.8000	1062.7238	BB	3.4638
2	21.2500	29618.1402	BB	96.5362
The Total		30680.8640		

Supplementary Figure 102. HPLC spectra for the fourth run. a, After reaction. **b**, The detail of integration.

A bioactive compounds obtained from asymmetric Friedel-Crafts alkylation catalyzed by 1-Mg

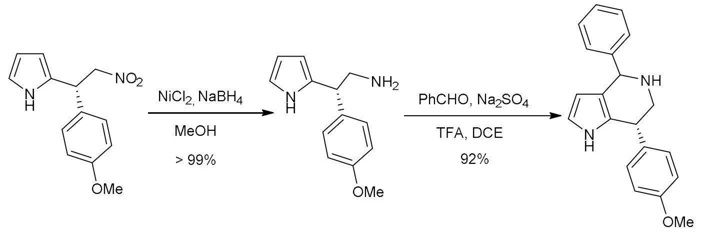

(7***S***)-4-phenyl-7-(4-methoxyphenyl)-4,5,6,7-tetrahydro-1H-pyrrolo[3,2-c]pyridine:
The desired compound was prepared according to the literature report to give the product in 86% yield with spectral properties reported in literature. Ee was determined by HPLC with a chiralcel OJ-H column: hexane/iPrOH = 90/10; flow rate = 2.0 mL/min; t_{minor} = 32.87 min, t_{major} = 45.75 min; ee = 94%. ^{1}H NMR (400MHz, $CDCl_3$) δ: 7.66 (br, 1H), 7.32-7.41 (m, 5H), 7.12 (d, *J* = 8.4Hz, 2H), 6.87 (d, *J* = 8.4Hz, 2H), 6.60 (m, 1H), 5.76 (m. 1H), 5.12 (d, 1H), 4.13 (m, 1H), 3.81 (s, 3H), 3.45 (dd, *J* = 12.6Hz, 5.4Hz, 1H), 2.99 (dd, *J*=12.6, 9.4 Hz, 1H).

a

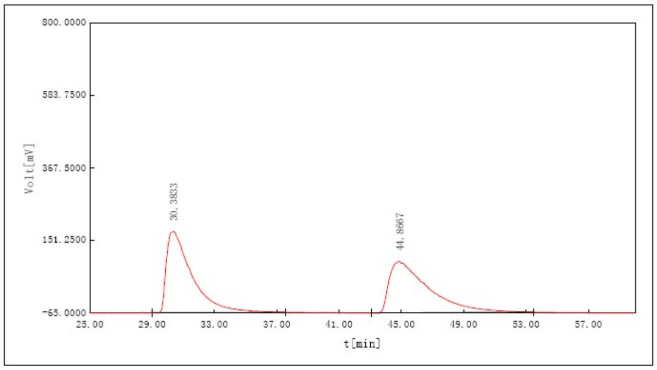

b

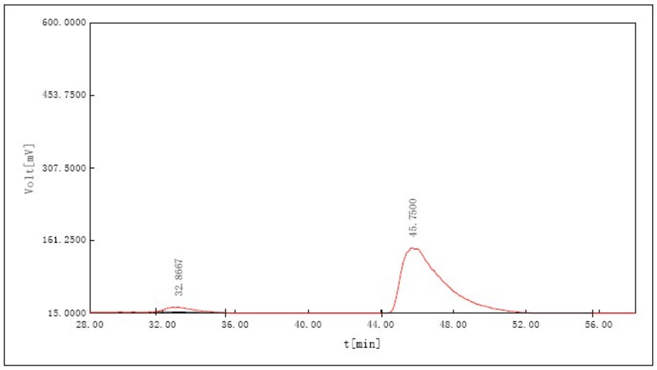

c

Serial Number	Retention Time[min]	Area[mAbs*s]	Type	Area%
1	32.8667	755.6563	BB	3.2447
2	45.7500	22533.2842	BB	96.7553
The Total		23288.9405		

Supplementary Figure 103. HPLC spectra for (7*S*)-4-phenyl-7-(4-methoxy phenyl)-4,5,6,7-tetrahydro-1*H*-pyrrolo[3,2-c]pyridine. **a**, Racemic standard. **b**, After reaction. **c**, The detail of integration.

(*S*)-Phenyl methyl sulfoxide:

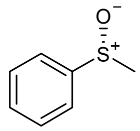

The compound was obtained in 93 % yield. Enantiomeric excess was determined by HPLC with a chiralcel OD-H column: hexane/iPrOH = 90/10; flow rate = 1.0 mL/min; t_{major} = 13.73 min, t_{minor} = 20.08 min; ee = 92%. ^{1}H NMR (400 MHz, $CDCl_3$) δ: 2.72 (s, 3H, CH_3), 7.51-7.53 (m, 3H, ArH), 7.64-7.66 (m, 2H, ArH).

a

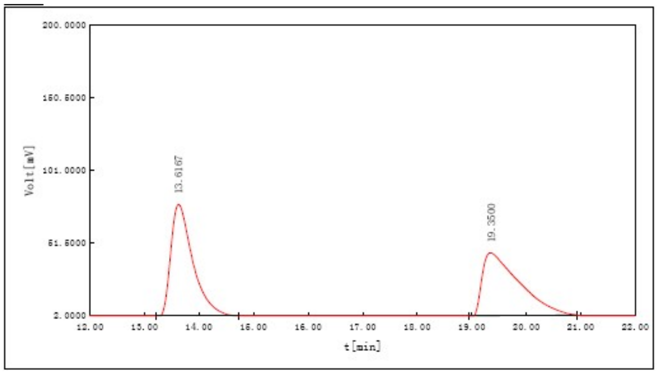

b

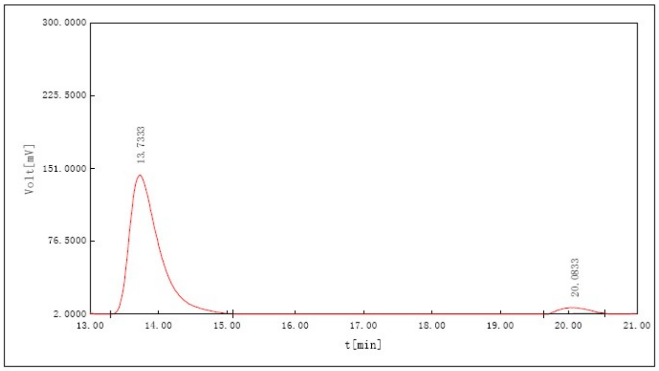

c

Serial Number	Retention Time[min]	Area[mAbs*s]	Type	Area%
1	13.7333	4348.0603	BB	95.7683
2	20.0833	192.1271	BB	4.2317
The Total		4540.1874		

Supplementary Figure 104. HPLC spectra for (*S*)-Phenyl methyl sulfoxide. a, Racemic standard. **b**, After reaction. **c**, The detail of integration.

(*S*)-*p*-Tolyl methyl sulfoxide:

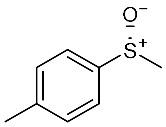

The product was obtained in 95 % yield. ee was determined by HPLC with a chiralcel OD-H column: hexane/iPrOH = 90/10; flow rate = 1.0 mL/min; t_{major} = 12.60 min, t_{minor} = 14.75 min; ee = 93%. ^{1}H NMR (400 MHz, $CDCl_3$) δ: 2.40 (s, 3H, Ar-CH_3), 2.69 (s, 3H, CH_3), 7.32 (d, *J* = 8.0 Hz, 2H, ArH), 7.53 (d, *J* = 8.0 Hz, 2H, ArH).

a

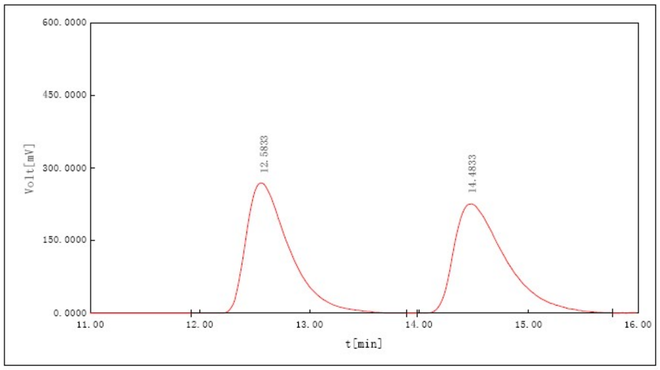

b

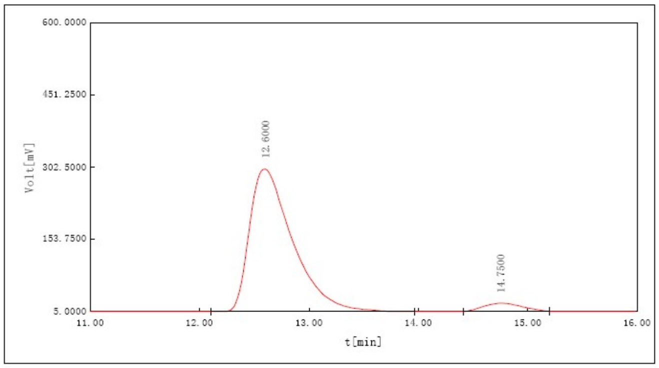

c

Serial Number	Retention Time[min]	Area[mAbs*s]	Type	Area%
1	12.6000	83049.3312	BB	96.3174
2	14.7500	317.5079	BB	3.6826
The Total		8621.8402		

Supplementary Figure 105. HPLC spectra for (*S*)-*p*-Tolyl methyl sulfoxide. a, Racemic standard. **b**, After reaction. **c**, The detail of integration.

(*S*)-*p*-Methoxyphenyl methyl sulfoxide:

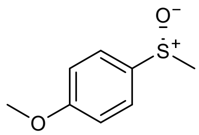

The product was obtained in 92 % yield. ee was determined by HPLC with a chiralcel OD-H column: hexane/iPrOH = 90/10; flow rate = 1.0 mL/min; t_{major} = 21.45 min, t_{minor} = 26.58 min; ee = 84%. ^{1}H NMR (400 MHz, $CDCl_3$) δ: 2.68 (s, 3H, CH_3), 3.84 (s, 3H, OCH_3), 7.01 (d, *J* = 8.8, 2H, ArH), 7.58 (dt, *J* = 8.8, 2H, ArH).

a

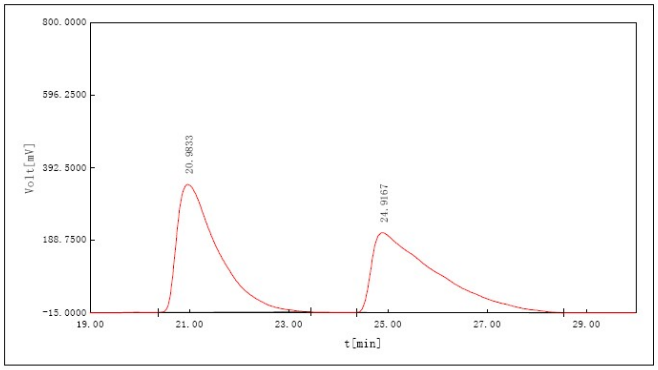

b

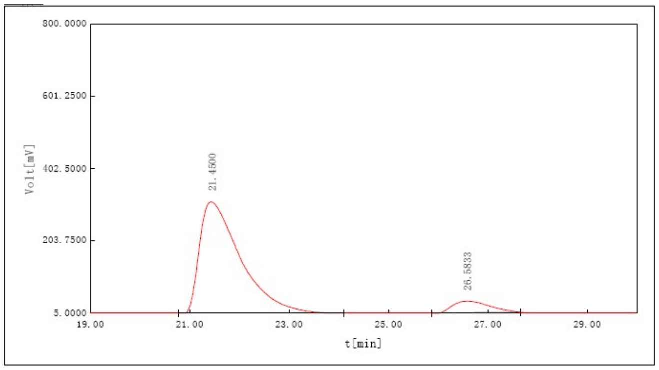

c

Serial Number	Retention Time[min]	Area[mAbs*s]	Type	Area%
1	21.4500	18554.3601	BB	91.8524
2	26.5833	1645.8308	BB	8.1476
The Total		20200.1909		

Supplementary Figure 106. HPLC spectra for (*S*)-*p*-Methoxyphenyl methyl sulfoxide. a, Racemic standard. **b**, After reaction. **c**, The detail of integration.

(*S*)-*p*-Chlorophenyl methyl sulfoxide:

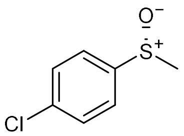

The product was obtained in 94 % yield. Ee was determined by HPLC with a chiralcel OD-H column: hexane/iPrOH = 95/5; flow rate = 1.0 mL/min; t_{major} = 24.17 min, t_{minor} = 25.97 min; ee = 89%. ^{1}H NMR (400 MHz, $CDCl_3$) δ: 2.71 (s, 3H, CH_3), 7.51 (d, *J* = 8.4 Hz, 2H, ArH), 7.58 (d, *J* = 8.4 Hz, 2H, ArH).

a

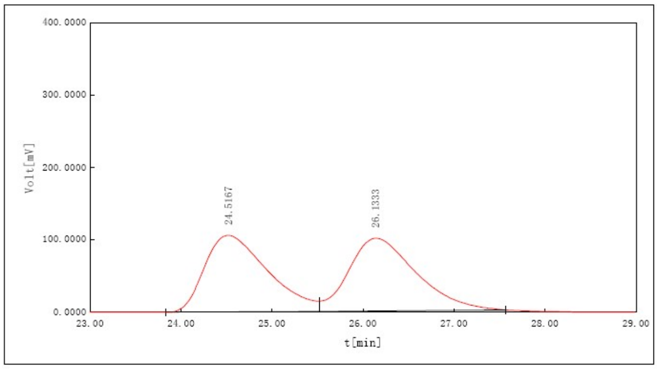

b

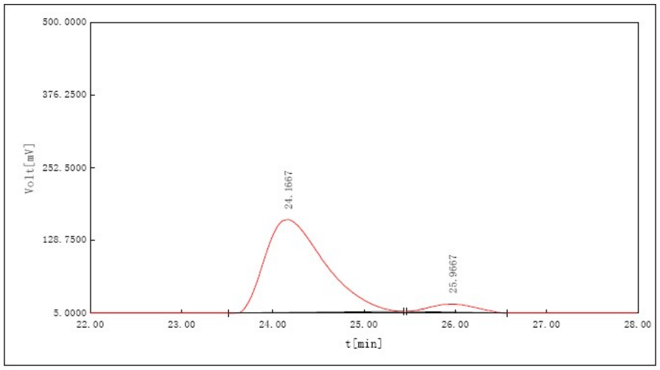

c

Serial Number	Retention Time[min]	Area[mAbs*s]	Type	Area%
1	24.1667	75843.0838	BB	94.2921
2	25.9667	459.0967	BB	5.7079
The Total		8043.1805		

Supplementary Figure 107. HPLC spectra for (*S*)-*p*-Chlorophenyl methyl sulfoxide. a, Racemic standard. **b**, After reaction. **c**, The detail of integration.

(*S*)-*m*-Methoxyphenyl methyl sulfoxide:

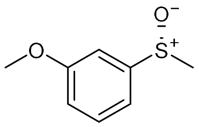

The product was obtained in 86 % yield. ee was determined by HPLC with a chiralcel OD-H column: hexane/iPrOH = 90/10; flow rate = 1.0 mL/min; t_{major} = 15.60 min, t_{minor} = 24.03 min; ee = 55%. ^{1}H NMR (400 MHz, $CDCl_3$) δ: 2.70 (s, 3H, CH_3), 3.84 (s, 3H, OCH_3), 6.98-7.00 (m, 1H, ArH), 7.10-7.12 (m, 1H, ArH), 6.23-7-24 (m, 1H, ArH), 7.37-7.41 (m, 1H, ArH).

a

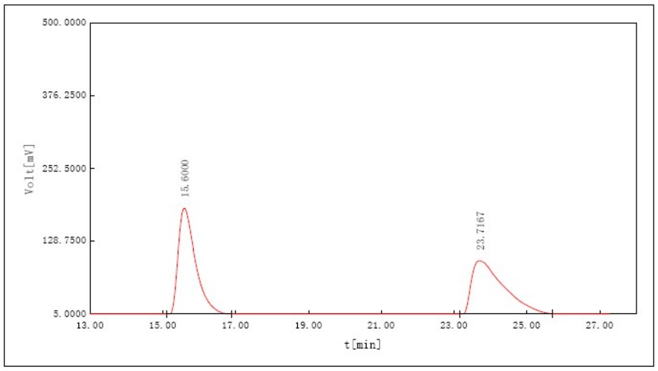

b

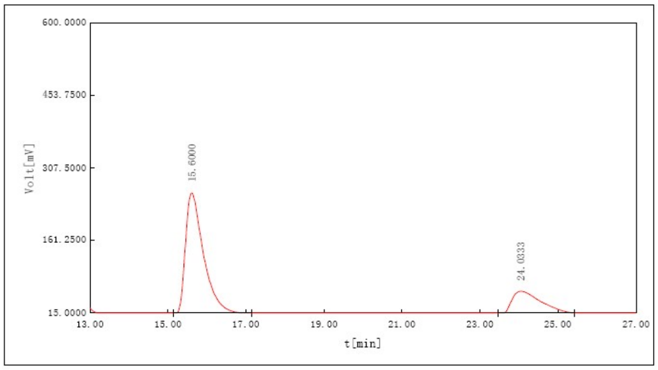

c

Serial Number	Retention Time[min]	Area[mAbs*s]	Type	Area%
1	15.6000	8187.0763	BB	77.5604
2	24.0333	2238.6665	BB	22.4396
The Total		10555.7428		

Supplementary Figure 108. HPLC spectra for (*S*)-*m*-Methoxyphenyl methyl sulfoxide. a, Racemic standard. **b**, After reaction. **c**, The detail of integration.

(*S*)-*m*-Fluorophenyl methyl sulfoxide:

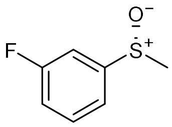

The product was obtained in 91 % yield. Ee was determined by HPLC with a chiralcel OD-H column: hexane/iPrOH = 90/10; flow rate = 1.0 mL/min; t_{major} = 12.30 min, t_{minor} = 14.28 min; ee = 87 %. ^{1}H NMR (400 MHz, $CDCl_3$) δ: 2.73 (s, 3H, CH_3), 7.16-7.21 (m, 1H, ArH), 7.37-7.47 (m, 2H, ArH), 7.49-7.53 (m, 1H, ArH).

a

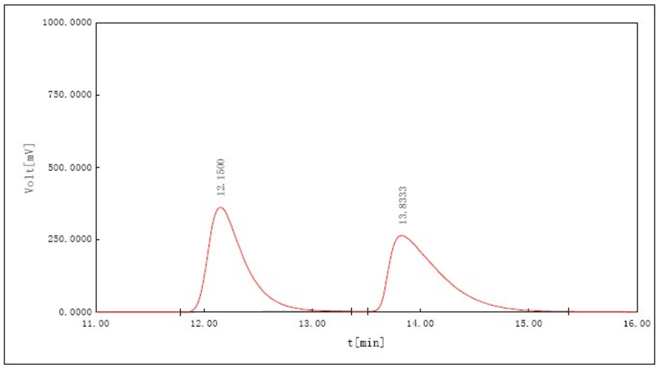

b

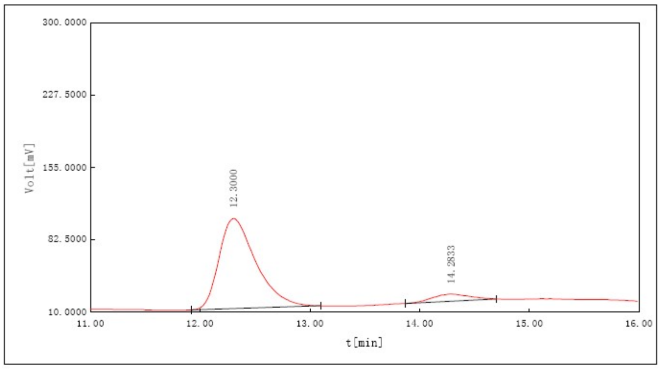

c

Serial Number	Retention Time[min]	Area[mAbs*s]	Type	Area%
1	12.3000	2133.3686	BB	93.6035
2	14.2833	145.7861	BB	6.3965
The Total		2279.1548		

Supplementary Figure 109. HPLC spectra for (*S*)-*m*-Fluorophenyl methyl sulfoxide. a, Racemic standard. **b**, After reaction. **c**, The detail of integration.

Recycling 1-Mn in oxidation of *p*-tolyl methyl sulfide

a

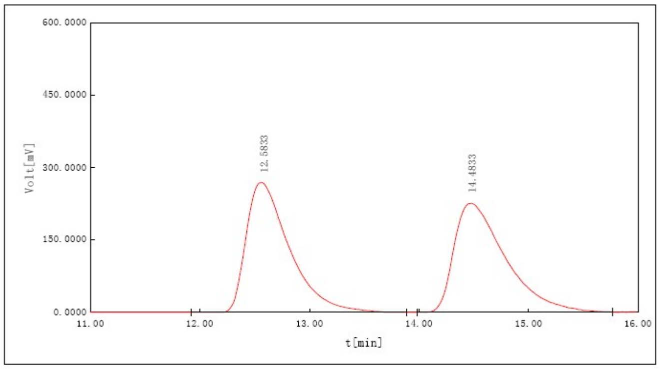

b

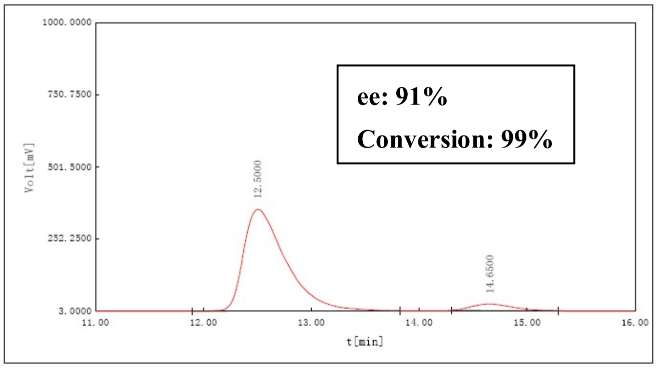

c

Serial Number	Retention Time[min]	Area[mAbs*s]	Type	Area%
1	12.5000	9806.0065	BB	95.3317
2	14.6500	480.1905	BB	4.6683
The Total		10286.1970		

Supplementary Figure 110. HPLC spectra for the second run. a, Racemic standard. **b**, After reaction. **c**, The detail of integration.

a

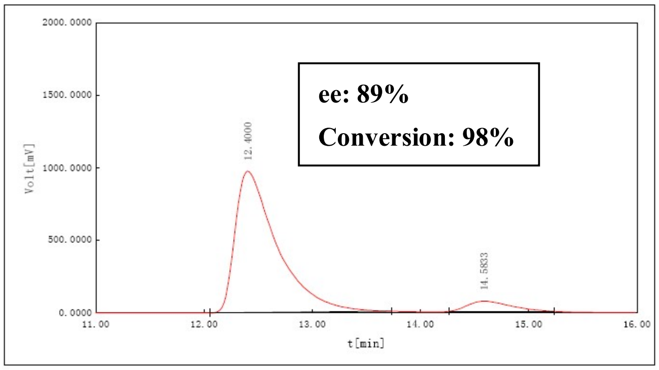

b

Serial Number	Retention Time[min]	Area[mAbs*s]	Type	Area%
1	12.4000	26503.6020	BB	94.1688
2	14.5833	1641.1784	BB	5.8412
The Total		28144.7804		

Supplementary Figure 103. HPLC spectra for the third run. a, After reaction. **b**, The detail of integration.

a

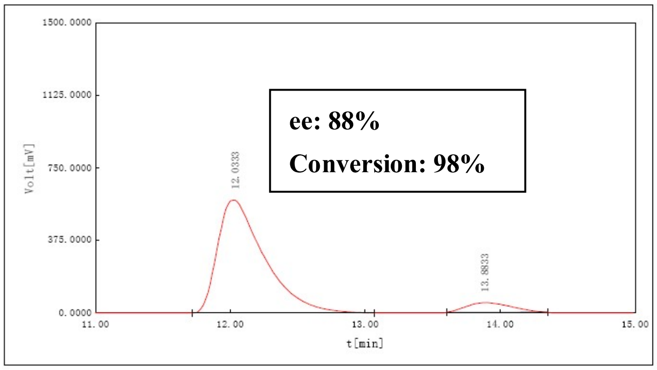

b

Serial Number	Retention Time[min]	Area[mAbs*s]	Type	Area%
1	12.0333	14474.9395	BB	93.8343
2	13.8833	951.1249	BB	6.1657
The Total		15426.0644		

Supplementary Figure 111. HPLC spectra for the fourth run. a, After reaction. **b**, The detail of integration.

2-Phenyl-1,2,3,4-tetrahydroquinoxaline:

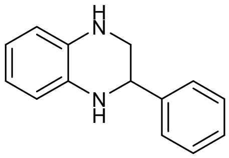

^{1}H NMR (400 MHz, $CDCl_3$) δ: 7.47-7.31 (m, 5H), 6.73-6.56 (m, 4H), 4.52 (d, *J = 5.9 Hz*, 1H), 3.50 (d, *J = 12.5 Hz*, 1H), 3.41-3.30 (m, 1H).

Supplementary References.

1. Rueping, M., Tato, F. & Schoepke, F. R. The First General, Efficient and Highly Enantioselective Reduction of Quinoxalines and Quinoxalinones. *Chem. Eur. J.* **16**, 2688-2691 (2010)

2. Kobayashi, S., Endo, T., Schneider, U. & Ueno, M. Aldehyde allylation with allylboronates providing a-addition products. *Chem. Commun.* **46**, 1260-1262 (2010).

3. Li, W., Su, Z. & Hu, C. Mechanism of Ketone Allylation with Allylboronates as Catalyzed by Zinc Compounds: A DFT Study. *Chem. Eur. J.* **19**, 124-134 (2013).

4. Kobayashi, S., Endo, T., Yoshino, T., Schneider, U. & Ueno, M. Allylation Reactions of Aldehydes with Allylboronates in Aqueous Media: Unique Reactivity and Selectivity that are Only Observed in the Presence of Water. *Chem. – Asian. J.* **8**, 2033-2045 (2013)

5. Dolomanov, O. V., Bourhis, L., J, Gildea R., J, Howard, J., Puschmann, H. OLEX2: A complete structure solution, refinement and analysis program, *J. Appl. Crystallogr.* **42**, 339-341 (2009)
