# Supplementary material for: Sixteen isostructural phosphonate metal-organic frameworks with controlled Lewis acidity and chemical stability for asymmetric catalysis
Source: Nat Commun. 2017 Dec 19;8:2171. doi: 10.1038/s41467-017-02335-0 (PMC5736719; doi:10.1038/s41467-017-02335-0)

checkCIF/PLATON report

You have not supplied any structure factors. As a result the full set of tests cannot be run.

THIS REPORT IS FOR GUIDANCE ONLY. IF USED AS PART OF A REVIEW PROCEDURE FOR PUBLICATION, IT SHOULD NOT REPLACE THE EXPERTISE OF AN EXPERIENCED CRYSTALLOGRAPHIC REFEREE.

No syntax errors found.   CIF dictionary   Interpreting this report

Datablock: 1-Cr

Bond precision:	C-C = 0.0088 A	Wavelength=1.54178	
Cell:	a=26.8122(5)	b=29.1445(5)	c=29.4502(5)
	alpha=90	beta=90	gamma=90
Temperature:	123 K		

	Calculated	Reported
Volume	23013.2(7)	23013.2(7)
Space group	C 2 2 21	C222(1)
Hall group	C 2c 2	?
Moiety formula	C74 H71 Cr3 O19 P2, 2(C2 O2)	?
Sum formula	C78 H71 Cr3 O23 P2	C78 H79 Cr3 O23 P2
Mr	1594.29	1602.35
Dx,g cm-3	0.920	0.925
Z	8	8
Mu (mm-1)	2.980	2.980
F000	6600.0	6664.0
F000’	6620.80	
h,k,lmax	28,30,31	28,30,31
Nref	14451[7756]	13840
Tmin,Tmax	0.788,0.836	0.788,0.836
Tmin’	0.788	

Correction method= # Reported T Limits: Tmin=0.788 Tmax=0.836
AbsCorr = MULTI-SCAN

Data completeness= 1.78/0.96   Theta(max)= 55.000

R(reflections)= 0.0893(13059)   wR2(reflections)= 0.2788(13840)

S = 1.158   Npar= 790

The following ALERTS were generated. Each ALERT has the format
test-name_ALERT_alert-type_alert-level.
Click on the hyperlinks for more details of the test.

Alert level A

```
THETM01_ALERT_3_A  The value of sine(theta_max)/wavelength is less than 0.550
            Calculated sin(theta_max)/wavelength =    0.5313
PLAT602_ALERT_2_A VERY LARGE Solvent Accessible VOID(S) in Structure        ! Info
```

Alert level B

```
PLAT035_ALERT_1_B _chemical_absolute_configuration info  Not given     Please Do !
PLAT049_ALERT_1_B Calculated Density less than 1.0 gcm-3 .........     0.9203 Check
PLAT230_ALERT_2_B Hirshfeld Test Diff for    C25    --  C26     ..        8.9 s.u.
PLAT234_ALERT_4_B Large Hirshfeld Difference C61    --  C64     ..       0.29 Ang.
PLAT430_ALERT_2_B Short Inter D...A Contact  O23    ..  O23     ..       2.59 Ang.
```

Alert level C

```
RFACR01_ALERT_3_C  The value of the weighted R factor is > 0.25
            Weighted R factor given   0.279
PLAT041_ALERT_1_C Calc. and Reported SumFormula    Strings  Differ     Please Check
PLAT043_ALERT_1_C Calculated and Reported Mol. Weight Differ by ..       8.06 Check
PLAT068_ALERT_1_C Reported F000 Differs from Calcd (or Missing)...     Please Check
PLAT084_ALERT_3_C High wR2 Value (i.e. > 0.25) ...................       0.28 Report
PLAT202_ALERT_3_C Isotropic non-H Atoms in Anion/Solvent .........          8 Check
PLAT220_ALERT_2_C Large Non-Solvent  C     Ueq(max)/Ueq(min) Range        4.3 Ratio
PLAT222_ALERT_3_C Large Non-Solvent  H     Uiso(max)/Uiso(min) ...        4.5 Ratio
PLAT230_ALERT_2_C Hirshfeld Test Diff for    C14    --  C16     ..        6.9 s.u.
PLAT230_ALERT_2_C Hirshfeld Test Diff for    C46    --  C47     ..        5.2 s.u.
PLAT234_ALERT_4_C Large Hirshfeld Difference C57    --  C58     ..       0.16 Ang.
PLAT234_ALERT_4_C Large Hirshfeld Difference C59    --  C60     ..       0.16 Ang.
PLAT234_ALERT_4_C Large Hirshfeld Difference C61    --  C62     ..       0.20 Ang.
PLAT234_ALERT_4_C Large Hirshfeld Difference C66    --  C67     ..       0.20 Ang.
PLAT234_ALERT_4_C Large Hirshfeld Difference C69    --  C72     ..       0.16 Ang.
PLAT241_ALERT_2_C High   'MainMol' Ueq as Compared to Neighbors of         O5 Check
PLAT241_ALERT_2_C High   'MainMol' Ueq as Compared to Neighbors of        O13 Check
PLAT241_ALERT_2_C High   'MainMol' Ueq as Compared to Neighbors of        C58 Check
PLAT241_ALERT_2_C High   'MainMol' Ueq as Compared to Neighbors of        C67 Check
PLAT242_ALERT_2_C Low    'MainMol' Ueq as Compared to Neighbors of        Cr1 Check
PLAT242_ALERT_2_C Low    'MainMol' Ueq as Compared to Neighbors of        Cr3 Check
PLAT242_ALERT_2_C Low    'MainMol' Ueq as Compared to Neighbors of        C25 Check
PLAT242_ALERT_2_C Low    'MainMol' Ueq as Compared to Neighbors of        C50 Check
PLAT242_ALERT_2_C Low    'MainMol' Ueq as Compared to Neighbors of        C59 Check
PLAT242_ALERT_2_C Low    'MainMol' Ueq as Compared to Neighbors of        C66 Check
PLAT242_ALERT_2_C Low    'MainMol' Ueq as Compared to Neighbors of        C69 Check
PLAT244_ALERT_4_C Low    'Solvent' Ueq as Compared to Neighbors of        C75 Check
PLAT341_ALERT_3_C Low Bond Precision on  C-C Bonds ...............    0.00875 Ang.
PLAT369_ALERT_2_C Long    C(sp2)-C(sp2) Bond  C10    -   C19    ..       1.55 Ang.
PLAT369_ALERT_2_C Long    C(sp2)-C(sp2) Bond  C46    -   C55    ..       1.53 Ang.
```

Alert level G

```
FORMU01_ALERT_2_G  There is a discrepancy between the atom counts in the
            _chemical_formula_sum and the formula from the _atom_site* data.
            Atom count from _chemical_formula_sum:C78 H79 Cr3 O23 P2
            Atom count from the _atom_site data:  C78 H71 Cr3 O23 P2
CELLZ01_ALERT_1_G Difference between formula and atom_site contents detected.
CELLZ01_ALERT_1_G WARNING: H atoms missing from atom site list. Is this intentional?
           From the CIF: _cell_formula_units_Z    8
```

```
          From the CIF: _chemical_formula_sum  C78 H79 Cr3 O23 P2
          TEST: Compare cell contents of formula and atom_site data

          atom    Z*formula  cif sites diff
          C        624.00    624.00    0.00
          H        632.00    568.00   64.00
          Cr        24.00     24.00    0.00
          O        184.00    184.00    0.00
          P         16.00     16.00    0.00
PLAT002_ALERT_2_G Number of Distance or Angle Restraints on AtSite         32 Note
PLAT004_ALERT_5_G Polymeric Structure Found with Maximum Dimension          3 Info
PLAT005_ALERT_5_G No Embedded Refinement Details found in the CIF      Please Do !
PLAT033_ALERT_4_G Flack x Value Deviates > 3.0 * sigma from Zero .      0.282 Note
PLAT066_ALERT_1_G Predicted and Reported Tmin&Tmax Range Identical          ? Check
PLAT072_ALERT_2_G SHELXL First  Parameter in WGHT  Unusually Large       0.23 Report
PLAT232_ALERT_2_G Hirshfeld Test Diff (M-X)  Cr1    --  O17     ..        5.5 s.u.
PLAT232_ALERT_2_G Hirshfeld Test Diff (M-X)  Cr1    --  O9_c    ..       10.0 s.u.
PLAT232_ALERT_2_G Hirshfeld Test Diff (M-X)  Cr2    --  O6      ..       11.5 s.u.
PLAT232_ALERT_2_G Hirshfeld Test Diff (M-X)  Cr2    --  O14     ..        9.3 s.u.
PLAT232_ALERT_2_G Hirshfeld Test Diff (M-X)  Cr2    --  O8_b    ..        5.7 s.u.
PLAT232_ALERT_2_G Hirshfeld Test Diff (M-X)  Cr2    --  O10_c   ..       10.0 s.u.
PLAT232_ALERT_2_G Hirshfeld Test Diff (M-X)  Cr2    --  O2_i    ..        5.7 s.u.
PLAT232_ALERT_2_G Hirshfeld Test Diff (M-X)  Cr3    --  O13     ..        8.3 s.u.
PLAT344_ALERT_2_G Unusual sp?     Angle Range in Solvent/Ion for .     C76    Check
PLAT344_ALERT_2_G Unusual sp?     Angle Range in Solvent/Ion for .     C78    Check
PLAT380_ALERT_4_G Incorrectly? Oriented X(sp2)-Methyl Moiety .....        C18 Check
PLAT380_ALERT_4_G Incorrectly? Oriented X(sp2)-Methyl Moiety .....        C29 Check
PLAT380_ALERT_4_G Incorrectly? Oriented X(sp2)-Methyl Moiety .....        C54 Check
PLAT380_ALERT_4_G Incorrectly? Oriented X(sp2)-Methyl Moiety .....        C65 Check
PLAT791_ALERT_4_G The Model has Chirality at P1      (Chiral SPGR)          S Verify
PLAT791_ALERT_4_G The Model has Chirality at P2      (Chiral SPGR)          R Verify
PLAT860_ALERT_3_G Number of Least-Squares Restraints .............         33 Note
PLAT899_ALERT_4_G SHELXL97   is Deprecated and Succeeded by SHELXL       2014 Note
```

---

2 **ALERT level A** = Most likely a serious problem - resolve or explain
5 **ALERT level B** = A potentially serious problem, consider carefully
30 **ALERT level C** = Check. Ensure it is not caused by an omission or oversight
27 **ALERT level G** = General information/check it is not something unexpected

8 ALERT type 1 CIF construction/syntax error, inconsistent or missing data
32 ALERT type 2 Indicator that the structure model may be wrong or deficient
7 ALERT type 3 Indicator that the structure quality may be low
15 ALERT type 4 Improvement, methodology, query or suggestion
2 ALERT type 5 Informative message, check

It is advisable to attempt to resolve as many as possible of the alerts in all categories. Often the minor alerts point to easily fixed oversights, errors and omissions in your CIF or refinement strategy, so attention to these fine details can be worthwhile. In order to resolve some of the more serious problems it may be necessary to carry out additional measurements or structure refinements. However, the purpose of your study may justify the reported deviations and the more serious of these should normally be commented upon in the discussion or experimental section of a paper or in the "special_details" fields of the CIF. checkCIF was carefully designed to identify outliers and unusual parameters, but every test has its limitations and alerts that are not important in a particular case may appear. Conversely, the absence of alerts does not guarantee there are no aspects of the results needing attention. It is up to the individual to critically assess their own results and, if necessary, seek expert advice.

Publication of your CIF in IUCr journals

A basic structural check has been run on your CIF. These basic checks will be run on all CIFs submitted for publication in IUCr journals (*Acta Crystallographica*, *Journal of Applied Crystallography*, *Journal of Synchrotron Radiation*); however, if you intend to submit to *Acta Crystallographica Section C* or *E*, you should make sure that full publication checks are run on the final version of your CIF prior to submission.

Publication of your CIF in other journals

Please refer to the *Notes for Authors* of the relevant journal for any special instructions relating to CIF submission.

PLATON version of 19/11/2015; check.def file version of 17/11/2015

Datablock 1-Cr - ellipsoid plot

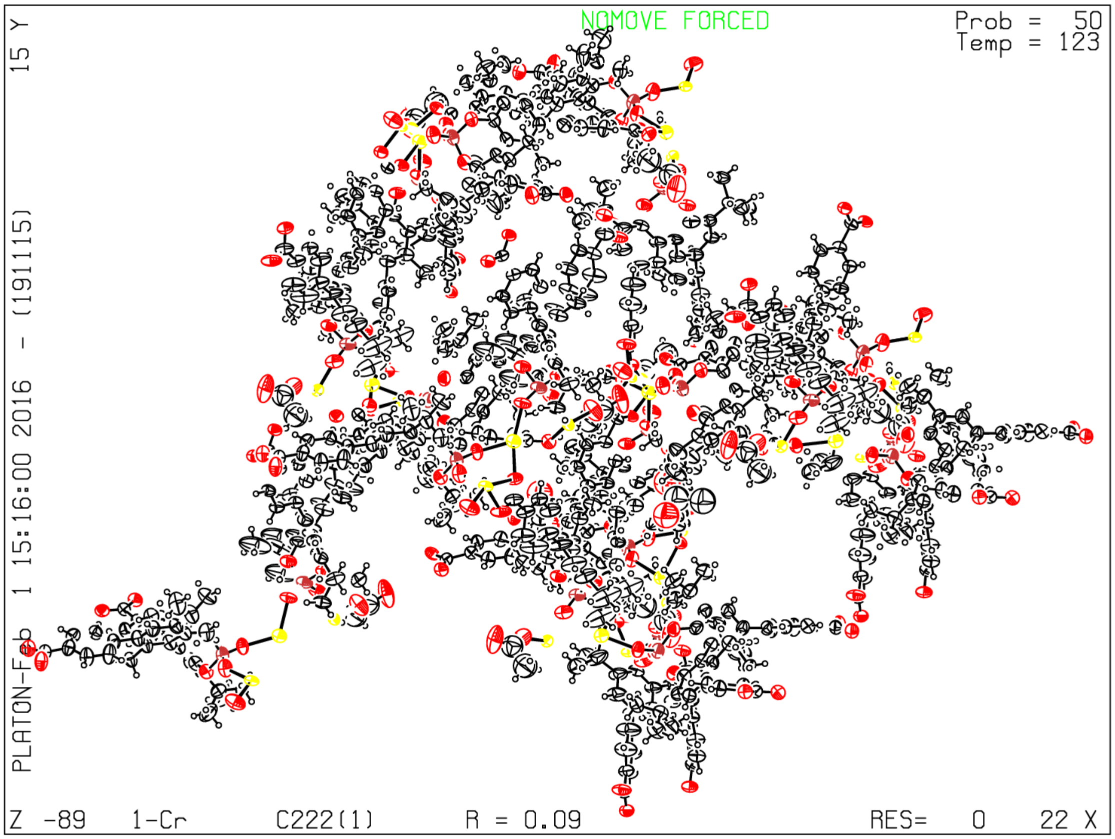

checkCIF/PLATON report

You have not supplied any structure factors. As a result the full set of tests cannot be run.

THIS REPORT IS FOR GUIDANCE ONLY. IF USED AS PART OF A REVIEW PROCEDURE FOR PUBLICATION, IT SHOULD NOT REPLACE THE EXPERTISE OF AN EXPERIENCED CRYSTALLOGRAPHIC REFEREE.

No syntax errors found. CIF dictionary Interpreting this report

Datablock: 1-Ga

```
Bond precision:  C-C = 0.0084 A          Wavelength=1.54178

Cell:            a=27.4162(7)     b=29.2521(8)     c=29.6153(7)
                 alpha=90         beta=90          gamma=90
Temperature:     123 K
```

	Calculated	Reported
Volume	23750.9(11)	23750.9(11)
Space group	C 2 2 21	C222(1)
Hall group	C 2c 2	?
Moiety formula	C74 H71 Ga3 O19 P2, 2(C2 O2)	?
Sum formula	C78 H71 Ga3 O23 P2	C78 H79 Ga3 O23 P2
Mr	1647.45	1655.51
Dx,g cm-3	0.922	0.926
Z	8	8
Mu (mm-1)	1.461	1.461
F000	6768.0	6832.0
F000’	6761.13	
h,k,lmax	33,35,35	32,34,35
Nref	21833[11617]	20356
Tmin,Tmax	0.900,0.916	0.900,0.916
Tmin’	0.890	

```
Correction method= # Reported T Limits: Tmin=0.900 Tmax=0.916
AbsCorr = MULTI-SCAN

Data completeness= 1.75/0.93      Theta(max)= 68.410

R(reflections)= 0.0851( 12961)    wR2(reflections)= 0.2563( 20356)

S = 1.101                  Npar= 787
```

The following ALERTS were generated. Each ALERT has the format
test-name_ALERT_alert-type_alert-level.
Click on the hyperlinks for more details of the test.

Alert level A

```
PLAT602_ALERT_2_A VERY LARGE Solvent Accessible VOID(S) in Structure        ! Info
```

Alert level B

```
PLAT035_ALERT_1_B _chemical_absolute_configuration info  Not given     Please Do !
PLAT049_ALERT_1_B Calculated Density less than 1.0 gcm-3 .........     0.9215 Check
PLAT213_ALERT_2_B Atom C17             has ADP max/min Ratio .....        4.2 prolat
PLAT220_ALERT_2_B Large Non-Solvent  C     Ueq(max)/Ueq(min) Range        6.1 Ratio
PLAT230_ALERT_2_B Hirshfeld Test Diff for    C61    --  C63     ..        9.0 s.u.
PLAT242_ALERT_2_B Low    'MainMol' Ueq as Compared to Neighbors of        C14 Check
PLAT430_ALERT_2_B Short Inter D...A Contact  O23    ..  O23     ..       2.58 Ang.
```

Alert level C

```
RFACR01_ALERT_3_C  The value of the weighted R factor is > 0.25
            Weighted R factor given   0.256
PLAT029_ALERT_3_C _diffrn_measured_fraction_theta_full Low .......      0.979 Note
PLAT041_ALERT_1_C Calc. and Reported SumFormula    Strings  Differ     Please Check
PLAT043_ALERT_1_C Calculated and Reported Mol. Weight Differ by ..       8.06 Check
PLAT068_ALERT_1_C Reported F000 Differs from Calcd (or Missing)...     Please Check
PLAT084_ALERT_3_C High wR2 Value (i.e. > 0.25) ..................       0.26 Report
PLAT202_ALERT_3_C Isotropic non-H Atoms in Anion/Solvent .........          8 Check
PLAT213_ALERT_2_C Atom O19             has ADP max/min Ratio .....        4.0 prolat
PLAT213_ALERT_2_C Atom C61             has ADP max/min Ratio .....        3.5 prolat
PLAT220_ALERT_2_C Large Non-Solvent  O     Ueq(max)/Ueq(min) Range        3.3 Ratio
PLAT222_ALERT_3_C Large Non-Solvent  H     Uiso(max)/Uiso(min) ...        6.5 Ratio
PLAT230_ALERT_2_C Hirshfeld Test Diff for    C21    --  C22     ..        5.8 s.u.
PLAT234_ALERT_4_C Large Hirshfeld Difference C25    --  C28     ..       0.16 Ang.
PLAT234_ALERT_4_C Large Hirshfeld Difference C32    --  C33     ..       0.16 Ang.
PLAT234_ALERT_4_C Large Hirshfeld Difference C39    --  C40     ..       0.16 Ang.
PLAT234_ALERT_4_C Large Hirshfeld Difference C59    --  C60     ..       0.16 Ang.
PLAT234_ALERT_4_C Large Hirshfeld Difference C61    --  C62     ..       0.20 Ang.
PLAT241_ALERT_2_C High   'MainMol' Ueq as Compared to Neighbors of        Ga2 Check
PLAT241_ALERT_2_C High   'MainMol' Ueq as Compared to Neighbors of         O5 Check
PLAT241_ALERT_2_C High   'MainMol' Ueq as Compared to Neighbors of         C6 Check
PLAT241_ALERT_2_C High   'MainMol' Ueq as Compared to Neighbors of        C40 Check
PLAT242_ALERT_2_C Low    'MainMol' Ueq as Compared to Neighbors of        Ga1 Check
PLAT242_ALERT_2_C Low    'MainMol' Ueq as Compared to Neighbors of        Ga3 Check
PLAT242_ALERT_2_C Low    'MainMol' Ueq as Compared to Neighbors of        C25 Check
PLAT242_ALERT_2_C Low    'MainMol' Ueq as Compared to Neighbors of        C50 Check
PLAT242_ALERT_2_C Low    'MainMol' Ueq as Compared to Neighbors of        C59 Check
PLAT242_ALERT_2_C Low    'MainMol' Ueq as Compared to Neighbors of        C61 Check
PLAT242_ALERT_2_C Low    'MainMol' Ueq as Compared to Neighbors of        C73 Check
PLAT244_ALERT_4_C Low    'Solvent' Ueq as Compared to Neighbors of        C75 Check
PLAT341_ALERT_3_C Low Bond Precision on  C-C Bonds ...............     0.0084 Ang.
PLAT367_ALERT_2_C Long?   C(sp?)-C(sp?) Bond  C75    -   C76    ..       1.57 Ang.
```

Alert level G

```
FORMU01_ALERT_2_G  There is a discrepancy between the atom counts in the
            _chemical_formula_sum and the formula from the _atom_site* data.
            Atom count from _chemical_formula_sum:C78 H79 Ga3 O23 P2
            Atom count from the _atom_site data:  C78 H71 Ga3 O23 P2
CELLZ01_ALERT_1_G Difference between formula and atom_site contents detected.
CELLZ01_ALERT_1_G WARNING: H atoms missing from atom site list. Is this intentional?
```

```
          From the CIF: _cell_formula_units_Z    8
          From the CIF: _chemical_formula_sum  C78 H79 Ga3 O23 P2
          TEST: Compare cell contents of formula and atom_site data

          atom    Z*formula  cif sites diff
          C        624.00    624.00    0.00
          H        632.00    568.00   64.00
          Ga        24.00     24.00    0.00
          O        184.00    184.00    0.00
          P         16.00     16.00    0.00
PLAT002_ALERT_2_G Number of Distance or Angle Restraints on AtSite         32 Note
PLAT004_ALERT_5_G Polymeric Structure Found with Maximum Dimension          3 Info
PLAT005_ALERT_5_G No Embedded Refinement Details found in the CIF      Please Do !
PLAT033_ALERT_4_G Flack x Value Deviates > 3.0 * sigma from Zero .     -0.090 Note
PLAT066_ALERT_1_G Predicted and Reported Tmin&Tmax Range Identical          ? Check
PLAT072_ALERT_2_G SHELXL First  Parameter in WGHT  Unusually Large       0.15 Report
PLAT232_ALERT_2_G Hirshfeld Test Diff (M-X)  Ga1    --  O5      ..        6.0 s.u.
PLAT232_ALERT_2_G Hirshfeld Test Diff (M-X)  Ga2    --  O6      ..       18.7 s.u.
PLAT232_ALERT_2_G Hirshfeld Test Diff (M-X)  Ga2    --  O14     ..       14.7 s.u.
PLAT232_ALERT_2_G Hirshfeld Test Diff (M-X)  Ga3    --  O13     ..        7.0 s.u.
PLAT344_ALERT_2_G Unusual sp?     Angle Range in Solvent/Ion for .     C76    Check
PLAT344_ALERT_2_G Unusual sp?     Angle Range in Solvent/Ion for .     C78    Check
PLAT380_ALERT_4_G Incorrectly? Oriented X(sp2)-Methyl Moiety .....        C18 Check
PLAT380_ALERT_4_G Incorrectly? Oriented X(sp2)-Methyl Moiety .....        C29 Check
PLAT380_ALERT_4_G Incorrectly? Oriented X(sp2)-Methyl Moiety .....        C54 Check
PLAT764_ALERT_4_G Overcomplete CIF Bond List Detected (Rep/Expd) .       1.10 Ratio
PLAT791_ALERT_4_G The Model has Chirality at P1      (Chiral SPGR)          R Verify
PLAT791_ALERT_4_G The Model has Chirality at P2      (Chiral SPGR)          S Verify
PLAT860_ALERT_3_G Number of Least-Squares Restraints .............         36 Note
PLAT899_ALERT_4_G SHELXL97   is Deprecated and Succeeded by SHELXL       2014 Note
```

---

1 **ALERT level A** = Most likely a serious problem - resolve or explain
7 **ALERT level B** = A potentially serious problem, consider carefully
31 **ALERT level C** = Check. Ensure it is not caused by an omission or oversight
23 **ALERT level G** = General information/check it is not something unexpected

8 ALERT type 1 CIF construction/syntax error, inconsistent or missing data
31 ALERT type 2 Indicator that the structure model may be wrong or deficient
7 ALERT type 3 Indicator that the structure quality may be low
14 ALERT type 4 Improvement, methodology, query or suggestion
2 ALERT type 5 Informative message, check

It is advisable to attempt to resolve as many as possible of the alerts in all categories. Often the minor alerts point to easily fixed oversights, errors and omissions in your CIF or refinement strategy, so attention to these fine details can be worthwhile. In order to resolve some of the more serious problems it may be necessary to carry out additional measurements or structure refinements. However, the purpose of your study may justify the reported deviations and the more serious of these should normally be commented upon in the discussion or experimental section of a paper or in the "special_details" fields of the CIF. checkCIF was carefully designed to identify outliers and unusual parameters, but every test has its limitations and alerts that are not important in a particular case may appear. Conversely, the absence of alerts does not guarantee there are no aspects of the results needing attention. It is up to the individual to critically assess their own results and, if necessary, seek expert advice.

Publication of your CIF in IUCr journals

A basic structural check has been run on your CIF. These basic checks will be run on all CIFs submitted for publication in IUCr journals (*Acta Crystallographica*, *Journal of Applied Crystallography*, *Journal of Synchrotron Radiation*); however, if you intend to submit to *Acta Crystallographica Section C* or *E*, you should make sure that full publication checks are run on the final version of your CIF prior to submission.

Publication of your CIF in other journals

Please refer to the *Notes for Authors* of the relevant journal for any special instructions relating to CIF submission.

PLATON version of 19/11/2015; check.def file version of 17/11/2015

Datablock 1-Ga - ellipsoid plot

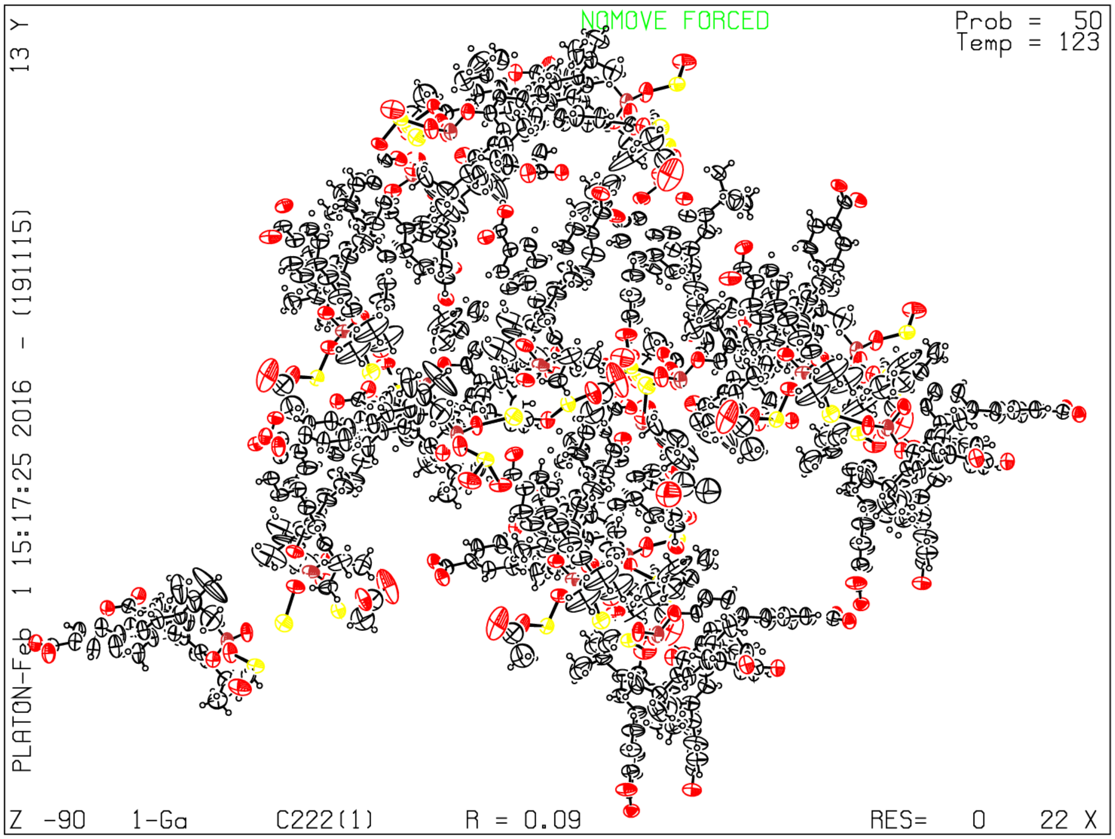

checkCIF/PLATON report

You have not supplied any structure factors. As a result the full set of tests cannot be run.

THIS REPORT IS FOR GUIDANCE ONLY. IF USED AS PART OF A REVIEW PROCEDURE FOR PUBLICATION, IT SHOULD NOT REPLACE THE EXPERTISE OF AN EXPERIENCED CRYSTALLOGRAPHIC REFEREE.

No syntax errors found. CIF dictionary Interpreting this report

Datablock: 1-Mn

```
Bond precision:  C-C = 0.0082 A          Wavelength=1.54178

Cell:           a=27.3307(12)     b=29.1106(12)     c=29.5760(12)
                alpha=90          beta=90           gamma=90
Temperature:    123 K

                Calculated                 Reported
Volume          23531.1(17)                23531.1(17)
Space group     C 2 2 21                   C222(1)
Hall group      C 2c 2                     ?
Moiety formula  C72 H68 Mn3 O18 P2         ?
Sum formula     C72 H68 Mn3 O18 P2         C72 H72 Mn3 O18 P2
Mr              1448.03                    1452.06
Dx,g cm-3       0.817                      0.820
Z               8                          8
Mu (mm-1)       3.180                      3.181
F000            5992.0                     6024.0
F000'           6001.34
h,k,lmax        30,32,33                   30,32,33
Nref            17495[ 9351]               17096
Tmin,Tmax       0.764,0.775                0.764,0.775
Tmin'           0.693

Correction method= # Reported T Limits: Tmin=0.764 Tmax=0.775
AbsCorr = MULTI-SCAN

Data completeness= 1.83/0.98      Theta(max)= 60.000

R(reflections)= 0.0944( 13248)    wR2(reflections)= 0.2672( 17096)

S = 1.050                  Npar= 760
```

The following ALERTS were generated. Each ALERT has the format
test-name_ALERT_alert-type_alert-level.
Click on the hyperlinks for more details of the test.

Alert level A

```
PLAT602_ALERT_2_A VERY LARGE Solvent Accessible VOID(S) in Structure        ! Info
```

Alert level B

```
THETM01_ALERT_3_B  The value of sine(theta_max)/wavelength is less than 0.575
            Calculated sin(theta_max)/wavelength =    0.5617
PLAT035_ALERT_1_B _chemical_absolute_configuration info  Not given     Please Do !
PLAT049_ALERT_1_B Calculated Density less than 1.0 gcm-3 .........     0.8175 Check
PLAT230_ALERT_2_B Hirshfeld Test Diff for    C14    --  C17     ..        7.2 s.u.
PLAT242_ALERT_2_B Low    'MainMol' Ueq as Compared to Neighbors of        Mn3 Check
```

Alert level C

```
RFACR01_ALERT_3_C  The value of the weighted R factor is > 0.25
            Weighted R factor given   0.267
STRVA01_ALERT_4_C           Flack test results are ambiguous.
           From the CIF: _refine_ls_abs_structure_Flack    0.358
           From the CIF: _refine_ls_abs_structure_Flack_su    0.007
PLAT041_ALERT_1_C Calc. and Reported SumFormula    Strings  Differ     Please Check
PLAT043_ALERT_1_C Calculated and Reported Mol. Weight Differ by ..       4.03 Check
PLAT068_ALERT_1_C Reported F000 Differs from Calcd (or Missing)...     Please Check
PLAT084_ALERT_3_C High wR2 Value (i.e. > 0.25) ...................       0.27 Report
PLAT213_ALERT_2_C Atom O18             has ADP max/min Ratio .....        3.1 prolat
PLAT220_ALERT_2_C Large Non-Solvent  C     Ueq(max)/Ueq(min) Range        3.9 Ratio
PLAT230_ALERT_2_C Hirshfeld Test Diff for    C20    --  C21     ..        6.1 s.u.
PLAT234_ALERT_4_C Large Hirshfeld Difference C2     --  C7      ..       0.16 Ang.
PLAT234_ALERT_4_C Large Hirshfeld Difference C61    --  C63     ..       0.18 Ang.
PLAT234_ALERT_4_C Large Hirshfeld Difference C61    --  C64     ..       0.23 Ang.
PLAT241_ALERT_2_C High   'MainMol' Ueq as Compared to Neighbors of         O5 Check
PLAT241_ALERT_2_C High   'MainMol' Ueq as Compared to Neighbors of        O13 Check
PLAT242_ALERT_2_C Low    'MainMol' Ueq as Compared to Neighbors of        Mn1 Check
PLAT242_ALERT_2_C Low    'MainMol' Ueq as Compared to Neighbors of        C14 Check
PLAT242_ALERT_2_C Low    'MainMol' Ueq as Compared to Neighbors of        C25 Check
PLAT242_ALERT_2_C Low    'MainMol' Ueq as Compared to Neighbors of        C61 Check
PLAT341_ALERT_3_C Low Bond Precision on  C-C Bonds ...............    0.00818 Ang.
PLAT369_ALERT_2_C Long    C(sp2)-C(sp2) Bond  C10    -   C19    ..       1.53 Ang.
```

Alert level G

```
FORMU01_ALERT_2_G  There is a discrepancy between the atom counts in the
            _chemical_formula_sum and the formula from the _atom_site* data.
            Atom count from _chemical_formula_sum:C72 H72 Mn3 O18 P2
            Atom count from the _atom_site data:  C72 H68 Mn3 O18 P2
CELLZ01_ALERT_1_G Difference between formula and atom_site contents detected.
CELLZ01_ALERT_1_G WARNING: H atoms missing from atom site list. Is this intentional?
           From the CIF: _cell_formula_units_Z    8
           From the CIF: _chemical_formula_sum  C72 H72 Mn3 O18 P2
           TEST: Compare cell contents of formula and atom_site data

           atom    Z*formula  cif sites diff
           C        576.00    576.00    0.00
           H        576.00    544.00   32.00
           Mn        24.00     24.00    0.00
           O        144.00    144.00    0.00
           P         16.00     16.00    0.00
PLAT002_ALERT_2_G Number of Distance or Angle Restraints on AtSite         20 Note
PLAT004_ALERT_5_G Polymeric Structure Found with Maximum Dimension          3 Info
PLAT005_ALERT_5_G No Embedded Refinement Details found in the CIF      Please Do !
PLAT033_ALERT_4_G Flack x Value Deviates > 3.0 * sigma from Zero .      0.358 Note
PLAT066_ALERT_1_G Predicted and Reported Tmin&Tmax Range Identical          ? Check
```

```
PLAT072_ALERT_2_G SHELXL First  Parameter in WGHT  Unusually Large       0.19 Report
PLAT232_ALERT_2_G Hirshfeld Test Diff (M-X)  Mn2    --  O6      ..        7.3 s.u.
PLAT232_ALERT_2_G Hirshfeld Test Diff (M-X)  Mn2    --  O14     ..        6.0 s.u.
PLAT232_ALERT_2_G Hirshfeld Test Diff (M-X)  Mn2    --  O2_h    ..        7.0 s.u.
PLAT380_ALERT_4_G Incorrectly? Oriented X(sp2)-Methyl Moiety .....        C18 Check
PLAT380_ALERT_4_G Incorrectly? Oriented X(sp2)-Methyl Moiety .....        C29 Check
PLAT380_ALERT_4_G Incorrectly? Oriented X(sp2)-Methyl Moiety .....        C65 Check
PLAT764_ALERT_4_G Overcomplete CIF Bond List Detected (Rep/Expd) .       1.11 Ratio
PLAT779_ALERT_4_G Suspect or Irrelevant (Bond) Angle in CIF .... #         75 Check
              O1   -C1   -MN3     1.555   1.555   8.556           44.30 Deg.
PLAT791_ALERT_4_G The Model has Chirality at P1      (Chiral SPGR)          S Verify
PLAT791_ALERT_4_G The Model has Chirality at P2      (Chiral SPGR)          R Verify
PLAT794_ALERT_5_G Tentative Bond Valency for Mn3     (I)     .....       0.72 Note
PLAT860_ALERT_3_G Number of Least-Squares Restraints .............         19 Note
PLAT899_ALERT_4_G SHELXL97   is Deprecated and Succeeded by SHELXL       2014 Note
```

```
   1 ALERT level A = Most likely a serious problem - resolve or explain
   5 ALERT level B = A potentially serious problem, consider carefully
  20 ALERT level C = Check. Ensure it is not caused by an omission or oversight
  22 ALERT level G = General information/check it is not something unexpected

   8 ALERT type 1 CIF construction/syntax error, inconsistent or missing data
  19 ALERT type 2 Indicator that the structure model may be wrong or deficient
   5 ALERT type 3 Indicator that the structure quality may be low
  13 ALERT type 4 Improvement, methodology, query or suggestion
   3 ALERT type 5 Informative message, check
```

It is advisable to attempt to resolve as many as possible of the alerts in all categories. Often the minor alerts point to easily fixed oversights, errors and omissions in your CIF or refinement strategy, so attention to these fine details can be worthwhile. In order to resolve some of the more serious problems it may be necessary to carry out additional measurements or structure refinements. However, the purpose of your study may justify the reported deviations and the more serious of these should normally be commented upon in the discussion or experimental section of a paper or in the "special_details" fields of the CIF. checkCIF was carefully designed to identify outliers and unusual parameters, but every test has its limitations and alerts that are not important in a particular case may appear. Conversely, the absence of alerts does not guarantee there are no aspects of the results needing attention. It is up to the individual to critically assess their own results and, if necessary, seek expert advice.

Publication of your CIF in IUCr journals

A basic structural check has been run on your CIF. These basic checks will be run on all CIFs submitted for publication in IUCr journals (*Acta Crystallographica*, *Journal of Applied Crystallography*, *Journal of Synchrotron Radiation*); however, if you intend to submit to *Acta Crystallographica Section C* or *E*, you should make sure that full publication checks are run on the final version of your CIF prior to submission.

Publication of your CIF in other journals

Please refer to the *Notes for Authors* of the relevant journal for any special instructions relating to CIF submission.

PLATON version of 19/11/2015; check.def file version of 17/11/2015

Datablock 1-Mn - ellipsoid plot

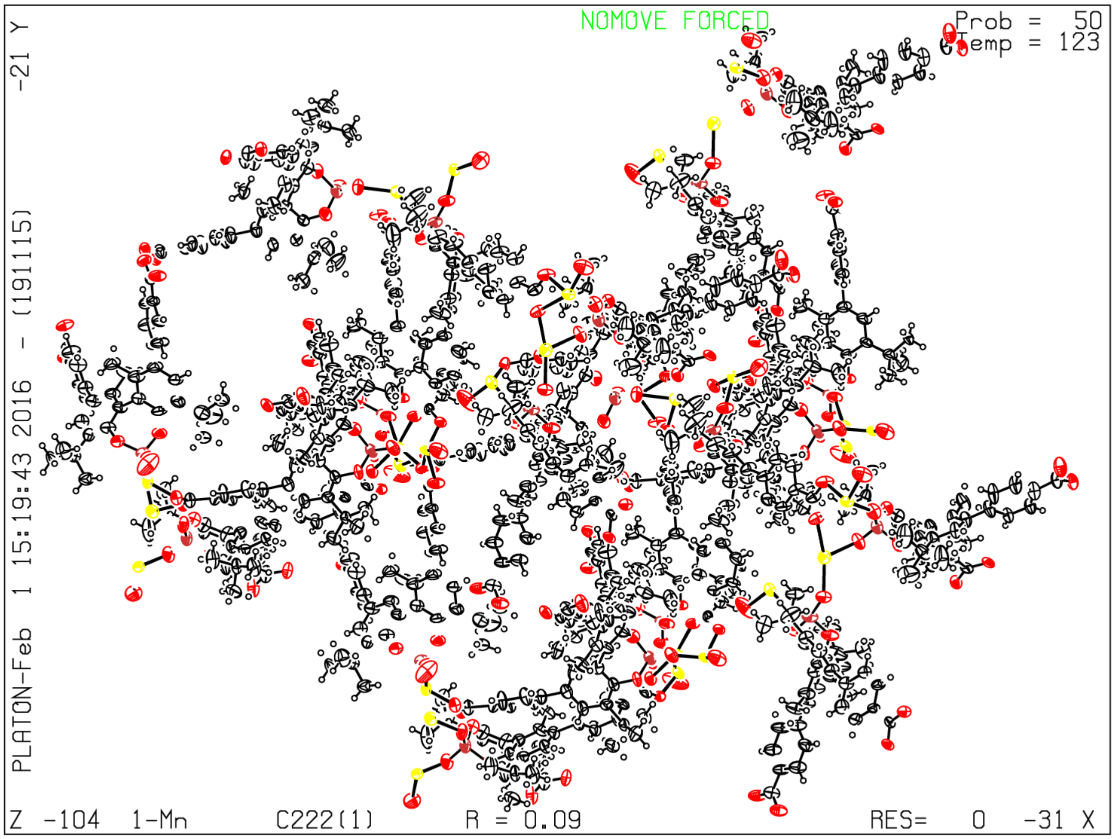

checkCIF/PLATON report

You have not supplied any structure factors. As a result the full set of tests cannot be run.

THIS REPORT IS FOR GUIDANCE ONLY. IF USED AS PART OF A REVIEW PROCEDURE FOR PUBLICATION, IT SHOULD NOT REPLACE THE EXPERTISE OF AN EXPERIENCED CRYSTALLOGRAPHIC REFEREE.

No syntax errors found. CIF dictionary Interpreting this report

Datablock: 1-Ti

Bond precision: C-C = 0.0146 A Wavelength=0.71073

Cell:	a=27.341(9)	b=29.274(11)	c=29.662(10)
	alpha=90	beta=90	gamma=90
Temperature:	123 K		

	Calculated	Reported
Volume	23741(14)	23741(14)
Space group	C 2 2 21	C222(1)
Hall group	C 2c 2	?
Moiety formula	C74 H71 O19 P2 Ti3, 0.5(C4 O4), C2 O2, 3(O)	?
Sum formula	C78 H71 O26 P2 Ti3	C78 H82 O26 P2 Ti3
Mr	1629.90	1641.08
Dx,g cm-3	0.912	0.918
Z	8	8
Mu (mm-1)	0.276	0.276
F000	6744.0	6832.0
F000’	6755.85	
h,k,lmax	32,34,35	32,34,35
Nref	20926[11142]	20885
Tmin,Tmax	0.978,0.984	0.978,0.984
Tmin’	0.978	

Correction method= # Reported T Limits: Tmin=0.978 Tmax=0.984
AbsCorr = ?

Data completeness= 1.87/1.00 Theta(max)= 25.000

R(reflections)= 0.0941(6479) wR2(reflections)= 0.2130(20885)

S = 1.020 Npar= 797

The following ALERTS were generated. Each ALERT has the format
test-name_ALERT_alert-type_alert-level.
Click on the hyperlinks for more details of the test.

Alert level A

```
PLAT602_ALERT_2_A VERY LARGE Solvent Accessible VOID(S) in Structure        ! Info
```

Alert level B

```
RINTA01_ALERT_3_B  The value of Rint is greater than 0.18
            Rint given   0.182
PLAT020_ALERT_3_B The value of Rint is greater than 0.12 .........      0.182 Report
PLAT026_ALERT_3_B Ratio Observed / Unique Reflections (too) Low ..         31 %
PLAT035_ALERT_1_B _chemical_absolute_configuration info  Not given     Please Do !
PLAT049_ALERT_1_B Calculated Density less than 1.0 gcm-3 .........     0.9120 Check
PLAT213_ALERT_2_B Atom O17              has ADP max/min Ratio .....       4.9 oblate
PLAT213_ALERT_2_B Atom C13              has ADP max/min Ratio .....       4.3 prolat
PLAT213_ALERT_2_B Atom C58              has ADP max/min Ratio .....       4.1 prolat
PLAT213_ALERT_2_B Atom C67              has ADP max/min Ratio .....       4.6 prolat
PLAT230_ALERT_2_B Hirshfeld Test Diff for    C2     --  C7      ..        7.4 s.u.
PLAT230_ALERT_2_B Hirshfeld Test Diff for    C8     --  C13     ..       12.3 s.u.
PLAT230_ALERT_2_B Hirshfeld Test Diff for    C9     --  C18     ..       10.9 s.u.
PLAT230_ALERT_2_B Hirshfeld Test Diff for    C10    --  C19     ..        8.0 s.u.
PLAT230_ALERT_2_B Hirshfeld Test Diff for    C12    --  C14     ..        9.5 s.u.
PLAT230_ALERT_2_B Hirshfeld Test Diff for    C20    --  C29     ..        9.3 s.u.
PLAT230_ALERT_2_B Hirshfeld Test Diff for    C25    --  C28     ..       17.7 s.u.
PLAT230_ALERT_2_B Hirshfeld Test Diff for    C59    --  C61     ..        9.5 s.u.
PLAT230_ALERT_2_B Hirshfeld Test Diff for    C61    --  C62     ..        9.0 s.u.
PLAT230_ALERT_2_B Hirshfeld Test Diff for    C61    --  C63     ..       19.7 s.u.
PLAT230_ALERT_2_B Hirshfeld Test Diff for    C61    --  C64     ..       12.0 s.u.
PLAT232_ALERT_2_B Hirshfeld Test Diff (M-X)  Ti1    --  O5      ..       17.7 s.u.
PLAT232_ALERT_2_B Hirshfeld Test Diff (M-X)  Ti3    --  O7_b    ..       15.2 s.u.
PLAT234_ALERT_4_B Large Hirshfeld Difference C5     --  C6      ..       0.28 Ang.
PLAT234_ALERT_4_B Large Hirshfeld Difference C5     --  C8      ..       0.30 Ang.
PLAT234_ALERT_4_B Large Hirshfeld Difference C8     --  C9      ..       0.26 Ang.
PLAT234_ALERT_4_B Large Hirshfeld Difference C14    --  C15     ..       0.28 Ang.
PLAT234_ALERT_4_B Large Hirshfeld Difference C25    --  C26     ..       0.30 Ang.
PLAT234_ALERT_4_B Large Hirshfeld Difference C41    --  C44     ..       0.26 Ang.
PLAT234_ALERT_4_B Large Hirshfeld Difference C67    --  C68     ..       0.28 Ang.
PLAT241_ALERT_2_B High   'MainMol' Ueq as Compared to Neighbors of        O5 Check
PLAT241_ALERT_2_B High   'MainMol' Ueq as Compared to Neighbors of       O18 Check
PLAT241_ALERT_2_B High   'MainMol' Ueq as Compared to Neighbors of       C58 Check
PLAT241_ALERT_2_B High   'MainMol' Ueq as Compared to Neighbors of       C71 Check
PLAT242_ALERT_2_B Low    'MainMol' Ueq as Compared to Neighbors of       Ti1 Check
PLAT242_ALERT_2_B Low    'MainMol' Ueq as Compared to Neighbors of       Ti3 Check
PLAT242_ALERT_2_B Low    'MainMol' Ueq as Compared to Neighbors of        C5 Check
PLAT242_ALERT_2_B Low    'MainMol' Ueq as Compared to Neighbors of       C14 Check
PLAT242_ALERT_2_B Low    'MainMol' Ueq as Compared to Neighbors of       C25 Check
PLAT242_ALERT_2_B Low    'MainMol' Ueq as Compared to Neighbors of       C59 Check
PLAT242_ALERT_2_B Low    'MainMol' Ueq as Compared to Neighbors of       C61 Check
PLAT306_ALERT_2_B Isolated Oxygen Atom (H-atoms Missing ?) .......       O24 Check
PLAT306_ALERT_2_B Isolated Oxygen Atom (H-atoms Missing ?) .......       O25 Check
PLAT306_ALERT_2_B Isolated Oxygen Atom (H-atoms Missing ?) .......       O26 Check
PLAT412_ALERT_2_B Short Intra XH3 .. XHn     H62B   ..  H64C    ..       1.72 Ang.
PLAT430_ALERT_2_B Short Inter D...A Contact  O17    ..  O25     ..       2.78 Ang.
```

Alert level C

```
STRVA01_ALERT_4_C           Flack parameter is too small
           From the CIF: _refine_ls_abs_structure_Flack   -0.270
```

```
          From the CIF: _refine_ls_abs_structure_Flack_su    0.040
PLAT041_ALERT_1_C Calc. and Reported SumFormula    Strings  Differ     Please Check
PLAT043_ALERT_1_C Calculated and Reported Mol. Weight Differ by ..      11.18 Check
PLAT052_ALERT_1_C Info on Absorption Correction Method   Not Given     Please Do !
PLAT068_ALERT_1_C Reported F000 Differs from Calcd (or Missing)...     Please Check
PLAT202_ALERT_3_C Isotropic non-H Atoms in Anion/Solvent .........         11 Check
PLAT213_ALERT_2_C Atom O8             has ADP max/min Ratio .....        3.2 oblate
PLAT213_ALERT_2_C Atom O18            has ADP max/min Ratio .....        3.2 oblate
PLAT213_ALERT_2_C Atom C15            has ADP max/min Ratio .....        3.2 prolat
PLAT213_ALERT_2_C Atom C27            has ADP max/min Ratio .....        4.0 prolat
PLAT213_ALERT_2_C Atom C28            has ADP max/min Ratio .....        3.3 prolat
PLAT213_ALERT_2_C Atom C35            has ADP max/min Ratio .....        4.0 prolat
PLAT213_ALERT_2_C Atom C62            has ADP max/min Ratio .....        3.2 prolat
PLAT213_ALERT_2_C Atom C63            has ADP max/min Ratio .....        3.2 prolat
PLAT213_ALERT_2_C Atom C64            has ADP max/min Ratio .....        3.2 prolat
PLAT213_ALERT_2_C Atom C69            has ADP max/min Ratio .....        3.7 prolat
PLAT213_ALERT_2_C Atom C72            has ADP max/min Ratio .....        3.2 oblate
PLAT220_ALERT_2_C Large Non-Solvent  C     Ueq(max)/Ueq(min) Range        5.0 Ratio
PLAT222_ALERT_3_C Large Non-Solvent  H     Uiso(max)/Uiso(min) ...        5.4 Ratio
PLAT230_ALERT_2_C Hirshfeld Test Diff for    P1     --  O5      ..        5.4 s.u.
PLAT230_ALERT_2_C Hirshfeld Test Diff for    P2     --  O11     ..        5.7 s.u.
PLAT230_ALERT_2_C Hirshfeld Test Diff for    O9     --  C37     ..        5.5 s.u.
PLAT230_ALERT_2_C Hirshfeld Test Diff for    C14    --  C16     ..        5.3 s.u.
PLAT230_ALERT_2_C Hirshfeld Test Diff for    C19    --  C24     ..        6.1 s.u.
PLAT230_ALERT_2_C Hirshfeld Test Diff for    C30    --  C35     ..        5.1 s.u.
PLAT230_ALERT_2_C Hirshfeld Test Diff for    C48    --  C49     ..        6.7 s.u.
PLAT230_ALERT_2_C Hirshfeld Test Diff for    C50    --  C51     ..        7.0 s.u.
PLAT230_ALERT_2_C Hirshfeld Test Diff for    C66    --  C71     ..        5.4 s.u.
PLAT232_ALERT_2_C Hirshfeld Test Diff (M-X)  Ti1    --  O9_c    ..        8.3 s.u.
PLAT232_ALERT_2_C Hirshfeld Test Diff (M-X)  Ti1    --  O16_g   ..        8.0 s.u.
PLAT232_ALERT_2_C Hirshfeld Test Diff (M-X)  Ti3    --  O13     ..        7.0 s.u.
PLAT232_ALERT_2_C Hirshfeld Test Diff (M-X)  Ti3    --  O1_i    ..        6.0 s.u.
PLAT234_ALERT_4_C Large Hirshfeld Difference Ti2    --  O2_i    ..       0.16 Ang.
PLAT234_ALERT_4_C Large Hirshfeld Difference Ti3    --  O18     ..       0.17 Ang.
PLAT234_ALERT_4_C Large Hirshfeld Difference O2     --  C1      ..       0.16 Ang.
PLAT234_ALERT_4_C Large Hirshfeld Difference O11    --  C47     ..       0.17 Ang.
PLAT234_ALERT_4_C Large Hirshfeld Difference O15    --  C72     ..       0.24 Ang.
PLAT234_ALERT_4_C Large Hirshfeld Difference C1     --  C2      ..       0.24 Ang.
PLAT234_ALERT_4_C Large Hirshfeld Difference C2     --  C3      ..       0.21 Ang.
PLAT234_ALERT_4_C Large Hirshfeld Difference C3     --  C4      ..       0.20 Ang.
PLAT234_ALERT_4_C Large Hirshfeld Difference C4     --  C5      ..       0.21 Ang.
PLAT234_ALERT_4_C Large Hirshfeld Difference C10    --  C11     ..       0.17 Ang.
PLAT234_ALERT_4_C Large Hirshfeld Difference C20    --  C21     ..       0.16 Ang.
PLAT234_ALERT_4_C Large Hirshfeld Difference C22    --  C23     ..       0.17 Ang.
PLAT234_ALERT_4_C Large Hirshfeld Difference C30    --  C31     ..       0.17 Ang.
PLAT234_ALERT_4_C Large Hirshfeld Difference C32    --  C33     ..       0.22 Ang.
PLAT234_ALERT_4_C Large Hirshfeld Difference C41    --  C42     ..       0.21 Ang.
PLAT234_ALERT_4_C Large Hirshfeld Difference C44    --  C45     ..       0.19 Ang.
PLAT234_ALERT_4_C Large Hirshfeld Difference C45    --  C54     ..       0.21 Ang.
PLAT234_ALERT_4_C Large Hirshfeld Difference C46    --  C47     ..       0.23 Ang.
PLAT234_ALERT_4_C Large Hirshfeld Difference C48    --  C50     ..       0.24 Ang.
PLAT234_ALERT_4_C Large Hirshfeld Difference C56    --  C57     ..       0.23 Ang.
PLAT234_ALERT_4_C Large Hirshfeld Difference C59    --  C60     ..       0.21 Ang.
PLAT234_ALERT_4_C Large Hirshfeld Difference C66    --  C67     ..       0.24 Ang.
PLAT234_ALERT_4_C Large Hirshfeld Difference C69    --  C70     ..       0.25 Ang.
PLAT234_ALERT_4_C Large Hirshfeld Difference C69    --  C72     ..       0.25 Ang.
PLAT241_ALERT_2_C High   'MainMol' Ueq as Compared to Neighbors of         O6 Check
PLAT241_ALERT_2_C High   'MainMol' Ueq as Compared to Neighbors of        O13 Check
PLAT241_ALERT_2_C High   'MainMol' Ueq as Compared to Neighbors of        O14 Check
PLAT241_ALERT_2_C High   'MainMol' Ueq as Compared to Neighbors of         C3 Check
PLAT241_ALERT_2_C High   'MainMol' Ueq as Compared to Neighbors of         C6 Check
PLAT241_ALERT_2_C High   'MainMol' Ueq as Compared to Neighbors of         C8 Check
```

```
PLAT241_ALERT_2_C High   'MainMol' Ueq as Compared to Neighbors of        C13 Check
PLAT241_ALERT_2_C High   'MainMol' Ueq as Compared to Neighbors of        C22 Check
PLAT241_ALERT_2_C High   'MainMol' Ueq as Compared to Neighbors of        C32 Check
PLAT241_ALERT_2_C High   'MainMol' Ueq as Compared to Neighbors of        C35 Check
PLAT241_ALERT_2_C High   'MainMol' Ueq as Compared to Neighbors of        C37 Check
PLAT241_ALERT_2_C High   'MainMol' Ueq as Compared to Neighbors of        C40 Check
PLAT241_ALERT_2_C High   'MainMol' Ueq as Compared to Neighbors of        C43 Check
PLAT241_ALERT_2_C High   'MainMol' Ueq as Compared to Neighbors of        C67 Check
PLAT242_ALERT_2_C Low    'MainMol' Ueq as Compared to Neighbors of        Ti2 Check
PLAT242_ALERT_2_C Low    'MainMol' Ueq as Compared to Neighbors of         O4 Check
PLAT242_ALERT_2_C Low    'MainMol' Ueq as Compared to Neighbors of         C2 Check
PLAT242_ALERT_2_C Low    'MainMol' Ueq as Compared to Neighbors of         C9 Check
PLAT242_ALERT_2_C Low    'MainMol' Ueq as Compared to Neighbors of        C12 Check
PLAT242_ALERT_2_C Low    'MainMol' Ueq as Compared to Neighbors of        C20 Check
PLAT242_ALERT_2_C Low    'MainMol' Ueq as Compared to Neighbors of        C23 Check
PLAT242_ALERT_2_C Low    'MainMol' Ueq as Compared to Neighbors of        C33 Check
PLAT242_ALERT_2_C Low    'MainMol' Ueq as Compared to Neighbors of        C36 Check
PLAT242_ALERT_2_C Low    'MainMol' Ueq as Compared to Neighbors of        C38 Check
PLAT242_ALERT_2_C Low    'MainMol' Ueq as Compared to Neighbors of        C42 Check
PLAT242_ALERT_2_C Low    'MainMol' Ueq as Compared to Neighbors of        C45 Check
PLAT242_ALERT_2_C Low    'MainMol' Ueq as Compared to Neighbors of        C47 Check
PLAT242_ALERT_2_C Low    'MainMol' Ueq as Compared to Neighbors of        C50 Check
PLAT242_ALERT_2_C Low    'MainMol' Ueq as Compared to Neighbors of        C56 Check
PLAT242_ALERT_2_C Low    'MainMol' Ueq as Compared to Neighbors of        C57 Check
PLAT242_ALERT_2_C Low    'MainMol' Ueq as Compared to Neighbors of        C66 Check
PLAT242_ALERT_2_C Low    'MainMol' Ueq as Compared to Neighbors of        C70 Check
PLAT242_ALERT_2_C Low    'MainMol' Ueq as Compared to Neighbors of        C73 Check
PLAT244_ALERT_4_C Low    'Solvent' Ueq as Compared to Neighbors of        C75 Check
PLAT250_ALERT_2_C Large U3/U1 Ratio for Average U(i,j) Tensor ....        2.2 Note
PLAT309_ALERT_2_C Single Bonded Oxygen (C-O > 1.3 Ang) ..........         O20 Check
PLAT341_ALERT_3_C Low Bond Precision on  C-C Bonds ...............    0.01457 Ang.
PLAT363_ALERT_2_C Long    C(sp3)-C(sp2) Bond  C56    -   C65    ..       1.63 Ang.
PLAT367_ALERT_2_C Long?   C(sp?)-C(sp?) Bond  C75    -   C76    ..       1.58 Ang.
PLAT367_ALERT_2_C Long?   C(sp?)-C(sp?) Bond  C76    -   C76_j  ..       1.65 Ang.
PLAT369_ALERT_2_C Long    C(sp2)-C(sp2) Bond  C10    -   C19    ..       1.55 Ang.
PLAT369_ALERT_2_C Long    C(sp2)-C(sp2) Bond  C46    -   C55    ..       1.53 Ang.
```

Alert level G

```
FORMU01_ALERT_2_G  There is a discrepancy between the atom counts in the
            _chemical_formula_sum and the formula from the _atom_site* data.
            Atom count from _chemical_formula_sum:C78 H82 O26 P2 Ti3
            Atom count from the _atom_site data:  C78 H71 O26 P2 Ti3
CELLZ01_ALERT_1_G Difference between formula and atom_site contents detected.
CELLZ01_ALERT_1_G WARNING: H atoms missing from atom site list. Is this intentional?
           From the CIF: _cell_formula_units_Z    8
           From the CIF: _chemical_formula_sum  C78 H82 O26 P2 Ti3
           TEST: Compare cell contents of formula and atom_site data

           atom    Z*formula  cif sites diff
           C        624.00    624.00    0.00
           H        656.00    568.00   88.00
           O        208.00    208.00    0.00
           P         16.00     16.00    0.00
           Ti        24.00     24.00    0.00
PLAT002_ALERT_2_G Number of Distance or Angle Restraints on AtSite         32 Note
PLAT003_ALERT_2_G Number of Uiso or Uij Restrained non-H Atoms ...          2 Report
PLAT004_ALERT_5_G Polymeric Structure Found with Maximum Dimension          3 Info
PLAT005_ALERT_5_G No Embedded Refinement Details found in the CIF      Please Do !
PLAT033_ALERT_4_G Flack x Value Deviates > 3.0 * sigma from Zero .     -0.270 Note
PLAT066_ALERT_1_G Predicted and Reported Tmin&Tmax Range Identical          ? Check
```

```
PLAT343_ALERT_2_G Unusual sp3      Angle Range in Main Residue for     C61    Check
PLAT344_ALERT_2_G Unusual sp?     Angle Range in Solvent/Ion for .     C76    Check
PLAT344_ALERT_2_G Unusual sp?     Angle Range in Solvent/Ion for .     C78    Check
PLAT380_ALERT_4_G Incorrectly? Oriented X(sp2)-Methyl Moiety .....        C29 Check
PLAT380_ALERT_4_G Incorrectly? Oriented X(sp2)-Methyl Moiety .....        C54 Check
PLAT380_ALERT_4_G Incorrectly? Oriented X(sp2)-Methyl Moiety .....        C74 Check
PLAT764_ALERT_4_G Overcomplete CIF Bond List Detected (Rep/Expd) .       1.11 Ratio
PLAT779_ALERT_4_G Suspect or Irrelevant (Bond) Angle in CIF .... #         75 Check
              O1   -C1   -TI3     1.555   1.555   8.645            42.30 Deg.
PLAT791_ALERT_4_G The Model has Chirality at P1      (Chiral SPGR)         S Verify
PLAT791_ALERT_4_G The Model has Chirality at P2      (Chiral SPGR)         R Verify
PLAT860_ALERT_3_G Number of Least-Squares Restraints .............         46 Note
PLAT899_ALERT_4_G SHELXL97   is Deprecated and Succeeded by SHELXL       2014 Note
```

```
  1 ALERT level A = Most likely a serious problem - resolve or explain
 45 ALERT level B = A potentially serious problem, consider carefully
 98 ALERT level C = Check. Ensure it is not caused by an omission or oversight
 21 ALERT level G = General information/check it is not something unexpected

  9 ALERT type 1 CIF construction/syntax error, inconsistent or missing data
105 ALERT type 2 Indicator that the structure model may be wrong or deficient
  7 ALERT type 3 Indicator that the structure quality may be low
 42 ALERT type 4 Improvement, methodology, query or suggestion
  2 ALERT type 5 Informative message, check
```

It is advisable to attempt to resolve as many as possible of the alerts in all categories. Often the minor alerts point to easily fixed oversights, errors and omissions in your CIF or refinement strategy, so attention to these fine details can be worthwhile. In order to resolve some of the more serious problems it may be necessary to carry out additional measurements or structure refinements. However, the purpose of your study may justify the reported deviations and the more serious of these should normally be commented upon in the discussion or experimental section of a paper or in the "special_details" fields of the CIF. checkCIF was carefully designed to identify outliers and unusual parameters, but every test has its limitations and alerts that are not important in a particular case may appear. Conversely, the absence of alerts does not guarantee there are no aspects of the results needing attention. It is up to the individual to critically assess their own results and, if necessary, seek expert advice.

Publication of your CIF in IUCr journals

A basic structural check has been run on your CIF. These basic checks will be run on all CIFs submitted for publication in IUCr journals (*Acta Crystallographica*, *Journal of Applied Crystallography*, *Journal of Synchrotron Radiation*); however, if you intend to submit to *Acta Crystallographica Section C* or *E*, you should make sure that full publication checks are run on the final version of your CIF prior to submission.

Publication of your CIF in other journals

Please refer to the *Notes for Authors* of the relevant journal for any special instructions relating to CIF submission.

PLATON version of 19/11/2015; check.def file version of 17/11/2015

Datablock 1-Ti - ellipsoid plot

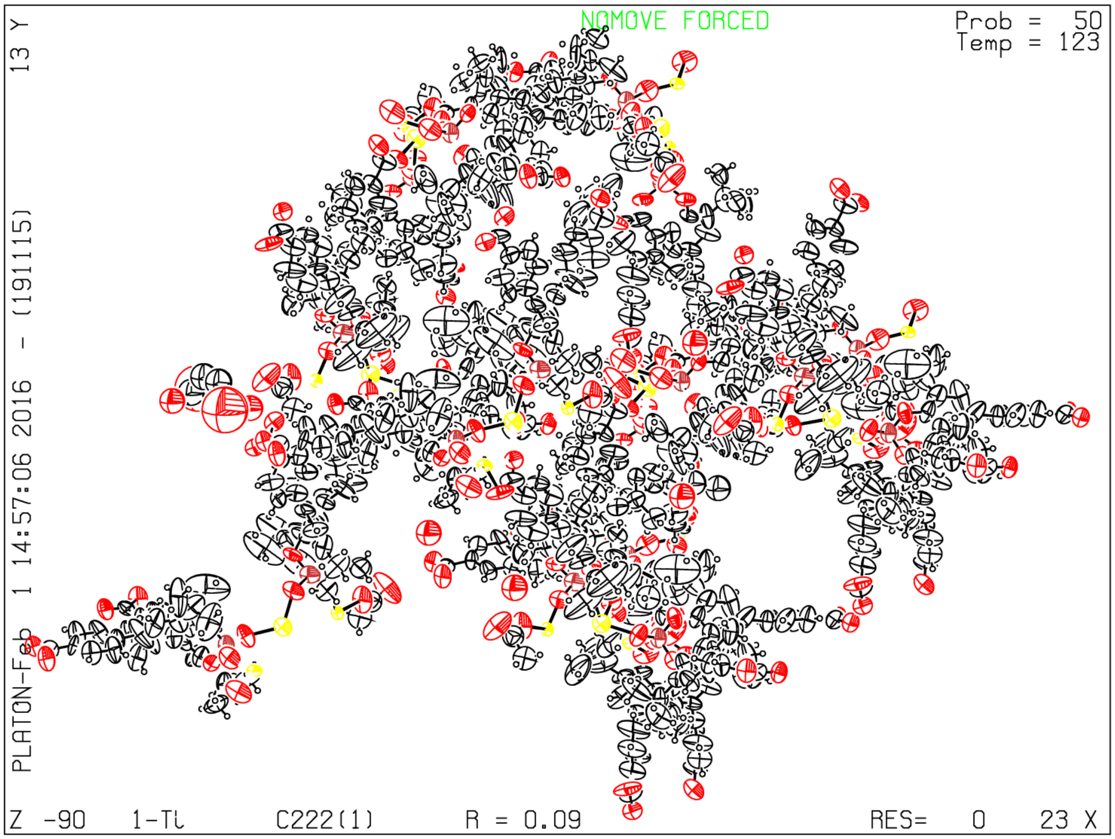

checkCIF/PLATON report

You have not supplied any structure factors. As a result the full set of tests cannot be run.

THIS REPORT IS FOR GUIDANCE ONLY. IF USED AS PART OF A REVIEW PROCEDURE FOR PUBLICATION, IT SHOULD NOT REPLACE THE EXPERTISE OF AN EXPERIENCED CRYSTALLOGRAPHIC REFEREE.

No syntax errors found. CIF dictionary Interpreting this report

Datablock: 1-Zr

```
Bond precision:  C-C = 0.0088 A          Wavelength=1.54178

Cell:            a=27.0284(6)     b=29.1892(6)     c=29.5728(6)
                 alpha=90         beta=90          gamma=90
Temperature:     123 K
```

	Calculated	Reported
Volume	23331.1(8)	23331.1(8)
Space group	C 2 2 21	C222(1)
Hall group	C 2c 2	?
Moiety formula	C74 H71 O19 P2 Zr3, 0.5(C4 O4), C2 O2, 3(O)	?
Sum formula	C78 H71 O26 P2 Zr3	C78 H82 O26 P2 Zr3
Mr	1759.95	1771.04
Dx,g cm-3	1.002	1.008
Z	8	8
Mu (mm-1)	2.867	2.868
F000	7176.0	7264.0
F000’	7196.74	
h,k,lmax	28,30,31	28,30,31
Nref	14670[7871]	13060
Tmin,Tmax	0.759,0.795	0.759,0.795
Tmin’	0.751	

```
Correction method= # Reported T Limits: Tmin=0.759 Tmax=0.795
AbsCorr = MULTI-SCAN

Data completeness= 1.66/0.89      Theta(max)= 55.000

R(reflections)= 0.0919( 12258)    wR2(reflections)= 0.2527( 13060)

S = 1.183                  Npar= 815
```

The following ALERTS were generated. Each ALERT has the format
test-name_ALERT_alert-type_alert-level.
Click on the hyperlinks for more details of the test.

Alert level A

```
THETM01_ALERT_3_A  The value of sine(theta_max)/wavelength is less than 0.550
            Calculated sin(theta_max)/wavelength =    0.5313
PLAT602_ALERT_2_A VERY LARGE Solvent Accessible VOID(S) in Structure        ! Info
```

Alert level B

```
PLAT035_ALERT_1_B _chemical_absolute_configuration info  Not given     Please Do !
PLAT213_ALERT_2_B Atom C61             has ADP max/min Ratio .....        5.0 prolat
PLAT220_ALERT_2_B Large Non-Solvent  C     Ueq(max)/Ueq(min) Range        7.1 Ratio
PLAT222_ALERT_3_B Large Non-Solvent  H     Uiso(max)/Uiso(min) ...        7.6 Ratio
PLAT230_ALERT_2_B Hirshfeld Test Diff for    O8     --  C36     ..        7.2 s.u.
PLAT230_ALERT_2_B Hirshfeld Test Diff for    O9     --  C37     ..        7.2 s.u.
PLAT230_ALERT_2_B Hirshfeld Test Diff for    O10    --  C37     ..        8.0 s.u.
PLAT241_ALERT_2_B High   'MainMol' Ueq as Compared to Neighbors of        Zr2 Check
PLAT306_ALERT_2_B Isolated Oxygen Atom (H-atoms Missing ?) .......        O24 Check
PLAT306_ALERT_2_B Isolated Oxygen Atom (H-atoms Missing ?) .......        O25 Check
PLAT306_ALERT_2_B Isolated Oxygen Atom (H-atoms Missing ?) .......        O26 Check
```

Alert level C

```
RFACR01_ALERT_3_C  The value of the weighted R factor is > 0.25
            Weighted R factor given   0.253
PLAT029_ALERT_3_C _diffrn_measured_fraction_theta_full Low .......      0.970 Note
PLAT041_ALERT_1_C Calc. and Reported SumFormula    Strings  Differ     Please Check
PLAT043_ALERT_1_C Calculated and Reported Mol. Weight Differ by ..      11.09 Check
PLAT068_ALERT_1_C Reported F000 Differs from Calcd (or Missing)...     Please Check
PLAT202_ALERT_3_C Isotropic non-H Atoms in Anion/Solvent .........         11 Check
PLAT213_ALERT_2_C Atom O10             has ADP max/min Ratio .....        3.3 oblate
PLAT213_ALERT_2_C Atom O15             has ADP max/min Ratio .....        3.1 prolat
PLAT213_ALERT_2_C Atom O17             has ADP max/min Ratio .....        3.3 prolat
PLAT213_ALERT_2_C Atom O18             has ADP max/min Ratio .....        3.7 prolat
PLAT213_ALERT_2_C Atom O19             has ADP max/min Ratio .....        3.2 oblate
PLAT213_ALERT_2_C Atom C7              has ADP max/min Ratio .....        3.4 prolat
PLAT213_ALERT_2_C Atom C14             has ADP max/min Ratio .....        3.1 prolat
PLAT213_ALERT_2_C Atom C59             has ADP max/min Ratio .....        4.0 prolat
PLAT213_ALERT_2_C Atom C64             has ADP max/min Ratio .....        3.5 prolat
PLAT213_ALERT_2_C Atom C66             has ADP max/min Ratio .....        3.7 prolat
PLAT213_ALERT_2_C Atom C67             has ADP max/min Ratio .....        3.1 prolat
PLAT213_ALERT_2_C Atom C72             has ADP max/min Ratio .....        3.2 prolat
PLAT230_ALERT_2_C Hirshfeld Test Diff for    O2     --  C1      ..        7.0 s.u.
PLAT230_ALERT_2_C Hirshfeld Test Diff for    O7     --  C36     ..        5.2 s.u.
PLAT230_ALERT_2_C Hirshfeld Test Diff for    C14    --  C16     ..        6.9 s.u.
PLAT230_ALERT_2_C Hirshfeld Test Diff for    C46    --  C47     ..        5.2 s.u.
PLAT230_ALERT_2_C Hirshfeld Test Diff for    C61    --  C63     ..        6.5 s.u.
PLAT234_ALERT_4_C Large Hirshfeld Difference C6     --  C7      ..       0.17 Ang.
PLAT234_ALERT_4_C Large Hirshfeld Difference C12    --  C13     ..       0.18 Ang.
PLAT234_ALERT_4_C Large Hirshfeld Difference C20    --  C21     ..       0.16 Ang.
PLAT234_ALERT_4_C Large Hirshfeld Difference C25    --  C26     ..       0.19 Ang.
PLAT234_ALERT_4_C Large Hirshfeld Difference C33    --  C34     ..       0.16 Ang.
PLAT234_ALERT_4_C Large Hirshfeld Difference C55    --  C60     ..       0.16 Ang.
PLAT234_ALERT_4_C Large Hirshfeld Difference C66    --  C67     ..       0.19 Ang.
PLAT241_ALERT_2_C High   'MainMol' Ueq as Compared to Neighbors of        C13 Check
PLAT241_ALERT_2_C High   'MainMol' Ueq as Compared to Neighbors of        C61 Check
PLAT241_ALERT_2_C High   'MainMol' Ueq as Compared to Neighbors of        C73 Check
PLAT242_ALERT_2_C Low    'MainMol' Ueq as Compared to Neighbors of         O6 Check
```

```
PLAT242_ALERT_2_C Low    'MainMol' Ueq as Compared to Neighbors of        O14 Check
PLAT242_ALERT_2_C Low    'MainMol' Ueq as Compared to Neighbors of        O18 Check
PLAT242_ALERT_2_C Low    'MainMol' Ueq as Compared to Neighbors of        C25 Check
PLAT242_ALERT_2_C Low    'MainMol' Ueq as Compared to Neighbors of        C59 Check
PLAT243_ALERT_4_C High   'Solvent' Ueq as Compared to Neighbors of        C77 Check
PLAT244_ALERT_4_C Low    'Solvent' Ueq as Compared to Neighbors of        C75 Check
PLAT250_ALERT_2_C Large U3/U1 Ratio for Average U(i,j) Tensor ....        2.1 Note
PLAT342_ALERT_3_C Low Bond Precision on  C-C Bonds ...............    0.00878 Ang.
PLAT369_ALERT_2_C Long    C(sp2)-C(sp2) Bond  C10    -   C19    ..       1.55 Ang.
PLAT369_ALERT_2_C Long    C(sp2)-C(sp2) Bond  C33    -   C36    ..       1.54 Ang.
PLAT369_ALERT_2_C Long    C(sp2)-C(sp2) Bond  C41    -   C44    ..       1.53 Ang.
PLAT395_ALERT_2_C Deviating  X-O-Y  Angle from 120 Deg for     O23      156.8 Degree
```

Alert level G

```
FORMU01_ALERT_2_G  There is a discrepancy between the atom counts in the
            _chemical_formula_sum and the formula from the _atom_site* data.
            Atom count from _chemical_formula_sum:C78 H82 O26 P2 Zr3
            Atom count from the _atom_site data:  C78 H71 O26 P2 Zr3
CELLZ01_ALERT_1_G Difference between formula and atom_site contents detected.
CELLZ01_ALERT_1_G WARNING: H atoms missing from atom site list. Is this intentional?
           From the CIF: _cell_formula_units_Z    8
           From the CIF: _chemical_formula_sum  C78 H82 O26 P2 Zr3
           TEST: Compare cell contents of formula and atom_site data

           atom    Z*formula  cif sites diff
           C        624.00    624.00    0.00
           H        656.00    568.00   88.00
           O        208.00    208.00    0.00
           P         16.00     16.00    0.00
           Zr        24.00     24.00    0.00
PLAT002_ALERT_2_G Number of Distance or Angle Restraints on AtSite         32 Note
PLAT003_ALERT_2_G Number of Uiso or Uij Restrained non-H Atoms ...          7 Report
PLAT004_ALERT_5_G Polymeric Structure Found with Maximum Dimension          3 Info
PLAT005_ALERT_5_G No Embedded Refinement Details found in the CIF      Please Do !
PLAT033_ALERT_4_G Flack x Value Deviates > 3.0 * sigma from Zero .      0.138 Note
PLAT066_ALERT_1_G Predicted and Reported Tmin&Tmax Range Identical          ? Check
PLAT072_ALERT_2_G SHELXL First  Parameter in WGHT  Unusually Large       0.20 Report
PLAT232_ALERT_2_G Hirshfeld Test Diff (M-X)  Zr1    --  O5      ..       11.7 s.u.
PLAT232_ALERT_2_G Hirshfeld Test Diff (M-X)  Zr1    --  O17     ..        6.3 s.u.
PLAT232_ALERT_2_G Hirshfeld Test Diff (M-X)  Zr1    --  O9_c    ..       14.0 s.u.
PLAT232_ALERT_2_G Hirshfeld Test Diff (M-X)  Zr1    --  O15_g   ..        6.0 s.u.
PLAT232_ALERT_2_G Hirshfeld Test Diff (M-X)  Zr2    --  O6      ..       36.0 s.u.
PLAT232_ALERT_2_G Hirshfeld Test Diff (M-X)  Zr2    --  O14     ..       41.0 s.u.
PLAT232_ALERT_2_G Hirshfeld Test Diff (M-X)  Zr2    --  O8_b    ..       33.5 s.u.
PLAT232_ALERT_2_G Hirshfeld Test Diff (M-X)  Zr2    --  O10_c   ..       34.0 s.u.
PLAT232_ALERT_2_G Hirshfeld Test Diff (M-X)  Zr2    --  O16_g   ..       24.7 s.u.
PLAT232_ALERT_2_G Hirshfeld Test Diff (M-X)  Zr2    --  O2_i    ..       24.3 s.u.
PLAT232_ALERT_2_G Hirshfeld Test Diff (M-X)  Zr3    --  O13     ..        7.3 s.u.
PLAT232_ALERT_2_G Hirshfeld Test Diff (M-X)  Zr3    --  O7_b    ..        9.3 s.u.
PLAT232_ALERT_2_G Hirshfeld Test Diff (M-X)  Zr3    --  O1_i    ..        8.0 s.u.
PLAT344_ALERT_2_G Unusual sp?     Angle Range in Solvent/Ion for .     C78    Check
PLAT344_ALERT_2_G Unusual sp?     Angle Range in Solvent/Ion for .     C76    Check
PLAT380_ALERT_4_G Incorrectly? Oriented X(sp2)-Methyl Moiety .....        C18 Check
PLAT380_ALERT_4_G Incorrectly? Oriented X(sp2)-Methyl Moiety .....        C29 Check
PLAT380_ALERT_4_G Incorrectly? Oriented X(sp2)-Methyl Moiety .....        C54 Check
PLAT380_ALERT_4_G Incorrectly? Oriented X(sp2)-Methyl Moiety .....        C65 Check
PLAT764_ALERT_4_G Overcomplete CIF Bond List Detected (Rep/Expd) .       1.15 Ratio
PLAT779_ALERT_4_G Suspect or Irrelevant (Bond) Angle in CIF .... #         94 Check
              O1   -C1   -ZR3     1.555   1.555   8.645            44.20 Deg.
PLAT779_ALERT_4_G Suspect or Irrelevant (Bond) Angle in CIF .... #        164 Check
```

```
             O7   -C36  -ZR3     1.555   1.555   3.555             37.10 Deg.
PLAT779_ALERT_4_G Suspect or Irrelevant (Bond) Angle in CIF .... #        170 Check
             O9   -C37  -ZR1     1.555   1.555   4.655             37.50 Deg.
PLAT791_ALERT_4_G The Model has Chirality at P1      (Chiral SPGR)          S Verify
PLAT791_ALERT_4_G The Model has Chirality at P2      (Chiral SPGR)          R Verify
PLAT791_ALERT_4_G The Model has Chirality at C72     (Chiral SPGR)          R Verify
PLAT794_ALERT_5_G Tentative Bond Valency for Zr1     (IV)    .....       4.08 Note
PLAT794_ALERT_5_G Tentative Bond Valency for Zr2     (IV)    .....       2.11 Note
PLAT794_ALERT_5_G Tentative Bond Valency for Zr3     (IV)    .....       3.92 Note
PLAT860_ALERT_3_G Number of Least-Squares Restraints .............         79 Note
PLAT899_ALERT_4_G SHELXL97   is Deprecated and Succeeded by SHELXL       2014 Note
```

```
  2 ALERT level A = Most likely a serious problem - resolve or explain
 11 ALERT level B = A potentially serious problem, consider carefully
 46 ALERT level C = Check. Ensure it is not caused by an omission or oversight
 41 ALERT level G = General information/check it is not something unexpected

  7 ALERT type 1 CIF construction/syntax error, inconsistent or missing data
 59 ALERT type 2 Indicator that the structure model may be wrong or deficient
  7 ALERT type 3 Indicator that the structure quality may be low
 22 ALERT type 4 Improvement, methodology, query or suggestion
  5 ALERT type 5 Informative message, check
```

It is advisable to attempt to resolve as many as possible of the alerts in all categories. Often the minor alerts point to easily fixed oversights, errors and omissions in your CIF or refinement strategy, so attention to these fine details can be worthwhile. In order to resolve some of the more serious problems it may be necessary to carry out additional measurements or structure refinements. However, the purpose of your study may justify the reported deviations and the more serious of these should normally be commented upon in the discussion or experimental section of a paper or in the "special_details" fields of the CIF. checkCIF was carefully designed to identify outliers and unusual parameters, but every test has its limitations and alerts that are not important in a particular case may appear. Conversely, the absence of alerts does not guarantee there are no aspects of the results needing attention. It is up to the individual to critically assess their own results and, if necessary, seek expert advice.

Publication of your CIF in IUCr journals

A basic structural check has been run on your CIF. These basic checks will be run on all CIFs submitted for publication in IUCr journals (*Acta Crystallographica*, *Journal of Applied Crystallography*, *Journal of Synchrotron Radiation*); however, if you intend to submit to *Acta Crystallographica Section C* or *E*, you should make sure that full publication checks are run on the final version of your CIF prior to submission.

Publication of your CIF in other journals

Please refer to the *Notes for Authors* of the relevant journal for any special instructions relating to CIF submission.

PLATON version of 19/11/2015; check.def file version of 17/11/2015

Datablock 1-Zr - ellipsoid plot

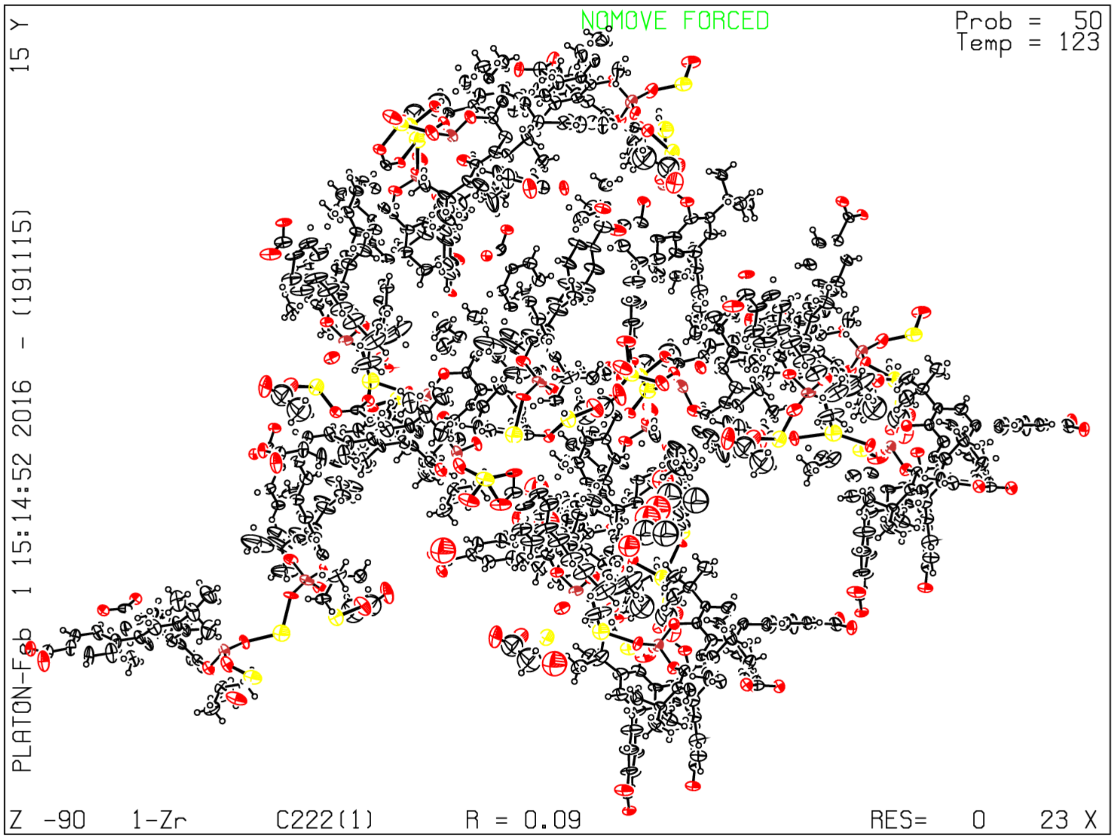

checkCIF/PLATON report

Structure factors have been supplied for datablock(s) me2l-mg

THIS REPORT IS FOR GUIDANCE ONLY. IF USED AS PART OF A REVIEW PROCEDURE FOR PUBLICATION, IT SHOULD NOT REPLACE THE EXPERTISE OF AN EXPERIENCED CRYSTALLOGRAPHIC REFEREE.

No syntax errors found. CIF dictionary Interpreting this report

Datablock: me2l-mg

```
Bond precision:  C-C = 0.0158 A          Wavelength=1.54178

Cell:            a=21.9287(12)     b=34.0887(18)     c=37.283(2)
                 alpha=90          beta=90           gamma=90
Temperature:     173 K
```

	Calculated	Reported
Volume	27870(3)	27870(3)
Space group	P 21 21 21	P 21 21 21
Hall group	P 2ac 2ab	P 2ac 2ab
Moiety formula	C228 H240 Mg3 O56 P6, 3(C4 O)	C228 H240 Mg3 O56 P6, 3(C4 O)
Sum formula	C240 H240 Mg3 O59 P6	C240 H240 Mg3 O59 P6
Mr	4327.08	4327.06
Dx,g cm-3	1.031	1.031
Z	4	4
Mu (mm-1)	0.973	0.973
F000	9112.0	9112.0
F000'	9149.05	
h,k,lmax	24,37,41	24,37,41
Nref	40418[21567]	39778
Tmin,Tmax	0.586,0.678	
Tmin'	0.437	

```
Correction method= Not given

Data completeness= 1.84/0.98      Theta(max)= 59.218

R(reflections)= 0.1313( 28098)    wR2(reflections)= 0.3439( 39778)

S = 1.244                   Npar= 2500
```

The following ALERTS were generated. Each ALERT has the format
test-name_ALERT_alert-type_alert-level.
Click on the hyperlinks for more details of the test.

Alert level A

```
PLAT602_ALERT_2_A VERY LARGE Solvent Accessible VOID(S) in Structure        ! Info
```

Alert level B

```
THETM01_ALERT_3_B  The value of sine(theta_max)/wavelength is less than 0.575
            Calculated sin(theta_max)/wavelength =    0.5572
PLAT035_ALERT_1_B _chemical_absolute_configuration info  Not given     Please Do !
PLAT340_ALERT_3_B Low Bond Precision on  C-C Bonds ...............    0.01578 Ang.
PLAT430_ALERT_2_B Short Inter D...A Contact  O37    ..  O47     ..       2.73 Ang.
PLAT987_ALERT_1_B The Flack x is >> 0 -  Do a BASF/TWIN Refinement     Please Check
```

Alert level C

```
DIFMX02_ALERT_1_C  The maximum difference density is > 0.1*ZMAX*0.75
            The relevant atom site should be identified.
PLAT018_ALERT_1_C _diffrn_measured_fraction_theta_max .NE. *_full           ! Check
PLAT057_ALERT_3_C Correction for Absorption Required   RT(exp) ...       1.16 Do !
PLAT082_ALERT_2_C High R1 Value ..................................       0.13 Report
PLAT084_ALERT_3_C High wR2 Value (i.e. > 0.25) ...................       0.34 Report
PLAT094_ALERT_2_C Ratio of Maximum / Minimum Residual Density ....       2.70 Report
PLAT097_ALERT_2_C Large Reported Max.  (Positive) Residual Density       1.49 eA-3
PLAT202_ALERT_3_C Isotropic non-H Atoms in Anion/Solvent .........         15 Check
PLAT220_ALERT_2_C Non-Solvent Resd 1   C   Ueq(max)/Ueq(min) Range        4.0 Ratio
PLAT220_ALERT_2_C Non-Solvent Resd 1   O   Ueq(max)/Ueq(min) Range        4.0 Ratio
PLAT222_ALERT_3_C Non-Solvent Resd 1   H Uiso(max)/Uiso(min) Range        4.6 Ratio
PLAT241_ALERT_2_C High   'MainMol' Ueq as Compared to Neighbors of        O59 Check
PLAT241_ALERT_2_C High   'MainMol' Ueq as Compared to Neighbors of       C183 Check
PLAT241_ALERT_2_C High   'MainMol' Ueq as Compared to Neighbors of       C186 Check
PLAT241_ALERT_2_C High   'MainMol' Ueq as Compared to Neighbors of       C187 Check
PLAT241_ALERT_2_C High   'MainMol' Ueq as Compared to Neighbors of       C195 Check
PLAT242_ALERT_2_C Low    'MainMol' Ueq as Compared to Neighbors of        O40 Check
PLAT242_ALERT_2_C Low    'MainMol' Ueq as Compared to Neighbors of        C17 Check
PLAT242_ALERT_2_C Low    'MainMol' Ueq as Compared to Neighbors of        C43 Check
PLAT242_ALERT_2_C Low    'MainMol' Ueq as Compared to Neighbors of        C64 Check
PLAT242_ALERT_2_C Low    'MainMol' Ueq as Compared to Neighbors of       C124 Check
PLAT242_ALERT_2_C Low    'MainMol' Ueq as Compared to Neighbors of       C132 Check
PLAT242_ALERT_2_C Low    'MainMol' Ueq as Compared to Neighbors of       C145 Check
PLAT242_ALERT_2_C Low    'MainMol' Ueq as Compared to Neighbors of       C182 Check
PLAT242_ALERT_2_C Low    'MainMol' Ueq as Compared to Neighbors of       C184 Check
PLAT242_ALERT_2_C Low    'MainMol' Ueq as Compared to Neighbors of       C201 Check
PLAT242_ALERT_2_C Low    'MainMol' Ueq as Compared to Neighbors of       C230 Check
PLAT242_ALERT_2_C Low    'MainMol' Ueq as Compared to Neighbors of       C231 Check
PLAT242_ALERT_2_C Low    'MainMol' Ueq as Compared to Neighbors of       C233 Check
PLAT243_ALERT_4_C High   'Solvent' Ueq as Compared to Neighbors of       C209 Check
PLAT243_ALERT_4_C High   'Solvent' Ueq as Compared to Neighbors of        O52 Check
PLAT244_ALERT_4_C Low    'Solvent' Ueq as Compared to Neighbors of        O48 Check
PLAT244_ALERT_4_C Low    'Solvent' Ueq as Compared to Neighbors of       C218 Check
PLAT244_ALERT_4_C Low    'Solvent' Ueq as Compared to Neighbors of       C227 Check
PLAT244_ALERT_4_C Low    'Solvent' Ueq as Compared to Neighbors of       C214 Check
PLAT430_ALERT_2_C Short Inter D...A Contact  O17    ..  O39     ..       2.88 Ang.
PLAT906_ALERT_3_C Large K value in the Analysis of Variance ......      2.640 Check
PLAT911_ALERT_3_C Missing # FCF Refl Between THmin & STh/L=  0.557        256 Report
PLAT918_ALERT_3_C Reflection(s) with I(obs) much Smaller I(calc) .         20 Check
PLAT934_ALERT_3_C Number of (Iobs-Icalc)/SigmaW > 10 Outliers ....          1 Check
PLAT939_ALERT_3_C Large Value of Not (SHELXL) Weight Optimized S .      34.67 Check
PLAT978_ALERT_2_C Number C-C Bonds with Positive Residual Density.          0 Info
```

Alert level G

```
PLAT002_ALERT_2_G Number of Distance or Angle Restraints on AtSite         42 Note
```

```
PLAT003_ALERT_2_G Number of Uiso or Uij Restrained non-H Atoms ...        308 Report
PLAT033_ALERT_4_G Flack x Value Deviates > 3.0 * sigma from Zero .      0.104 Note
PLAT063_ALERT_4_G Crystal Size Likely too Large for Beam Size ....       0.80 mm
PLAT072_ALERT_2_G SHELXL First  Parameter in WGHT  Unusually Large       0.20 Report
PLAT172_ALERT_4_G The CIF-Embedded .res File Contains DFIX Records         19 Report
PLAT174_ALERT_4_G The CIF-Embedded .res File Contains FLAT Records          4 Report
PLAT177_ALERT_4_G The CIF-Embedded .res File Contains DELU Records          1 Report
PLAT178_ALERT_4_G The CIF-Embedded .res File Contains SIMU Records          1 Report
PLAT344_ALERT_2_G Unusual sp?     Angle Range in Solvent/Ion for .       C209 Check
PLAT344_ALERT_2_G Unusual sp?     Angle Range in Solvent/Ion for .       C216 Check
PLAT344_ALERT_2_G Unusual sp?     Angle Range in Solvent/Ion for .       C220 Check
PLAT344_ALERT_2_G Unusual sp?     Angle Range in Solvent/Ion for .       C221 Check
PLAT344_ALERT_2_G Unusual sp?     Angle Range in Solvent/Ion for .       C218 Check
PLAT344_ALERT_2_G Unusual sp?     Angle Range in Solvent/Ion for .       C227 Check
PLAT344_ALERT_2_G Unusual sp?     Angle Range in Solvent/Ion for .       C242 Check
PLAT344_ALERT_2_G Unusual sp?     Angle Range in Solvent/Ion for .       C243 Check
PLAT344_ALERT_2_G Unusual sp?     Angle Range in Solvent/Ion for .       C180 Check
PLAT344_ALERT_2_G Unusual sp?     Angle Range in Solvent/Ion for .       C214 Check
PLAT344_ALERT_2_G Unusual sp?     Angle Range in Solvent/Ion for .       C222 Check
PLAT344_ALERT_2_G Unusual sp?     Angle Range in Solvent/Ion for .       C241 Check
PLAT367_ALERT_2_G Long?   C(sp?)-C(sp?) Bond  C209   -   C221   ..       1.53 Ang.
PLAT367_ALERT_2_G Long?   C(sp?)-C(sp?) Bond  C227   -   C243   ..       1.65 Ang.
PLAT367_ALERT_2_G Long?   C(sp?)-C(sp?) Bond  C180   -   C241   ..       1.53 Ang.
PLAT367_ALERT_2_G Long?   C(sp?)-C(sp?) Bond  C214   -   C222   ..       1.54 Ang.
PLAT380_ALERT_4_G Incorrectly? Oriented X(sp2)-Methyl Moiety .....       C191 Check
PLAT395_ALERT_2_G Deviating  X-O-Y  Angle from 120 Deg for  O1          120.0 Degree
PLAT395_ALERT_2_G Deviating  X-O-Y  Angle from 120 Deg for  O3          122.0 Degree
PLAT395_ALERT_2_G Deviating  X-O-Y  Angle from 120 Deg for  O5          123.0 Degree
PLAT395_ALERT_2_G Deviating  X-O-Y  Angle from 120 Deg for  O6          124.0 Degree
PLAT395_ALERT_2_G Deviating  X-O-Y  Angle from 120 Deg for  O9          121.1 Degree
PLAT395_ALERT_2_G Deviating  X-O-Y  Angle from 120 Deg for  O10         124.3 Degree
PLAT395_ALERT_2_G Deviating  X-O-Y  Angle from 120 Deg for  O11         120.2 Degree
PLAT395_ALERT_2_G Deviating  X-O-Y  Angle from 120 Deg for  O14         122.3 Degree
PLAT395_ALERT_2_G Deviating  X-O-Y  Angle from 120 Deg for  O16         118.2 Degree
PLAT395_ALERT_2_G Deviating  X-O-Y  Angle from 120 Deg for  O18         124.5 Degree
PLAT395_ALERT_2_G Deviating  X-O-Y  Angle from 120 Deg for  O21         125.9 Degree
PLAT395_ALERT_2_G Deviating  X-O-Y  Angle from 120 Deg for  O22         116.5 Degree
PLAT398_ALERT_2_G Deviating  C-O-C  Angle from 120 Deg for O58          107.3 Degree
PLAT398_ALERT_2_G Deviating  C-O-C  Angle from 120 Deg for O55          104.9 Degree
PLAT720_ALERT_4_G Number of Unusual/Non-Standard Labels ..........          1 Note
PLAT860_ALERT_3_G Number of Least-Squares Restraints .............       2750 Note
PLAT909_ALERT_3_G Percentage of I>2sig(I) Data at Theta(Max) Still        37% Note
PLAT910_ALERT_3_G Missing # of FCF Reflection(s) Below Theta(Min).          1 Note
PLAT913_ALERT_3_G Missing # of Very Strong Reflections in FCF ....          1 Note
PLAT933_ALERT_2_G Number of OMIT Records in Embedded .res File ...         22 Note
```

---

1 **ALERT level A** = Most likely a serious problem - resolve or explain
5 **ALERT level B** = A potentially serious problem, consider carefully
42 **ALERT level C** = Check. Ensure it is not caused by an omission or oversight
46 **ALERT level G** = General information/check it is not something unexpected

4 ALERT type 1 CIF construction/syntax error, inconsistent or missing data
61 ALERT type 2 Indicator that the structure model may be wrong or deficient
15 ALERT type 3 Indicator that the structure quality may be low
14 ALERT type 4 Improvement, methodology, query or suggestion
0 ALERT type 5 Informative message, check

It is advisable to attempt to resolve as many as possible of the alerts in all categories. Often the minor alerts point to easily fixed oversights, errors and omissions in your CIF or refinement strategy, so attention to these fine details can be worthwhile. In order to resolve some of the more serious problems it may be necessary to carry out additional measurements or structure refinements. However, the purpose of your study may justify the reported deviations and the more serious of these should normally be commented upon in the discussion or experimental section of a paper or in the "special_details" fields of the CIF. checkCIF was carefully designed to identify outliers and unusual parameters, but every test has its limitations and alerts that are not important in a particular case may appear. Conversely, the absence of alerts does not guarantee there are no aspects of the results needing attention. It is up to the individual to critically assess their own results and, if necessary, seek expert advice.

Publication of your CIF in IUCr journals

A basic structural check has been run on your CIF. These basic checks will be run on all CIFs submitted for publication in IUCr journals (*Acta Crystallographica*, *Journal of Applied Crystallography*, *Journal of Synchrotron Radiation*); however, if you intend to submit to *Acta Crystallographica Section C* or *E* or *IUCrData*, you should make sure that full publication checks are run on the final version of your CIF prior to submission.

Publication of your CIF in other journals

Please refer to the *Notes for Authors* of the relevant journal for any special instructions relating to CIF submission.

PLATON version of 13/08/2017; check.def file version of 27/07/2017

Datablock me2l-mg - ellipsoid plot

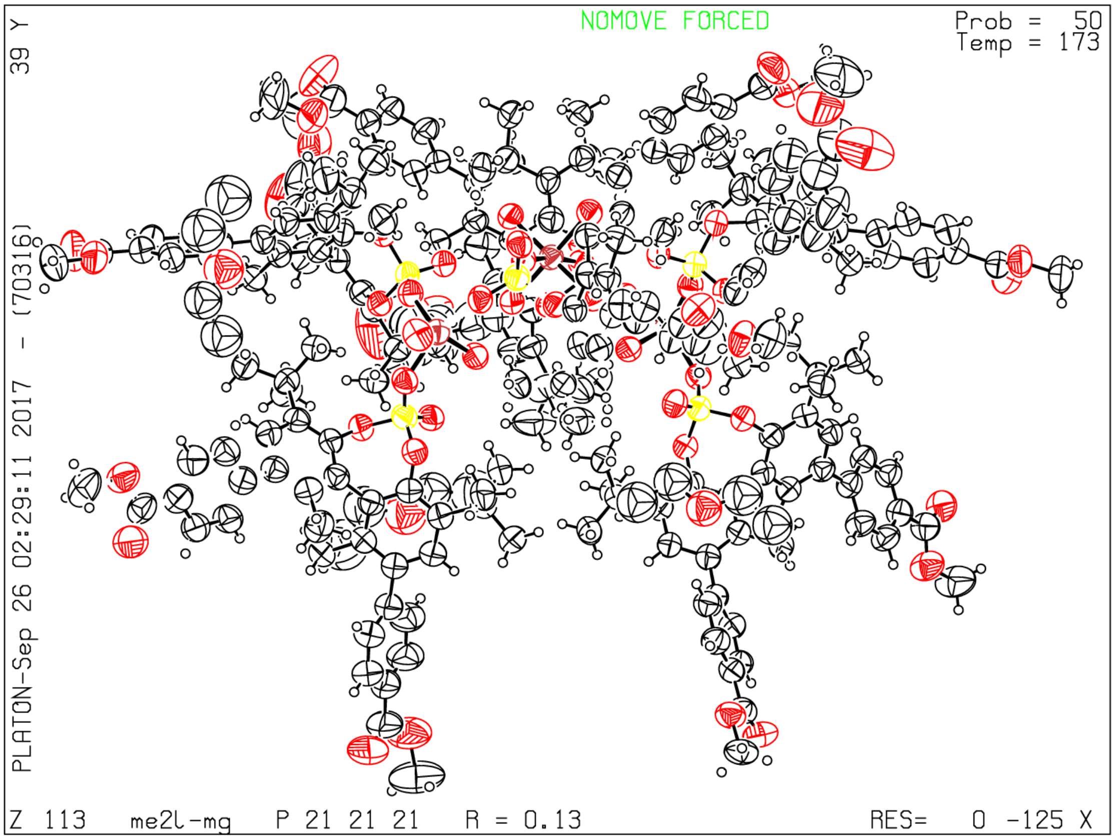

checkCIF/PLATON report

Structure factors have been supplied for datablock(s) me2l-mn

THIS REPORT IS FOR GUIDANCE ONLY. IF USED AS PART OF A REVIEW PROCEDURE FOR PUBLICATION, IT SHOULD NOT REPLACE THE EXPERTISE OF AN EXPERIENCED CRYSTALLOGRAPHIC REFEREE.

No syntax errors found. CIF dictionary Interpreting this report

Datablock: me2l-mn

Bond precision: C-C = 0.0242 A Wavelength=1.54178

Cell:	a=22.1113(18)	b=33.662(3)	c=37.400(3)
	alpha=90	beta=90	gamma=90
Temperature:	173 K		

	Calculated	Reported
Volume	27837(4)	27837(4)
Space group	P 21 21 21	P 21 21 21
Hall group	P 2ac 2ab	P 2ac 2ab
Moiety formula	C228 H240 Mn3 O56 P6, C6 O, 2(C4 O)	C228 H240 Mn3 O56 P6, C6 O, 2(C4 O)
Sum formula	C242 H240 Mn3 O59 P6	C242 H240 Mn3 O59 P6
Mr	4442.97	4442.97
Dx,g cm-3	1.060	1.060
Z	4	4
Mu (mm-1)	1.968	1.968
F000	9316.0	9316.0
F000’	9344.99	
h,k,lmax	22,34,38	22,34,38
Nref	32225[17274]	31396
Tmin,Tmax	0.517,0.675	
Tmin’	0.356	

Correction method= Not given

Data completeness= 1.82/0.97 Theta(max)= 52.873

R(reflections)= 0.1391(26703) wR2(reflections)= 0.3769(31396)

S = 1.623 Npar= 2580

The following ALERTS were generated. Each ALERT has the format
test-name_ALERT_alert-type_alert-level.
Click on the hyperlinks for more details of the test.

Alert level A

```
THETM01_ALERT_3_A  The value of sine(theta_max)/wavelength is less than 0.550
            Calculated sin(theta_max)/wavelength =    0.5171
PLAT413_ALERT_2_A Short Inter XH3 .. XHn     H3LA   ..  H3UA    ..       1.85 Ang.
PLAT602_ALERT_2_A VERY LARGE Solvent Accessible VOID(S) in Structure        ! Info
```

Alert level B

```
PLAT035_ALERT_1_B _chemical_absolute_configuration info  Not given     Please Do !
PLAT057_ALERT_3_B Correction for Absorption Required   RT(exp) ...       1.30 Do !
PLAT084_ALERT_3_B High wR2 Value (i.e. > 0.25) ...................       0.38 Report
PLAT201_ALERT_2_B Isotropic non-H Atoms in Main Residue(s) .......          2 Report
PLAT242_ALERT_2_B Low    'MainMol' Ueq as Compared to Neighbors of        O58 Check
PLAT341_ALERT_3_B Low Bond Precision on  C-C Bonds ..............    0.02425 Ang.
PLAT413_ALERT_2_B Short Inter XH3 .. XHn     H7IA   ..  H7UA    ..       1.97 Ang.
PLAT430_ALERT_2_B Short Inter D...A Contact  O40    ..  O55     ..       2.78 Ang.
PLAT934_ALERT_3_B Number of (Iobs-Icalc)/SigmaW > 10 Outliers ....         10 Check
PLAT987_ALERT_1_B The Flack x is >> 0 -  Do a BASF/TWIN Refinement     Please Check
```

Alert level C

```
PLAT018_ALERT_1_C _diffrn_measured_fraction_theta_max .NE. *_full          ! Check
PLAT082_ALERT_2_C High R1 Value ..................................       0.14 Report
PLAT090_ALERT_3_C Poor Data / Parameter Ratio (Zmax > 18) ........       6.62 Note
PLAT202_ALERT_3_C Isotropic non-H Atoms in Anion/Solvent .........         17 Check
PLAT220_ALERT_2_C Non-Solvent Resd 1   C   Ueq(max)/Ueq(min) Range        4.9 Ratio
PLAT220_ALERT_2_C Non-Solvent Resd 1   O   Ueq(max)/Ueq(min) Range        4.7 Ratio
PLAT222_ALERT_3_C Non-Solvent Resd 1   H Uiso(max)/Uiso(min) Range        5.7 Ratio
PLAT241_ALERT_2_C High   'MainMol' Ueq as Compared to Neighbors of         C9 Check
PLAT241_ALERT_2_C High   'MainMol' Ueq as Compared to Neighbors of        C48 Check
PLAT241_ALERT_2_C High   'MainMol' Ueq as Compared to Neighbors of       C101 Check
PLAT241_ALERT_2_C High   'MainMol' Ueq as Compared to Neighbors of       C102 Check
PLAT241_ALERT_2_C High   'MainMol' Ueq as Compared to Neighbors of       C114 Check
PLAT241_ALERT_2_C High   'MainMol' Ueq as Compared to Neighbors of       C153 Check
PLAT241_ALERT_2_C High   'MainMol' Ueq as Compared to Neighbors of       C170 Check
PLAT241_ALERT_2_C High   'MainMol' Ueq as Compared to Neighbors of       C199 Check
PLAT241_ALERT_2_C High   'MainMol' Ueq as Compared to Neighbors of       C201 Check
PLAT242_ALERT_2_C Low    'MainMol' Ueq as Compared to Neighbors of        Mn1 Check
PLAT242_ALERT_2_C Low    'MainMol' Ueq as Compared to Neighbors of        Mn3 Check
PLAT242_ALERT_2_C Low    'MainMol' Ueq as Compared to Neighbors of        O48 Check
PLAT242_ALERT_2_C Low    'MainMol' Ueq as Compared to Neighbors of        C24 Check
PLAT242_ALERT_2_C Low    'MainMol' Ueq as Compared to Neighbors of        C40 Check
PLAT242_ALERT_2_C Low    'MainMol' Ueq as Compared to Neighbors of       C100 Check
PLAT242_ALERT_2_C Low    'MainMol' Ueq as Compared to Neighbors of       C103 Check
PLAT242_ALERT_2_C Low    'MainMol' Ueq as Compared to Neighbors of       C104 Check
PLAT242_ALERT_2_C Low    'MainMol' Ueq as Compared to Neighbors of       C136 Check
PLAT242_ALERT_2_C Low    'MainMol' Ueq as Compared to Neighbors of       C167 Check
PLAT242_ALERT_2_C Low    'MainMol' Ueq as Compared to Neighbors of       C174 Check
PLAT242_ALERT_2_C Low    'MainMol' Ueq as Compared to Neighbors of       C200 Check
PLAT242_ALERT_2_C Low    'MainMol' Ueq as Compared to Neighbors of       C209 Check
PLAT242_ALERT_2_C Low    'MainMol' Ueq as Compared to Neighbors of       C219 Check
PLAT242_ALERT_2_C Low    'MainMol' Ueq as Compared to Neighbors of       C230 Check
PLAT243_ALERT_4_C High   'Solvent' Ueq as Compared to Neighbors of       C125 Check
PLAT244_ALERT_4_C Low    'Solvent' Ueq as Compared to Neighbors of       C148 Check
PLAT244_ALERT_4_C Low    'Solvent' Ueq as Compared to Neighbors of       C162 Check
PLAT244_ALERT_4_C Low    'Solvent' Ueq as Compared to Neighbors of       C129 Check
PLAT244_ALERT_4_C Low    'Solvent' Ueq as Compared to Neighbors of        O51 Check
PLAT244_ALERT_4_C Low    'Solvent' Ueq as Compared to Neighbors of       C183 Check
PLAT309_ALERT_2_C Single Bonded Oxygen (C-O > 1.3 Ang) ..........       O0AA Check
PLAT334_ALERT_2_C Small Average Benzene  C-C Dist. C37    -C81           1.37 Ang.
PLAT361_ALERT_2_C Long   C(sp3)-C(sp3) Bond   C158   -   C231   ..       1.66 Ang.
```

```
PLAT369_ALERT_2_C Long    C(sp2)-C(sp2) Bond  C30    -   C40    ..       1.53 Ang.
PLAT369_ALERT_2_C Long    C(sp2)-C(sp2) Bond  C46    -   C209   ..       1.54 Ang.
PLAT369_ALERT_2_C Long    C(sp2)-C(sp2) Bond  C117   -   C215   ..       1.53 Ang.
PLAT369_ALERT_2_C Long    C(sp2)-C(sp2) Bond  C147   -   C219   ..       1.53 Ang.
PLAT369_ALERT_2_C Long    C(sp2)-C(sp2) Bond  C181   -   C205   ..       1.55 Ang.
PLAT412_ALERT_2_C Short Intra XH3 .. XHn     H3BA   ..  H5MA    ..       1.84 Ang.
PLAT412_ALERT_2_C Short Intra XH3 .. XHn     H9DA   ..  H8KA    ..       1.85 Ang.
PLAT413_ALERT_2_C Short Inter XH3 .. XHn     H0UA   ..  H2UA    ..       2.14 Ang.
PLAT430_ALERT_2_C Short Inter D...A Contact  O10    ..  O34     ..       2.88 Ang.
PLAT430_ALERT_2_C Short Inter D...A Contact  O32    ..  O50     ..       2.85 Ang.
PLAT906_ALERT_3_C Large K value in the Analysis of Variance ......      2.273 Check
PLAT911_ALERT_3_C Missing # FCF Refl Between THmin & STh/L=  0.517        201 Report
PLAT913_ALERT_3_C Missing # of Very Strong Reflections in FCF ....          4 Note
PLAT918_ALERT_3_C Reflection(s) with I(obs) much Smaller I(calc) .         13 Check
PLAT939_ALERT_3_C Large Value of Not (SHELXL) Weight Optimized S .      10.01 Check
PLAT977_ALERT_2_C Check the Negative Difference Density on    H2DA      -0.34 eA-3
PLAT977_ALERT_2_C Check the Negative Difference Density on    H0UA      -0.37 eA-3
PLAT978_ALERT_2_C Number C-C Bonds with Positive Residual Density.          0 Info
```

Alert level G

```
PLAT002_ALERT_2_G Number of Distance or Angle Restraints on AtSite         62 Note
PLAT003_ALERT_2_G Number of Uiso or Uij Restrained non-H Atoms ...        313 Report
PLAT033_ALERT_4_G Flack x Value Deviates > 3.0 * sigma from Zero .      0.112 Note
PLAT072_ALERT_2_G SHELXL First  Parameter in WGHT  Unusually Large       0.20 Report
PLAT172_ALERT_4_G The CIF-Embedded .res File Contains DFIX Records         38 Report
PLAT174_ALERT_4_G The CIF-Embedded .res File Contains FLAT Records          4 Report
PLAT176_ALERT_4_G The CIF-Embedded .res File Contains SADI Records          2 Report
PLAT178_ALERT_4_G The CIF-Embedded .res File Contains SIMU Records          1 Report
PLAT187_ALERT_4_G The CIF-Embedded .res File Contains RIGU Records          1 Report
PLAT301_ALERT_3_G Main Residue  Disorder .............(Resd  1)..         1% Note
PLAT335_ALERT_2_G Check Large C6 Ring    C-C Range C22    -C134          0.19 Ang.
PLAT344_ALERT_2_G Unusual sp?     Angle Range in Solvent/Ion for .       C148 Check
PLAT344_ALERT_2_G Unusual sp?     Angle Range in Solvent/Ion for .       C162 Check
PLAT344_ALERT_2_G Unusual sp?     Angle Range in Solvent/Ion for .       C169 Check
PLAT344_ALERT_2_G Unusual sp?     Angle Range in Solvent/Ion for .       C177 Check
PLAT344_ALERT_2_G Unusual sp?     Angle Range in Solvent/Ion for .       C235 Check
PLAT344_ALERT_2_G Unusual sp?     Angle Range in Solvent/Ion for .       C236 Check
PLAT344_ALERT_2_G Unusual sp?     Angle Range in Solvent/Ion for .       C129 Check
PLAT344_ALERT_2_G Unusual sp?     Angle Range in Solvent/Ion for .       C130 Check
PLAT344_ALERT_2_G Unusual sp?     Angle Range in Solvent/Ion for .       C178 Check
PLAT344_ALERT_2_G Unusual sp?     Angle Range in Solvent/Ion for .       C237 Check
PLAT344_ALERT_2_G Unusual sp?     Angle Range in Solvent/Ion for .       C125 Check
PLAT344_ALERT_2_G Unusual sp?     Angle Range in Solvent/Ion for .       C183 Check
PLAT344_ALERT_2_G Unusual sp?     Angle Range in Solvent/Ion for .       C239 Check
PLAT344_ALERT_2_G Unusual sp?     Angle Range in Solvent/Ion for .       C240 Check
PLAT367_ALERT_2_G Long?   C(sp?)-C(sp?) Bond  C148   -   C235   ..       1.52 Ang.
PLAT367_ALERT_2_G Long?   C(sp?)-C(sp?) Bond  C148   -   C236   ..       1.51 Ang.
PLAT367_ALERT_2_G Long?   C(sp?)-C(sp?) Bond  C162   -   C169   ..       1.51 Ang.
PLAT367_ALERT_2_G Long?   C(sp?)-C(sp?) Bond  C130   -   C237   ..       1.57 Ang.
PLAT380_ALERT_4_G Incorrectly? Oriented X(sp2)-Methyl Moiety .....       C141 Check
PLAT395_ALERT_2_G Deviating  X-O-Y  Angle from 120 Deg for  O1          121.4 Degree
PLAT395_ALERT_2_G Deviating  X-O-Y  Angle from 120 Deg for  O3          121.4 Degree
PLAT395_ALERT_2_G Deviating  X-O-Y  Angle from 120 Deg for  O5          120.2 Degree
PLAT395_ALERT_2_G Deviating  X-O-Y  Angle from 120 Deg for  O7          122.1 Degree
PLAT395_ALERT_2_G Deviating  X-O-Y  Angle from 120 Deg for  O13         120.4 Degree
PLAT395_ALERT_2_G Deviating  X-O-Y  Angle from 120 Deg for  O14         116.0 Degree
PLAT395_ALERT_2_G Deviating  X-O-Y  Angle from 120 Deg for  O15         121.9 Degree
PLAT395_ALERT_2_G Deviating  X-O-Y  Angle from 120 Deg for  O16         118.6 Degree
PLAT395_ALERT_2_G Deviating  X-O-Y  Angle from 120 Deg for  O18         125.0 Degree
PLAT395_ALERT_2_G Deviating  X-O-Y  Angle from 120 Deg for  O25         122.6 Degree
```

```
PLAT395_ALERT_2_G Deviating  X-O-Y  Angle from 120 Deg for  O26         119.7 Degree
PLAT395_ALERT_2_G Deviating  X-O-Y  Angle from 120 Deg for  O35         122.4 Degree
PLAT398_ALERT_2_G Deviating  C-O-C  Angle from 120 Deg for O57          109.0 Degree
PLAT398_ALERT_2_G Deviating  C-O-C  Angle from 120 Deg for O58          105.9 Degree
PLAT398_ALERT_2_G Deviating  C-O-C  Angle from 120 Deg for O1AA         134.2 Degree
PLAT720_ALERT_4_G Number of Unusual/Non-Standard Labels ..........        246 Note
PLAT791_ALERT_4_G The Model has Chirality at P3      (Chiral SPGR)          S Verify
PLAT794_ALERT_5_G Tentative Bond Valency for Mn2     (I)     .....       0.57 Info
PLAT860_ALERT_3_G Number of Least-Squares Restraints .............       4462 Note
PLAT909_ALERT_3_G Percentage of I>2sig(I) Data at Theta(Max) Still        50% Note
PLAT910_ALERT_3_G Missing # of FCF Reflection(s) Below Theta(Min).          1 Note
```

```
   3 ALERT level A = Most likely a serious problem - resolve or explain
  10 ALERT level B = A potentially serious problem, consider carefully
  58 ALERT level C = Check. Ensure it is not caused by an omission or oversight
  51 ALERT level G = General information/check it is not something unexpected

   3 ALERT type 1 CIF construction/syntax error, inconsistent or missing data
  86 ALERT type 2 Indicator that the structure model may be wrong or deficient
  17 ALERT type 3 Indicator that the structure quality may be low
  15 ALERT type 4 Improvement, methodology, query or suggestion
   1 ALERT type 5 Informative message, check
```

It is advisable to attempt to resolve as many as possible of the alerts in all categories. Often the minor alerts point to easily fixed oversights, errors and omissions in your CIF or refinement strategy, so attention to these fine details can be worthwhile. In order to resolve some of the more serious problems it may be necessary to carry out additional measurements or structure refinements. However, the purpose of your study may justify the reported deviations and the more serious of these should normally be commented upon in the discussion or experimental section of a paper or in the "special_details" fields of the CIF. checkCIF was carefully designed to identify outliers and unusual parameters, but every test has its limitations and alerts that are not important in a particular case may appear. Conversely, the absence of alerts does not guarantee there are no aspects of the results needing attention. It is up to the individual to critically assess their own results and, if necessary, seek expert advice.

Publication of your CIF in IUCr journals

A basic structural check has been run on your CIF. These basic checks will be run on all CIFs submitted for publication in IUCr journals (*Acta Crystallographica*, *Journal of Applied Crystallography*, *Journal of Synchrotron Radiation*); however, if you intend to submit to *Acta Crystallographica Section C* or *E* or *IUCrData*, you should make sure that full publication checks are run on the final version of your CIF prior to submission.

Publication of your CIF in other journals

Please refer to the *Notes for Authors* of the relevant journal for any special instructions relating to CIF submission.

PLATON version of 13/08/2017; check.def file version of 27/07/2017

Datablock me2l-mn - ellipsoid plot

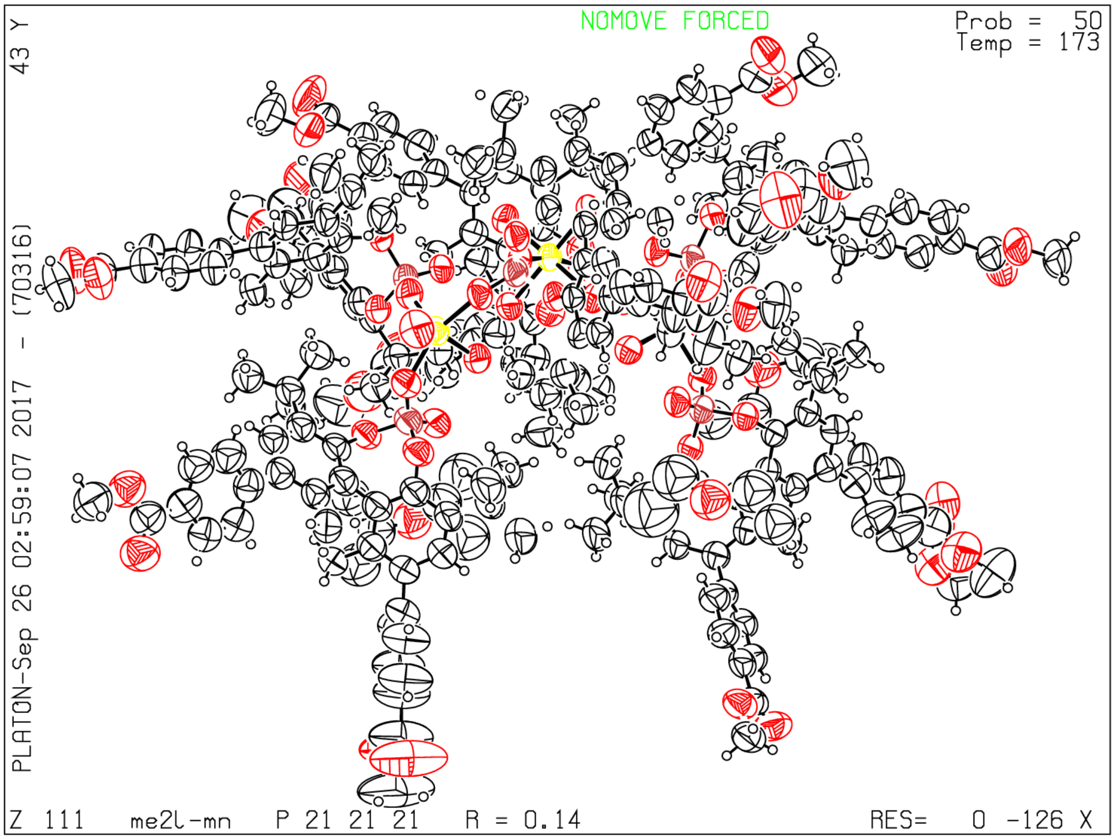

Supplement: Supplementary file 5 — Supplementary Data 2 [file 41467_2017_2335_MOESM5_ESM.pdf]
